# Bidirectional Electron Transfer Strategies for Anti-Markovnikov Olefin Aminofunctionalization via Arylamine Radicals

**DOI:** 10.1021/acscatal.4c04110

**Published:** 2024-08-17

**Authors:** Pritam Roychowdhury, Samya Samanta, Lauren M. Brown, Saim Waheed, David C. Powers

**Affiliations:** Department of Chemistry, Texas A&M University, College Station, Texas 77843, United States

**Keywords:** arylamine radicals, *N*-aminopyridinium salts, olefin difunctionalization, synthetic methods, anti-Markovnikov addition

## Abstract

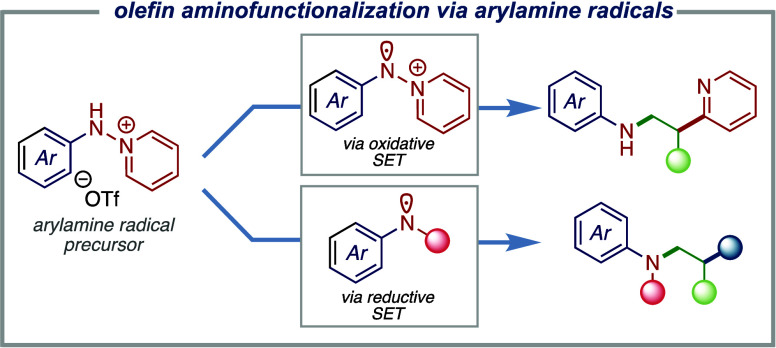

Arylamines are common structural motifs in pharmaceuticals, natural products, and materials precursors. While olefin aminofunctionalization chemistry can provide entry to arylamines, classical polar reactions typically afford Markovnikov products. Nitrogen-centered radical intermediates provide the opportunity to access anti-Markovnikov selectivity; however, anti-Markovnikov arylamination is unknown in large part due to the lack of arylamine radical precursors. Here, we introduce bidirectional electron transfer processes to generate arylamine radical intermediates from *N*-pyridinium arylamines: Single-electron oxidation provides arylamine radicals that engage in anti-Markovnikov olefin aminopyridylation; single-electron reduction unveils arylamine radicals that engage in anti-Markovnikov olefin aminofunctionalization. The development of bidirectional redox processes complements classical design principles for radical precursors, which typically function via a single redox manifold. Demonstration of both oxidative and reductive mechanisms to generate arylamine radicals from a common *N*-aminopyridinium precursor provides complementary methods to rapidly construct and diversify arylamine scaffolds from readily available radical precursors.

## Introduction

**Figure 1:**
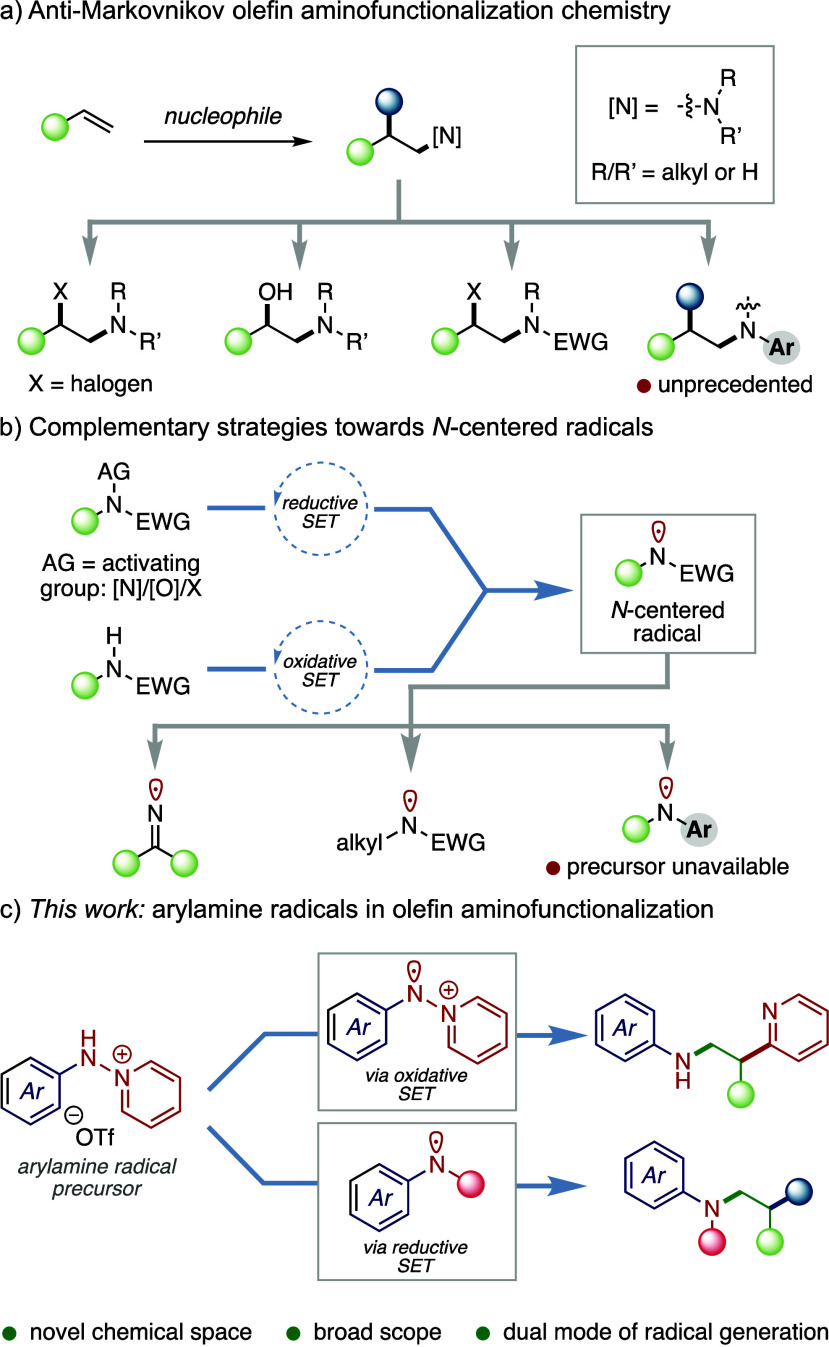
(a) Anti-Markovnikov olefin aminofunctionalization methods with arylamines remain a significant challenge. (b) Precursors for *N*-arylamine radicals are not generally available, which prevents development of olefin functionalization chemistry with this important fragment. (c) Here, we demonstrate *N*-aryl-*N-*aminopyridinium salts as an efficient *N*-centered arylamine radical precursor (generated via oxidative and reductive SET) for intermolecular olefin aminofunctionalization.

Arylamines are important structural motifs in molecules at all levels of the organic chemistry value chain, from pharmaceuticals and biologically active compounds to materials and polymers.^1^ Given the widespread availability of olefinic substrates, alkene functionalization chemistry represents a powerful potential disconnection toward functionalized arylamines.^2^ In general, polar reaction mechanisms for 1,2-aminofunctionalization—including oxyamination,^3−5^ carboamination,^6−9^ and amino halogenation^10−15^—proceed with Markovnikov selectivity. In contrast, for many functionalization reactions, achieving anti-Markovnikov selectivity remains a challenge.^16,17^*N*-centered radical intermediates provide the potential to access complementary anti-Markovnikov olefin functionalization products.^18^ While anti-Markovnikov olefin addition reactions via *N*-centered radicals have been developed,^19−25^ the lack of general precursors for arylamine radicals has prevented translation of these methods to the construction of functional aryl amines (Figure 1a).

Two general strategies have been developed to access *N*-centered radical intermediates: (i) One-electron oxidation of a negatively charged or H-bond-activated precursor (normally containing an electron-withdrawing group, EWG) to depress the N–H p*K*_a_ and (ii) one-electron reduction of the redox-active N–X bond (X = halogen, [O], [N]; Figure 1b).^26−32^ In general, radical precursors engage in one of these reactivity paradigms to unveil radical intermediates. We envisioned that the development of bidirectional electron transfer chemistry from a common precursor containing both an electron-withdrawing and a redox-active *N*-substituent would facilitate complementary redox strategies to access *N-*centered radicals for olefin functionalization chemistry.

Here, we introduce *N*-aryl-*N*-aminopyridinium salts as bidirectional radical precursors: Both oxidative and reductive processes afford *N*-arylamine radicals that can be used in olefin functionalization chemistry. In the oxidative manifold, pyridinium ylides, accessed by *in situ* deprotonation of *N*-aryl-*N*-aminopyridinium salts under mild conditions,^33^ undergo one-electron oxidation to generate arylamine radicals. Subsequent [3 + 2] cycloaddition between these transient arylamine radicals and olefinic partners affords the products of 1,2-aminopyridylation (Figure 1c). In the reductive manifold, one-electron reduction of *N*-aminopyridinium salts leads to cleavage of the N–N bond and the formation of arylamine radicals poised to participate in anti-Markovnikov 1,2-aminofunctionalization chemistry. The generation of *N*-centered radicals via both oxidative and reductive activation modes is confirmed by spin-trapped electron paramagnetic resonance (EPR) spectroscopy. These results leverage the inherent bifunctionality of *N*-aminopyridinium salts by harnessing both oxidative and reductive pathways for the generation of *N*-centered radicals,^34−36^ thereby expanding the scope and accessible selectivity of olefin aminofunctionalization.

## Results and Discussion

We initiated the development of bidirectional electron transfer strategies by examining oxidative radical generation from *N*-aryl-*N*-aminopyridinium precursors. Recognizing the widespread utility and predictable regioselectivity of 1,3-dipolar cycloadditions to C–C π-bonds,^37−39^ we envisioned that the one-electron oxidation of pyridinium ylides, generated by deprotonation of *N*-aminopyridinium salts with an appropriate base, would provide arylamine radicals poised to engage in rapid cycloaddition with olefin **2**. If the initial oxidation was affected by a photocatalyst excited state ([PC]*), then the reduction of the cycloadduct by the reduced photocatalyst [PC]^−^ would ultimately afford C2-pyridyl β-amino compounds, which are frequently encountered structural motifs in drug discovery^40,41^ and materials science.^42,43^ This sequence of events would comprise an atom-economical olefin aminopyridylation reaction in which both the amino and pyridine components of **1** are incorporated into reaction product **3**. As such, the method would complement existing olefin aminopyridylation chemistry, which is limited to electron-withdrawing *N-*substituents.^44^

In furtherance of the targeted olefin aminopyridylation chemistry, we identified the combination of **1a**, tetramethylethylenediamine (TMEDA), and Ru(bpy)_3_Cl_2_·6H_2_O effects of the aminopyridylation of methyl acrylate (**2a**) to afford compound **3a** in 88% yield (see the Supporting Information, Section B.1 for optimization details). The same chemistry can be accessed in the absence of an exogenous base if **1a** is replaced by pyridinium ylide **1a′**.

**Figure 2:**
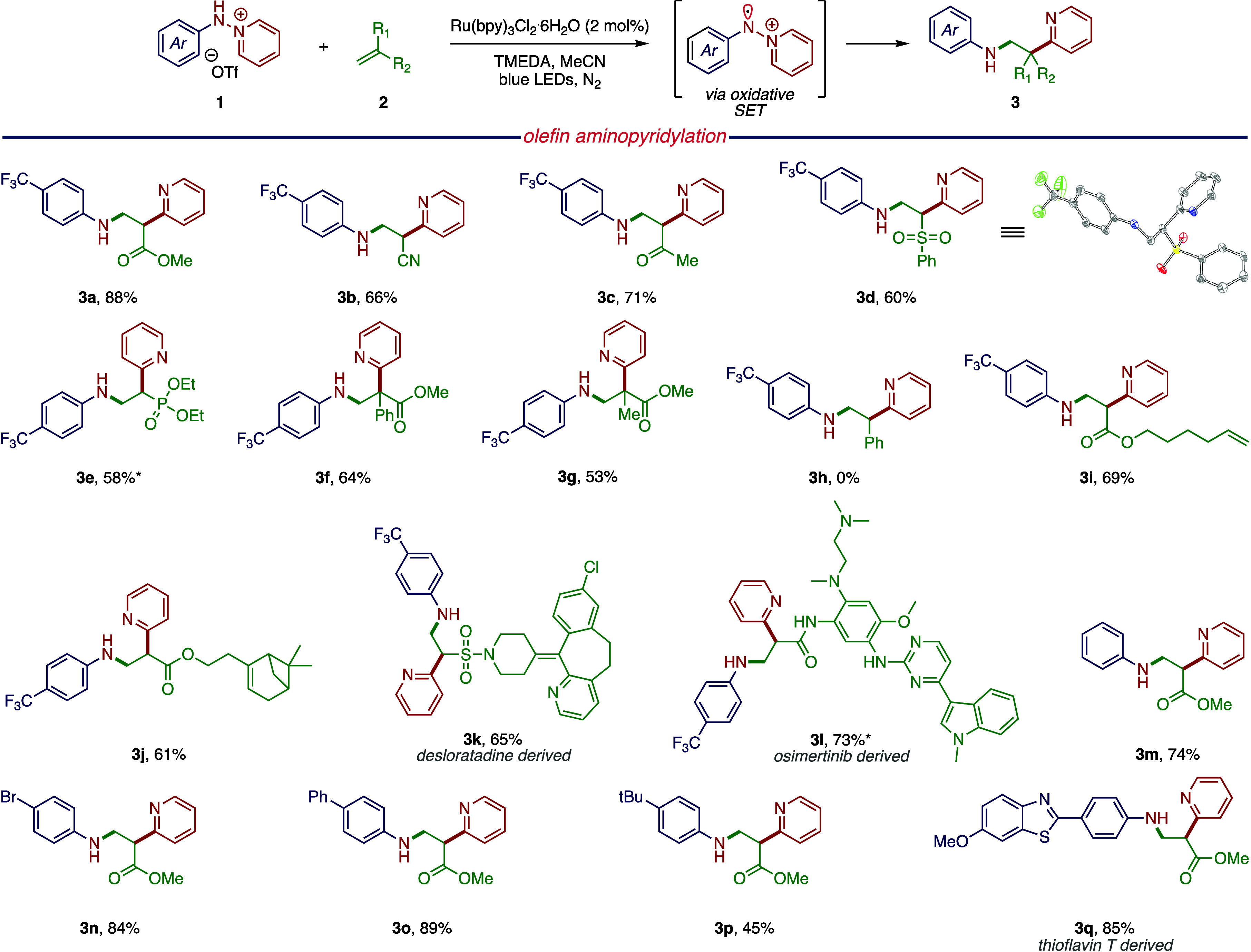
Olefin aminopyridylation enabled by the oxidative activation of pyridinium ylides. Condition A: **1** (1.0 equiv), **2** (1.5 equiv), Ru(bpy)_3_Cl_2_·6H_2_O (2 mol %), TMEDA (2.0 equiv), CH_3_CN, 23 °C, N_2_, 16 h; *Condition B: **1** (1.5 equiv), **2** (1.0 equiv), *fac*-Ir(ppy)_3_ (2 mol %), TMEDA (2.0 equiv), CH_3_CN, 23 °C, N_2_, 16 h; yields are isolated.

The optimized olefin aminopyridylation conditions accommodate the substitution of both aminopyridinium and olefin coupling partners (Figure 2). With respect to the olefinic substrate (**2**), an array of electron-poor substrates participated in efficient aminopyridylation: Methyl acrylate (**2a**), acrylonitrile (**2b**), methyl vinyl ketone (**2c**), vinyl sulfone (**2d**), and vinyl phosphonate (**2e**) all afforded the corresponding aminopyridylated products in high yields. 1,2-Disubstituted acrylates **2f** and **2g** underwent aminopyridylation to afford compounds **3f** and **3g** in 64 and 53% yields, respectively. Electron-rich olefins do not participate in productive aminopyridylation chemistry (see **3h** and Supporting Information, Figure S6), highlighting the selectivity of this process for electron-deficient olefins. This selectivity preference is further highlighted by the aminopyridylation of **2i** and **2j**, which affords **3i** (69%) and **3j** (61%) by selective functionalization of the electron-deficient olefin in preference to available aliphatic olefins. The developed aminopyridylation chemistry is further applicable in complex, pharmaceutically relevant molecular scaffolds: Aminopyridylation of loratadine-derived olefin **2k** afforded the corresponding product (**3k**) in 65% yield. Osimertinib (**2l**), a drug employed in treating nonsmall-cell lung carcinomas, was successfully converted to **3l** in 73% yield using *fac*-Ir(ppy)_3_ as the photocatalyst (whereas Ru(bpy)_3_Cl_2_·6H_2_O produced **3l** in 35% yield). These examples underscore the compatibility of aminopyridylation with basic heterocycles, amides, carbamates, and amines.

Olefin aminopyridylation is also compatible with the substitution of the *N*-aryl group of the pyridinium reaction partner (**1**). Electron-deficient, electron-neutral, and electron-donating groups all afford the respective aminopyridylated products in excellent yields (**3m**–**3p**). Additionally, thioflavin-T-derived aminopyridinium salt **1q**, upon reaction with methyl acrylate (**2a**), yielded product **3q** in 85% yield.

**Figure 3:**
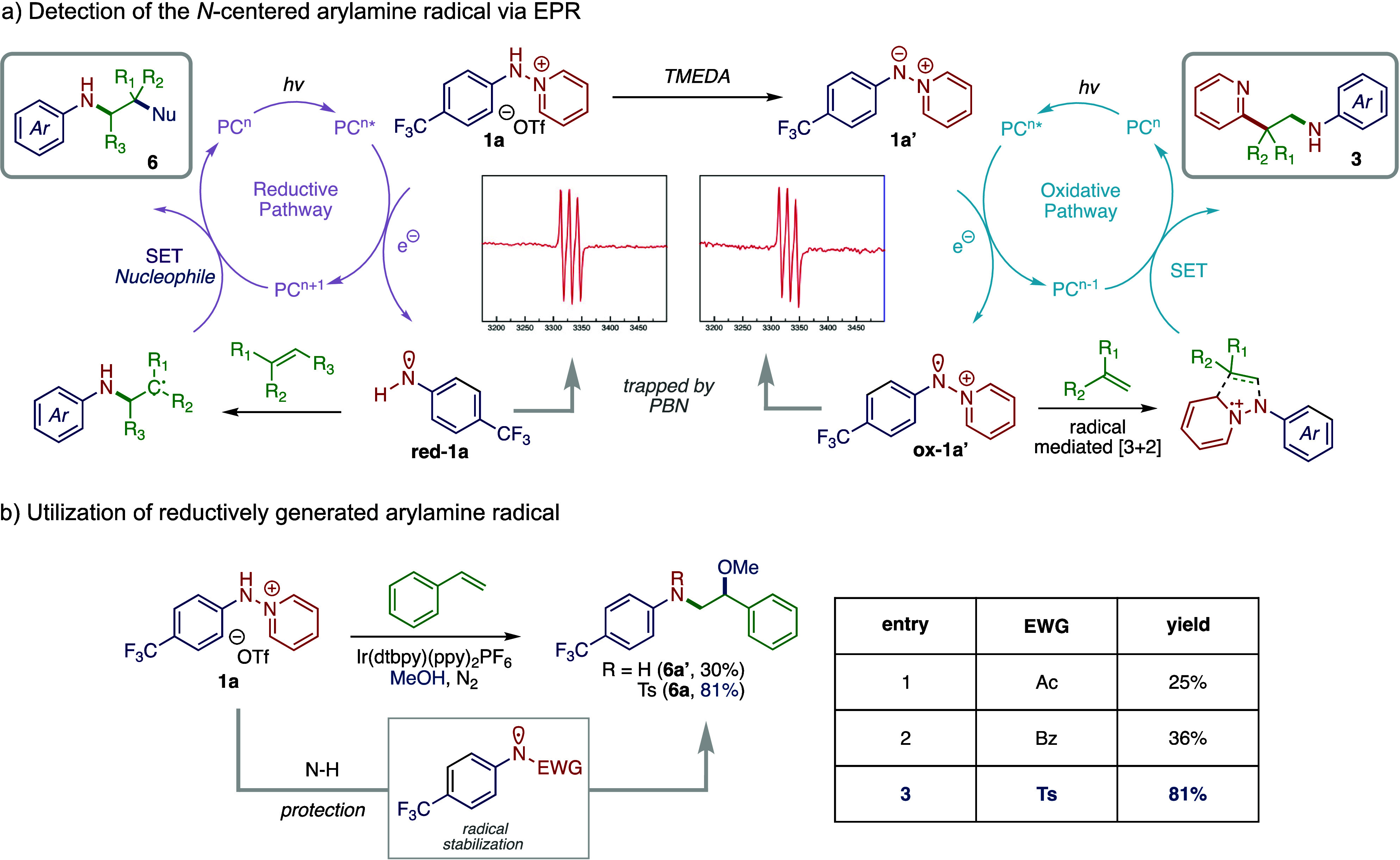
(a) EPR spectra of the PBN spin-trapped species of both the oxidatively and reductively generated *N*-centered radicals. (b) Utilization of the reductively generated arylamine radicals in olefin aminofunctionalization. Tosylation of the N–H valence of **1a** gives a stable radical and hence improved reaction efficiency.

To evaluate the hypothesis that olefin aminopyridylation proceeds via oxidative formation of an arylamine radical intermediate, we photolyzed an MeCN solution of **1a**, TMEDA, and Ru(bpy)_3_ Cl_2_·6H_2_O in the presence of *N*-*tert*-butyl-α-phenylnitrone (PBN), which is a commonly employed radical trapping agent.^34^ Analysis of the resulting reaction mixture by electron paramagnetic resonance (EPR) spectroscopy revealed spectral features characteristic of PBN-trapped arylamine radical **ox-1a′** (Figure 3a). The formation of the PBN adduct was further confirmed by high-resolution mass spectrometry (*m*/*z* = 416.1938 (expt), 416.1944 (calc); Supporting Information, Section C.1).

After demonstrating olefin aminopyridylation via oxidatively generated *N*-centered radicals, we sought to demonstrate bidirectional redox activation of **1** by developing complementary olefin functionalization chemistry via reductively generated arylamine radicals.^19^ To this end, we targeted reductive cleavage of the N–N bond by single-electron transfer (SET) to generate **red-1a**. We envisioned that **red-1a** would add to an appropriate olefinic substrate in an anti-Markovnikov fashion to generate a *C*-centered radical intermediate (Figure 3a). One-electron oxidation by PC^*n*+1^ and trapping of the incipient carbocation with an appropriate nucleophile would result in overall anti-Markovnikov olefin aminofunctionalization. Initial investigation of this scheme identified that the photolysis of a MeOH solution of **1a**, styrene, and [Ir(dtbbpy)(ppy)_2_]PF_6_ (1 mol %) afforded compound **6a′**, the product of 1,2-aminoalkoxylation, in 30% yield (Figure 3b). To enhance the stability of the reductively generated arylamine radical **red-1a**, and thus increase the lifetime for productive trapping by the olefinic substrate, we evaluated the substitution of the N–H valence in **1a** with stabilizing groups such as Ac, Bz, and Ts. Notably, tosyl substitution emerged as the most effective for our olefin oxyamination reaction, affording anti-Markovnikov addition product **6a** in an 81% yield (Figure 3b). A positive correlation was observed between the reaction yield for various *N*-functionalized starting materials and the acidity of the corresponding amides. Specifically, tosyl amide, with a p*K*_a_ of 16.1,^45^ exhibits the highest acidity and consequently outperforms the acetyl (p*K*_a_ = 25.5)^46^ and benzoyl (p*K*_a_ = 23.3)^47^ protecting groups. This correlation further implies that the tosyl-protecting group provides the highest stabilization of the *N*-centered radical, consistent with its enhanced acidity. This represents the first example of anti-Markovnikov oxyamination of olefins utilizing arylamines.

Consistent with the hypothesis of reductive radical generation, photolysis of a MeCN solution of **1a** and [Ir(dtbbpy)(ppy)_2_]PF_6_ in the presence of PBN provided an EPR spectrum diagnostic of the adduct of radical **red-1a** with PBN (Figure 3a and Supporting Information, Figure S2, Section C.1). The addition of TEMPO to the oxyamination reaction of **4a** resulted in the complete inhibition of olefin functionalization chemistry, which further supports the formation of an *N*-centered radical intermediate.

Additional support for the bidirectional electron transfer chemistry of *N*-aryl-*N*-aminopyridinium derivatives is evident from cyclic voltammetry (CV) experiments: **1a′** displays an irreversible oxidative feature at 0.45 V vs SCE, and **1a** displays an irreversible reductive feature at −0.86 V vs SCE, see the Supporting Information, Section C.2. Notably, cyclic voltammetry studies reveal that **1a** is inert to oxidation, while **1a′** is inert to reduction within the solvent window. The CV data also provide insight into the selection of photocatalysts for the two transformations. Specifically, the relevant electrochemical features of **1a** and **1a′** are well matched with those of the respective photocatalyst excited state potentials^48^ (see the Supporting Information, Section F.2).

The developed anti-Markovnikov aminofunctionalization chemistry can be applied to styrenyl olefins decorated with both electron-donating and electron-withdrawing substituents (i.e., **6a**–**6e**). Substitution of the aryl olefin coupling partner is well tolerated, as evidenced by the yields of substrates **6f** (82%), **6g** (49%), and **6h** (85%). Furthermore, thiophene-based olefin **5i** engaged in aminofunctionalization to afford **6i** in 50% yield. Disubstituted olefin partners also engage in productive anti-Markovnikov functionalization: α-Phenyl substituted styrene yielded product **6j** in 88% yield, while β-substituted olefins produced **6k** (52%) and **6l** (71%). The developed arylamine radical chemistry is also applicable to a range of *N*-aryl group substitutions of the pyridinium reaction partner (**4**), yielding products **6m** in 32% (free amine) and **6n** in 59% yield. Nopol-derived olefin substrate **5p** was converted to **6p** in 70% yield, which highlights the selectivity for the more activated C=C bond. Finally, probenecid-, oxaprozin-, and loratadine-derived olefins yielded their corresponding products in good yields (**6q**–**6s**), collectively confirming the utility of our method within pharmaceutically relevant motifs.

**Figure 4:**
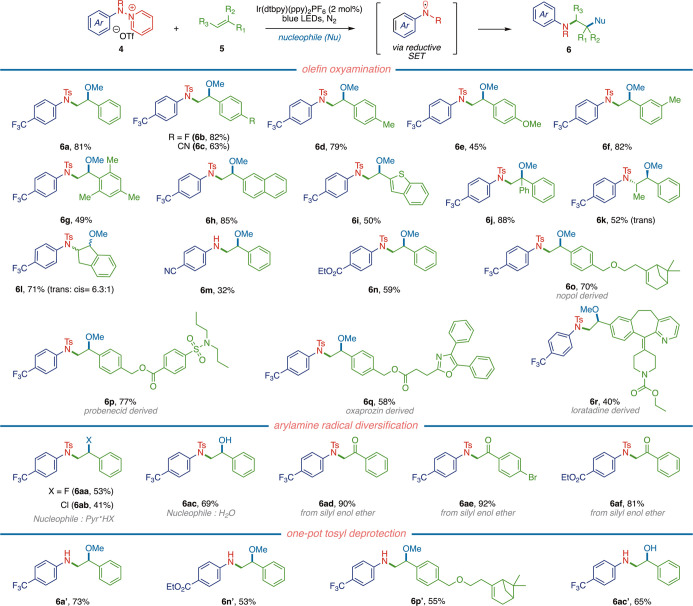
Scope of olefin aminofunctionalization with **4**. Oxyamination: **4** (1.0 equiv), **5** (1.5 equiv), Ir(dtbpy)(ppy)_2_PF_6_ (2 mol %), MeOH, 23 °C, N_2_, 16 h; aminohalogenation: **4** (1.0 equiv), **5** (1.5 equiv), Ir(dtbpy)(ppy)_2_PF_6_ (2 mol %), pyridine·HX (X = F or Cl), CH_2_Cl_2_, 40 °C, N_2_, 16 h; hydroxyamination: **4** (1.0 equiv), **5** (1.5 equiv), Ir(dtbpy)(ppy)_2_PF_6_ (2 mol %), acetone/water (1:1), 23 °C, N_2_, 16 h; α-amination of silyl enol ethers: **4** (1.0 equiv), **5** (1.5 equiv), Ir(dtbpy)(ppy)_2_PF_6_ (2 mol %), MeCN, 23 °C, N_2_, 16 h.

Addition of exogenous nucleophiles, such as pyridine·HF and pyridine·HCl, provided access to fluoroaminated and chloroaminated products **6aa** and **6ab**, respectively, with yields of 53 and 41%, and replacement of MeOH with 1:1 acetone/water resulted in hydroxyamination chemistry to generate **6ac** in 69% yield. In all cases, nitrogen addition proceeded with anti-Markovnikov selectivity. The developed chemistry could be extended to the α-amination of carbonyls (i.e., formal aza-Rubottom chemistry) by employing silyl enol ethers as the olefinic reaction partners. In this reaction modality, α-amination products **6ad**–**6af** were all obtained in excellent yields (Figure 4).

Of significance, the tosyl group that was introduced to stabilize the reductively generated arylamine radical could be straightforwardly removed. Free amines could be accessed in a one-pot procedure: Following aminofunctionalization, acridinium-based organic photocatalyst 9-mesityl-3,6-di-*tert*-butyl-10-phenylacridinium tetrafluoroborate and *N,N*-diisopropylethylamine (DIPEA, 3 equiv) were added to the reaction. Photolysis with 390 nm LEDs yielded the corresponding free amines. This one-pot procedure is efficient and general: Compounds **6a′**–**6n′** are all prepared using this protocol in excellent yields (the reported yields represent yields over two steps, Figure 4; see the Supporting Information, Section D.8).^49^

## Conclusions

In summary, we introduce bidirectional electron transfer chemistry as a platform to access *N*-radicals via complementary one-electron oxidation and reduction events. The oxidative generation of *N*-arylamine radicals—enabled by facile access to the corresponding ylide followed by one-electron oxidation by Ru(bpy)_3_Cl_2_·6H_2_O photocatalysts—affords anti-Markovnikov olefin aminopyridylation; the reductive generation of *N*-arylamine radicals—enabled by the one-electron reduction of the N–N linking of *N*-aminopyridinium compounds utilizing Ir(dtbpy)(ppy)_2_PF_6_ photocatalysts—affords anti-Markovnikov aminofunctionalization chemistry. Demonstration of oxidative and reductive mechanisms to common arylamine radicals from *N*-aminopyridinium salts provides new methods to rapidly construct and diversify arylamine scaffolds from readily available radical precursors and significantly expands anti-Markovnikov olefin aminofunctionalization chemistry.

## Supporting Information Available

Experimental methods, optimization data, spectral data, experimental spectra, and diffraction data (PDF)
CIF file of the compounds used in this work (CIF)

## Supplementary Material

cs4c04110_si_001.pdf

cs4c04110_si_002.cif
