# Supplementary material for: Bidirectional Electron Transfer Strategies for Anti-Markovnikov Olefin Aminofunctionalization via Arylamine Radicals
Source: ACS Catal. 2024 Aug 17;14(17):13156–62. doi: 10.1021/acscatal.4c04110 (PMC11385361; doi:10.1021/acscatal.4c04110)
Supplement: Supplementary file 1 — cs4c04110_si_001.pdf [file cs4c04110_si_001.pdf]

Supporting Information

Bidirectional Electron Transfer Strategies for Anti-Markovnikov Olefin Aminofunctionalization via Arylamine Radicals

Pritam Roychowdhury, Samya Samanta, Lauren Brown, Saim Waheed, and David C. Powers*

[a]Department of Chemistry, Texas A&M University, College Station, Texas 77843, USA

Email: powers@chem.tamu.edu

Table of Contents

A. General Considerations **S3**
A.1 Materials S3
A.2 Photolysis Details S3
A.3 Characterization Details S3
A.4 X-Ray Diffraction Details S4

B. Oxidative Olefin Aminopyridylation **S5**
B.1 Optimization Studies for the Olefin Aminopyridylation Reaction of **1a** S5
B.2 Synthesis and Charecterization for the Olefin Aminopyridylated Products **3** S6

C. Evidence for Bidirectional Electron Transfer **S13**
C.1 Spin-Trapping Experiments S13
C.2 Cyclic Voltammetry Data for **1a** and **1a'** S15

D. Reductive Olefin Aminofunctionalization **S17**
D.1 Effect of Protecting Groups on **1a** for Efficient Olefin Oxyamination S17
D.2 Tosyl Protection of *N*-aryl-*N*-aminopyridnium Derivatives **1** S18
D.3 Optimization Studies for the Olefin Oxyamination Reaction of **4a** S19
D.4 Olefin Oxyamination with *N*-aryl-*N*-aminopyridnium Derivatives **4** S20
D.5 Olefin Haloamination with *N*-aryl-*N*-aminopyridnium Derivatives S27
D.6 Olefin Hydroxyamination with *N*-aryl-*N*-aminopyridnium Derivatives S28
D.7 Formal α-Amination of Carbonyls with *N*-aryl-*N*-aminopyridnium Derivatives S29
D.8 One-pot Sulfonamide Deprotection Protocol S31

E. X-ray Diffraction Data **S33**

F. Additional Data **S35**
F.1 Summary of Unproductive Substrates S35
F.2 Comparison of the Excited State Redox Potentials of the Photocatalysts Used S36

G. NMR Spectra for New Compounds **S37**

H. References **S129**

A. General Considerations

A.1 Materials All chemicals and solvents were obtained as ACS reagent grade and used as received. Acrylonitrile (**2b**), but-3-en-2-one (**2c**), (vinylsulfonyl)benzene (**2d**), diethyl vinylphosphonate (**2e**), methyl 2-phenylacrylate (**2f**), methacrylate (**2g**), osimertinib (**2l**), [Ir(dtbbpy)(ppy)$_2$]PF$_6$, and *fac*-tris(2-phenylpyridine)iridium were obtained from Ambeed, Inc. Hexanes, ethyl acetate, acetone, dimethyl sulfoxide (DMSO), 4-(dimethylamino)pyridine (DMAP), styrene (**5a**), 1-fluoro-4-vinylbenzene (**5b**), 4-vinylbenzonitrile (**5c**), 1-methyl-4-vinylbenzene (**5d**), 1-methoxy-4-vinylbenzene (**5e**), 1-methyl-3-vinylbenzene (**5f**), 1,3,5-trimethyl-2-vinylbenzene (**5g**), 2-vinylnaphthalene (**5h**), 2-vinylbenzo[b]thiophene (**5i),** ethene-1,1-diyldibenzene (**5j**), trans-β-methylstyrene (**5k**), 1H-indene (**5l**), tetramethylethylenediame (TMEDA), *N*-tert-butyl-α-phenylnitrone (PBN), 9-mesityl-3,6-di-*tert*-butyl-10-phenylacridinium tetrafluoroborate, pyridine·HF, pyridine·HCl, and trimethyl((1-phenylvinyl)oxy)silane (**5s**) were obtained from Sigma Aldrich. Anhydrous methanol was obtained from Supelco. Triethylamine, *p*-toluenesulfonyl chloride, and *N,N*-diisopropylethylamine were obtained from TCI. Dry dichloromethane and acetonitrile (purchased from Fisher scientific, HPLC grade) was obtained from a drying column and stored over activated 4 Å molecular sieves.[1] NMR solvents were purchased from Cambridge Isotope Laboratories and were used as received. All reactions were carried out under ambient atmosphere unless otherwise noted.

1-((4-(Trifluoromethyl)phenyl)amino)-pyridin-1-ium triflate (**1a**),[2] 1-(phenylamino)pyridin-1-ium triflate (**1m**),[2] 1-((4-bromophenyl)amino)pyridin-1-ium triflate (**1n**),[2] 1-([1,1'-biphenyl]-4-ylamino)pyridin-1-ium triflate (**1o**),[2] 1-((4-(*tert*-butyl)phenyl)amino)pyridin-1-ium triflate (**1p**),[2] 1-((4-(6-methoxybenzo[d]thiazol-2-yl)phenyl)amino)pyridin-1-ium triflate (**1q**),[2] 1-((4-(ethoxycarbonyl)phenyl)amino)pyridin-1-ium trifluoromethanesulfonate,[2] hex-5-en-1-yl acrylate (**2i**),[3] 2-(6,6-dimethylbicyclo[3.1.1]hept-2-en-2-yl)ethyl acrylate (**2j**),[4] 8-chloro-11-(1-(vinylsulfonyl)piperidin-4-ylidene)-6,11-dihydro-5H-benzo[5,6]cyclohepta[1,2-b]pyridine (**2k**),[5] 1-((4-cyanophenyl)amino)pyridin-1-ium trifluoromethanesulfonate (**4m**),[2] 4-vinylbenzyl 4-(*N,N*-dipropylsulfamoyl)benzoate (**5p**),[6] 4-vinylbenzyl 3-(4,5-diphenyloxazol-2-yl)propanoate (**5q**),[6] ethyl 4-(8-vinyl-5,6-dihydro-11*H*-benzo[5,6]cyclohepta[1,2-*b*]pyridin-11-ylidene)piperidine-1-carboxylate (**5r**),[7] and ((1-(4-bromophenyl)vinyl)oxy)trimethylsilane (**5t**)[8] were prepared according to literature procedures.

A.2 Photolysis Details YUNBO blue LED strip lights (λ_{max} = 455 nm, 36 W) and a YUNBO LED power adapter were purchased from Amazon Inc. For photolysis experiments, the inside of a crystallizing dish was wrapped with the LED light strip and the reactions were performed using 20-mL scintillation vials held 3-4 cm from the LED lights without any filters. Reactions were cooled inside a fume hood using a Comfort Zone CZ6D 6" desk fan which was purchased from Amazon Inc.

A.3 Characterization Details ^{1}H and ^{13}C NMR spectral acquisitions were recorded on an Inova 500 FT NMR (Varian), a VNMRS 500 FT NMR (Varian), or an Acsend™ 400 NMR (Bruker) and were referenced against residual proteo solvent signals: $CDCl_3$ (7.26 ppm, ^{1}H; 77.16 ppm, ^{13}C) and acetonitrile-d_3 (1.94 ppm, ^{1}H).[8] ^{1}H NMR data are reported as follows: chemical shift (δ, ppm), (multiplicity: s (singlet), d (doublet), t (triplet), m (multiplet), br (broad), integration). ^{13}C NMR data are reported as follows: chemical shift (δ, ppm). Mass spectrometry data were recorded on either Orbitrap Fusion™ Tribrid™ Mass Spectrometer or Q Exactive™ Focus Hybrid

Quadrupole-Orbitrap™ Mass Spectrometer from ThermoFisher Scientific. EPR spectra were recorded at X-band (9.35 GHz) on a Bruker ELEXSYS Spectrometer at 23 °C in 2 mm EPR tubes.

A.4 X-Ray Diffraction Details Experimental details of crystallization are included in the synthetic procedures for the relevant compound. A Bruker APEX 2 Duo X-ray (three-circle) diffractometer was used for crystal screening, unit cell determination, and data collection for the X-ray crystal structures of **3d**. Crystal suitable for X-ray diffraction were mounted on a MiTeGen dual-thickness micro-mount and placed under a cold N_2 stream (Oxford). The X-ray radiation employed was generated from a Mo sealed X-ray tube (Kα = 0.70173 Å with a potential of 40 kV and a current of 40 mA). Bruker AXS APEX II software was used for data collection and reduction. Absorption corrections were applied using the program SADABS. A solution was obtained using XT/XS in APEX2 and refined in Olex2.[9, 10] Hydrogen atoms were placed in idealized positions and were set riding on the respective parent atoms. All non- hydrogen atoms were refined with anisotropic thermal parameters. The structure was refined (weighted least squares refinement on F2) to convergence.[10]

B. Oxidative Olefin Aminopyridylation

B.1 Optimization Studies for the Olefin Aminopyridylation Reaction of 1a

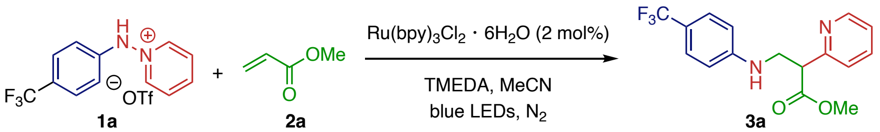

Entry	Deviation from Standard Conditions	Yield[a]
1	None	91%
2	In dark	0%
3	No $Ru(bpy)_3Cl_2{\cdot}6H_2O$	0%
4	No base	0%
5	Using Et_2NH as the base	64%
6	Using $K_2HPO_4{\cdot}3H_2O$ as the base	30%
7	Using DCM as the solvent	62%
8	Using DMSO as the solvent	83%
9	Using $(Ir[dF(CF_3)ppy]_2(dtbpy))PF_6$ as the photocatalyst	24%

[a] Determined by ^1H NMR using 1,3,5-trimethoxybenzene as internal standard

B.2 Synthesis and Charecterization for the Olefin Aminopyridylated Products 3

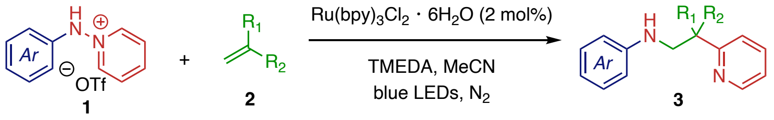

General Procedure A: A 20-mL scintillation vial was charged with *N*-aminopyridinium derivative (**1**, 0.10 mmol, 1.0 equiv), tris(2,2′-bipyridyl)dichlororuthenium(II) hexahydrate (1.5 mg, 0.0020 mmol, 0.020 equiv), and a magnetic stir bar in an N_2-filled dry box. Tetramethylethylenediamine (TMEDA, 30 µL, 0.20 mmol, 2.0 equiv), the appropriate olefinic substrate (**2**, 0.15 mmol, 1.5 equiv), and acetonitrile (1.0 mL) were added to the reaction vessel. The resulting solution was stirred for 16 h at 23 °C while being irradiated by blue LEDs (λ_{max} = 455 nm, 36W). After this time, the reaction mixture was concentrated under reduced pressure. The residue was then purified by neutral alumina column chromatography (hexanes/EtOAc) to afford the title compound **3**.

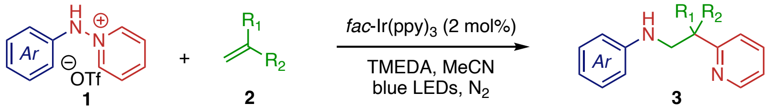

General Procedure B: A 20-mL scintillation vial was charged with *N*-aminopyridinium derivative (**1**, 0.15 mmol, 1.5 equiv), *fac*-tris(2-phenylpyridine)iridium(III) (1.3 mg, 0.0020 mmol, 0.020 equiv), and a magnetic stir bar in an N_2-filled dry box. Tetramethylethylenediamine (TMEDA, 30 µL, 0.20 mmol, 2.0 equiv), the appropriate olefinic substrate (**2**, 0.10 mmol, 1.0 equiv), and acetonitrile (1.0 mL) were then added to the reaction vessel. The resulting solution was stirred for 16 h at 23 °C while being irradiated by blue LEDs (λ_{max} = 455 nm, 36W). After this time, the reaction mixture was concentrated under reduced pressure. The residue was then purified by neutral alumina column chromatography (hexanes/EtOAc) to afford the title compound **3**.

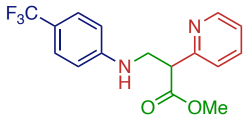

3a

*Methyl 2-(pyridin-2-yl)-3-((4-(trifluoromethyl)phenyl)amino)propanoate (**3a**)*. Prepared from 1-((4-(trifluoromethyl)phenyl)amino)-pyridin-1-ium triflate (**1a**) and methyl acrylate (**2a**) following General Procedure A and obtained as a pale yellow oil (28.5 mg, 88% yield). ^{1}H NMR (δ, 23 °C, 400 MHz, $CDCl_3$): 8.63 (dd, J = 4.8, 0.8 Hz, 1H), 7.70 (td, J = 7.7, 1.8 Hz, 1H), 7.41 (d, J = 8.5 Hz, 2H), 7.29–7.25 (m, 2H), 6.64 (d, J = 8.5 Hz, 2H), 4.60 (bs, 1H), 4.18 (d, J = 13.8 Hz, 1H), 3.95 (dt, J = 13.7, 6.8 Hz, 1H), 3.79 (dd, J = 13.5, 6.7 Hz, 1H), 3.74 (s, 3H). ^{13}C NMR (δ, 23 °C, 100 MHz, $CDCl_3$): 172.2, 156.4, 150.1, 149.9, 137.1, 126.8, 123.6 (q, $^1J_{C\text{–}F}$ = 269 Hz), 123.4, 122.8, 119.0 (q, $^2J_{C\text{–}F}$ = 33 Hz), 112.1, 52.5, 52.5, 44.9. HRMS (APCI) m/z: calculated for $[M+H]^+$ = 325.1158, observed $[M+H]^+$ = 325.1157.

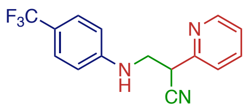

3b

*2-(pyridin-2-yl)-3-((4-(trifluoromethyl)phenyl)amino)propanenitrile (**3b**)*. Prepared from 1-((4-(trifluoromethyl)phenyl)amino)-pyridin-1-ium triflate (**1a**) and acrylonitrile (**2b**) following General Procedure A and obtained as a white solid (19.2 mg, 66% yield). ^{1}H NMR (δ, 23 °C, 400 MHz, $CDCl_3$): 8.66-8.65 (m, 1H), 7.77–7.73 (m, 1H), 7.45 (dd, J = 17.2, 8.2 Hz, 3H), 7.33–7.30 (m, 1H), 6.68 (d, J = 8.5 Hz, 2H), 4.66 (bs, 1H), 4.31 (dd, J = 8.5, 4.7 Hz, 1H), 3.97–3.90 (m, 1H), 3.85–3.80 (m, 1H). ^{13}C NMR (δ, 23 °C, 100 MHz, $CDCl_3$): 152.8, 150.3, 150.0, 137.8, 137.5, 132.4, 127.0, 126.9, 126.8, 124.4, 123.7 (q, $^{1}J_{C\text{–}F}$ = 269 Hz), 122.6, 121.2, 119.0 (q, $^{2}J_{C\text{–}F}$ = 33 Hz), 114.3, 112.5, 46.7, 39.4. ^{19}F NMR (δ, 23 °C, 376 MHz, $CDCl_3$): –61.3. HRMS (APCI) m/z: calculated for $[M+H]^+$ = 292.1056, observed $[M+H]^+$ = 292.1053.

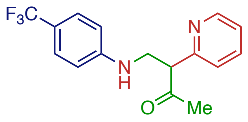

3c

*3-(pyridin-2-yl)-4-((4-(trifluoromethyl)phenyl)amino)butan-2-one (**3c**)*. Prepared from 1-((4-(trifluoromethyl)phenyl)amino)-pyridin-1-ium triflate (**1a**) and but-3-en-2-one (**2c**) following General Procedure A and obtained as a colorless oil (21.8 mg, 71% yield). ^{1}H NMR (δ, 23 °C, 400 MHz, $CDCl_3$): 8.62 (dt, J = 4.8, 0.8 Hz, 1H), 7.70 (td, J = 7.7, 1.8 Hz, 1H), 7.38 (d, J = 8.5 Hz, 2H), 7.27–7.21 (m, 2H), 6.59 (d, J = 8.5 Hz, 2H), 4.46 (s, 1H), 4.18 (dd, J = 7.5, 6.0 Hz, 1H), 3.93–3.86 (m, 1H), 3.64–3.57 (m, 1H), 2.10 (s, 3H). ^{13}C NMR (δ, 23 °C, 100 MHz, $CDCl_3$): 206.7, 156.7, 150.4, 137.4, 126.9, 126.8, 123.6 (q, $^{1}J_{C\text{–}F}$ = 269 Hz), 119.0 (q, $^{2}J_{C\text{–}F}$ = 32 Hz), 122.9, 114.3, 112.1, 60.4, 44.4, 29.6. HRMS (APCI) m/z: calculated for $[M+H]^+$ = 309.1209, observed $[M+H]^+$ = 309.1206.

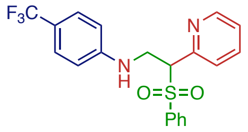

3d

*N-(2-(phenylsulfonyl)-2-(pyridin-2-yl)ethyl)-4-(trifluoromethyl)aniline (**3d**)*. Prepared from 1-((4-(trifluoromethyl)phenyl)amino)-pyridin-1-ium triflate (**1a**) and (vinylsulfonyl)benzene (**2d**) following General Procedure A and obtained as a white solid (24.4 mg, 60% yield). ^{1}H NMR (δ, 23 °C, 400 MHz, $CDCl_3$): 8.45 (dt, J = 4.8, 0.8 Hz, 1H), 7.67 (td, J = 7.7, 1.8 Hz, 1H), 7.62–7.58 (m, 1H), 7.54–7.52 (m, 2H), 7.44–7.36 (m, 5H), 7.25–7.23 (m, 1H), 6.55 (d, J = 8.5 Hz, 2H), 4.65 (dd, J = 7.4, 5.6 Hz, 1H), 4.56 (s, 1H), 4.21–4.12 (m, 2H). ^{13}C NMR (δ, 23 °C, 100 MHz, $CDCl_3$): 151.2, 149.7, 149.3, 136.9, 134.2, 129.2, 129.0, 126.9, 126.5, 123.9, 123.7 (q, $^{1}J_{C\text{–}F}$ = 269 Hz), 120.0 (q, $^{2}J_{C\text{–}F}$ = 32 Hz), 119.6, 112.4, 70.2, 41.7. HRMS (APCI) m/z: calculated for $[M+H]^+$ = 407.1036, observed $[M+H]^+$ = 407.1029.

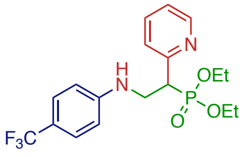

3e

*diethyl (1-(pyridin-2-yl)-2-((4-(trifluoromethyl)phenyl)amino)ethyl)phosphonate (**3e**).* Prepared from 1-((4-(trifluoromethyl)phenyl)amino)-pyridin-1-ium triflate (**1a**) and diethyl vinylphosphonate (**2e**) following General Procedure B and obtained as a white solid (23.3 mg, 58% yield). ^{1}H NMR (δ, 23 °C, 400 MHz, $CDCl_3$): 8.63–8.62 (m, 1H), 7.68 (td, J = 7.7, 1.7 Hz, 1H), 7.40 (d, J = 8.6 Hz, 3H), 7.24 (t, J = 6.2 Hz, 1H), 6.61 (d, J = 8.6 Hz, 2H), 4.66 (bs, 1H), 4.13–4.05 (m, 6H), 3.73 (ddd, J = 22.8, 8.1, 5.7 Hz, 1H), 1.30 (t, J = 7.1 Hz, 3H), 1.24 (t, J = 7.1 Hz, 3H). ^{13}C NMR (δ, 23 °C, 100 MHz, $CDCl_3$): 155.1, 150.0, 149.7, 136.8, 126.8, 124.9, 123.7, 122.6, 119.1 (q, $^{2}J_{C\text{–}F}$ = 32 Hz), 112.3, 62.8, 62.8, 62.7, 62.6, 46.8, 45.5, 42.9, 16.6, 16.5, 16.4. ^{31}P NMR (δ, 23 °C, 162 MHz, $CDCl_3$): 25.0. HRMS (APCI) m/z: calculated for $[M+H]^+$ = 403.1393, observed $[M+H]^+$ = 403.1389.

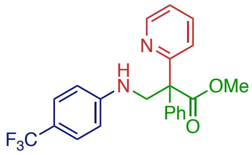

3f

*methyl 2-phenyl-2-(pyridin-2-yl)-3-((4-(trifluoromethyl)phenyl)amino)propanoate (**3f**).* Prepared from 1-((4-(trifluoromethyl)phenyl)amino)-pyridin-1-ium triflate (**1a**) and methyl 2-phenylacrylate (**2f**) following General Procedure A and obtained as a colorless oil (25.6 mg, 64% yield). ^{1}H NMR (δ, 23 °C, 400 MHz, $CDCl_3$): 8.61 (dd, J = 4.8, 0.7 Hz, 1H), 7.59 (td, J = 7.8, 1.8 Hz, 1H), 7.33–7.28 (m, 5H), 7.21 (dt, J = 6.1, 1.8 Hz, 3H), 7.13 (d, J = 8.0 Hz, 1H), 6.53 (d, J = 8.6 Hz, 2H), 5.33–5.30 (m, 1H), 4.31–4.21 (m, 2H), 3.72 (s, 3H). ^{13}C NMR (δ, 23 °C, 100 MHz, $CDCl_3$): 173.5, 161.0, 150.9, 148.4, 140.6, 136.4, 128.7, 128.2, 127.8, 126.4, 125.6, 123.7 (q, $^{1}J_{C\text{–}F}$ = 269 Hz), 122.5, 118.3 (q, $^{2}J_{C\text{–}F}$ = 32 Hz), 112.2, 63.6, 52.8, 50.4. ^{19}F NMR (δ, 23 °C, 376 MHz, $CDCl_3$): –60.9. HRMS (APCI) m/z: calculated for $[M+H]^+$ = 401.1471, observed $[M+H]^+$ = 401.1467.

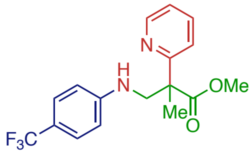

3g

*methyl 2-methyl-2-(pyridin-2-yl)-3-((4-(trifluoromethyl)phenyl)amino)propanoate (**3g**).* Prepared from 1-((4-(trifluoromethyl)phenyl)amino)-pyridin-1-ium triflate (**1a**) and methyl methacrylate (**2g**) following General Procedure A and obtained as a colorless oil (17.9 mg, 53% yield). ^{1}H NMR (δ, 23 °C, 400 MHz, $CDCl_3$): 8.58–8.57 (m, 1H), 7.66 (td, J = 7.8, 1.9 Hz, 1H), 7.32 (d, J = 8.5 Hz, 2H), 7.26 (t, J = 4.0 Hz, 1H), 7.19 (ddd, J = 7.5, 4.9, 1.0 Hz, 1H), 6.55 (d, J = 8.6 Hz, 2H), 4.79 (s, 1H), 3.77 (dd, J = 8.3, 5.2 Hz, 2H), 3.70 (s, 3H), 1.69 (s, 3H). ^{13}C NMR (δ, 23 °C, 100 MHz, $CDCl_3$): 175.5, 160.8, 151.0, 149.1, 136.9, 126.6, 123.7 (q, $^{1}J_{C\text{–}F}$ = 269 Hz), 122.4, 121.5,

118.4 (q, $^{2}J_{C\text{–}F}$ = 32 Hz), 112.0, 54.7, 52.6, 50.7, 21.8. ^{19}F NMR (*δ*, 23 °C, 376 MHz, $CDCl_3$): –61.0. HRMS (APCI) m/z: calculated for $[M+H]^+$ = 339.1315, observed $[M+H]^+$ = 339.1311.

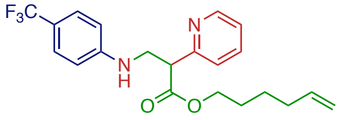

3i

hex-5-en-1-yl 2-(pyridin-2-yl)-3-((4-(trifluoromethyl)phenyl)amino)propanoate ***(3i)***. Prepared from 1-((4-(trifluoromethyl)phenyl)amino)-pyridin-1-ium triflate (**1a**) and hex-5-en-1-yl acrylate (**2i**) following General Procedure A and obtained as a colorless oil (27.0 mg, 69% yield). ^{1}H NMR (*δ*, 23 °C, 400 MHz, $CDCl_3$): 8.60 (d, *J* = 4.5 Hz, 1H), 7.69–7.65 (m, 1H), 7.38 (d, *J* = 8.5 Hz, 2H), 7.27–7.21 (m, 2H), 6.61 (d, *J* = 8.5 Hz, 2H), 5.70 (dq, *J* = 17.0, 8.5 Hz, 1H), 4.97–4.91 (m, 2H), 4.60 (t, *J* = 6.3 Hz, 1H), 4.13 (q, *J* = 6.2 Hz, 3H), 3.92 (dt, *J* = 13.7, 6.8 Hz, 1H), 3.76 (dt, *J* = 13.5, 6.7 Hz, 1H), 1.98 (q, *J* = 7.2 Hz, 2H), 1.57 (dt, *J* = 14.8, 7.1 Hz, 2H), 1.30 (td, *J* = 14.6, 7.2 Hz, 2H). ^{13}C NMR (*δ*, 23 °C, 100 MHz, $CDCl_3$): 171.8, 156.6, 150.1, 149.9, 138.3, 137.0, 126.8, 123.7 (q, $^{1}J_{C\text{–}F}$ = 269 Hz), 123.4, 122.7, 119.0 (q, $^{2}J_{C\text{–}F}$ = 32 Hz), 114.9, 112.1, 65.3, 52.7, 44.9, 33.2, 28.0, 25.1. ^{19}F NMR (*δ*, 23 °C, 376 MHz, $CDCl_3$): –61.0. HRMS (APCI) m/z: calculated for $[M+H]^+$ = 393.1784, observed $[M+H]^+$ = 393.1778.

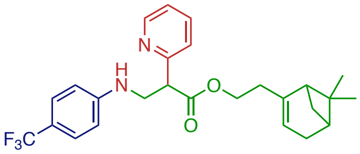

3j

2-(6,6-dimethylbicyclo[3.1.1]hept-2-en-2-yl)ethyl 2-(pyridin-2-yl)-3-((4-(trifluoromethyl)phenyl)amino)propanoate ***(3j)***. Prepared from 1-((4-(trifluoromethyl)phenyl)amino)-pyridin-1-ium triflate (**1a**) and 2-(6,6-dimethylbicyclo[3.1.1]hept-2-en-2-yl)ethyl acrylate (**2j**) following General Procedure A and obtained as a colorless oil (27.9 mg, 61% yield). ^{1}H NMR (*δ*, 23 °C, 400 MHz, $CDCl_3$): 8.60 (d, *J* = 4.6 Hz, 1H), 7.66 (td, *J* = 7.7, 1.8 Hz, 1H), 7.38 (d, *J* = 8.5 Hz, 2H), 7.26–7.20 (m, 2H), 6.61 (d, *J* = 8.5 Hz, 2H), 5.15 (d, *J* = 11.2 Hz, 1H), 4.58 (s, 1H), 4.16–4.09 (m, 3H), 3.91 (t, *J* = 6.8 Hz, 1H), 3.75 (td, *J* = 6.8, 1.6 Hz, 1H), 2.30–1.95 (m, 7H), 1.22 (s, 3H), 1.03 (dd, *J* = 8.5, 4.5 Hz, 1H), 0.72 (d, *J* = 3.7 Hz, 3H). ^{13}C NMR (*δ*, 23 °C, 100 MHz, $CDCl_3$): 171.7, 156.5, 150.1, 149.9, 143.9, 137.0, 126.8, 123.5, 122.7, 123.7 (q, $^{1}J_{C\text{–}F}$ = 269 Hz), 119.3, 119.0 (q, $^{2}J_{C\text{–}F}$ = 32 Hz), 118.9, 112.1, 63.6, 63.5, 52.6, 45.7, 45.6, 44.8, 40.8, 38.1, 38.1, 35.9, 35.9, 31.7, 31.7, 31.4, 26.3, 21.2. HRMS (APCI) m/z: calculated for $[M+H]^+$ = 459.2254, observed $[M+H]^+$ = 459.2247.

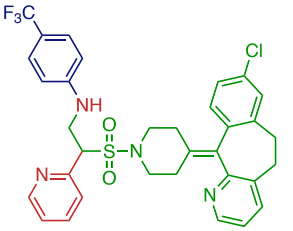

3k

*N-(2-((4-(8-chloro-5,6-dihydro-11H-benzo[5,6]cyclohepta[1,2-b]pyridin-11-ylidene)piperidin-1-yl)sulfonyl)-2-(pyridin-2-yl)ethyl)-4-(trifluoromethyl)aniline (**3k**).* Prepared from 1-((4-(trifluoromethyl)phenyl)amino)-pyridin-1-ium triflate (**1a**) and 8-chloro-11-(1-(vinylsulfonyl)piperidin-4-ylidene)-6,11-dihydro-5H-benzo[5,6]cyclohepta[1,2-b]pyridine (**2k**) following General Procedure A and obtained as a white solid (41.5 mg, 65% yield). ^{1}H NMR (δ, 23 °C, 400 MHz, $CDCl_3$): 8.65 (t, J = 4.4 Hz, 1H), 8.40–8.38 (m, 1H), 7.76–7.72 (m, 1H), 7.53 (dd, J = 7.9, 4.0 Hz, 1H), 7.42 (dd, J = 18.0, 8.1 Hz, 3H), 7.34 (t, J = 6.2 Hz, 1H), 7.17–7.11 (m, 3H), 7.04 (t, J = 7.6 Hz, 1H), 6.63 (d, J = 8.6 Hz, 2H), 4.64 (q, J = 7.4 Hz, 2H), 4.20–4.05 (m, 2H), 3.38–3.29 (m, 4H), 2.96–2.82 (m, 3H), 2.66 (q, J = 11.1 Hz, 1H), 2.47–2.23 (m, 4H). ^{13}C NMR (δ, 23 °C, 100 MHz, $CDCl_3$): 156.7, 152.6, 149.8, 146.8, 139.5, 137.5, 137.1, 136.1, 133.4, 133.2, 130.5, 129.2, 126.9, 126.4, 126.3, 125.6, 125.5, 124.2, 124.1, 123.6, 122.5, 119.6 (q, $^{2}J_{C-F}$ = 32 Hz), 112.4, 68.2, 60.5, 53.9, 47.4, 43.0, 31.7. ^{19}F NMR (δ, 23 °C, 376 MHz, $CDCl_3$): –61.1. HRMS (APCI) m/z: calculated for $[M+H]^+$ = 639.1803, observed $[M+H]^+$ = 639.1795.

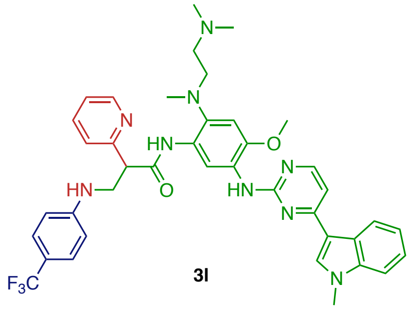

3l

*N-(2-((2-(dimethylamino)ethyl)(methyl)amino)-4-methoxy-5-((4-(1-methyl-1H-indol-3-yl)pyrimidin-2-yl)amino)phenyl)-2-(pyridin-2-yl)-3-((4-(trifluoromethyl) phenyl)amino)propanamide (**3l**).* Prepared from 1-((4-(trifluoromethyl)phenyl)amino)-pyridin-1-ium triflate (**1a**) and Osimertinib (**2l**) following General Procedure B and obtained as a yellow oil (53.8 mg, 73% yield). ^{1}H NMR (δ, 23 °C, 400 MHz, $CDCl_3$): 10.26 (s, 1H), 9.66 (s, 1H), 8.94 (s, 1H), 8.65 (dd, J = 4.9, 0.9 Hz, 1H), 8.39 (d, J = 5.3 Hz, 1H), 8.12–8.09 (m, 1H), 7.71 (s, 1H), 7.64 (td, J = 7.7, 1.8 Hz, 1H), 7.47 (d, J = 7.9 Hz, 1H), 7.37 (d, J = 8.3 Hz, 3H), 7.29–7.26 (m, 2H), 7.24–7.20 (m, 2H), 6.74 (s, 1H), 6.65 (d, J = 8.6 Hz, 2H), 4.92 (t, J = 6.3 Hz, 1H), 4.23 (dd, J = 7.8, 5.9 Hz, 1H), 4.04–3.99 (m, 1H), 3.91 (dd, J = 9.6, 4.7 Hz, 1H), 3.86 (s, 6H), 2.89–2.85 (m, 2H), 2.50 (s, 3H), 2.15–2.12 (m, 2H), 2.07 (s, 6H). ^{13}C NMR (δ, 23 °C, 100 MHz, $CDCl_3$): 207.1, 168.4, 162.2, 159.7, 158.0, 150.4, 149.7, 144.6, 138.3, 137.1, 134.6, 129.2, 127.7, 126.7, 126.7, 126.4, 126.0, 123.7, 123.1, 122.6, 122.1, 121.1, 120.5, 119.2 (q, $^{2}J_{C-F}$ = 32 Hz), 113.8, 112.3, 110.2, 110.0, 108.2, 104.7, 57.3, 56.2, 55.4, 54.9, 53.9, 46.0, 45.2, 44.5, 33.1, 31.9, 31.0, 29.4. ^{19}F NMR (δ, 23 °C, 376 MHz, $CDCl_3$): –61.0. HRMS (ESI) m/z: calculated for $[M+H]^+$ = 738.3486, observed $[M+H]^+$ = 738.3471.

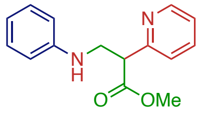

3m

*methyl 3-(phenylamino)-2-(pyridin-2-yl)propanoate (**3m**).* Prepared from 1-(phenylamino)pyridin-1-ium triflate (**1m**) and methyl acrylate (**2a**) following General Procedure A and obtained as a colorless oil (18.9 mg, 74% yield). ^{1}H NMR (δ, 23 °C, 400 MHz, $CDCl_3$): 8.61 (dd, J = 4.9, 0.8 Hz, 1H), 7.66 (td, J = 7.7, 1.8 Hz, 1H), 7.26 (t, J = 3.9 Hz, 1H), 7.23–7.15 (m, 3H), 6.71 (t, J = 7.3 Hz, 1H), 6.62 (dd, J = 8.5, 0.8 Hz, 2H), 4.19 (dd, J = 7.5, 6.6 Hz, 2H), 3.90 (dt, J = 14.1, 7.3 Hz, 1H), 3.73–3.68 (m, 4H). ^{13}C NMR (δ, 23 °C, 100 MHz, $CDCl_3$): 172.5, 156.9, 149.9, 147.5, 137.0, 129.5, 123.4, 122.6, 117.8, 113.1, 52.8, 52.4, 45.6. HRMS (APCI) m/z: calculated for $[M+H]^+$ = 257.1285, observed $[M+H]^+$ = 257.1283.

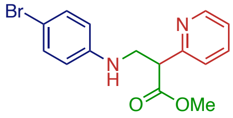

3n

*methyl 3-((4-bromophenyl)amino)-2-(pyridin-2-yl)propanoate (**3n**).* Prepared from 1-((4-bromophenyl)amino)pyridin-1-ium triflate (**1n**) and methyl acrylate (**2a**) following General Procedure A and obtained as a colorless oil (28.0 mg, 84% yield). ^{1}H NMR (δ, 23 °C, 400 MHz, $CDCl_3$): 8.60 (dd, J = 4.8, 0.8 Hz, 1H), 7.66 (td, J = 7.7, 1.8 Hz, 1H), 7.26–7.20 (m, 4H), 6.49 (d, J = 8.9 Hz, 2H), 4.22–4.21 (m, 1H), 4.14 (t, J = 6.9 Hz, 1H), 3.89–3.83 (m, 1H), 3.71–3.65 (m, 4H). ^{13}C NMR (δ, 23 °C, 100 MHz, $CDCl_3$): 172.3, 156.6, 149.9, 146.5, 137.1, 132.1, 123.4, 122.7, 114.7, 109.4, 52.6, 52.5, 45.5. HRMS (APCI) m/z: calculated for $[M+H]^+$ = 335.0390, observed $[M+H]^+$ = 335.0387.

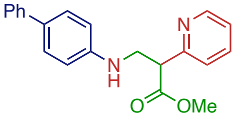

3o

*methyl 3-([1,1'-biphenyl]-4-ylamino)-2-(pyridin-2-yl)propanoate (**3o**).* Prepared from 1-([1,1'-biphenyl]-4-ylamino)pyridin-1-ium triflate (**1o**) and methyl acrylate (**2a**) following General Procedure A and obtained as a colorless oil (29.5 mg, 89% yield). ^{1}H NMR (δ, 23 °C, 400 MHz, $CDCl_3$): 8.62 (ddd, J = 4.9, 1.7, 0.8 Hz, 1H), 7.68 (td, J = 7.7, 1.8 Hz, 1H), 7.54–7.52 (m, 2H), 7.45–7.37 (m, 4H), 7.29–7.21 (m, 3H), 6.71–6.69 (m, 2H), 4.27–4.20 (m, 2H), 3.95 (dd, J = 13.6, 7.6 Hz, 1H), 3.78-3.72 (m, 4H). ^{13}C NMR (δ, 23 °C, 100 MHz, $CDCl_3$): 172.5, 156.8, 150.0, 146.9, 141.3, 137.1, 130.7, 128.8, 128.2, 126.4, 126.2, 123.4, 122.7, 115.5, 113.4, 52.8, 52.5, 45.6. HRMS (APCI) m/z: calculated for $[M+H]^+$ = 333.1598, observed $[M+H]^+$ = 333.1594.

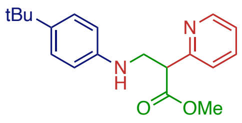

3p

*methyl 3-((4-(tert-butyl)phenyl)amino)-2-(pyridin-2-yl)propanoate (**3p**).* Prepared from 1-((4-(*tert*-butyl)phenyl)amino)pyridin-1-ium triflate (**1p**) and methyl acrylate (**2a**) following General Procedure A and obtained as a colorless oil (14.0 mg, 45% yield). ^{1}H NMR (δ, 23 °C, 400 MHz, $CDCl_3$): 8.60 (dd, J = 4.8, 0.8 Hz, 1H), 7.66 (td, J = 7.7, 1.8 Hz, 1H), 7.28 (s, 1H), 7.21 (td, J = 7.0, 1.6 Hz, 3H), 6.59 (d, J = 8.7 Hz, 2H), 4.20–3.86 (m, 3H), 3.70–3.65 (m, 4H), 1.27 (s, 9H). ^{13}C NMR (δ, 23 °C, 100 MHz, $CDCl_3$): 172.6, 157.0, 149.9, 145.1, 140.6, 137.0, 126.2, 123.3, 122.6, 112.9, 52.9, 52.4, 45.9, 34.0, 31.7. HRMS (APCI) m/z: calculated for $[M+H]^+$ = 313.1911, observed $[M+H]^+$ = 313.1908.

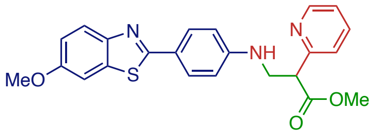

3q

*methyl 3-((4-(6-methoxybenzo[d]thiazol-2-yl)phenyl)amino)-2-(pyridin-2-yl)propanoate (**3q**).* Prepared from 1-((4-(6-methoxybenzo[d]thiazol-2-yl)phenyl)amino)pyridin-1-ium triflate (**1q)** and methyl acrylate (**2a**) following General Procedure A and obtained as an off-white solid (35.6 mg, 85% yield). ^{1}H NMR (δ, 23 °C, 400 MHz, $CDCl_3$): 8.62–8.61 (m, 1H), 7.85 (dd, J = 8.7, 6.0 Hz, 3H), 7.67 (td, J = 7.7, 1.7 Hz, 1H), 7.31 (d, J = 2.5 Hz, 1H), 7.27–7.21 (m, 2H), 7.03 (dd, J = 8.9, 2.5 Hz, 1H), 6.65 (d, J = 8.7 Hz, 2H), 4.61–4.58 (m, 1H), 4.19 (t, J = 6.9 Hz, 1H), 4.00–3.94 (m, 1H), 3.87 (s, 3H), 3.80 (dt, J = 13.5, 6.7 Hz, 1H), 3.72 (s, 3H). ^{13}C NMR (δ, 23 °C, 100 MHz, $CDCl_3$): 172.3, 166.3, 157.3, 156.5, 149.9, 149.6, 149.0, 137.1, 136.0, 128.9, 123.5, 123.3, 123.0, 122.8, 115.1, 112.7, 104.5, 55.9, 52.6, 52.5, 45.0. HRMS (APCI) m/z: calculated for $[M+H]^+$ = 420.1376, observed $[M+H]^+$ = 420.1374.

C. Evidence for Bidirectional Electron Transfer

C.1 Spin-Trapping Experiments

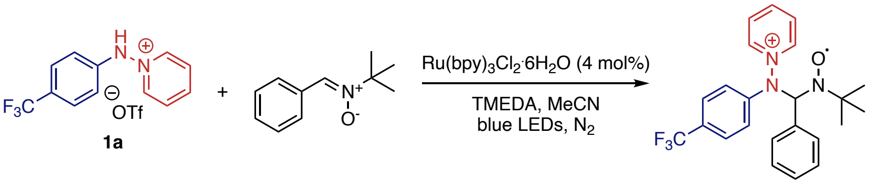

A 20-mL scintillation vial was charged with 1-((4-(trifluoromethyl)phenyl)amino)-pyridin-1-ium triflate (**1a**, 0.10 mmol, 1.0 equiv), tris(2,2′-bipyridyl)dichlororuthenium(II) hexahydrate (3.0 mg, 0.0040 mmol, 0.040 equiv), and a magnetic stir bar in an N_2-filled dry box. Tetramethylethylenediamine (TMEDA, 30 μL, 0.20 mmol, 2.0 equiv), *N*-tert-butyl-α-phenylnitrone (PBN, 21.2 mg, 0.12 mmol, 1.2 equiv) and acetonitrile (1.0 mL) were then added to the reaction vessel. The reaction mixture was irradiated for 2 h with blue LEDs (λ_{max} = 455 nm, 36W). An aliquot of the resulting solution (50 μL) was transferred to a 2 mm EPR tube and the EPR spectrum was collected (pictured below in Figure S1).

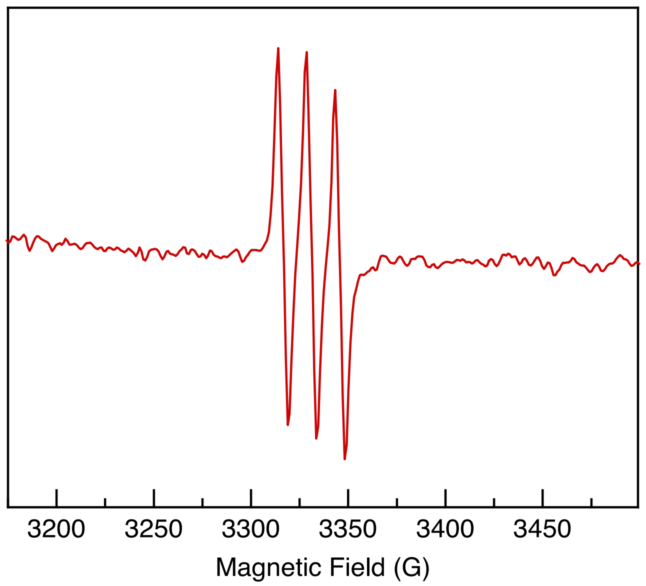

Figure S1. EPR spectrum for arylamine radical **ox-1a'** trapped by PBN. Formation of PBN-trapped arylamine radical was further confirmed by mass analysis of the EPR sample where HRMS-ESI: calculated for $[M+H]^+$ = 416.1944, observed $[M+H]^+$ = 416.1938.

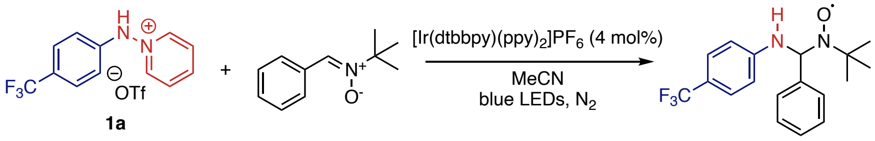

A 20-mL scintillation vial was charged with 1-((4-(trifluoromethyl)phenyl)amino)-pyridin-1-ium triflate (**1a**, 0.10 mmol, 1.0 equiv), [Ir(dtbbpy)(ppy)$_2$]PF$_6$ (4.0 mg, 0.0040 mmol, 0.040 equiv), and a magnetic stir bar in an N_2-filled dry box. *N*-tert-butyl-α-phenylnitrone (PBN, 21.2 mg, 0.12 mmol, 1.2 equiv) and acetonitrile (1.0 mL) were then added to the reaction vessel. The reaction mixture was irradiated for 2 h with blue LEDs (λ_{max} = 455 nm, 36W). An aliquot of the resulting solution (50 µL) was transferred to a 2 mm EPR tube and the EPR spectrum was collected (pictured below in Figure S2).

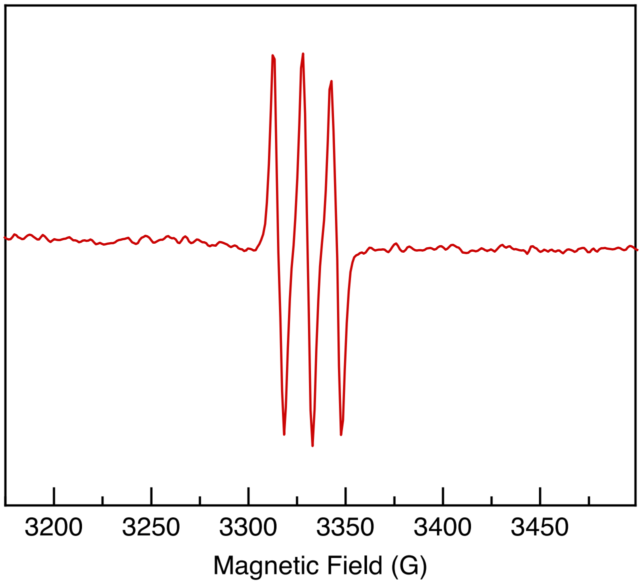

Figure S2. EPR spectrum for arylamine radical **red-1a** trapped by PBN.

C.2 Cyclic Voltammetry Data for 1a and 1a'

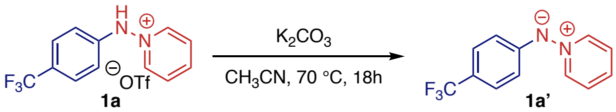

A 20 mL scintillation vial with a magnetic stir bar was charged with 1-((4-(trifluoromethyl)phenyl)amino)-pyridin-1-ium triflate (**1a,** 0.100 mmol, 1.00 equiv), K_2CO_3 (20.2 mg, 0.100 mmol, 1.00 equiv), and placed in a N_2 filled glovebox. After 30 min, anhydrous CH_3CN (2.50 mL) was added to the vial and the reaction mixture was heated at 70 °C. After for 18 h.The reaction mixture was cooled to 23 °C and an aliquot (~0.1 mL) of the reaction mixture was taken into an NMR tube under inert atmosphere, diluted with dry CD_3CN (~0.4 mL), and ^1H NMR was recorded. ^1H NMR (400 MHz, CD_3CN): δ 8.49 (dd, J = 7.0, 1.1 Hz, 2H), 7.67–7.62 (m, 1H), 7.57–7.54 (m, 2H), 7.27–7.24 (m, 2H), 6.60 (d, J = 8.7 Hz, 2H).

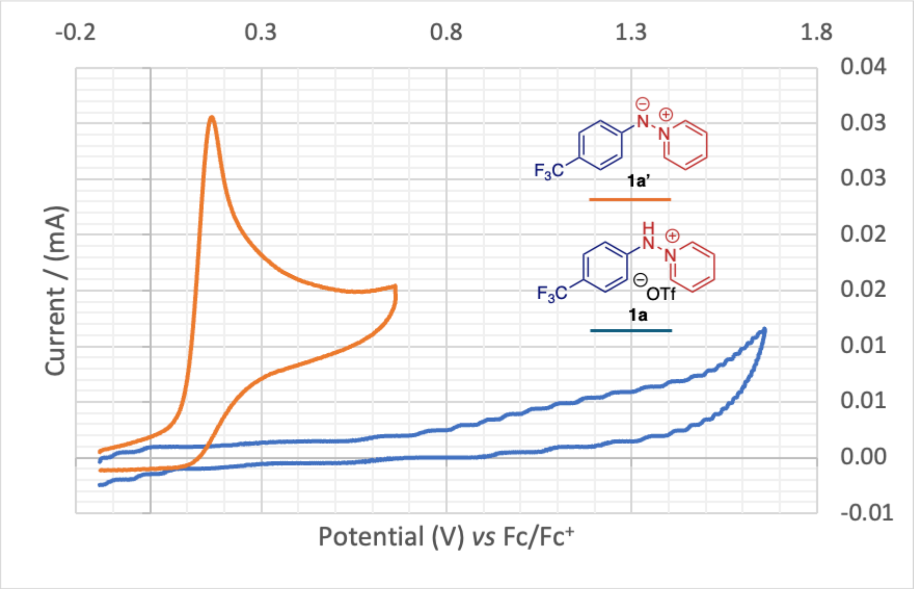

Figure S3. Cyclic voltammograms of pyridin-1-ium-1-yl(4-(trifluoromethyl)phenyl)amide (**1a'**) (—) and 1-((4-(trifluoromethyl)phenyl)amino)-pyridin-1-ium triflate (**1a**) (—). CV conditions: substrate 5 mM, 0.2 M [TBA]PF_6 solution of CH_3CN, glassy carbon working electrode, Pt counter electrode, and scan rate = 0.10 V/s. The onset oxidation potential at 0.065 V shows one electron oxidation of **1a'** where **1a** is silent.

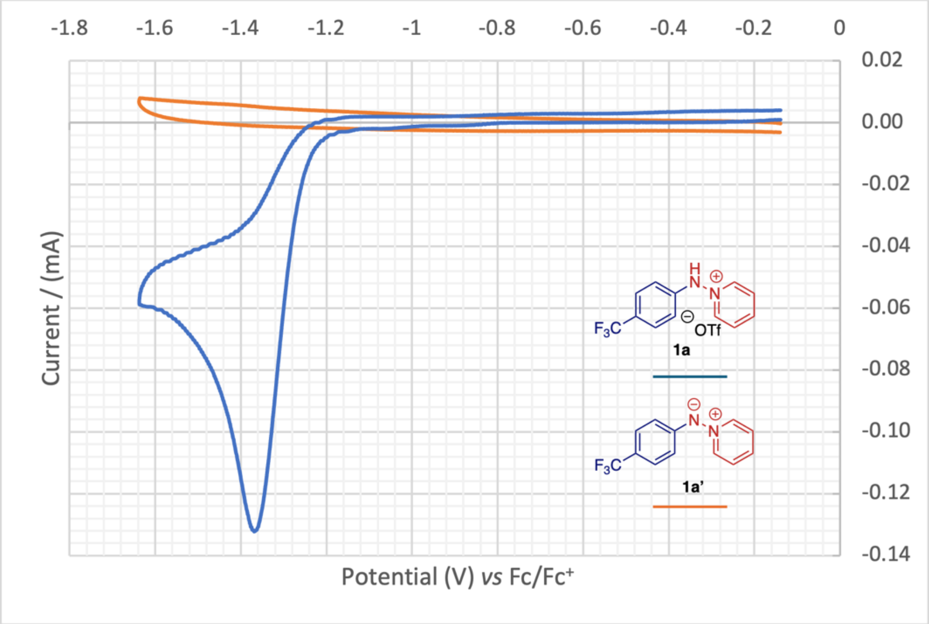

Figure S4. Cyclic voltammograms of 1-((4-(trifluoromethyl)phenyl)amino)-pyridin-1-ium triflate (**1a**) (—) and pyridin-1-ium-1-yl(4-(trifluoromethyl)phenyl)amide (**1a'**) (—). CV conditions: substrate 5 mM, 0.2 M [TBA]PF_6 solution of CH_3CN, glassy carbon working electrode, Pt counter electrode, and scan rate = 0.10 V/s. The onset reduction potential at –1.24 V shows one electron reduction of **1a** where the ylide **1a'** is silent.

D. Reductive Olefin Aminofunctionalization

D.1 Effect of Protecting Groups on 1a for Efficient Olefin Oxyamination

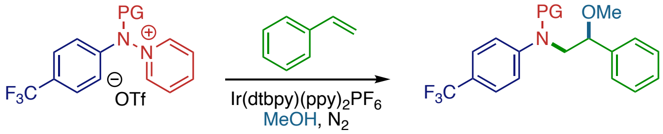

A 20-mL scintillation vial was charged with appropriate *N*-protected-*N*-arylaminopyridinium derivative (0.10 mmol, 1.0 equiv), $[Ir(dtbbpy)(ppy)_2]PF_6$ (2.0 mg, 0.0020 mmol, 0.020 equiv), and a magnetic stir bar in an N_2-filled dry box. Styrene (17 µL, 0.15 mmol, 1.5 equiv) and methanol (1.0 mL) were then added to the reaction vessel. The resulting solution was stirred for 16 h at 23 °C while being irradiated by blue LEDs (λ_{max} = 455 nm, 36W). After this time, the reaction mixture was concentrated under reduced pressure. The residue was then purified by silica gel column chromatography (hexanes/EtOAc) to afford the corresponding oxyaminated product.

Entry	Protecting Group	Yield
1	None (N–H)	30%
**2**	Ac	25%
**3**	**Ts**	**81%**
4	Bz	36%

D.2 Tosyl Protection of *N*-aryl-*N*-aminopyridnium Derivatives 1

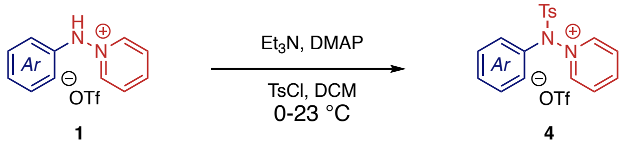

General Procedure: A 25-mL round-bottomed flask was charged with the appropriate *N*-aryl-*N*-aminopyridinium derivative (**1**, 1.00 mmol, 1.00 equiv), DMAP (12.2 mg, 0.100 mmol, 10.0 mol%), and dichloromethane (10.0 mL) and cooled to 0 °C. Triethylamine (167 µL, 1.20 mmol, 1.20 equiv) and TsCl (229 mg, 1.20 mmol, 1.20 equiv) were then added to the reaction vessel. The resulting mixture was slowly warmed to 23°C and stirred for 16 h. After this time, the reaction mixture was concentrated under reduced pressure and the residue was purified by silica gel column chromatography (eluent ethyl acetate followed by 85:15 EtOAC:MeOH) to give the title compound **4**. The obtained residue can be further purified by recrystallization (1:2 MeCN:Et_2O mixture).

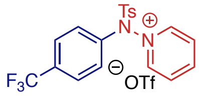

4a

*1-((4-methyl-N-(4-(trifluoromethyl)phenyl)phenyl)sulfonamido)pyridin-1-ium trifluoromethanesulfonate (**4a**).* Prepared from 1-((4-(trifluoromethyl)phenyl)amino)-pyridin-1-ium triflate (**1a**) following the General Procedure and obtained as a white solid (488 mg, 90% yield). ^{1}H NMR (δ, 23 °C, 400 MHz, CD_3CN): 9.01 (dd, J = 6.9, 1.2 Hz, 2H), 8.80–8.76 (m, 1H), 8.20 (dd, J = 7.8, 7.0 Hz, 2H), 7.84 (d, J = 8.6 Hz, 2H), 7.77 (d, J = 8.5 Hz, 2H), 7.73–7.71 (m, 2H), 7.50 (dd, J = 8.6, 0.5 Hz, 2H), 2.49 (s, 3H). ^{13}C NMR (δ, 23 °C, 100 MHz, CD_3CN): 151.6, 149.8, 149.2, 141.7, 133.2, 132.4, 132.2, 131.8, 130.4, 129.5, 128.8, 126.2, 120.8 (q, $^1J_{C–F}$ = 318 Hz), 118.9, 22.2. ^{19}F NMR (δ, 23 °C, 376 MHz, CD_3CN): –63.5, –79.3. HRMS (APCI) m/z: calculated for $[M]^+$ = 393.0879, observed $[M]^+$ = 393.0870.

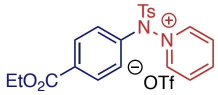

4n

*1-((N-(4-(ethoxycarbonyl)phenyl)-4-methylphenyl)sulfonamido)pyridin-1-ium trifluoromethanesulfonate (**4n**).* Prepared from 1-((4-(ethoxycarbonyl)phenyl)amino)pyridin-1-ium trifluoromethanesulfonate following the General Procedure and obtained as a pale yellow solid (470 mg, 86% yield). ^{1}H NMR (δ, 23 °C, 400 MHz, CD_3CN): 9.02–9.00 (m, 2H), 8.78 (tt, J = 7.9, 1.3 Hz, 1H), 8.21 (dd, J = 7.8, 7.0 Hz, 2H), 8.11–8.08 (m, 2H), 7.73–7.71 (m, 2H), 7.68–7.66 (m, 2H), 7.50 (dd, J = 8.6, 0.6 Hz, 2H), 4.35 (q, J = 7.1 Hz, 2H), 2.49 (s, 3H), 1.35 (t, J = 7.1 Hz, 3H). ^{13}C NMR (δ, 23 °C, 100 MHz, CD_3CN): 165.8, 151.2, 149.4, 148.9, 141.7, 133.5, 132.1, 132.0, 131.9, 131.4, 130.1, 128.4, 120.5 (q, $^1J_{C–F}$ = 319 Hz), 62.5, 21.9, 14.5. ^{19}F NMR (δ, 23 °C, 376 MHz, CD_3CN): –79.3. HRMS (APCI) m/z: calculated for $[M]^+$ = 397.1217, observed $[M]^+$ = 397.1209.

D.3 Optimization Studies for the Olefin Oxyamination Reaction of 4a

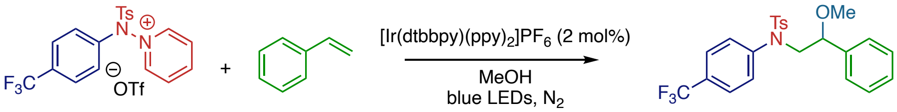

4a 5a 6a

Entry	Deviation from Standard Conditions	Yield[a]
1	None	84%
2	In dark	0%
3	No Photocatalyst	0%
4	In presence of 1 equiv of TEMPO	0%
5	Using *fac*-Ir(ppy)$_3$ as the photocatalyst	30%
6	Using Ru(bpy)$_3$Cl$_2$·6H$_2$O as the photocatalyst	26%

[a] Determined by ^{1}H NMR using 1,3,5-trimethoxybenzene as internal standard

D.4 Olefin Oxyamination with *N*-aryl-*N*-aminopyridnium Derivatives 4

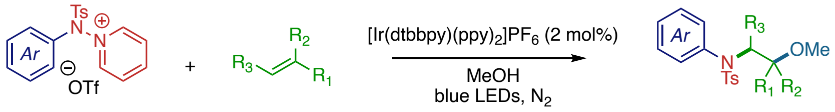

General Procedure: A 20-mL scintillation vial was charged with *N*-aryl-*N*-aminopyridinium derivative (**4**, 0.10 mmol, 1.0 equiv), $[Ir(dtbbpy)(ppy)_2]PF_6$ (2.0 mg, 0.0020 mmol, 0.020 equiv), and a magnetic stir bar in an N_2-filled dry box. The appropriate olefinic substrate (**5**, 0.15 mmol, 1.5 equiv) and methanol (1.0 mL) were then added to the reaction vessel. The resulting solution was stirred for 16 h at 23 °C while being irradiated by blue LEDs (λ_{max} = 455 nm, 36W). After this time, the reaction mixture was concentrated under reduced pressure. The residue was then purified by silica gel column chromatography (hexanes/EtOAc) to afford the title compound **6**.

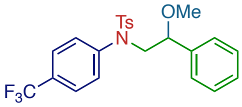

6a

*N-(2-methoxy-2-phenylethyl)-4-methyl-N-(4-(trifluoromethyl)phenyl)benzenesulfonamide (**6a**).* Prepared from 1-((4-methyl-N-(4-(trifluoromethyl)phenyl)phenyl)sulfonamido)pyridin-1-ium trifluoromethanesulfonate (**4a**) and styrene (**5a**) and obtained as a colorless oil (36.4 mg, 81% yield). ^{1}H NMR (δ, 23 °C, 400 MHz, $CDCl_3$): 7.55 (d, J = 8.4 Hz, 2H), 7.46 (d, J = 8.3 Hz, 2H), 7.33 (t, J = 5.0 Hz, 3H), 7.27–7.24 (m, 4H), 7.13 (d, J = 8.3 Hz, 2H), 4.39 (dd, J = 8.2, 4.9 Hz, 1H), 3.83 (dd, J = 14.2, 8.2 Hz, 1H), 3.69 (dd, J = 14.2, 4.9 Hz, 1H), 3.17 (s, 3H), 2.43 (s, 3H). ^{13}C NMR (δ, 23 °C, 100 MHz, $CDCl_3$): 144.0, 143.9, 138.8, 135.4, 129.8, 129.7, 129.5, 129.2, 128.7, 128.4, 127.7, 127.1, 126.1, 122.6 (q, $^1J_{C\text{–}F}$ = 270 Hz), 82.9, 57.4, 57.0, 21.7. ^{19}F NMR (δ, 23 °C, 376 MHz, $CDCl_3$): –62.5. HRMS (APCI) m/z: calculated for $[M+H]^+$ = 450.1345, observed $[M+H]^+$ = 450.1337.

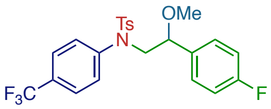

6b

*N-(2-(4-fluorophenyl)-2-methoxyethyl)-4-methyl-N-(4-(trifluoromethyl)phenyl) benzenesulfonamide (**6b**).* Prepared from 1-((4-methyl-N-(4-(trifluoromethyl)phenyl)phenyl)sulfonamido)pyridin-1-ium trifluoromethanesulfonate (**4a**) and 1-fluoro-4-vinylbenzene (**5b**) and obtained as a colorless oil (38.3 mg, 82% yield). ^{1}H NMR (δ, 23 °C, 400 MHz, $CDCl_3$): 7.53 (d, J = 8.3 Hz, 2H), 7.43 (d, J = 8.3 Hz, 2H), 7.24–7.19 (m, 4H), 7.09 (d, J = 8.2 Hz, 2H), 7.00 (t, J = 8.7 Hz, 2H), 4.36 (dd, J = 7.9, 5.3 Hz, 1H), 3.78 (dd, J = 14.2, 8.0 Hz, 1H), 3.64 (dd, J = 14.2, 5.3 Hz, 1H), 3.13 (s, 3H), 2.41 (s, 3H). ^{13}C NMR (δ, 23 °C, 100 MHz, $CDCl_3$): 164.0, 161.6, 144.0, 135.2, 134.6, 129.9, 129.7, 129.6, 129.1, 128.9, 128.8, 127.7, 126.2, 126.2, 126.1, 122.6 (q, $^1J_{C\text{–}F}$ = 270 Hz), 115.7, 115.5, 82.2, 57.3, 56.9, 21.7. ^{19}F NMR (δ, 23 °C, 376 MHz, $CDCl_3$): –62.5, –113.8. HRMS (ESI) m/z: calculated for $[M+H]^+$ = 468.1251, observed $[M+H]^+$ = 468.1248.

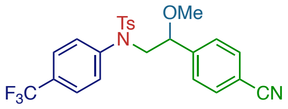

6c

N-(2-(4-cyanophenyl)-2-methoxyethyl)-4-methyl-N-(4-(trifluoromethyl)phenyl)benzenesulfonamide (***6c***). Prepared from 1-((4-methyl-N-(4-(trifluoromethyl)phenyl)phenyl)sulfonamido)pyridin-1-ium trifluoromethanesulfonate (**4a**) and 4-vinylbenzonitrile (**5c**) and obtained as a colorless oil (29.8 mg, 63% yield). ^{1}H NMR (δ, 23 °C, 400 MHz, $CDCl_3$): 7.63 (d, J = 8.3 Hz, 2H), 7.54 (d, J = 8.4 Hz, 2H), 7.40 (t, J = 7.8 Hz, 4H), 7.23 (d, J = 8.0 Hz, 2H), 7.09 (d, J = 8.3 Hz, 2H), 4.48 (dd, J = 7.7, 5.4 Hz, 1H), 3.75 (dd, J = 14.3, 7.7 Hz, 1H), 3.66 (dd, J = 14.3, 5.3 Hz, 1H), 3.18 (s, 3H), 2.41 (s, 3H). ^{13}C NMR (δ, 23 °C, 100 MHz, $CDCl_3$): 144.5, 144.2, 143.9, 134.9, 132.6, 131.9, 130.1, 129.8, 129.0, 128.5, 127.8, 127.7, 126.3, 122.6 (q, $^1J_{C\text{–}F}$ = 270 Hz), 118.6, 112.4, 82.5, 57.4, 57.1, 21.7. ^{19}F NMR (δ, 23 °C, 376 MHz, $CDCl_3$): –62.5. HRMS (ESI) m/z: calculated for $[M+H]^+$ = 475.1298, observed $[M+H]^+$ = 475.1293.

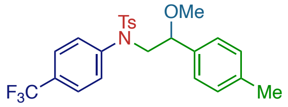

6d

N-(2-methoxy-2-(p-tolyl)ethyl)-4-methyl-N-(4-(trifluoromethyl)phenyl)benzenesulfonamide (***6d***). Prepared from 1-((4-methyl-N-(4-(trifluoromethyl)phenyl)phenyl)sulfonamido)pyridin-1-ium trifluoromethanesulfonate (**4a**) and 1-methyl-4-vinylbenzene (**5d**) and obtained as a colorless oil (36.6 mg, 79% yield). ^{1}H NMR (δ, 23 °C, 400 MHz, $CDCl_3$): 7.53 (d, J = 8.4 Hz, 2H), 7.44 (d, J = 8.3 Hz, 2H), 7.23–7.21 (m, 2H), 7.13–7.11 (m, 6H), 4.31 (dd, J = 8.3, 4.9 Hz, 1H), 3.79 (dd, J = 14.2, 8.3 Hz, 1H), 3.66 (dd, J = 14.1, 4.9 Hz, 1H), 3.13 (s, 3H), 2.41 (s, 3H), 2.33 (s, 3H). ^{13}C NMR (δ, 23 °C, 100 MHz, $CDCl_3$): 143.9, 138.2, 135.7, 135.4, 129.8, 129.7, 129.5, 129.4, 129.2, 127.7, 126.1, 126.0, 122.6 (q, $^1J_{C\text{–}F}$ = 270 Hz), 82.6, 57.3, 56.8, 21.7, 21.3. ^{19}F NMR (δ, 23 °C, 376 MHz, $CDCl_3$): –62.5. HRMS (ESI) m/z: calculated for $[M+H]^+$ = 464.1502, observed $[M+H]^+$ = 464.1499.

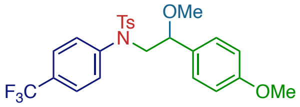

6e

N-(2-methoxy-2-(4-methoxyphenyl)ethyl)-4-methyl-N-(4-(trifluoromethyl)phenyl)benzenesulfonamide (***6e***). Prepared from 1-((4-methyl-N-(4-(trifluoromethyl)phenyl)phenyl)sulfonamido)pyridin-1-ium trifluoromethanesulfonate (**4a**) and 1-methoxy-4-vinylbenzene (**5e**) and obtained as a colorless oil (21.5 mg, 45% yield). ^{1}H NMR (δ, 23 °C, 400 MHz, $CDCl_3$): 7.52 (d, J = 8.4 Hz, 2H), 7.43 (d, J = 8.3 Hz, 2H), 7.21 (s, 2H), 7.12 (dd, J = 16.3, 8.4 Hz, 4H), 6.84 (d, J = 8.7 Hz, 2H), 4.29 (dd, J = 8.1, 5.2 Hz, 1H), 3.81–3.79 (m, 4H), 3.64 (dd, J = 14.1, 5.2 Hz, 1H), 3.11 (s, 3H), 2.41 (s, 3H). ^{13}C NMR (δ, 23 °C, 100 MHz, $CDCl_3$): 159.8, 143.9, 135.4, 130.7, 129.7, 129.2, 128.4, 127.7, 126.1, 122.6 (q, $^1J_{C\text{–}F}$ = 270 Hz), 114.1, 82.3, 57.2, 56.7, 55.4, 21.7. ^{19}F NMR (δ, 23 °C, 376 MHz, $CDCl_3$): –62.5. HRMS (APCI) m/z: calculated for $[M\text{-}CH_3OH+H]^+$ = 448.1189, observed $[M\text{-}CH_3OH+H]^+$ = 448.1181.

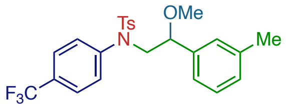

6f

N-(2-methoxy-2-(m-tolyl)ethyl)-4-methyl-N-(4-(trifluoromethyl)phenyl)benzenesulfonamide ***(6f)***. Prepared from 1-((4-methyl-N-(4-(trifluoromethyl)phenyl)phenyl)sulfonamido)pyridin-1-ium trifluoromethanesulfonate (**4a**) and 1-methyl-3-vinylbenzene (**5f**) and obtained as a colorless oil (38.0 mg, 82% yield). ^{1}H NMR (δ, 23 °C, 400 MHz, $CDCl_3$): 7.53 (d, J = 8.7 Hz, 2H), 7.46–7.43 (m, 2H), 7.24–7.18 (m, 3H), 7.10 (dd, J = 12.3, 8.2 Hz, 3H), 7.03 (d, J = 6.6 Hz, 2H), 4.32 (dd, J = 8.4, 4.7 Hz, 1H), 3.80 (dd, J = 14.2, 8.4 Hz, 1H), 3.65 (dd, J = 14.2, 4.7 Hz, 1H), 3.14 (s, 3H), 2.41 (s, 3H), 2.31 (s, 3H). ^{13}C NMR (δ, 23 °C, 100 MHz, $CDCl_3$): 144.0, 143.9, 138.7, 138.4, 135.5, 129.7, 129.3, 129.2, 128.6, 127.7, 127.7, 126.1, 126.0, 124.2, 122.6 (q, $^{1}J_{C\text{–}F}$ = 270 Hz), 82.9, 57.4, 56.9, 21.7, 21.5. ^{19}F NMR (δ, 23 °C, 376 MHz, $CDCl_3$): –62.5. HRMS (ESI) m/z: calculated for $[M+H]^+$ = 464.1502, observed $[M+H]^+$ = 464.1493.

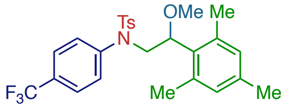

6g

N-(2-mesityl-2-methoxyethyl)-4-methyl-N-(4-(trifluoromethyl)phenyl)benzenesulfonamide ***(6g)***. Prepared from 1-((4-methyl-N-(4-(trifluoromethyl)phenyl)phenyl)sulfonamido)pyridin-1-ium trifluoromethanesulfonate (**4a**) and 1,3,5-trimethyl-2-vinylbenzene (**5g**) and obtained as a colorless oil (24.0 mg, 49% yield). ^{1}H NMR (δ, 23 °C, 400 MHz, $CDCl_3$): 7.51 (d, J = 8.4 Hz, 2H), 7.45 (d, J = 8.3 Hz, 2H), 7.23 (d, J = 8.0 Hz, 2H), 7.14 (d, J = 8.2 Hz, 2H), 6.74 (s, 2H), 4.85 (dd, J = 9.2, 4.7 Hz, 1H), 4.07 (dd, J = 14.4, 9.2 Hz, 1H), 3.65 (dd, J = 14.4, 4.8 Hz, 1H), 3.09 (s, 3H), 2.41 (s, 3H), 2.25 (s, 6H), 2.22 (s, 3H). ^{13}C NMR (δ, 23 °C, 100 MHz, $CDCl_3$): 143.9, 143.8, 137.6, 137.4, 135.6, 130.6, 129.7, 129.1, 127.7, 125.9, 122.6 (q, $^{1}J_{C\text{–}F}$ = 270 Hz), 79.4, 56.4, 54.2, 21.7, 20.8. ^{19}F NMR (δ, 23 °C, 376 MHz, $CDCl_3$): –62.5. HRMS (ESI) m/z: calculated for $[M+H]^+$ = 492.1815, observed $[M+H]^+$ = 492.1813.

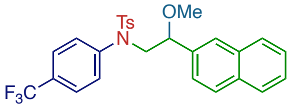

6h

N-(2-methoxy-2-(naphthalen-2-yl)ethyl)-4-methyl-N-(4-(trifluoromethyl)phenyl)benzenesulfonamide ***(6h)***. Prepared from 1-((4-methyl-N-(4-(trifluoromethyl)phenyl)phenyl)sulfonamido)pyridin-1-ium trifluoromethanesulfonate (**4a**) and 2-vinylnaphthalene (**5h**) and obtained as a colorless oil (42.4 mg, 85% yield). ^{1}H NMR (δ, 23 °C, 400 MHz, $CDCl_3$): 7.82–7.78 (m, 3H), 7.69 (s, 1H), 7.51–7.42 (m, 6H), 7.35 (dd, J = 8.3, 0.6 Hz, 1H), 7.20 (d, J = 8.0 Hz, 2H), 7.12 (d, J = 8.2 Hz, 2H), 4.53 (dd, J = 8.1, 5.0 Hz, 1H), 3.89 (dd, J = 14.2, 8.2 Hz, 1H), 3.77 (dd, J = 14.2, 5.0 Hz, 1H), 3.19 (s, 3H), 2.39 (s, 3H). ^{13}C NMR (δ, 23 °C, 100 MHz, $CDCl_3$): 144.0, 143.9, 136.2, 135.4, 133.5, 133.3, 129.8, 129.7, 129.5, 129.2, 128.7, 128.0, 127.9, 127.7, 126.7, 126.5, 126.3, 126.1, 124.3, 122.6 (q, $^{1}J_{C\text{–}F}$ = 270 Hz), 83.0, 57.2, 57.0, 21.7. ^{19}F NMR (δ, 23 °C, 376 MHz, $CDCl_3$): –62.5. HRMS (ESI) m/z: calculated for $[M+H]^+$ = 500.1502, observed $[M+H]^+$ = 500.1498.

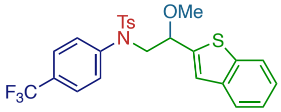

6i

N-(2-(benzo[b]thiophen-2-yl)-2-methoxyethyl)-4-methyl-N-(4-(trifluoromethyl)phenyl)benzenesulfonamide ***(6i)***. Prepared from 1-((4-methyl-N-(4-(trifluoromethyl)phenyl)phenyl)sulfonamido)pyridine-1-ium trifluoromethanesulfonate (**4a**) and 2-vinylbenzo[b]thiophene (**5i**) and obtained as a white solid (25.2 mg, 50% yield). ^{1}H NMR (δ, 23 °C, 400 MHz, $CDCl_3$): 7.77–7.71 (m, 2H), 7.46 (dd, J = 16.7, 8.3 Hz, 4H), 7.35–7.31 (m, 2H), 7.23–7.21 (m, 2H), 7.12 (d, J = 8.3 Hz, 2H), 4.75 (dd, J = 7.6, 5.5 Hz, 1H), 3.94 (dd, J = 14.2, 7.7 Hz, 1H), 3.81 (dd, J = 14.2, 5.5 Hz, 1H), 3.27 (s, 3H), 2.40 (s, 3H). ^{13}C NMR (δ, 23 °C, 100 MHz, $CDCl_3$): 144.1, 143.4, 139.9, 139.3, 135.1, 129.7, 129.2, 127.8, 126.1, 124.7, 124.5, 123.7, 123.5, 122.6 (q, $^{1}J_{C\text{–}F}$ = 270 Hz), 79.4, 57.3, 57.2, 21.7. ^{19}F NMR (δ, 23 °C, 376 MHz, $CDCl_3$): –62.5. HRMS (APCI) m/z: calculated for $[M\text{-}CH_3OH\text{+}H]^+$ = 474.0804, observed $[M\text{-}CH_3OH\text{+}H]^+$ = 474.0795.

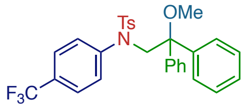

6j

N-(2-methoxy-2,2-diphenylethyl)-4-methyl-N-(4-(trifluoromethyl)phenyl)benzenesulfonamide ***(6j)***. Prepared from 1-((4-methyl-N-(4-(trifluoromethyl)phenyl)phenyl)sulfonamido)pyridin-1-ium trifluoromethanesulfonate (**4a**) and ethene-1,1-diyldibenzene (**5j**) and obtained as a white solid (46.2 mg, 88% yield). ^{1}H NMR (δ, 23 °C, 400 MHz, $CDCl_3$): 7.32–7.26 (m, 4H), 7.20–7.18 (m, 12H), 6.89 (d, J = 8.2 Hz, 2H), 4.67 (s, 2H), 2.80 (s, 3H), 2.41 (s, 3H). ^{13}C NMR (δ, 23 °C, 100 MHz, $CDCl_3$): 143.8, 143.5, 143.0, 135.3, 130.2, 129.5, 129.4, 129.1, 128.1, 128.0, 127.4, 127.3, 125.3, 122.6 (q, $^{1}J_{C\text{–}F}$ = 270 Hz), 82.6, 53.7, 50.9, 21.7. ^{19}F NMR (δ, 23 °C, 376 MHz, $CDCl_3$): –62.6. HRMS (APCI) m/z: calculated for $[M\text{-}CH_3OH\text{+}H]^+$ = 494.1396, observed $[M\text{-}CH_3OH\text{+}H]^+$ = 494.1386.

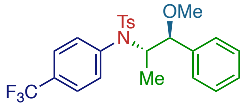

6k

N-((1S,2S)-1-methoxy-1-phenylpropan-2-yl)-4-methyl-N-(4-(trifluoromethyl)phenyl)benzenesulfonamide ***(6k)***. Prepared from 1-((4-methyl-*N*-(4-(trifluoromethyl)phenyl)phenyl)sulfonamido)pyridin-1-ium trifluoromethanesulfonate (**4a**) and trans-β-Methylstyrene (**5k**) and obtained as a white solid (24.0 mg, 52% yield, trans isomer). ^{1}H NMR (δ, 23 °C, 400 MHz, $CDCl_3$): 7.53 (dd, J = 19.9, 8.3 Hz, 4H), 7.39–7.30 (m, 5H), 7.23 (d, J = 8.2 Hz, 2H), 7.14 (d, J = 8.3 Hz, 2H), 4.62 (d, J = 3.9 Hz, 1H), 4.41 (tt, J = 7.0, 3.5 Hz, 1H), 3.10 (s, 3H), 2.41 (s, 3H), 1.02 (t, J = 6.9 Hz, 3H). ^{13}C NMR (δ, 23 °C, 100 MHz, $CDCl_3$): 143.6, 141.1, 139.2, 137.9, 132.6, 129.7, 128.6, 128.0, 127.5, 127.2, 125.6, 122.6 (q, $^{1}J_{C\text{–}F}$ = 270 Hz), 87.2, 61.6, 56.9, 21.7, 13.3. ^{19}F NMR (δ, 23 °C, 376 MHz, $CDCl_3$): –62.6. HRMS (ESI) m/z: calculated for $[M+H]^+$ = 464.1502, observed $[M+H]^+$ = 464.1496.

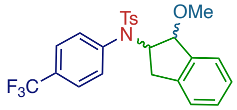

6l

N-(1-methoxy-2,3-dihydro-1H-inden-2-yl)-4-methyl-N-(4-(trifluoromethyl)phenyl)benzenesulfonamide ***(6l)***. Prepared from 1-((4-methyl-N-(4-(trifluoromethyl)phenyl)phenyl)sulfonamido)pyridin-1-ium trifluoromethanesulfonate (**4a**) and 1H-indene (**5l**) and obtained as a colorless oil (32.7 mg, 71% yield, *trans*:*cis* = 6.3:1).

Major isomer (*trans*-**6l**): ^{1}H NMR (δ, 23 °C, 400 MHz, $CDCl_3$): 7.69–7.67 (m, 2H), 7.56 (d, J = 8.3 Hz, 2H), 7.32 (s, 2H), 7.21 (s, 3H), 7.18–7.16 (m, 2H), 7.06 (t, J = 4.2 Hz, 1H), 5.11 (q, J = 7.9 Hz, 1H), 4.75 (d, J = 7.3 Hz, 1H), 3.47 (s, 3H), 3.23 (dd, J = 15.5, 8.0 Hz, 1H), 2.79 (dd, J = 15.5, 8.6 Hz, 1H), 2.46 (s, 3H). ^{13}C NMR (δ, 23 °C, 100 MHz, $CDCl_3$): 143.9, 139.8, 139.4, 138.6, 137.6, 132.7, 130.8, 129.7, 128.7, 127.9, 127.2, 126.4, 126.3, 124.9, 124.4, 122.4 (q, $^1J_{C\text{–}F}$ = 270 Hz), 84.9, 65.1, 55.9, 34.3, 21.7. ^{19}F NMR (δ, 23 °C, 376 MHz, $CDCl_3$): –62.8. HRMS (ESI) m/z: calculated for $[M+H]^+$ = 462.1345, observed $[M+H]^+$ = 462.1340.

Minor isomers (*cis*-**6l**): ^{1}H NMR (δ, 23 °C, 400 MHz, $CDCl_3$): 7.62 (d, J = 8.3 Hz, 2H), 7.51 (d, J = 8.3 Hz, 2H), 7.37 (d, J = 8.2 Hz, 2H), 7.30 (t, J = 7.8 Hz, 3H), 7.22–7.16 (m, 2H), 7.09–7.07 (m, 1H), 4.85 (ddd, J = 9.6, 7.6, 5.1 Hz, 1H), 4.70 (d, J = 5.1 Hz, 1H), 3.31 (s, 3H), 2.79 (dd, J = 15.1, 7.6 Hz, 1H), 2.69 (dd, J = 15.1, 9.6 Hz, 1H), 2.45 (s, 3H). ^{13}C NMR (δ, 23 °C, 100 MHz, $CDCl_3$): 143.7, 141.4, 140.4, 139.6, 137.4, 132.8, 129.6, 129.2, 127.8, 126.8, 125.7, 125.6, 125.6, 124.9, 83.8, 62.6, 56.3, 35.2. ^{19}F NMR (δ, 23 °C, 376 MHz, $CDCl_3$): –62.6. HRMS (ESI) m/z: calculated for $[M+H]^+$ = 462.1345, observed $[M+H]^+$ = 462.1340.

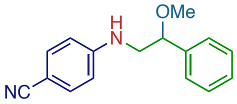

6m

4-((2-methoxy-2-phenylethyl)amino)benzonitrile ***(6m)***. Prepared from 1-((4-cyanophenyl)amino)pyridin-1-ium trifluoromethanesulfonate (**4m**) and styrene (**5a**) and obtained as colorless oil (8.1 mg, 32% yield). ^{1}H NMR (400 MHz, $CDCl_3$) δ 7.43–7.32 (m, 7H), 6.57 (d, J = 8.8 Hz, 2H), 4.66 (s, 1H), 4.37 (dd, J = 8.1, 4.6 Hz, 1H), 3.33 (dt, J = 8.4, 4.5 Hz, 2H), 3.28 (s, 3H). ^{13}C NMR (126 MHz, $CDCl_3$): δ 151.3, 139.2, 133.9, 128.9, 128.6, 126.8, 120.5, 112.7, 99.3, 82.0, 57.1, 49.8. HRMS (APCI) m/z: calculated for $[M+H]^+$ = 253.1335, observed $[M+H]^+$ = 253.1332.

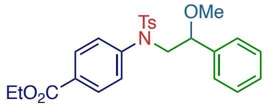

6n

ethyl 4-((N-(2-methoxy-2-phenylethyl)-4-methylphenyl)sulfonamido)benzoate ***(6n)***. Prepared from 1-((N-(4-(ethoxycarbonyl)phenyl)-4-methylphenyl)sulfonamido)pyridin-1-ium trifluoromethanesulfonate (**4n**) and styrene (**5a**) and obtained as a colorless oil (26.7 mg, 59% yield). ^{1}H NMR (δ, 23 °C, 400 MHz, $CDCl_3$): 7.95 (d, J = 8.4 Hz, 2H), 7.42 (d, J = 8.3 Hz, 2H), 7.30 (s, 3H), 7.24–7.19 (m, 4H), 7.06 (d, J = 8.5 Hz, 2H), 4.35 (dq, J = 18.1, 5.7 Hz, 3H), 3.66 (dd, J = 14.1, 5.0 Hz, 1H), 3.14 (s, 3H), 2.39 (s, 3H), 1.39 (t, J = 7.1 Hz, 3H). ^{13}C NMR (δ, 23 °C, 100 MHz, $CDCl_3$): 166.1, 144.7, 143.8, 138.9, 135.4, 130.4, 129.6, 128.7, 128.6, 128.4, 127.7,

127.1, 82.7, 61.3, 57.0, 21.7, 14.5. HRMS (APCI) m/z: calculated for $[M+H]^+$ = 454.1683, observed $[M+H]^+$ = 454.1672.

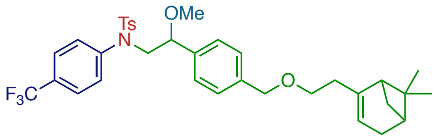

6o

*N-(2-(4-((2-(6,6-dimethylbicyclo[3.1.1]hept-2-en-2-yl)ethoxy)methyl)phenyl)-2-methoxyethyl)-4-methyl-N-(4-(trifluoromethyl)phenyl)benzenesulfonamide (**6o**).* Prepared from 1-((4-methyl-N-(4-(trifluoromethyl)phenyl)phenyl)sulfonamido)pyridin-1-ium trifluoromethanesulfonate (**4a**) and 6,6-dimethyl-2-(2-((4-vinylbenzyl)oxy)ethyl)bicyclo[3.1.1]hept-2-ene (**5o**) and obtained as a colorless oil (43.9 mg, 70% yield). ^{1}H NMR (δ, 23 °C, 400 MHz, $CDCl_3$): 7.53 (d, J = 8.5 Hz, 2H), 7.43 (d, J = 8.3 Hz, 2H), 7.28 (d, J = 8.0 Hz, 2H), 7.22 (t, J = 7.4 Hz, 4H), 7.12 (d, J = 8.3 Hz, 2H), 5.28 (s, 1H), 4.46 (s, 2H), 4.36 (dd, J = 8.3, 4.7 Hz, 1H), 3.77 (dd, J = 14.2, 8.3 Hz, 1H), 3.65 (dd, J = 14.2, 4.8 Hz, 1H), 3.50 (t, J = 7.1 Hz, 2H), 3.13 (s, 3H), 2.41 (s, 3H), 2.37–2.21 (m, 5H), 2.08–2.05 (m, 2H), 1.27 (s, 3H), 1.16 (d, J = 8.5 Hz, 1H), 0.83 (s, 3H). ^{13}C NMR (δ, 23 °C, 100 MHz, $CDCl_3$): 145.2, 144.0, 143.9, 139.0, 138.0, 135.4, 129.7, 129.2, 128.0, 127.7, 127.1, 126.1, 122.6 (q, $^1J_{C\text{–}F}$ = 270 Hz), 118.1, 82.7, 72.7, 69.2, 57.4, 56.9, 46.0, 40.9, 38.1, 37.3, 31.8, 31.5, 26.5, 21.7, 21.3. ^{19}F NMR (δ, 23 °C, 376 MHz, $CDCl_3$): –62.5. HRMS (ESI) m/z: calculated for $[M+NH_4]^+$ = 645.2968, observed $[M+NH_4]^+$ = 645.2957.

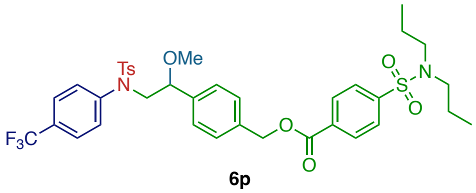

6p

*4-(1-methoxy-2-((4-methyl-N-(4-(trifluoromethyl)phenyl)phenyl)sulfonamido)ethyl)benzyl 4-(N,N-dipropylsulfamoyl)benzoate (**6p**).* Prepared from 1-((4-methyl-*N*-(4-(trifluoromethyl)phenyl)phenyl)sulfonamido)pyridin-1-ium trifluoromethanesulfonate (**4a**) and 4-vinylbenzyl 4-(*N*,*N*-dipropylsulfamoyl)benzoate (**5p**) and obtained as a colorless oil (57.4 mg, 77% yield). ^{1}H NMR (δ, 23 °C, 400 MHz, $CDCl_3$): 8.18 (d, J = 8.4 Hz, 2H), 7.88 (d, J = 8.4 Hz, 2H), 7.51 (d, J = 8.5 Hz, 2H), 7.41 (t, J = 8.5 Hz, 4H), 7.28 (d, J = 8.1 Hz, 2H), 7.22 (d, J = 8.2 Hz, 2H), 7.09 (d, J = 8.3 Hz, 2H), 5.36 (s, 2H), 4.40 (dd, J = 8.0, 5.2 Hz, 1H), 3.79 (dd, J = 14.2, 8.0 Hz, 1H), 3.67 (dd, J = 14.2, 5.2 Hz, 1H), 3.15 (s, 3H), 3.09 (t, J = 7.7 Hz, 4H), 2.40 (s, 3H), 1.55 (dt, J = 15.1, 7.5 Hz, 4H), 0.86 (t, J = 7.4 Hz, 6H). ^{13}C NMR (δ, 23 °C, 100 MHz, $CDCl_3$): 165.2, 144.6, 144.0, 139.3, 135.8, 135.2, 133.4, 130.4, 129.7, 129.2, 128.7, 127.7, 127.5, 127.2, 126.1, 122.6 (q, $^1J_{C\text{–}F}$ = 270 Hz), 82.6, 67.0, 57.3, 57.0, 50.1, 22.1, 21.7, 11.3. ^{19}F NMR (δ, 23 °C, 376 MHz, $CDCl_3$): –62.5. HRMS (ESI) m/z: calculated for $[M+H]^+$ = 747.2380, observed $[M+H]^+$ = 747.2371.

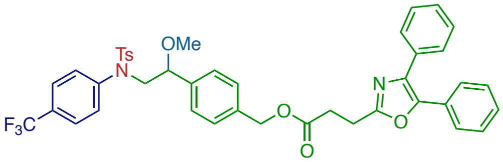

6q

4-(1-methoxy-2-((4-methyl-N-(4-(trifluoromethyl)phenyl)phenyl)sulfonamido)ethyl)benzyl 3-(4,5-diphenyloxazol-2-yl)propanoate ***(6q)***. Prepared from 1-((4-methyl-N-(4-(trifluoromethyl)phenyl)phenyl)sulfonamido)pyridin-1-ium trifluoromethanesulfonate (**4a**) and 4-vinylbenzyl 3-(4,5-diphenyloxazol-2-yl)propanoate (**5q**) and obtained as a white solid (43.7 mg, 58% yield). ^{1}H NMR (δ, 23 °C, 400 MHz, $CDCl_3$): 7.62 (dd, J = 8.0, 1.3 Hz, 2H), 7.57–7.52 (m, 4H), 7.43 (d, J = 8.2 Hz, 2H), 7.35–7.28 (m, 8H), 7.20 (dd, J = 17.3, 8.1 Hz, 4H), 7.11 (d, J = 8.3 Hz, 2H), 5.15 (s, 2H), 4.34 (dd, J = 8.3, 4.8 Hz, 1H), 3.77–3.61 (m, 2H), 3.22 (t, J = 7.4 Hz, 2H), 3.12 (s, 3H), 2.98 (t, J = 7.4 Hz, 2H), 2.40 (s, 3H). ^{13}C NMR (δ, 23 °C, 100 MHz, $CDCl_3$): 172.0, 161.8, 145.6, 144.0, 138.9, 136.1, 135.3, 132.6, 129.7, 129.2, 129.1, 128.8, 128.7, 128.6, 128.4, 128.2, 128.0, 127.7, 127.3, 126.6, 126.1, 122.6 (q, $^1J_{C\text{–}F}$ = 270 Hz), 82.6, 66.3, 57.4, 57.0, 31.2, 23.7, 21.7. ^{19}F NMR (δ, 23 °C, 376 MHz, $CDCl_3$): –62.5. HRMS (ESI) m/z: calculated for $[M+H]^+$ = 755.2397, observed $[M+H]^+$ = 755.2379.

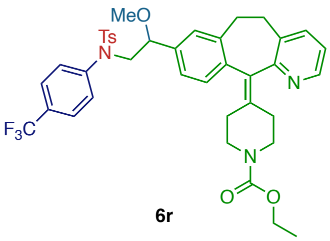

6r

Ethyl 4-(8-(1-methoxy-2-((4-methyl-N-(4-(trifluoromethyl)phenyl)phenyl)sulfonamido)ethyl)-5,6-dihydro-11H-benzo[5,6]cyclohepta[1,2-b]pyridin-11-ylidene)piperidine-1-carboxylate ***(6r).*** Prepared from 1-((4-methyl-*N*-(4-(trifluoromethyl)phenyl)phenyl)sulfonamido)pyridin-1-ium trifluoromethanesulfonate (**4a**) and ethyl 4-(8-vinyl-5,6-dihydro-11*H*-benzo[5,6]cyclohepta[1,2-*b*]pyridin-11-ylidene)piperidine-1-carboxylate (**5r**) and obtained as colorless oil (28.8 mg, 40% yield). ^{1}H NMR (400 MHz, $CDCl_3$) δ 8.42–8.41 (m, 1H), 7.47–7.39 (m, 5H), 7.26 (d, J = 2.8 Hz, 1H), 7.20 (t, J = 7.2 Hz, 2H), 7.14–7.10 (m, 2H), 7.05–6.98 (m, 3H), 4.36–4.32 (m, 1H), 4.13 (q, J = 7.0 Hz, 2H), 3.80–3.78 (m, 3H), 3.61–3.57 (m, 1H), 3.39–3.33 (m, 2H), 3.15 (dd, J = 4.3, 2.9 Hz, 4H), 2.85–2.75 (m, 2H), 2.51–2.26 (m, 7H), 1.81–1.76 (m, 1H), 1.25 (dd, J = 7.9, 6.3 Hz, 3H).^{13}C NMR (126 MHz, $CDCl_3$) δ 157.6, 155.6, 146.7, 144.1, 143.9, 139.3, 138.0, 137.6, 137.1, 135.4, 135.0, 133.8, 129.7, 129.2, 127.8, 127.7, 126.0, 125.0, 124.7, 122.8, 122.6 (q, $^1J_{C\text{–}F}$ = 270 Hz), 82.8, 82.6, 61.4, 57.5, 57.1, 45.0, 44.9, 32.0, 31.7, 30.9, 30.6, 29.4, 21.7, 14.8. ^{19}F NMR (376 MHz, $CDCl_3$) δ –62.4, –62.5. HRMS (APCI) m/z: calculated for $[M+H]^+$ = 720.2714, observed $[M+H]^+$ = 720.2700.

D.5 Olefin Haloamination with *N*-aryl-*N*-aminopyridnium Derivatives

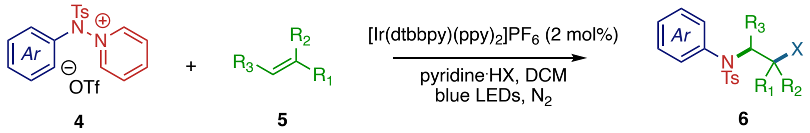

General Procedure: A 20-mL plastic vial was charged with *N*-aminopyridinium derivative (**4**, 0.10 mmol, 1.0 equiv), [Ir(dtbbpy)(ppy)$_2$]PF$_6$ (2.0 mg, 0.0020 mmol, 0.020 equiv), and a magnetic stir bar in an N_2-filled dry box. The appropriate olefinic substrate (**5**, 0.15 mmol, 1.5 equiv), pyridinium halide reagent (pyridine·HX), and dichloromethane (1.0 mL) were then added to the reaction vessel. The resulting solution was stirred for 16 h at 40 °C while being irradiated by blue LEDs (λ_{max} = 455 nm, 36W). After this time, the reaction mixture was concentrated under reduced pressure. The residue was then purified by silica gel column chromatography (hexanes/EtOAc) to afford the title compound **6**.

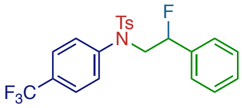

6aa

*N-(2-fluoro-2-phenylethyl)-4-methyl-N-(4-(trifluoromethyl)phenyl)benzenesulfonamide (**6aa**).* Prepared from 1-((4-methyl-*N*-(4-(trifluoromethyl)phenyl)phenyl)sulfonamido)pyridin-1-ium trifluoromethanesulfonate (**4a**), pyridine·HF (10 equiv HF), and styrene (**5a**) and obtained as a colorless oil (23.2 mg, 53% yield). ^{1}H NMR (δ, 23 °C, 400 MHz, $CDCl_3$): 7.57 (d, J = 8.5 Hz, 2H), 7.46 (d, J = 8.3 Hz, 2H), 7.36 (dd, J = 5.2, 1.8 Hz, 3H), 7.29 (t, J = 3.8 Hz, 2H), 7.24 (m, 2H), 7.18 (d, J = 8.4 Hz, 2H), 5.73–5.58 (m, 1H), 4.01–3.82 (m, 2H), 2.42 (s, 3H). ^{13}C NMR (δ, 23 °C, 100 MHz, $CDCl_3$): 144.3, 143.6, 136.9, 135.2, 129.8, 129.4, 129.2, 128.8, 127.8, 126.4, 125.9, 122.6 (q, $^1J_{C-F}$ = 270 Hz), 92.2 (d, $^1J_{C-F}$ = 176 Hz), 57.1, 56.8 (d, $^2J_{C-F}$ = 27 Hz), 21.7. ^{19}F NMR (δ, 23 °C, 376 MHz, $CDCl_3$): –62.6, –181.0. HRMS (ESI) m/z: calculated for [M+H]$^+$ = 438.1145, observed [M+H]$^+$ = 438.1139.

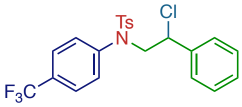

6ab

*N-(2-chloro-2-phenylethyl)-4-methyl-N-(4-(trifluoromethyl)phenyl)benzenesulfonamide (**6ab**).* Prepared from 1-((4-methyl-N-(4-(trifluoromethyl)phenyl)phenyl)sulfonamido)pyridin-1-ium trifluoromethanesulfonate (**4a**), pyridine·HCl (3 equiv HCl), and styrene (**5a**) and obtained as a colorless oil (18.6 mg, 41% yield). ^{1}H NMR (δ, 23 °C, 400 MHz, $CDCl_3$): 7.48 (d, J = 8.5 Hz, 2H), 7.42 (d, J = 8.3 Hz, 2H), 7.29 (s, 5H), 7.24 (s, 2H), 6.96 (d, J = 8.5 Hz, 2H), 5.00 (t, J = 7.5 Hz, 1H), 4.12 (dd, J = 14.2, 7.4 Hz, 1H), 3.95 (dd, J = 14.2, 7.6 Hz, 1H), 2.42 (s, 3H). ^{13}C NMR (δ, 23 °C, 100 MHz, $CDCl_3$): 144.3, 143.2, 138.1, 134.8, 129.8, 129.3, 129.1, 128.9, 127.8, 126.2, 125.2, 122.6 (q, $^1J_{C-F}$ = 270 Hz), 60.6, 58.3, 21.7. ^{19}F NMR (δ, 23 °C, 376 MHz, $CDCl_3$): –62.6. HRMS (APCI) m/z: calculated for [M+H]$^+$ = 454.0850, observed [M+H]$^+$ = 454.0846.

D.6 Olefin Hydroxyamination with *N*-aryl-*N*-aminopyridnium Derivatives

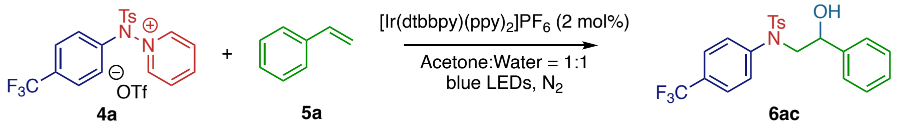

N-(2-hydroxy-2-phenylethyl)-4-methyl-N-(4-(trifluoromethyl)phenyl)benzenesulfonamide (***6ac***). A 20-mL scintillation vial was charged with *N*-aminopyridinium derivative (**4a**, 0.10 mmol, 1.0 equiv), $[Ir(dtbbpy)(ppy)_2]PF_6$ (2.0 mg, 0.0020 mmol, 0.020 equiv), and a magnetic stir bar. Styrene (**5a**, 0.15 mmol, 1.5 equiv) and acetone/water (1:1, 1.0 mL) were added to the reaction vessel and the reaction mixture was purged with N_2 for 15 min. The resulting solution was stirred for 16 h at 23 °C while being irradiated by blue LEDs (λ_{max} = 455 nm, 36W). After this time, the reaction mixture was concentrated under reduced pressure. The residue was then purified by silica gel column chromatography (hexanes/EtOAc) to afford the title compound **6ac** (30.0 mg, 69% yield). ^{1}H NMR (δ, 23 °C, 400 MHz, $CDCl_3$): 7.57 (d, J = 8.3 Hz, 2H), 7.46–7.44 (m, 2H), 7.31–7.29 (m, 5H), 7.24 (s, 2H), 7.17 (d, J = 8.2 Hz, 2H), 4.81 (dt, J = 8.5, 3.0 Hz, 1H), 3.84 (dd, J = 14.3, 8.9 Hz, 1H), 3.63 (dd, J = 14.3, 3.7 Hz, 1H), 2.73 (d, J = 2.8 Hz, 1H), 2.42 (s, 3H). ^{13}C NMR (δ, 23 °C, 100 MHz, $CDCl_3$): 144.3, 143.5, 140.7, 134.7, 129.9, 129.1, 128.7, 128.3, 127.8, 126.4, 126.2, 122.5 (q, $^1J_{C\text{–}F}$ = 270 Hz), 72.5, 59.0, 21.7. ^{19}F NMR (δ, 23 °C, 376 MHz, $CDCl_3$): –62.6. HRMS (ESI) m/z: calculated for $[M+H]^+$ = 436.1189, observed $[M+H]^+$ = 436.1184.

D.7 Formal α-Amination of Carbonyls with *N*-aryl-*N*-aminopyridnium Derivatives

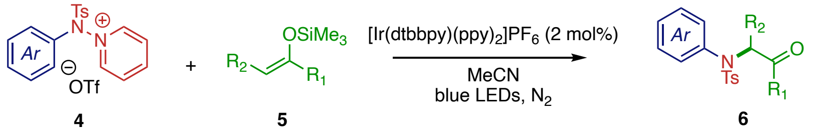

General Procedure: A 20-mL scintillation vial was charged with *N*-aminopyridinium derivative (**4**, 0.10 mmol, 1.0 equiv), $[Ir(dtbbpy)(ppy)_2]PF_6$ (2.0 mg, 0.0020 mmol, 0.020 equiv), and a magnetic stir bar in an N_2-filled dry box. The appropriate silyl enol ether (**5**, 0.15 mmol, 1.5 equiv), and acetonitrile (1.0 mL) were then added to the reaction vessel. The resulting solution was stirred for 16 h at 23 °C while being irradiated by blue LEDs (λ_{max} = 455 nm, 36W). After this time, the reaction mixture was concentrated under reduced pressure. The residue was then purified by silica gel column chromatography (hexanes/EtOAc) to afford the title compound **6**.

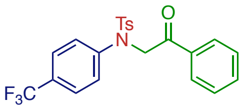

6ad

4-methyl-N-(2-oxo-2-phenylethyl)-N-(4-(trifluoromethyl)phenyl)benzenesulfonamide ***(6ad)***. Prepared from 1-((4-methyl-*N*-(4-(trifluoromethyl)phenyl)phenyl)sulfonamido)pyridin-1-ium trifluoromethanesulfonate (**4a**) and trimethyl((1-phenylvinyl)oxy)silane (**5s**) and obtained as a white solid (39.0 mg, 90% yield). ^{1}H NMR (δ, 23 °C, 400 MHz, $CDCl_3$): 7.93 (dd, J = 8.3, 1.2 Hz, 2H), 7.60–7.57 (m, 3H), 7.50 (dd, J = 14.5, 8.2 Hz, 4H), 7.30 (dd, J = 16.6, 8.2 Hz, 4H), 5.11 (s, 2H), 2.43 (s, 3H). ^{13}C NMR (δ, 23 °C, 100 MHz, $CDCl_3$): 193.3, 144.4, 143.1, 135.3, 134.7, 134.1, 129.8, 129.0, 128.4, 128.3, 128.0, 127.3, 126.4, 126.4, 126.3, 126.3, 125.2, 122.5, 119.9, 57.2, 21.7. ^{19}F NMR (δ, 23 °C, 376 MHz, $CDCl_3$): –62.6. HRMS (ESI) m/z: calculated for $[M+H]^+$ = 434.1032, observed $[M+H]^+$ = 434.1025.

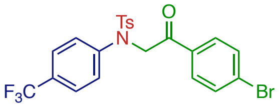

6ae

N-(2-(4-bromophenyl)-2-oxoethyl)-4-methyl-N-(4-(trifluoromethyl)phenyl)benzenesulfonamide ***(6ae)***. Prepared from 1-((4-methyl-*N*-(4-(trifluoromethyl)phenyl)phenyl)sulfonamido)pyridin-1-ium trifluoromethanesulfonate (**4a**) and ((1-(4-bromophenyl)vinyl)oxy)trimethylsilane (**5t**) and obtained as a white solid (47.0 mg, 92% yield). ^{1}H NMR (δ, 23 °C, 400 MHz, $CDCl_3$): 7.80 (d, J = 8.5 Hz, 2H), 7.61 (d, J = 8.6 Hz, 2H), 7.53 (dd, J = 15.1, 8.3 Hz, 4H), 7.28 (d, J = 7.8 Hz, 4H), 5.03 (s, 2H), 2.44 (s, 3H). ^{13}C NMR (δ, 23 °C, 100 MHz, $CDCl_3$): 192.6, 144.5, 142.9, 135.0, 133.5, 132.4, 130.2, 129.9, 129.8, 129.4, 128.4, 128.0, 126.4, 57.2, 21.8. ^{19}F NMR (δ, 23 °C, 376 MHz, $CDCl_3$): –62.6. HRMS (ESI) m/z: calculated for $[M+H]^+$ = 512.0137, observed $[M+H]^+$ = 512.0131.

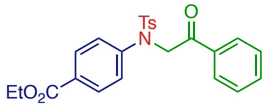

6af

*ethyl 4-((4-methyl-N-(2-oxo-2-phenylethyl)phenyl)sulfonamido)benzoate (**6af**)*. Prepared from 1-((N-(4-(ethoxycarbonyl)phenyl)-4-methylphenyl)sulfonamido)pyridin-1-ium trifluoromethanesulfonate (**4n**) and trimethyl((1-phenylvinyl)oxy)silane (**5s**) and obtained as a colorless oil (35.4 mg, 81% yield). ^{1}H NMR (δ, 23 °C, 400 MHz, $CDCl_3$): 7.94–7.91 (m, 4H), 7.61–7.56 (m, 3H), 7.47 (t, J = 7.8 Hz, 2H), 7.27 (s, 1H), 7.25–7.23 (m, 3H), 5.10 (s, 2H), 4.34 (q, J = 7.1 Hz, 2H), 2.42 (s, 3H), 1.35 (d, J = 7.1 Hz, 3H). ^{13}C NMR (δ, 23 °C, 100 MHz, $CDCl_3$): 193.5, 165.9, 144.3, 143.9, 135.2, 134.8, 134.0, 130.5, 129.7, 129.6, 128.9, 128.4, 128.1, 127.5, 61.3, 57.2, 21.8, 14.4. HRMS (APCI) m/z: calculated for $[M+H]^+$ = 438.1370, observed $[M+H]^+$ = 438.1364.

D.8 One-pot Sulfonamide Deprotection Protocol

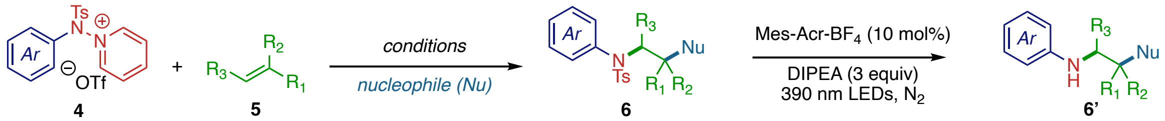

General Procedure: After completing the desired aminofunctionalization reaction, the reaction mixture (in a 20-mL scintillation vial, 0.10 mmol scale) was brought inside an N_2-filled dry box. At this stage, 9-mesityl-3,6-di-*tert*-butyl-10-phenylacridinium tetrafluoroborate (5.7 mg, 0.01 mmol, 0.10 equiv) and N,N-diisopropylethylamine (DIPEA, 52 μL, 0.30 mmol, 3.0 equiv) were added to the reaction mixture. The resulting solution was stirred for 16 h at 23 °C while being irradiated by blue LEDs (λ_{max} = 390 nm). After this time, the reaction mixture was concentrated under reduced pressure. The residue was then purified by silica gel column chromatography (hexanes/EtOAc) to afford the title compound **6'**.

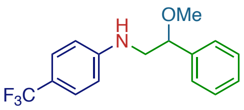

6a'

*N-(2-methoxy-2-phenylethyl)-4-(trifluoromethyl)aniline (**6a'**).* After carrying out the oxyaination reaction according to the General Procedure described in **Section D.3***,* the reaction mixture was subjected to one-pot sulfonamide deprotection protocol and the free amine was obtained as a colorless oil (21.5 mg, 73% yield). ^{1}H NMR (δ, 23 °C, 400 MHz, $CDCl_3$): 7.41–7.33 (m, 7H), 6.63 (d, J = 8.3 Hz, 2H), 4.52 (bs, 1H), 4.40–4.37 (m, 1H), 3.35–3.28 (m, 5H). ^{13}C NMR (δ, 23 °C, 100 MHz, $CDCl_3$): 150.6, 139.5, 128.9, 128.5, 126.9, 124.0, 119.1, 112.4, 82.1, 57.0, 50.3. ^{19}F NMR (δ, 23 °C, 376 MHz, $CDCl_3$): –61.0. HRMS (APCI) m/z: calculated for $[M+H]^+$ = 296.1257, observed $[M+H]^+$ = 296.1254.

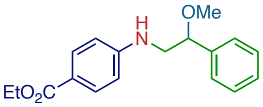

6n'

*ethyl 4-((2-methoxy-2-phenylethyl)amino)benzoate (**6n'**).* After carrying out the oxyaination reaction according to the General Procedure described in **Section D.3***,* the reaction mixture was subjected to one pot sulfonamide deprotection protocol and the free amine was obtained as a colorless oil (15.8 mg, 53% yield). ^{1}H NMR (δ, 23 °C, 400 MHz, $CDCl_3$): 7.87 (d, J = 8.8 Hz, 2H), 7.42–7.32 (m, 5H), 6.57 (d, J = 8.8 Hz, 2H), 4.58 (s, 1H), 4.38 (t, J = 4.1 Hz, 1H), 4.31 (q, J = 7.1 Hz, 2H), 3.36 (td, J = 7.5, 4.4 Hz, 2H), 3.28 (s, 3H), 1.36 (t, J = 7.1 Hz, 3H). ^{13}C NMR (δ, 23 °C, 100 MHz, $CDCl_3$): 167.0, 151.8, 139.4, 131.6, 128.9, 128.5, 126.9, 119.2, 112.0, 82.1, 60.3, 57.1, 50.0, 14.6. HRMS (APCI) m/z: calculated for $[M+H]^+$ = 300.1594, observed $[M+H]^+$ = 300.1589.

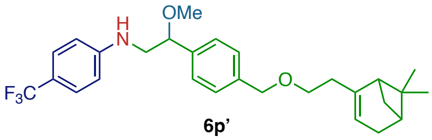

6p'

*N-(2-(4-((2-(6,6-dimethylbicyclo[3.1.1]hept-2-en-2-yl)ethoxy)methyl)phenyl)-2-methoxyethyl)-4-(trifluoromethyl)aniline (**6p'**).* After carrying out the oxyaination reaction according to the General Procedure described in **Section D.3***,* the reaction mixture was subjected to one pot sulfonamide deprotection protocol and the free amine was obtained as a colorless oil (21.5 mg, 55% yield). ^{1}H NMR (δ, 23 °C, 400 MHz, $CDCl_3$): 7.41–7.30 (m, 6H), 6.62 (d, J = 8.6 Hz, 2H), 5.29 (dt, J = 2.8, 1.4 Hz, 1H), 4.51 (s, 2H), 4.37 (dd, J = 7.7, 5.0 Hz, 1H), 3.53 (t, J = 7.1 Hz, 2H), 3.33–3.27 (m, 5H), 2.37–2.29 (m, 3H), 2.25–2.21 (m, 2H), 2.09–2.05 (m, 2H), 1.27 (s, 3H), 1.17 (d, J = 8.5 Hz, 1H), 0.83 (s, 3H). ^{13}C NMR (δ, 23 °C, 100 MHz, $CDCl_3$): 150.6, 145.2, 139.0, 138.7, 128.2, 126.9, 126.8, 126.7, 126.4, 123.7, 119.1, 118.1, 112.4, 81.9, 72.7, 69.3, 57.0, 50.2, 46.0, 40.9, 38.2, 37.3, 31.8, 31.5, 26.5, 21.3. ^{19}F NMR (δ, 23 °C, 376 MHz, $CDCl_3$): –61.0. HRMS (APCI) m/z: calculated for $[M+H]^+$ = 474.2614, observed $[M+H]^+$ = 474.2608.

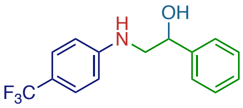

6ac'

*1-phenyl-2-((4-(trifluoromethyl)phenyl)amino)ethan-1-ol (**6ac'**).* After carrying out the hydroxyaination reaction according to the General Procedure described in **Section D.5***,* the reaction mixture was subjected to one pot sulfonamide deprotection protocol and the free amine was obtained as a colorless oil (18.3 mg, 65% yield). ^{1}H NMR (δ, 23 °C, 400 MHz, $CDCl_3$): 7.41 (m, 6H), 7.37–7.34 (m, 1H), 6.66 (d, J = 8.6 Hz, 2H), 4.94 (dd, J = 8.4, 4.1 Hz, 1H), 3.46 (dd, J = 13.2, 4.1 Hz, 1H), 3.36 (dd, J = 13.2, 8.4 Hz, 1H). The obtained spectral data are in good agreement with those reported in literature.[11]

E. X-Ray Diffraction Data

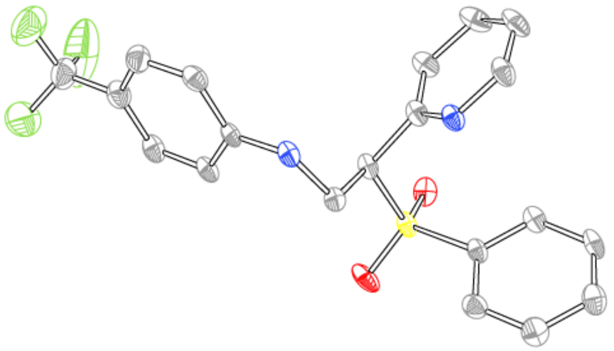

Figure S5. Displacement ellipsoid plot of **3d** plotted at 50% probability. H-atoms and solvent are removed for clarity. The crystalline sample used in this diffraction experiment was obtained from a concentrated Et_2O solution at −5 °C.

Table S1. X-ray experimental details of **3d** (CCDC 2367116).

Crystal data	
Chemical formula	$C_{20}H_{17}F_3N_2O_2S$
M_r	406.41
Crystal system, space group	Triclinic, $P\bar{1}$
Temperature (K)	100
a, *b*, *c* (Å)	10.9322(4), 12.9536(4), 13.8256(7)
α, β, γ (°)	108.451(4), 91.843(4), 91.343(3)
V (Å^3)	1855.1(1)
Z	4
Radiation type	Cu *K*α
μ (mm^{-1})	1.99
Crystal size (mm)	0.1 × 0.05 × 0.03
Data collection	
Diffractometer	XtaLAB Synergy, Dualflex, HyPix
Absorption correction	Multi-scan<br>*CrysAlis PRO* 1.171.42.101a (Rigaku Oxford Diffraction, 2023)<br>Empirical absorption correction using spherical harmonics, implemented in SCALE3 ABSPACK scaling algorithm.
T_{min}, T_{max}	0.688, 1.000
No. of measured, independent and observed [$I > 2\sigma(I)$] reflections	32252, 7183, 6398
R_{int}	0.052
$(\sin \theta/\lambda)_{max}$ (Å^{-1})	0.628
Refinement	
$R[F^2 > 2\sigma(F^2)]$, $wR(F^2)$, S	0.118, 0.327, 1.48
No. of reflections	7183
No. of parameters	505
H-atom treatment	H-atom parameters constrained
$\Delta\rho_{max}$, $\Delta\rho_{min}$ (e Å^{-3})	2.90, −0.77

F. Additional Data

F.1 Summary of Unproductive Substrates

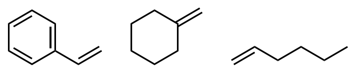

Figure S6. Summary of olefins that do not participate in productive olefin aminopyridylation reaction under the optimized reaction conditions.

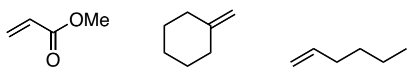

Figure S7. Summary of olefins that do not participate in productive olefin oxyamination reaction under the optimized reaction conditions.

F.2 Comparison of the Excited State Redox Potentials of the Photocatalysts Used[12]

Entry	Substrate/Photocatalyst	Reduction Potential (V vs SCE)	Oxidation Potential (V vs SCE)
1	**1a**	-0.86	–
2	**1a’**	–	+0.45
3	$Ir(dtbpy)(ppy)_2PF_6$*	-0.96	+0.66
4	$Ru(bpy)_3Cl_2 \cdot 6H_2O$*	-0.81	+0.77
5	*fac*-$Ir(ppy)_3$*	-1.88	+0.35

G. NMR Spectra for New Compounds

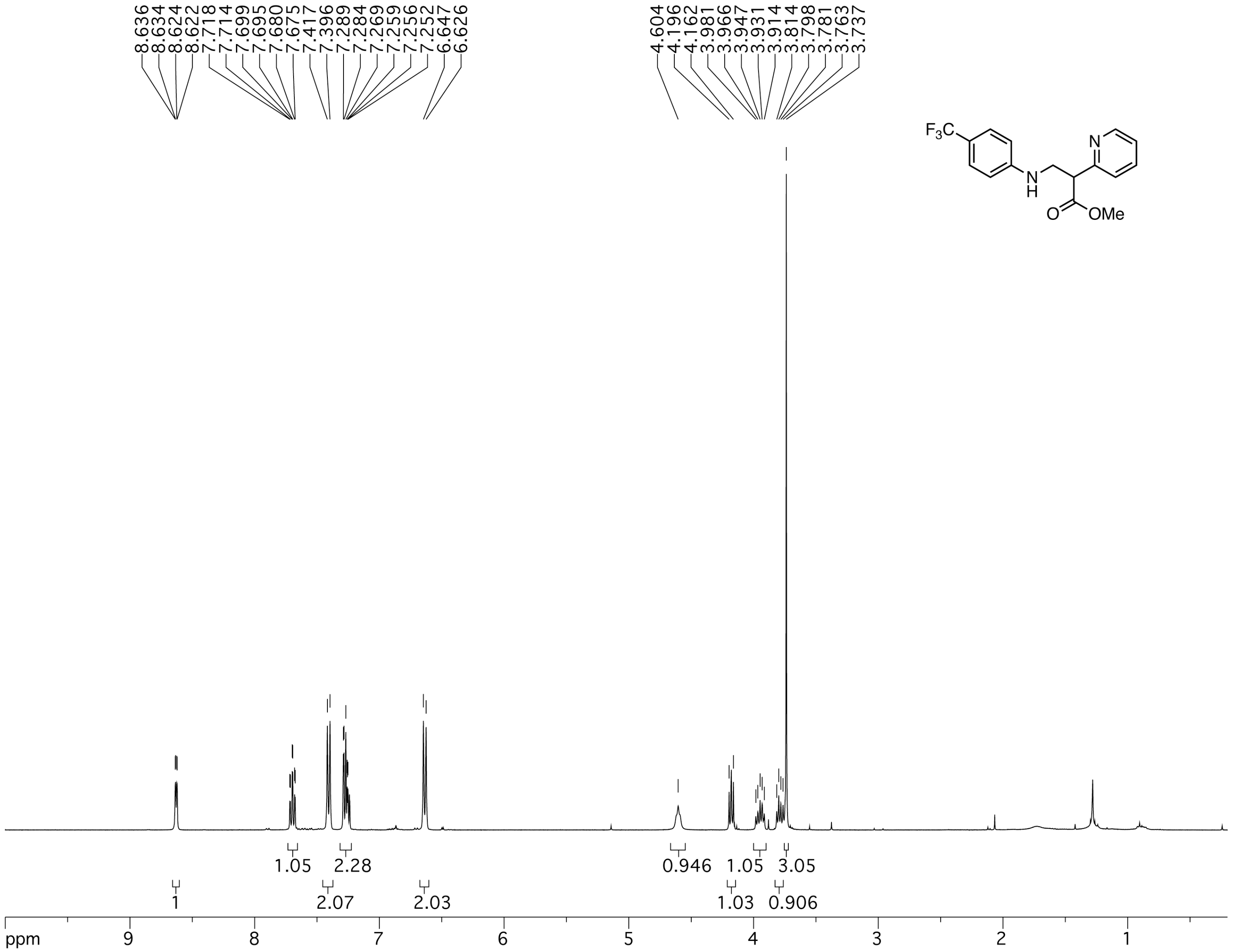

Figure S8. ^{1}H NMR spectrum of **3a** in $CDCl_3$ (400 MHz) measured at 23 °C.

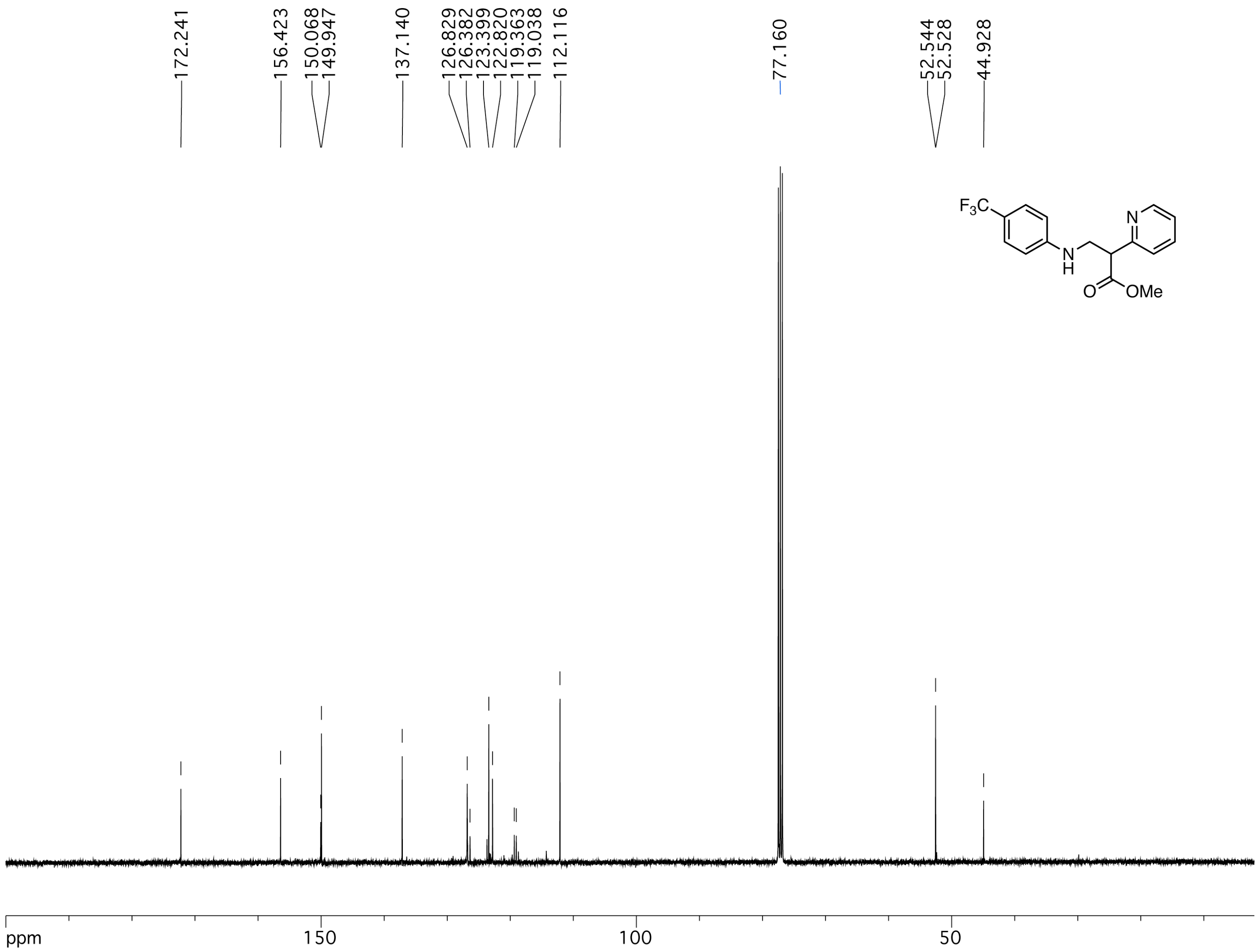

Figure S9. ^{13}C NMR spectrum of **3a** in $CDCl_3$ (100 MHz) measured at 23 °C.

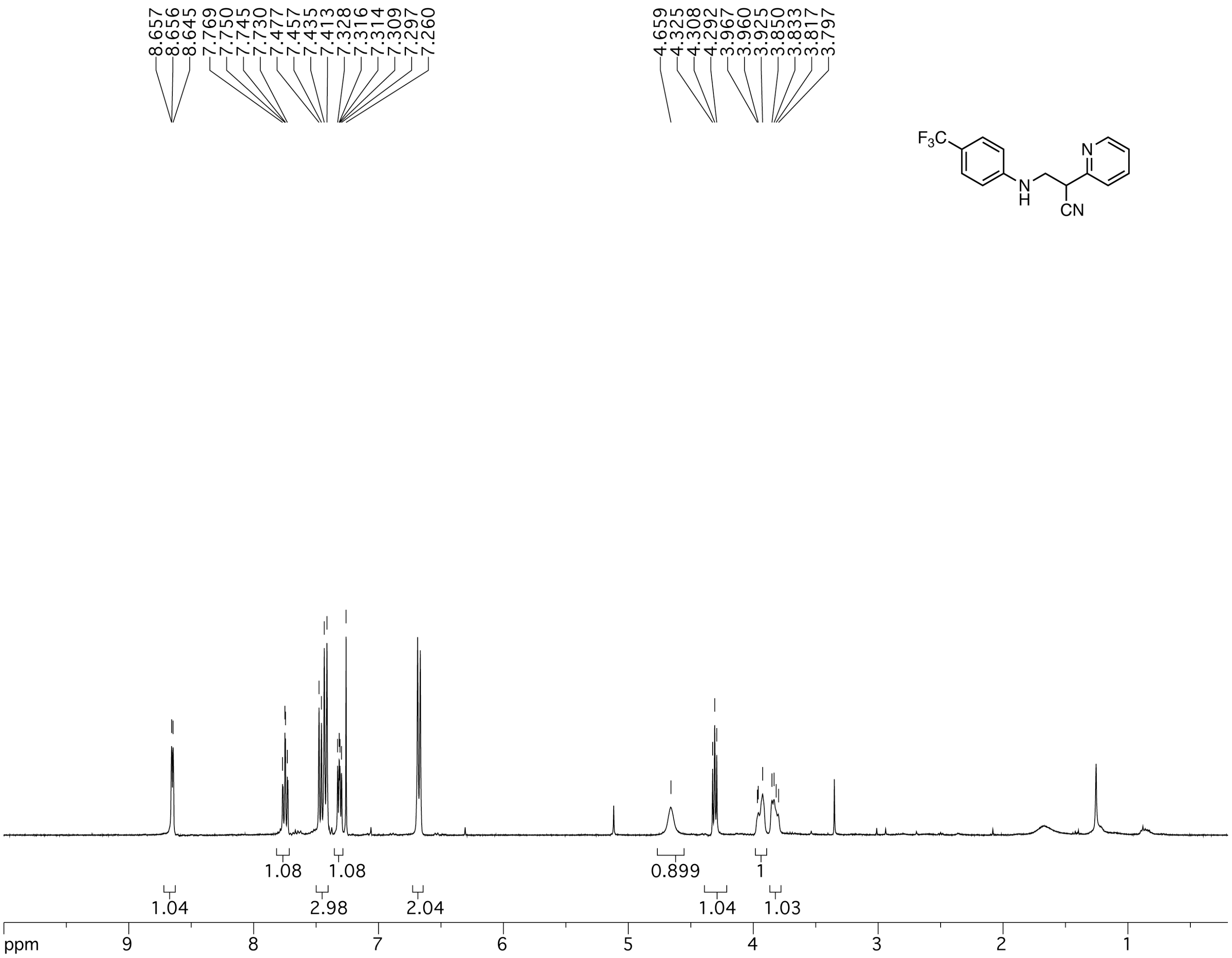

Figure S10. ^{1}H NMR spectrum of **3b** in $CDCl_3$ (400 MHz) measured at 23 °C.

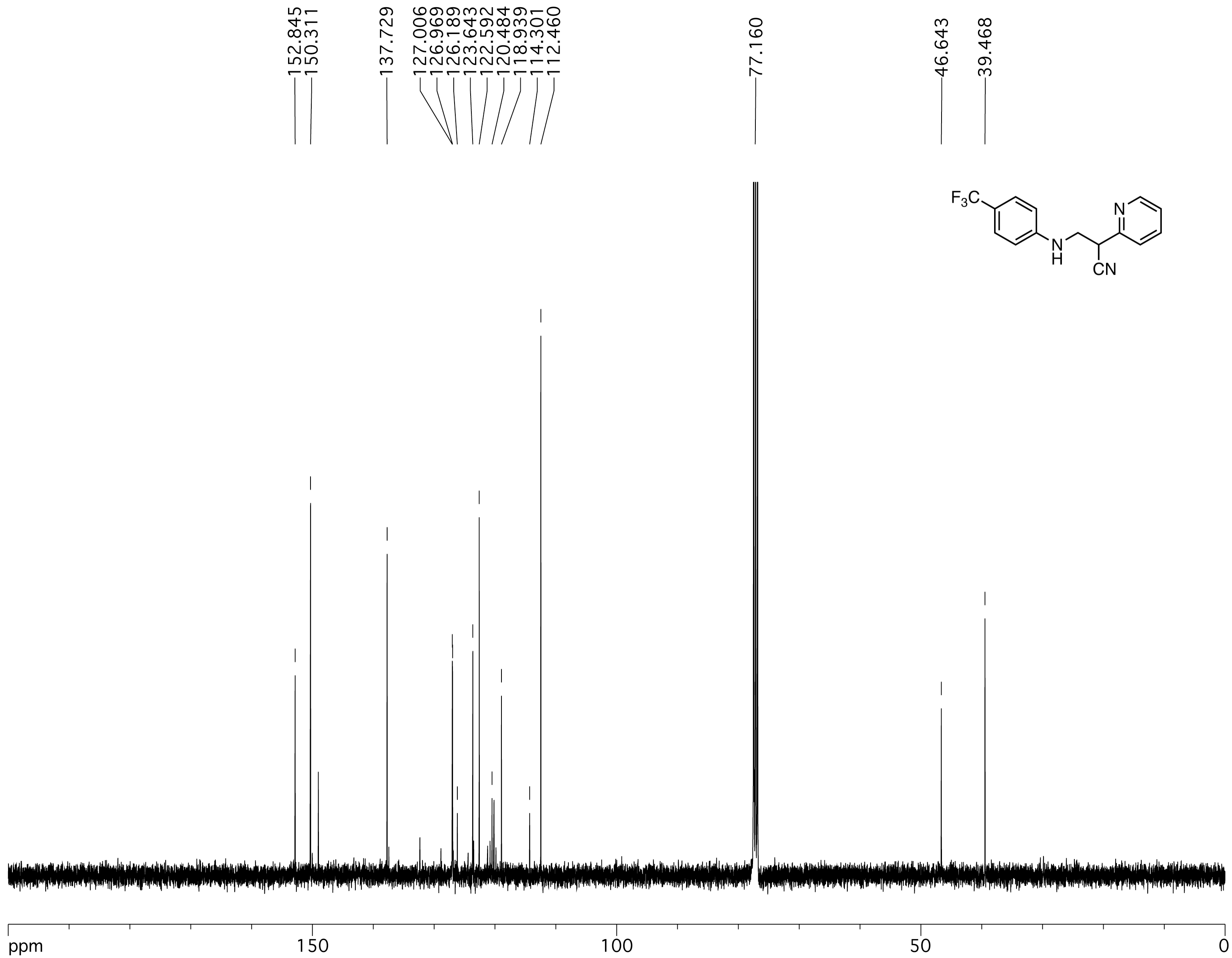

Figure S11. ^{13}C NMR spectrum of **3b** in $CDCl_3$ (100 MHz) measured at 23 °C.

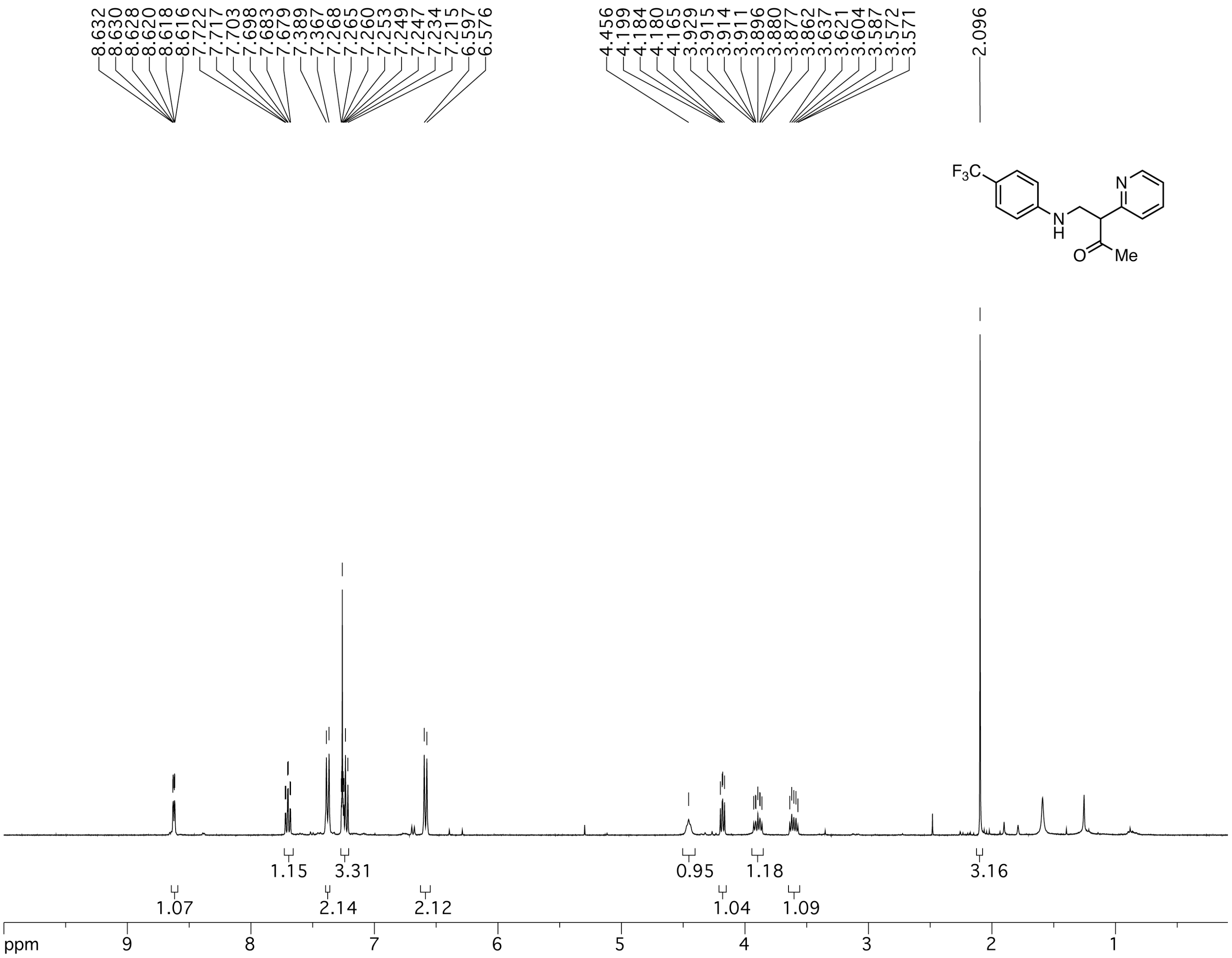

Figure S12. ^{1}H NMR spectrum of **3c** in $CDCl_3$ (400 MHz) measured at 23 °C.

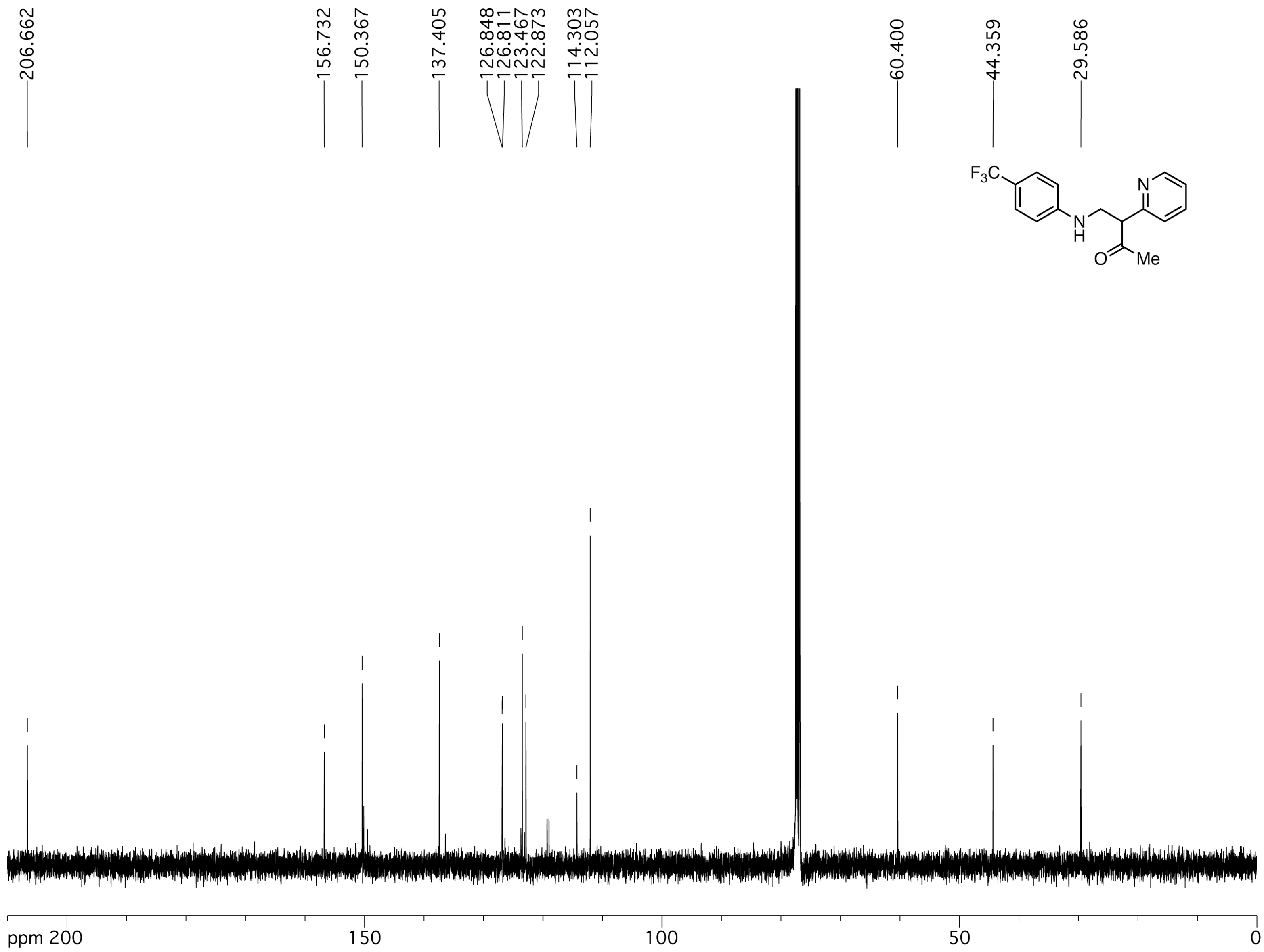

Figure S13. ^{13}C NMR spectrum of **3c** in $CDCl_3$ (100 MHz) measured at 23 °C.

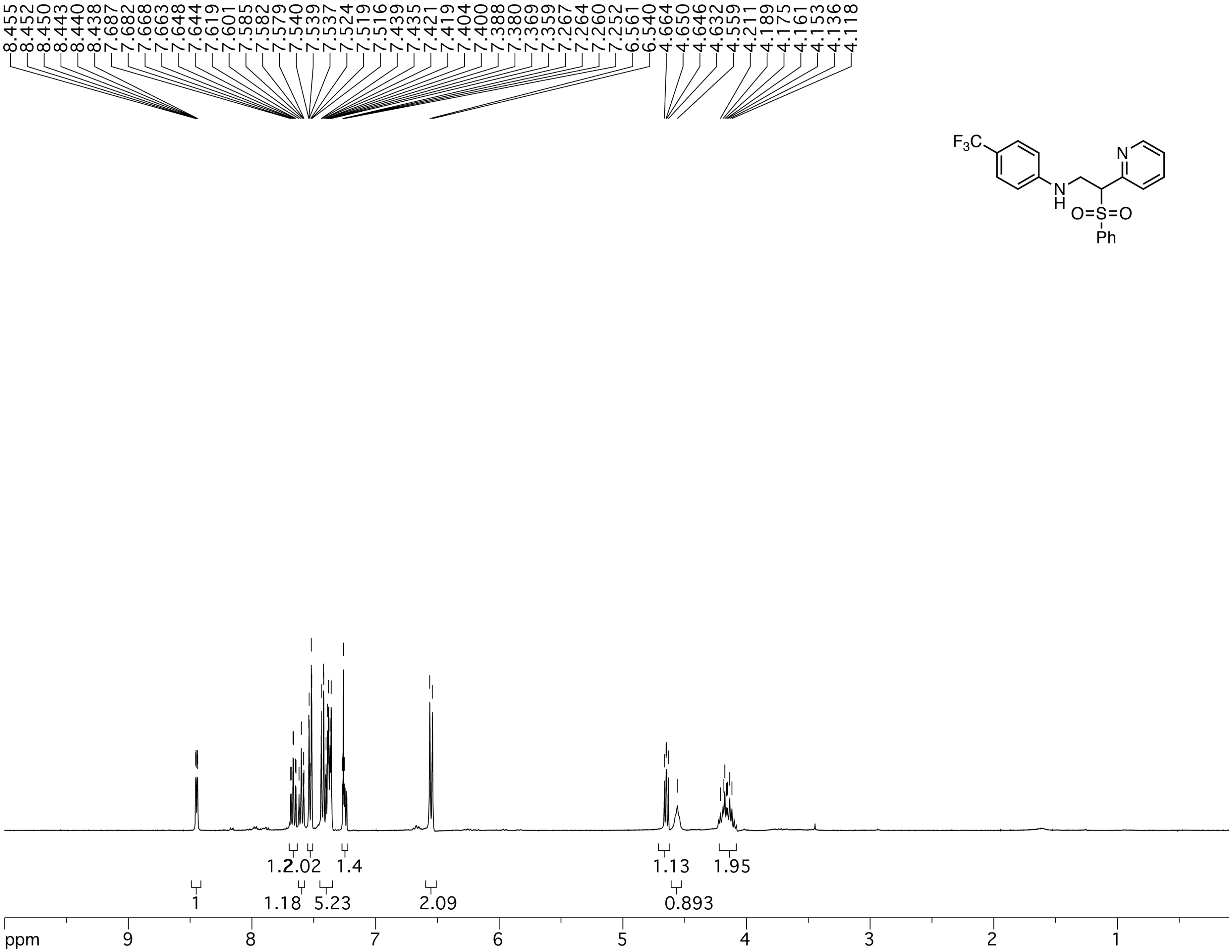

Figure S14. ^{1}H NMR spectrum of **3d** in $CDCl_3$ (400 MHz) measured at 23 °C.

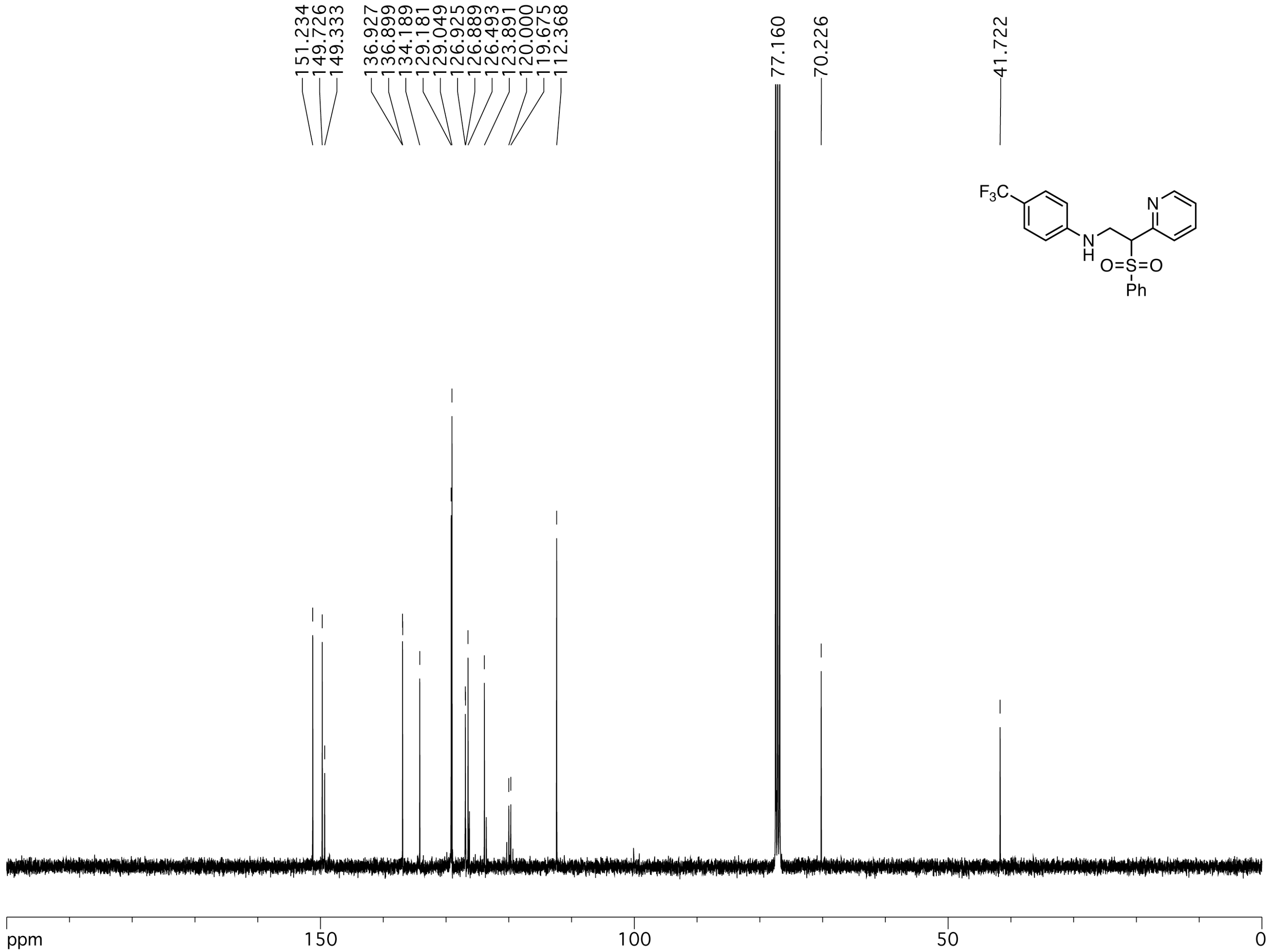

Figure S15. ^{13}C NMR spectrum of **3d** in $CDCl_3$ (100 MHz) measured at 23 °C.

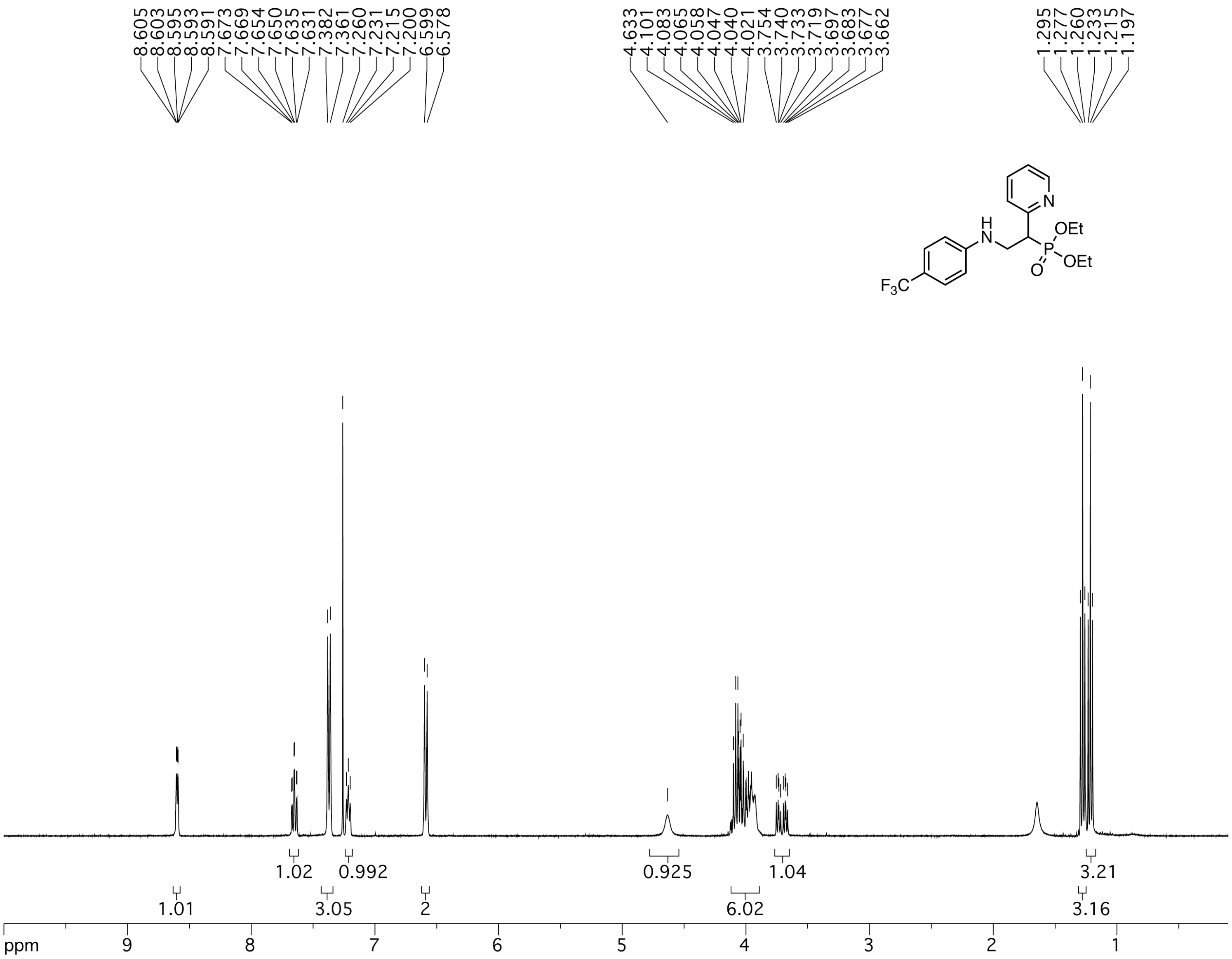

Figure S16. ^{1}H NMR spectrum of **3e** in $CDCl_3$ (400 MHz) measured at 23 °C.

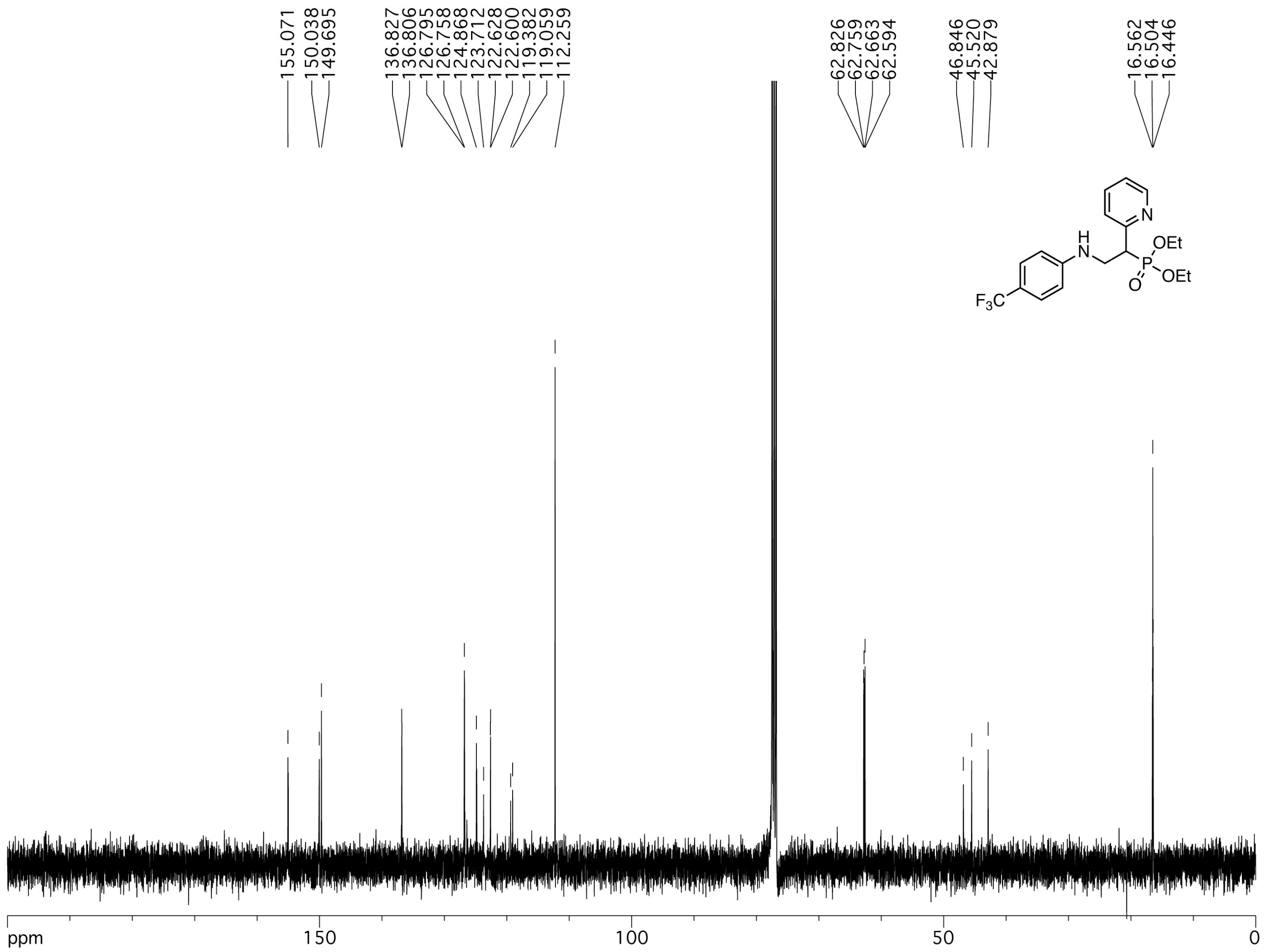

Figure S17. ^{13}C NMR spectrum of **3e** in $CDCl_3$ (100 MHz) measured at 23 °C.

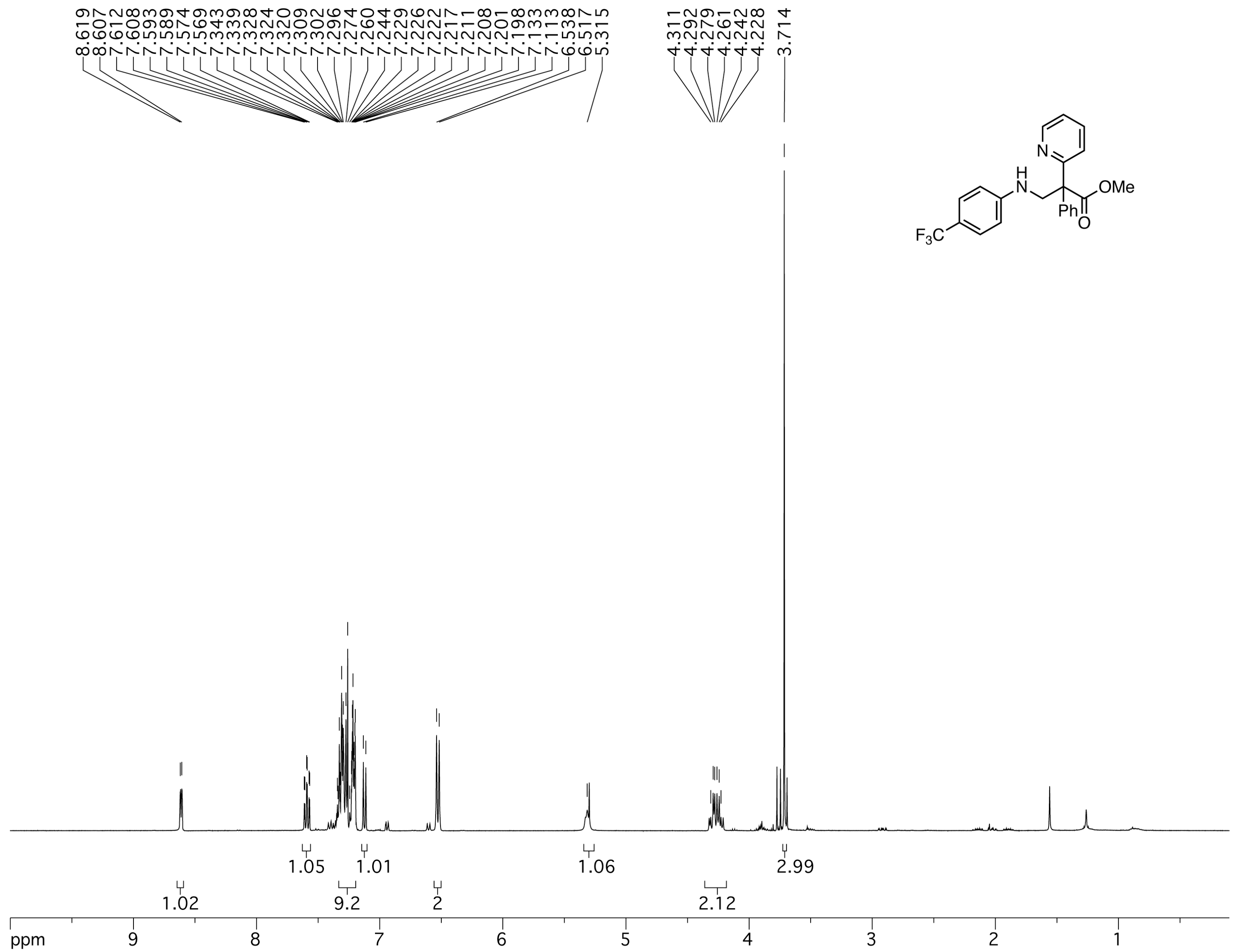

Figure S18. ^{1}H NMR spectrum of **3f** in $CDCl_3$ (400 MHz) measured at 23 °C.

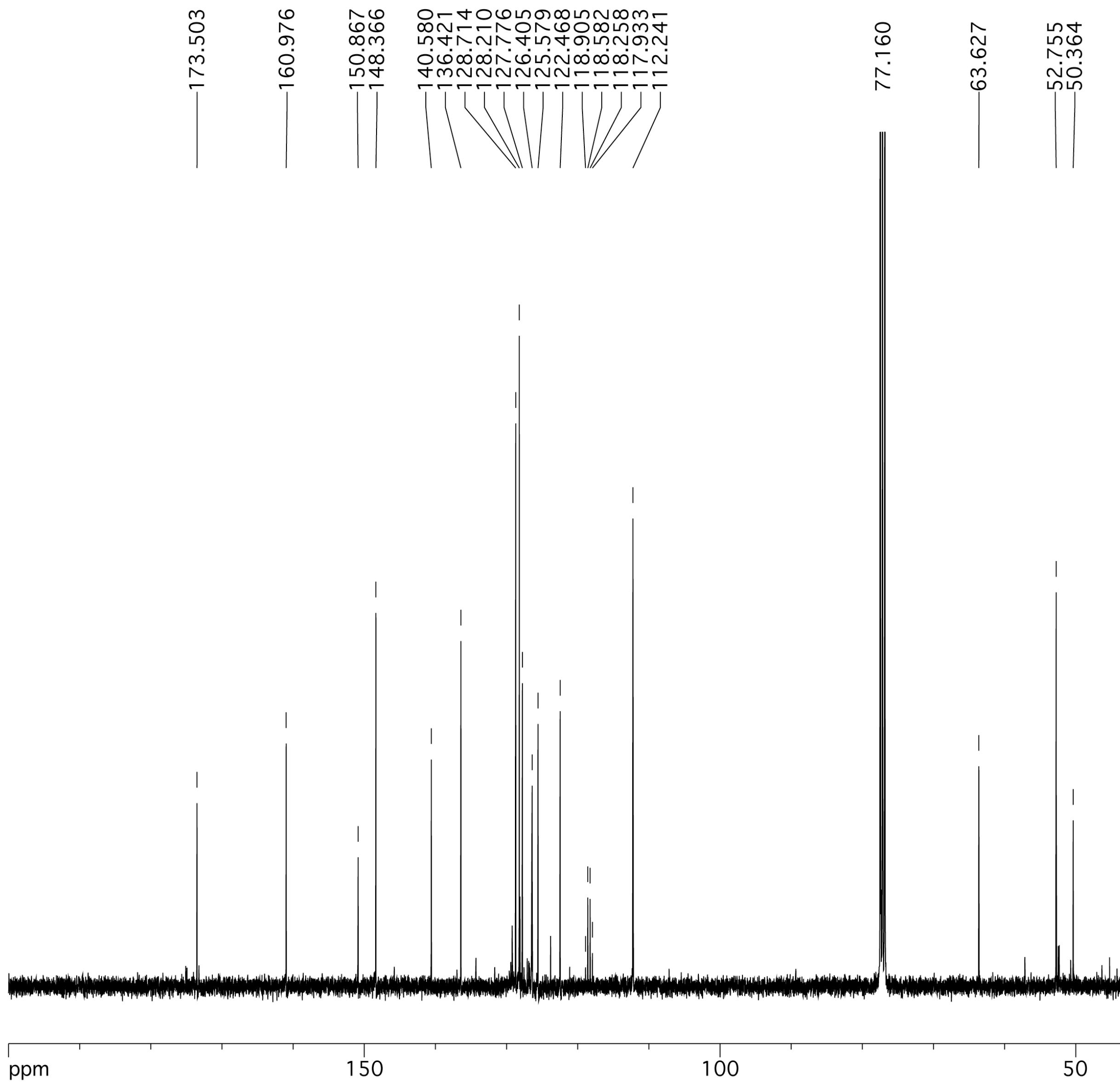

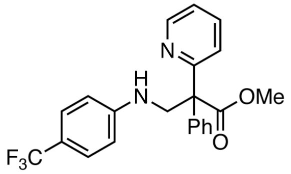

Figure S19. ^{13}C NMR spectrum of **3f** in $CDCl_3$ (100 MHz) measured at 23 °C.

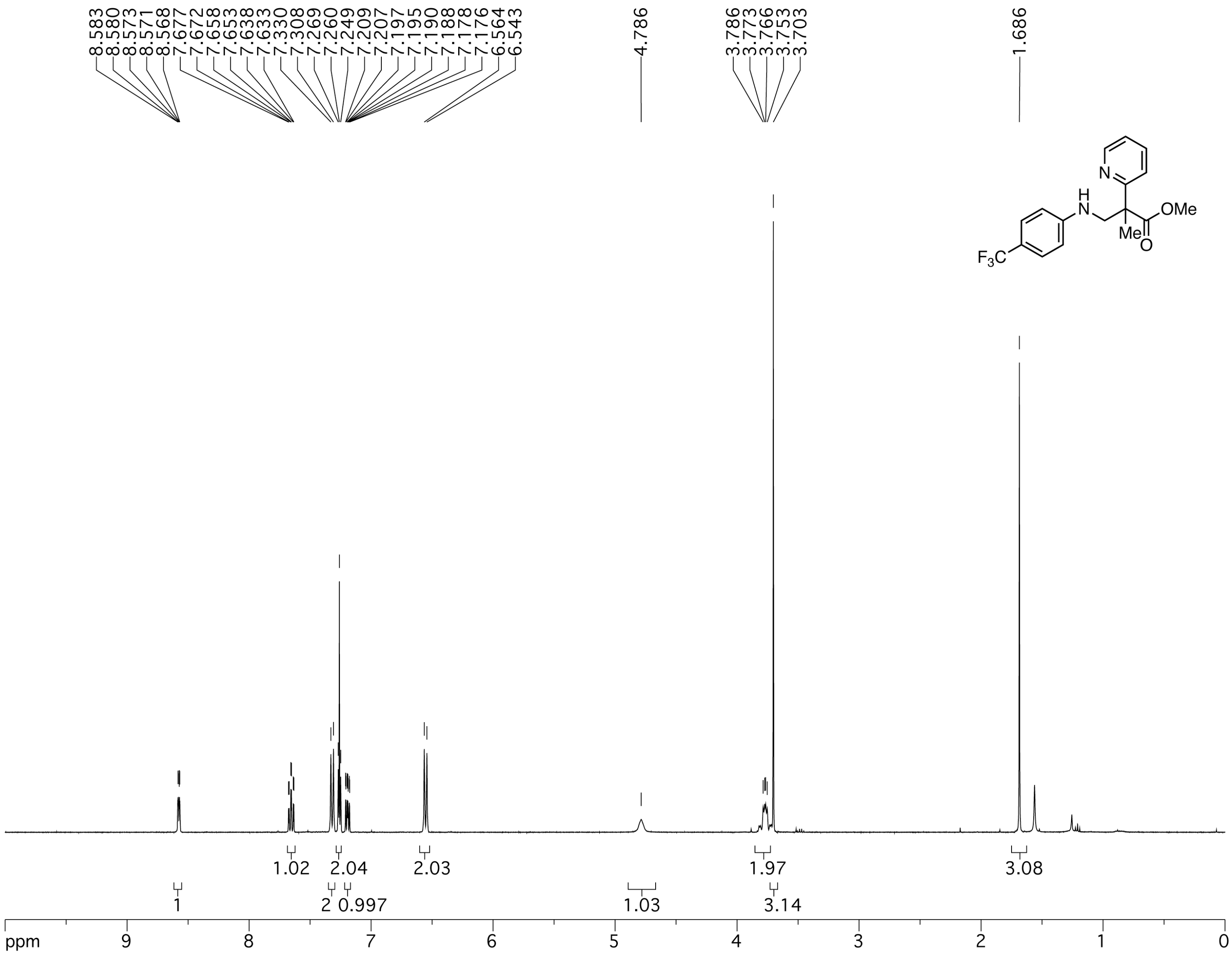

Figure S20. ^{1}H NMR spectrum of **3g** in $CDCl_3$ (400 MHz) measured at 23 °C.

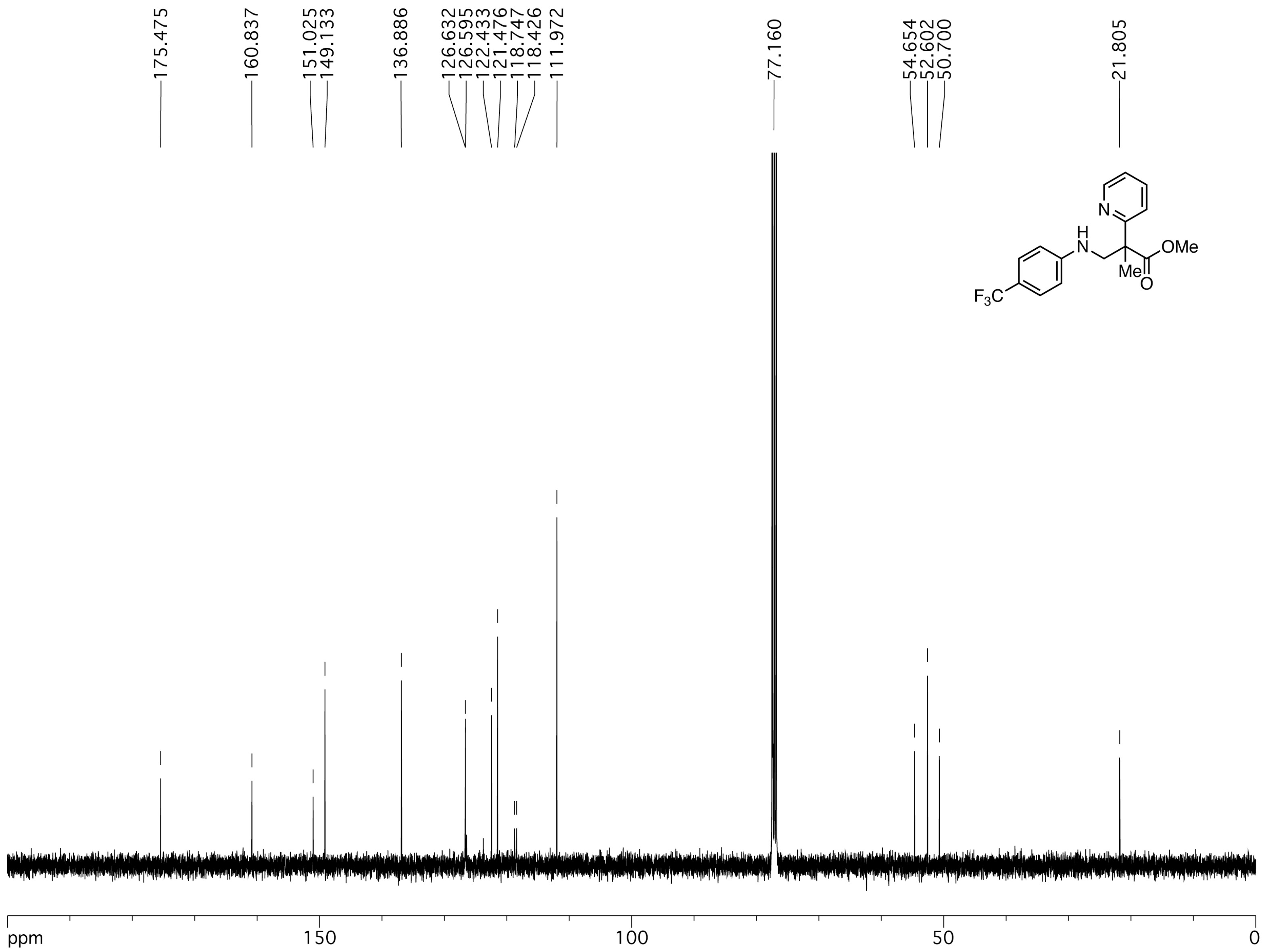

Figure S21. ^{13}C NMR spectrum of **3g** in $CDCl_3$ (100 MHz) measured at 23 °C.

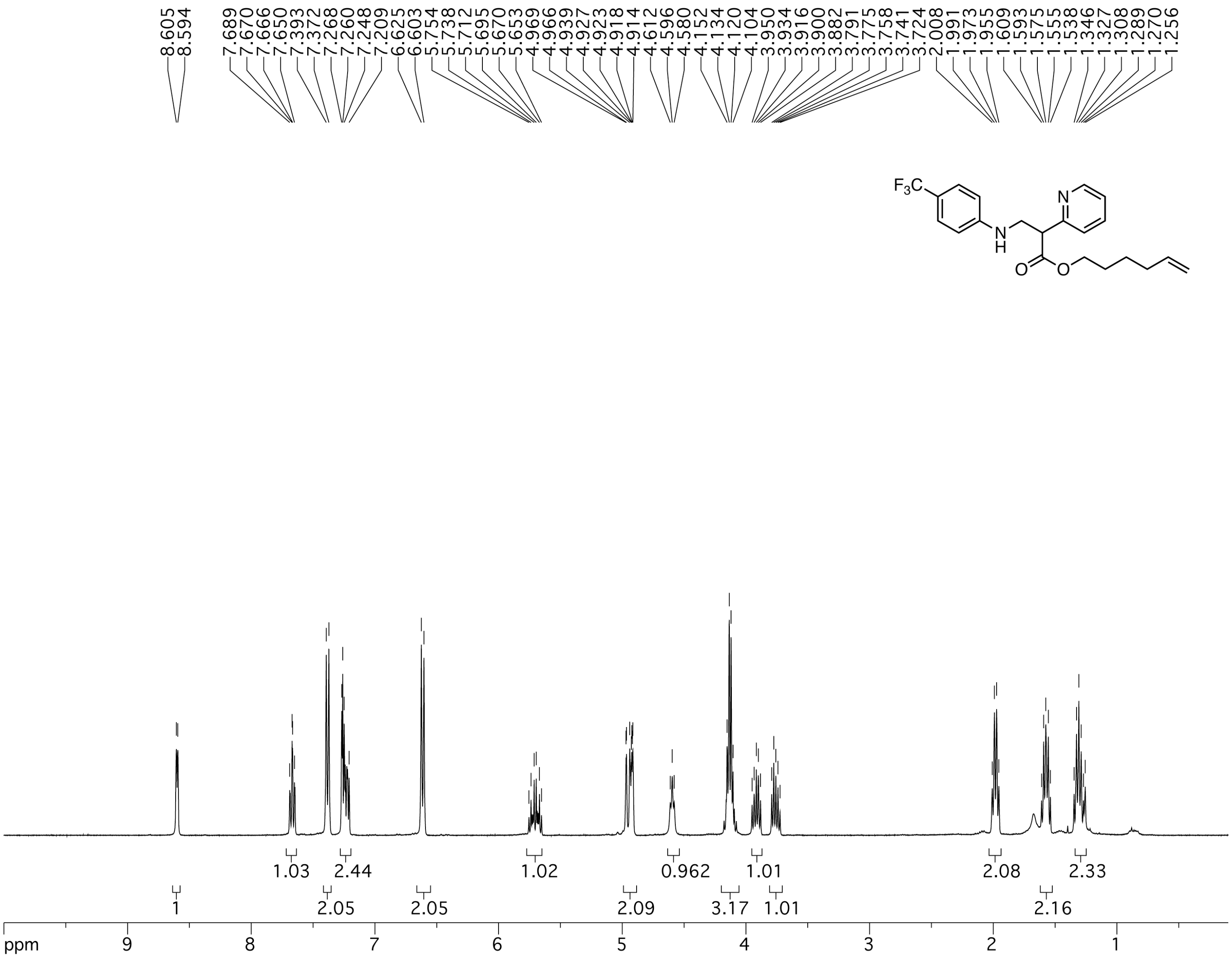

Figure S22. ^{1}H NMR spectrum of **3i** in $CDCl_3$ (400 MHz) measured at 23 °C.

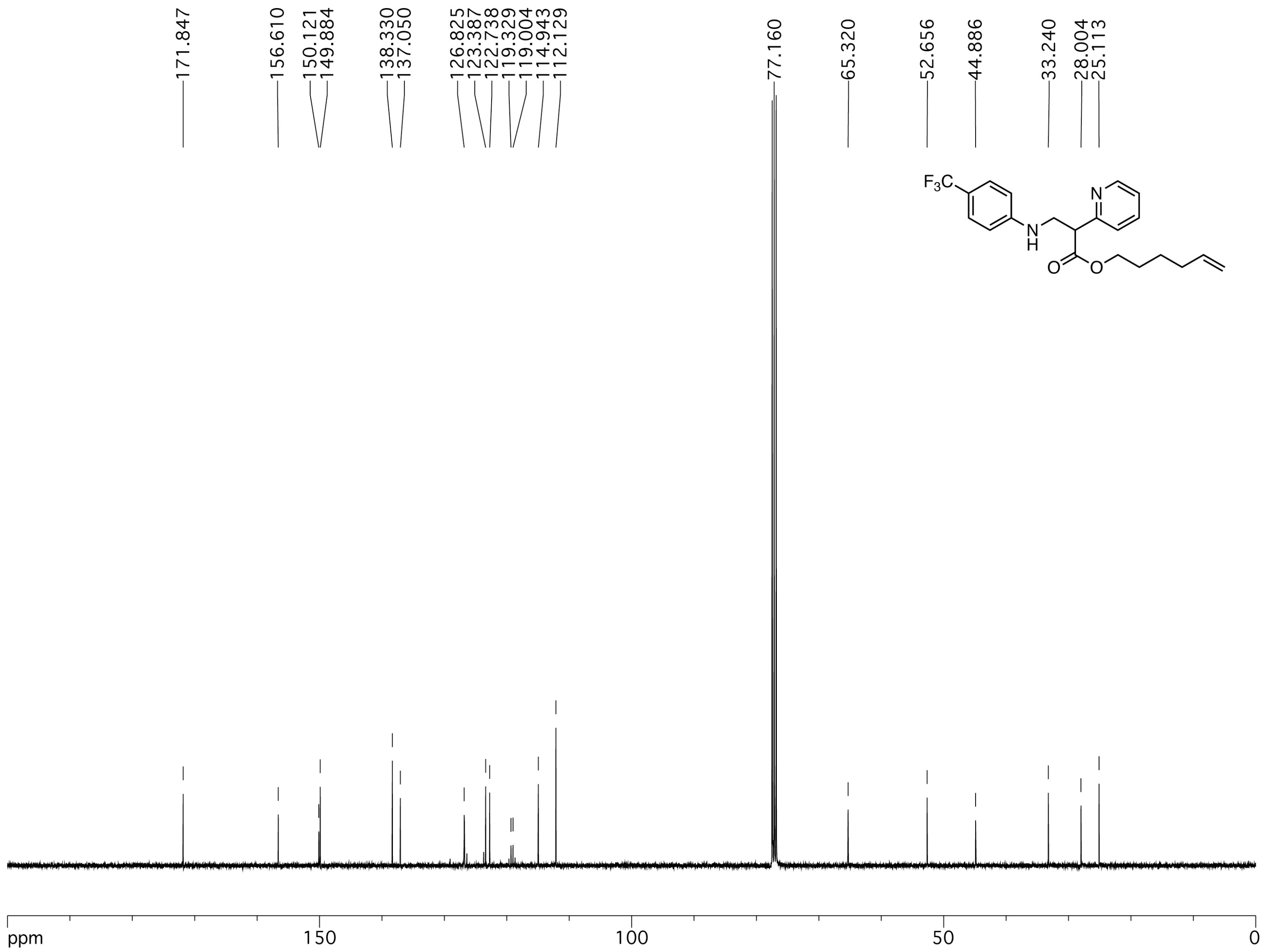

Figure S23. ^{13}C NMR spectrum of **3i** in $CDCl_3$ (100 MHz) measured at 23 °C.

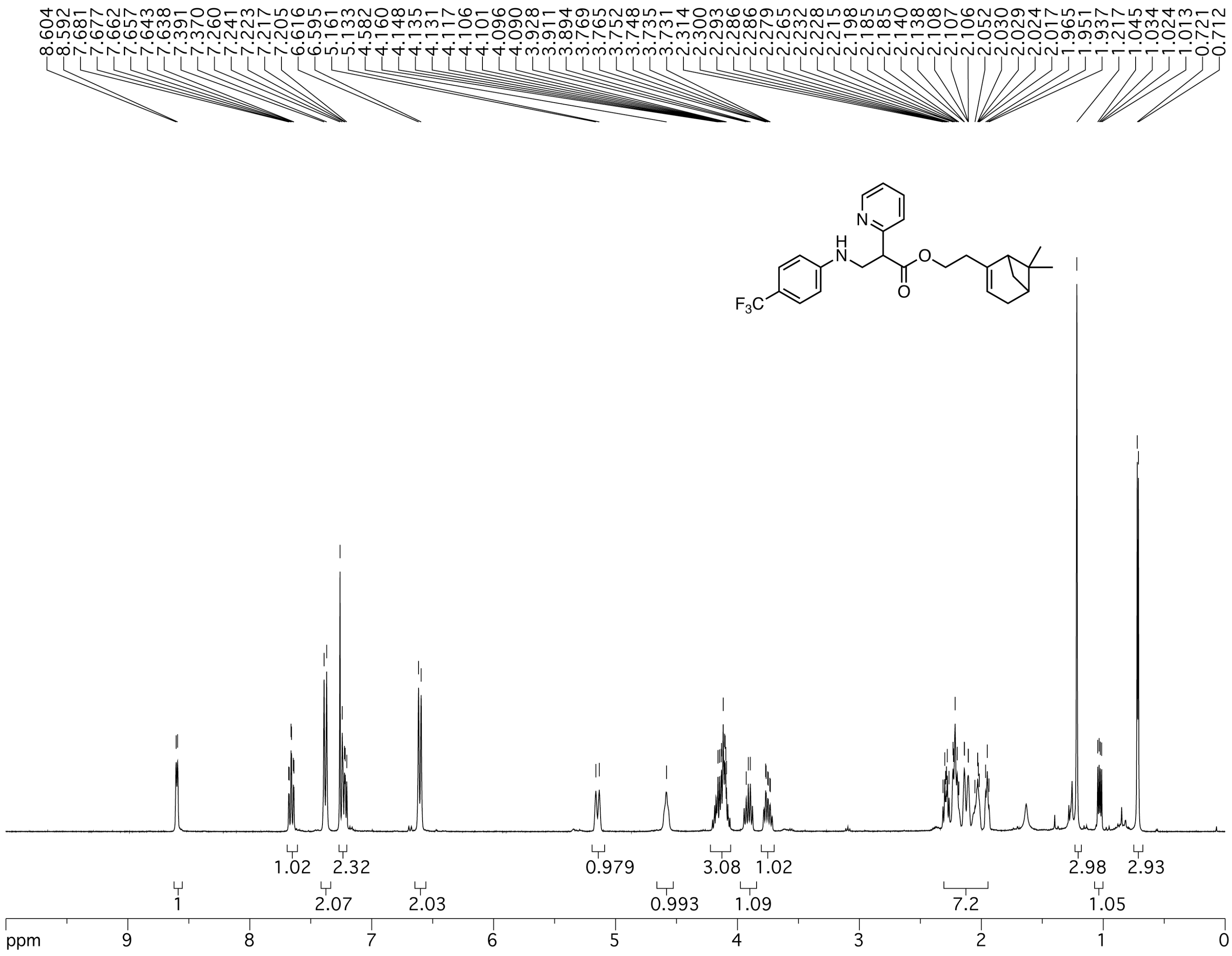

Figure S24. ^{1}H NMR spectrum of **3j** in $CDCl_3$ (400 MHz) measured at 23 °C.

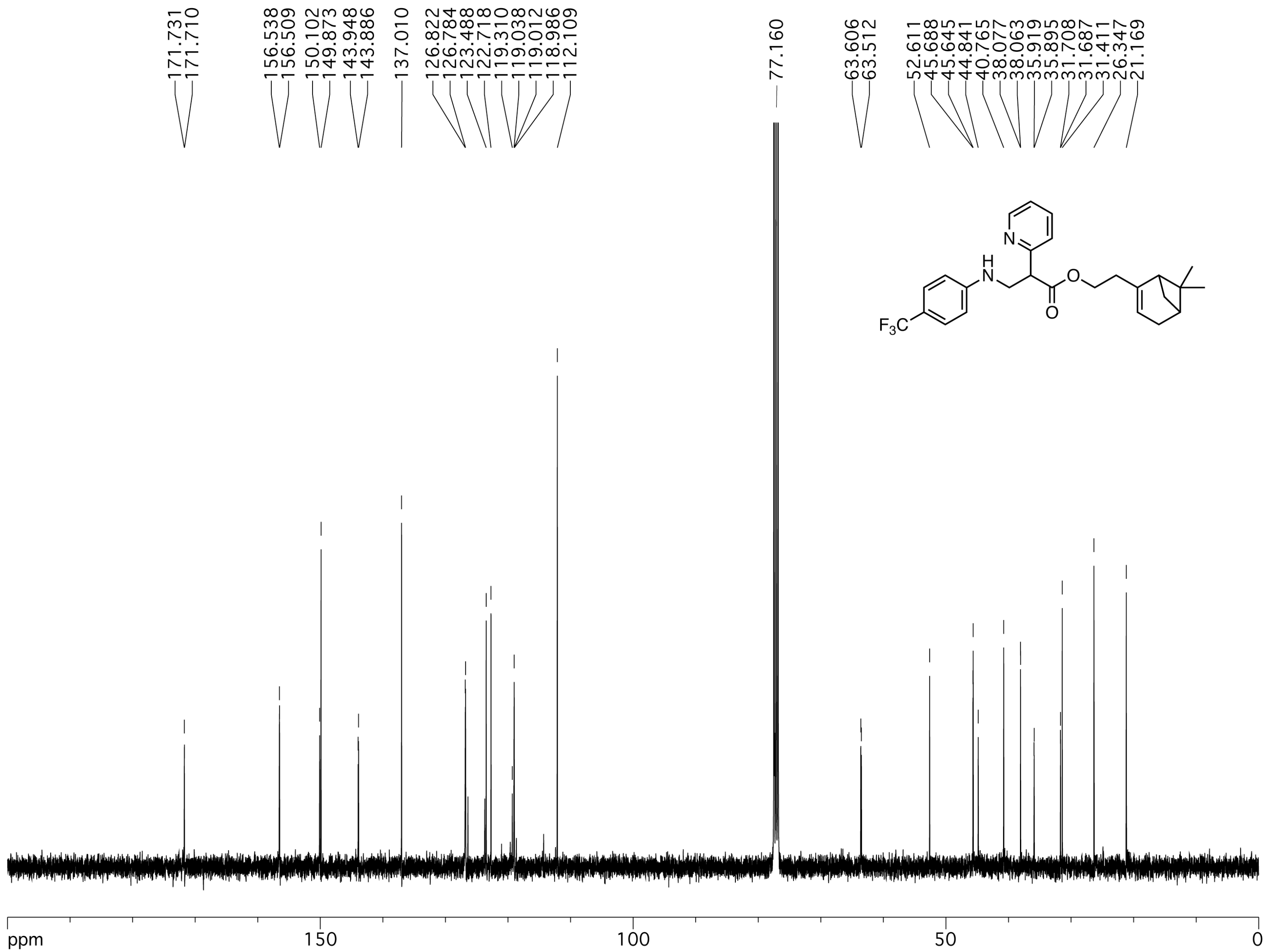

Figure S25. ^{13}C NMR spectrum of **3j** in $CDCl_3$ (100 MHz) measured at 23 °C.

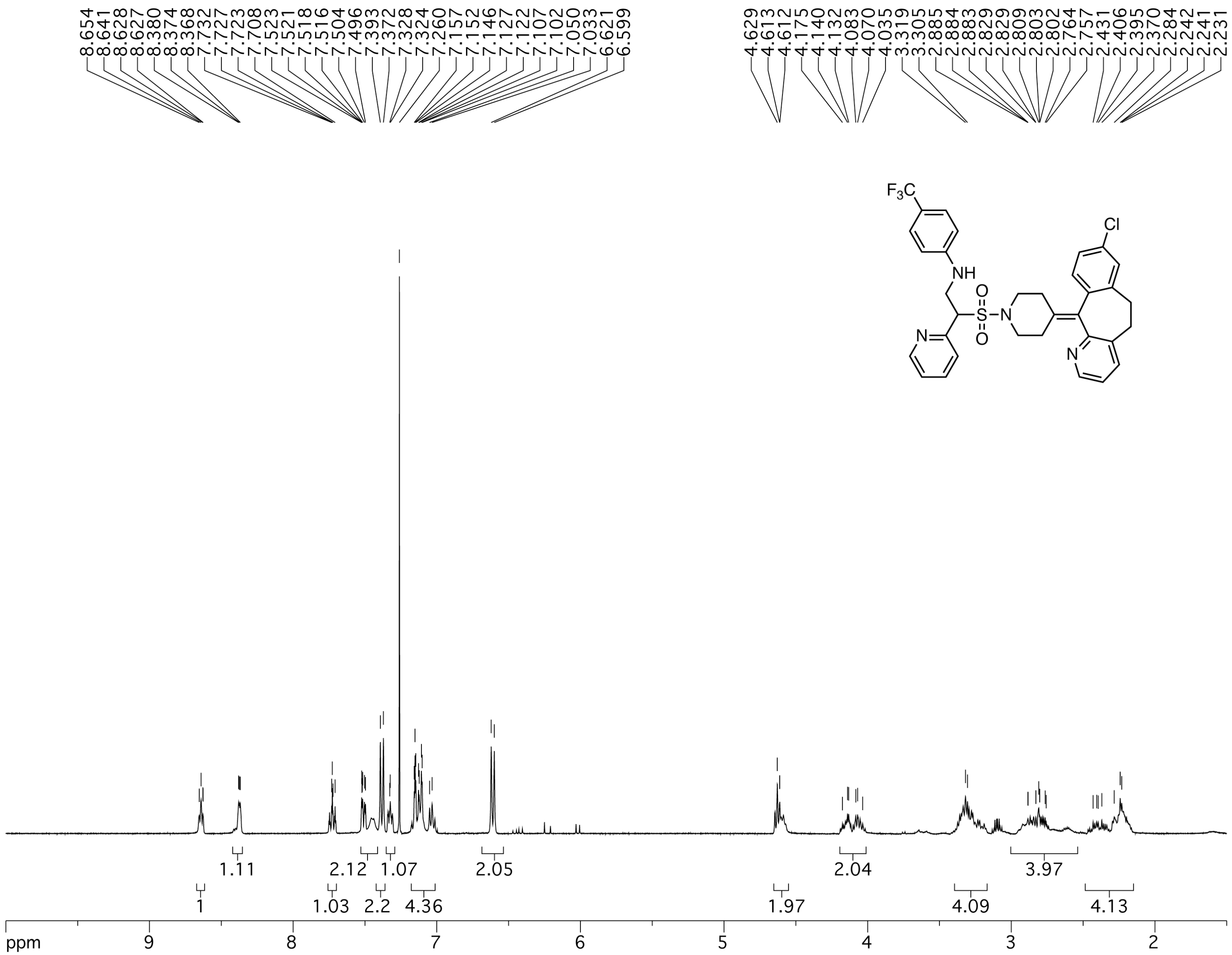

Figure S26. ^{1}H NMR spectrum of **3k** in $CDCl_3$ (400 MHz) measured at 23 °C.

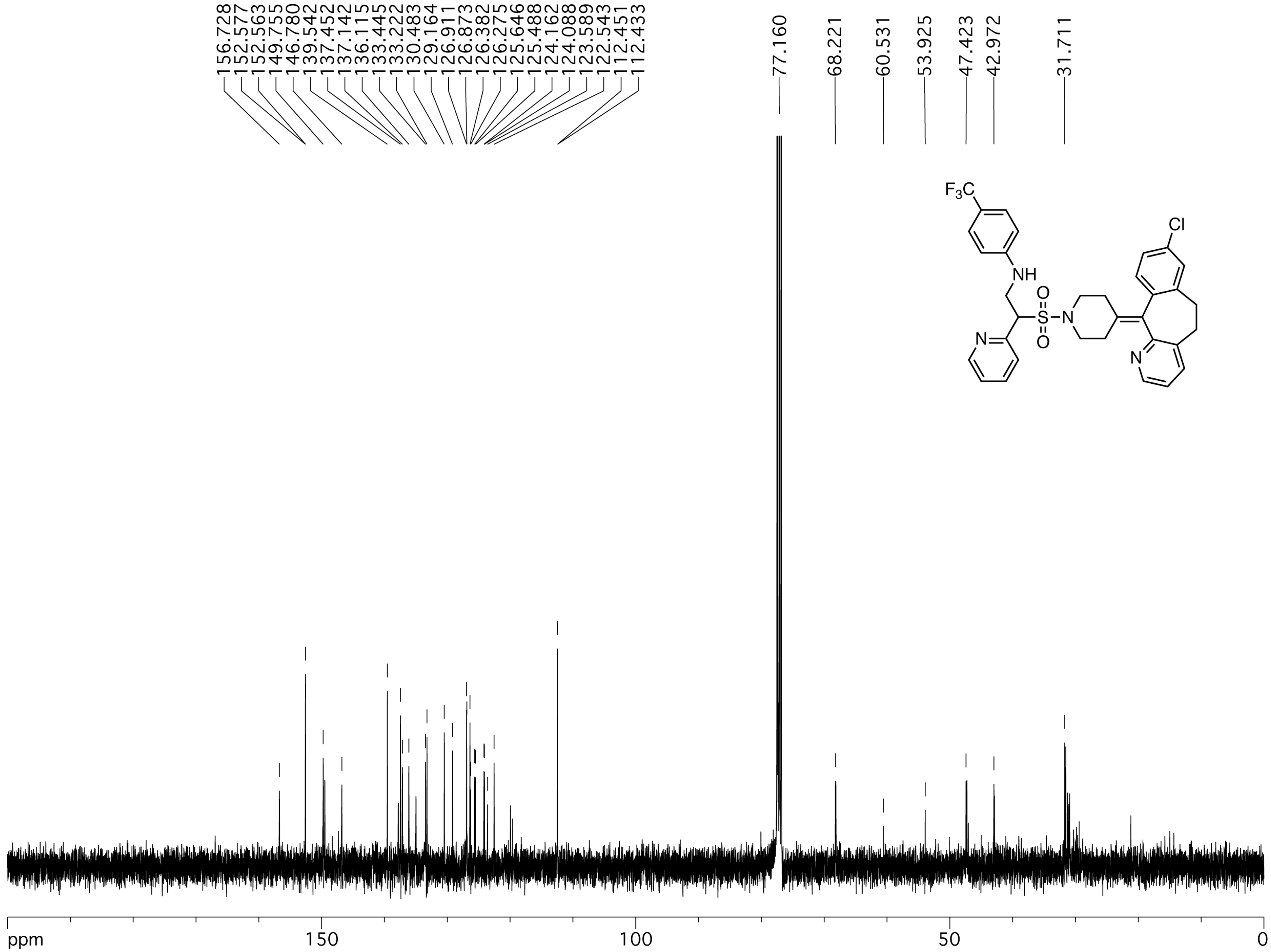

Figure S27. ^{13}C NMR spectrum of **3k** in $CDCl_3$ (100 MHz) measured at 23 °C.

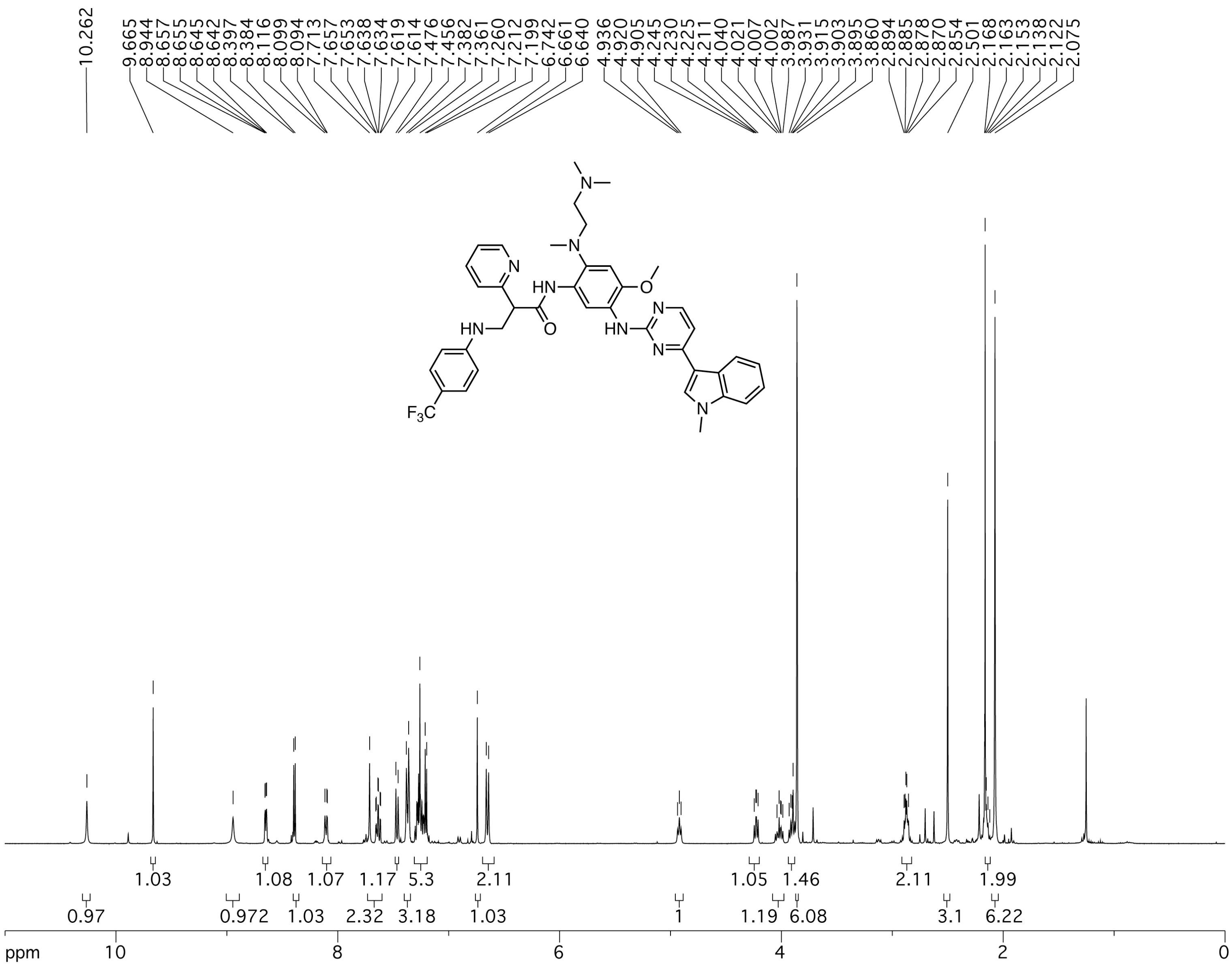

Figure S28. ^{1}H NMR spectrum of **3l** in $CDCl_3$ (400 MHz) measured at 23 °C.

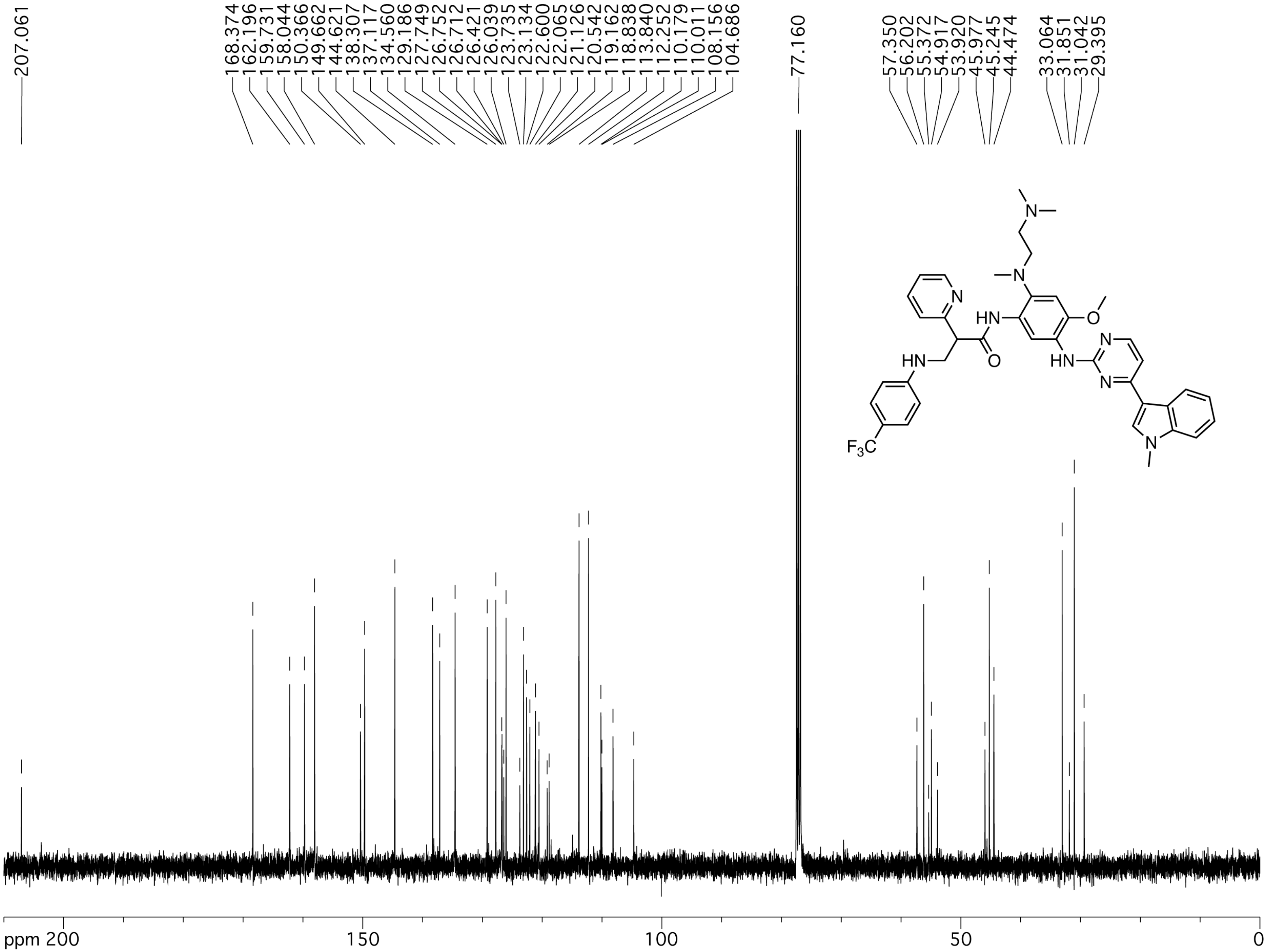

Figure S29. ^{13}C NMR spectrum of **3l** in $CDCl_3$ (100 MHz) measured at 23 °C.

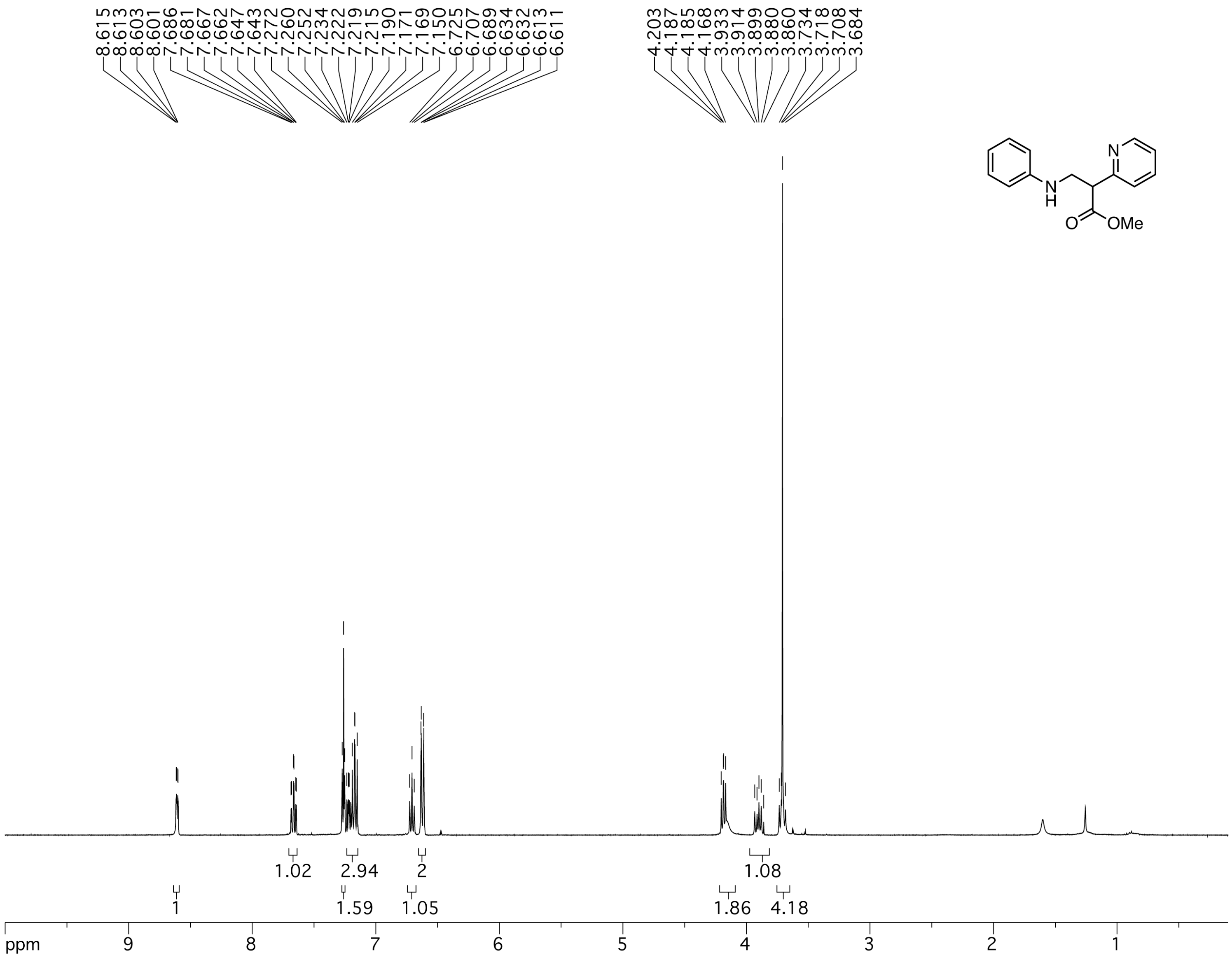

Figure S30. ^{1}H NMR spectrum of **3m** in $CDCl_3$ (400 MHz) measured at 23 °C.

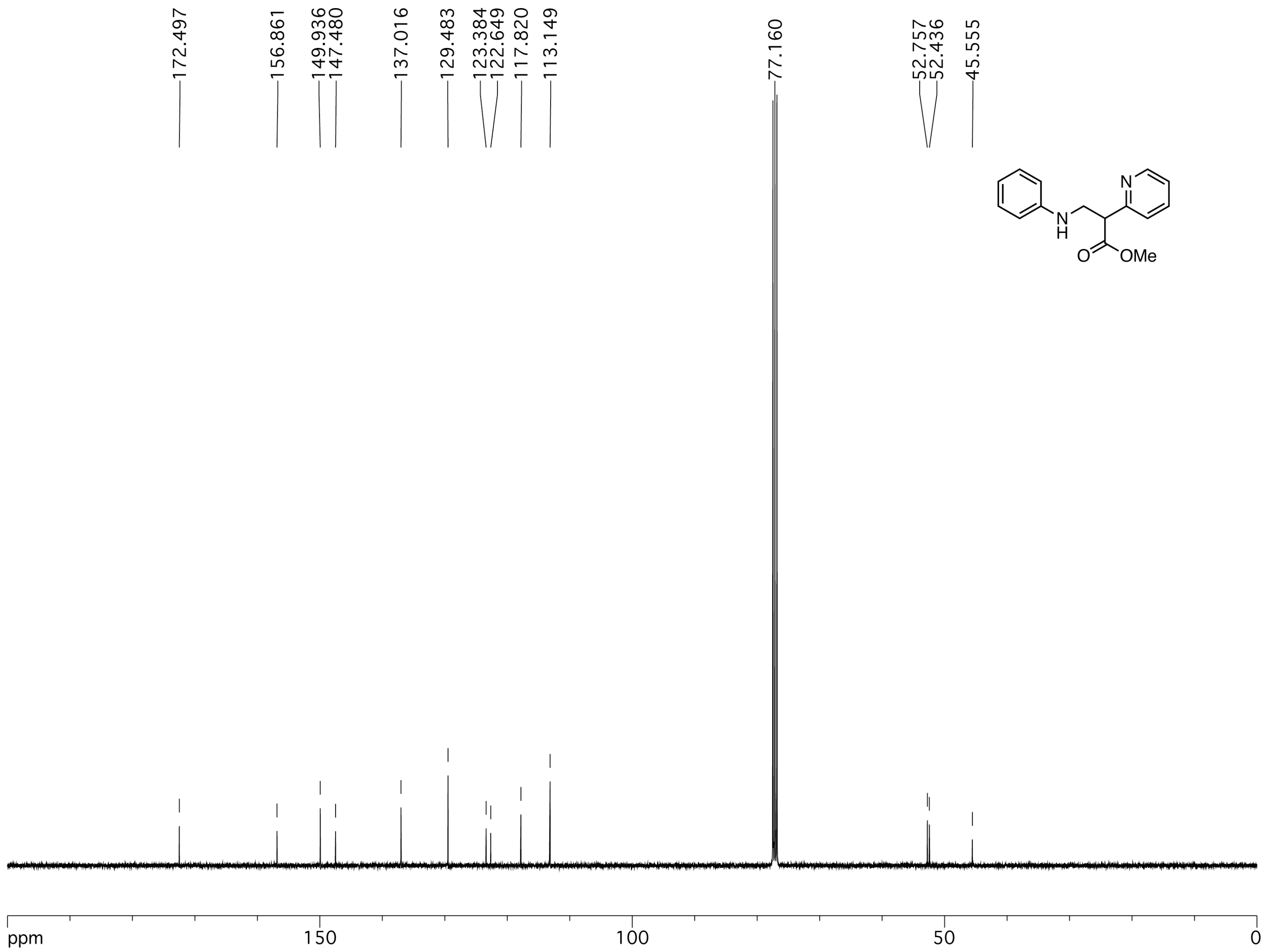

Figure S31. ^{13}C NMR spectrum of **3m** in $CDCl_3$ (100 MHz) measured at 23 °C.

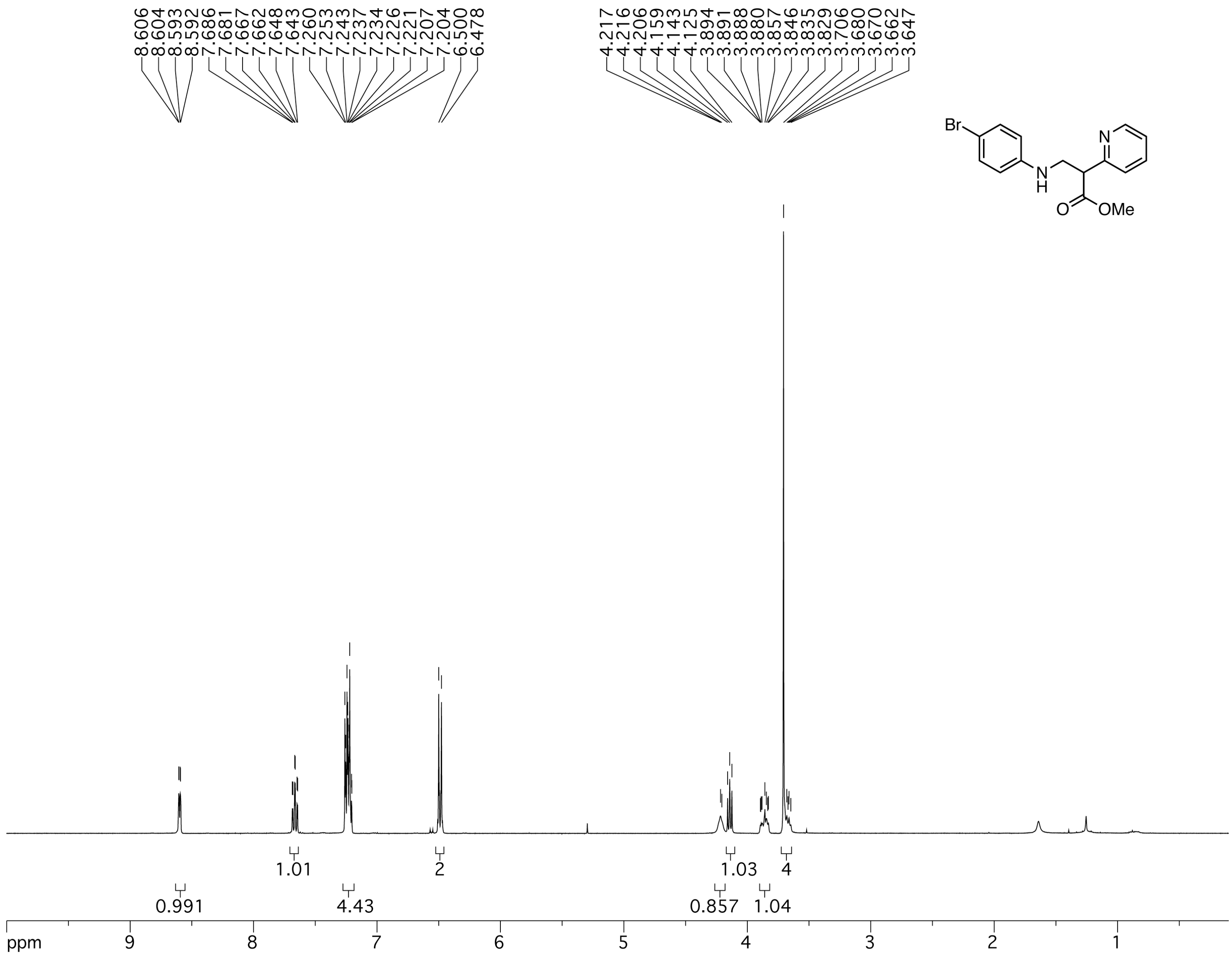

Figure S32. ^{1}H NMR spectrum of **3n** in $CDCl_3$ (400 MHz) measured at 23 °C.

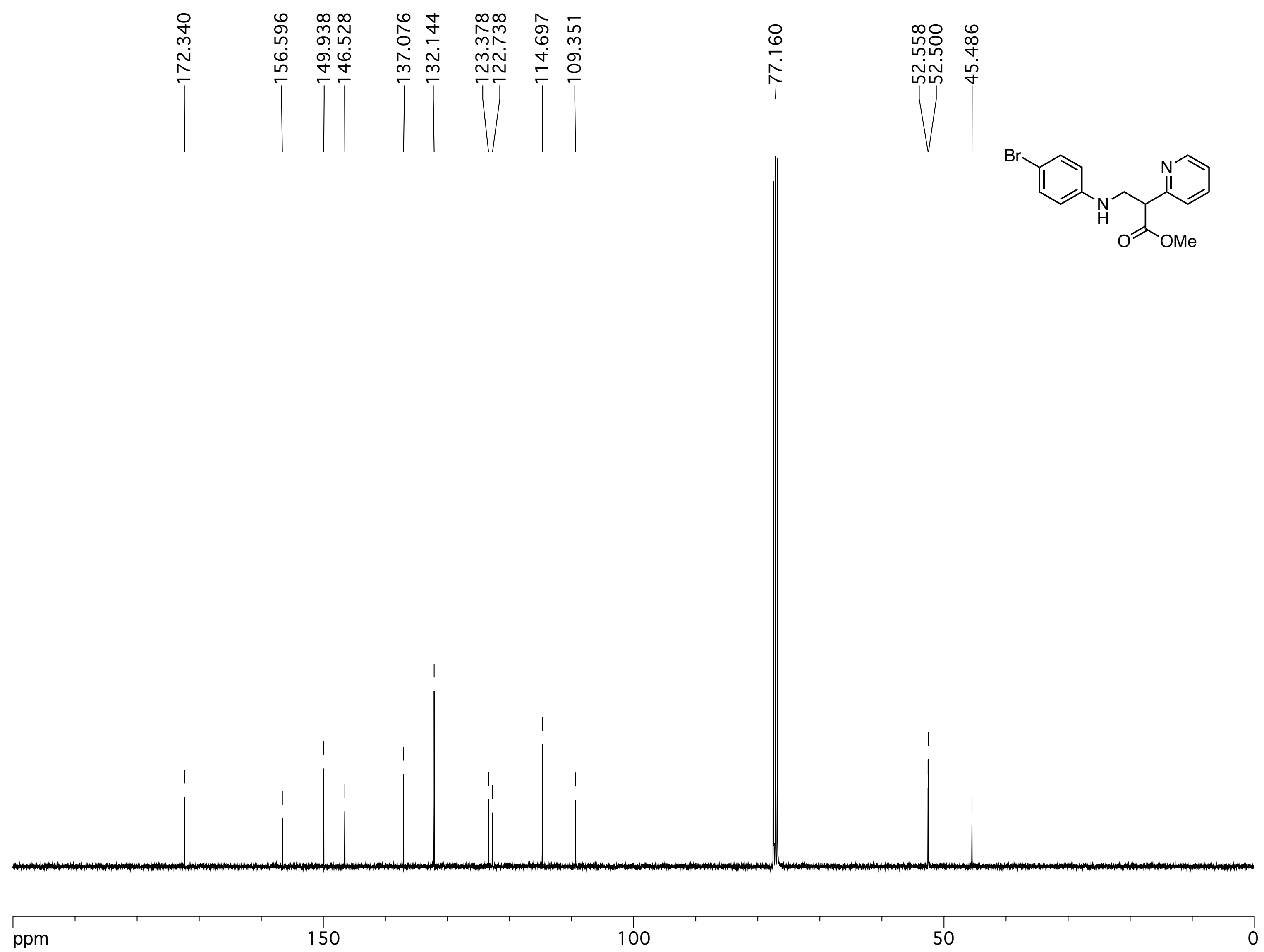

Figure S33. ^{13}C NMR spectrum of **3n** in $CDCl_3$ (100 MHz) measured at 23 °C.

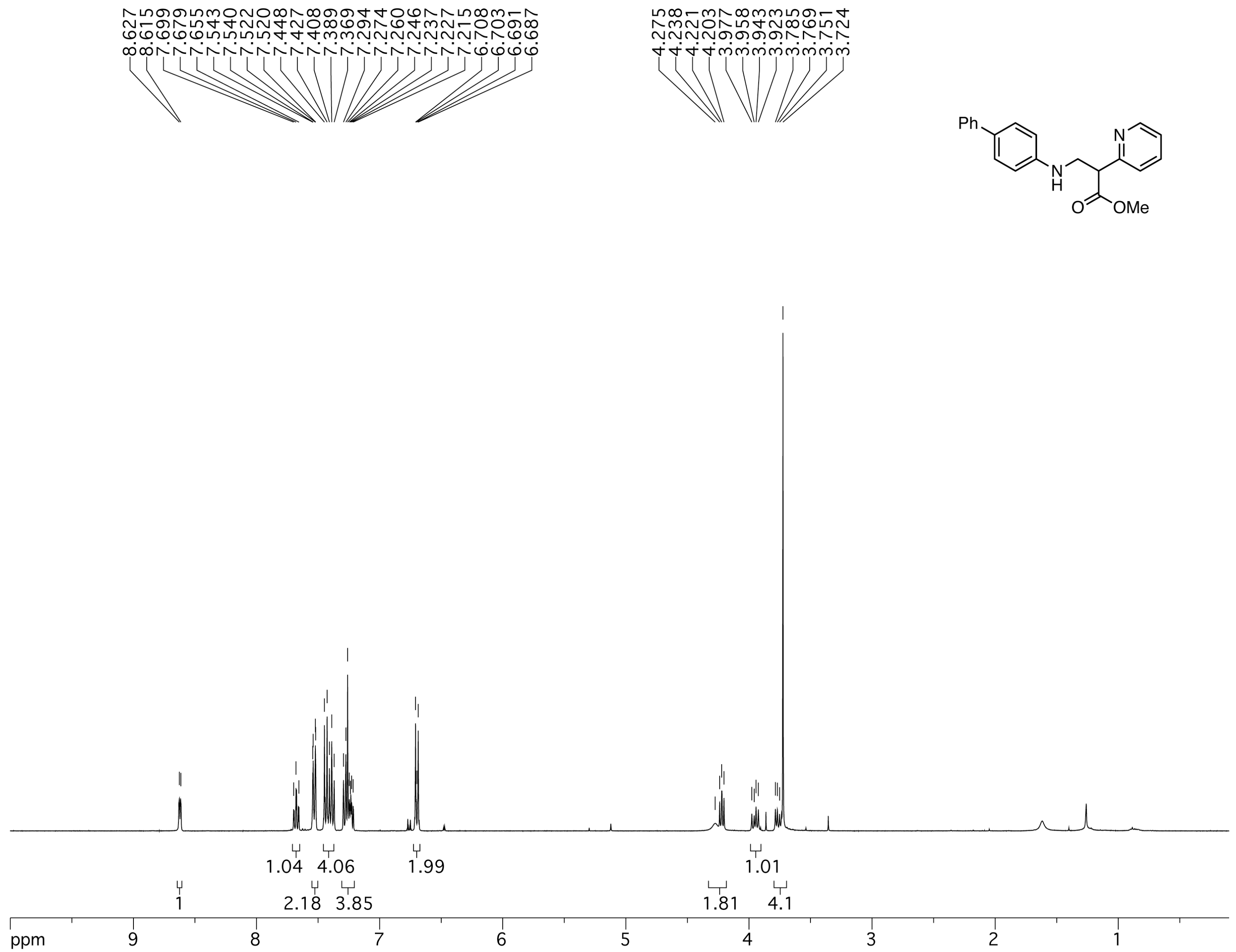

Figure S34. ^{1}H NMR spectrum of **3o** in $CDCl_3$ (400 MHz) measured at 23 °C.

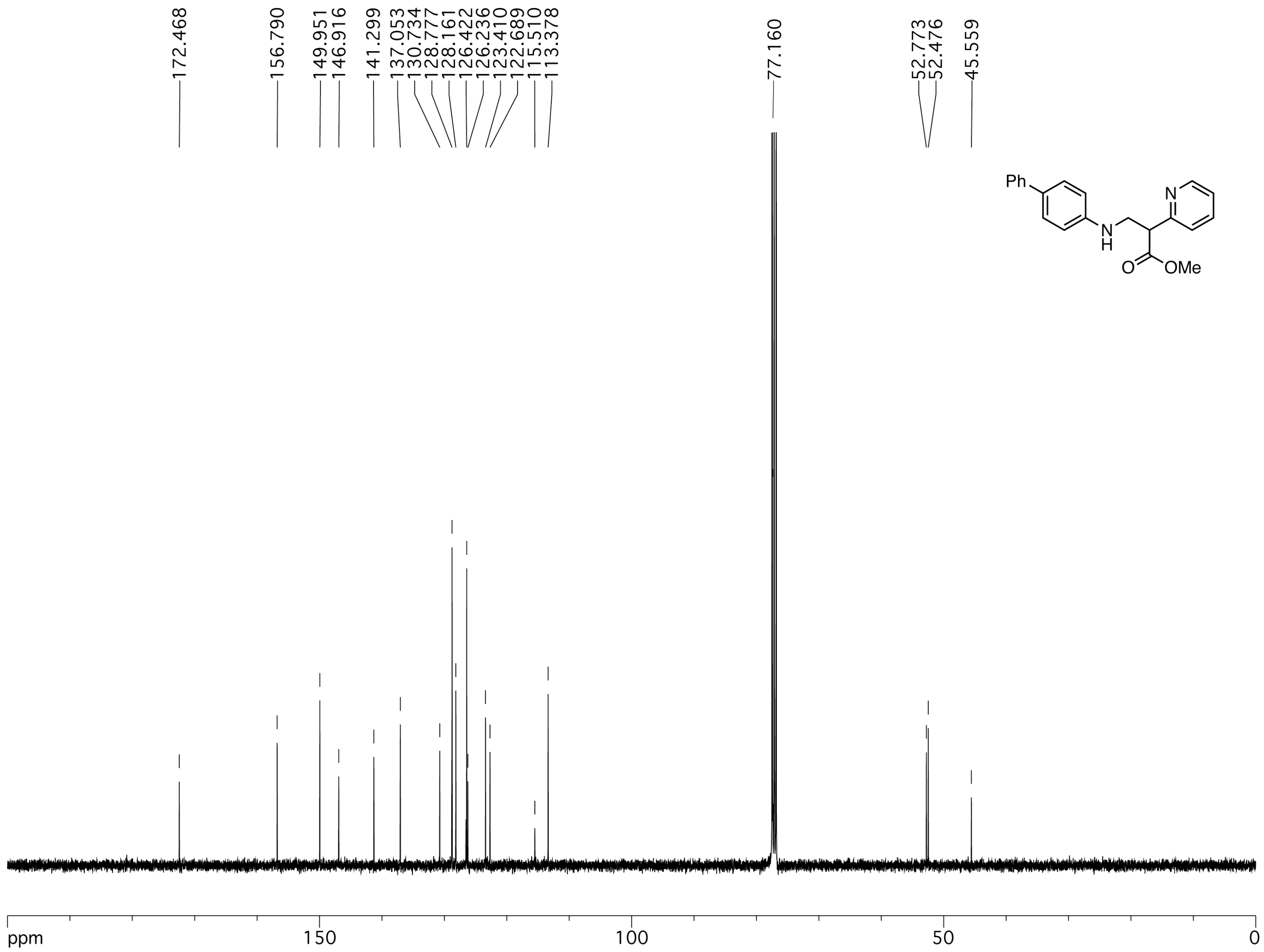

Figure S35. ^{13}C NMR spectrum of **3o** in $CDCl_3$ (100 MHz) measured at 23 °C.

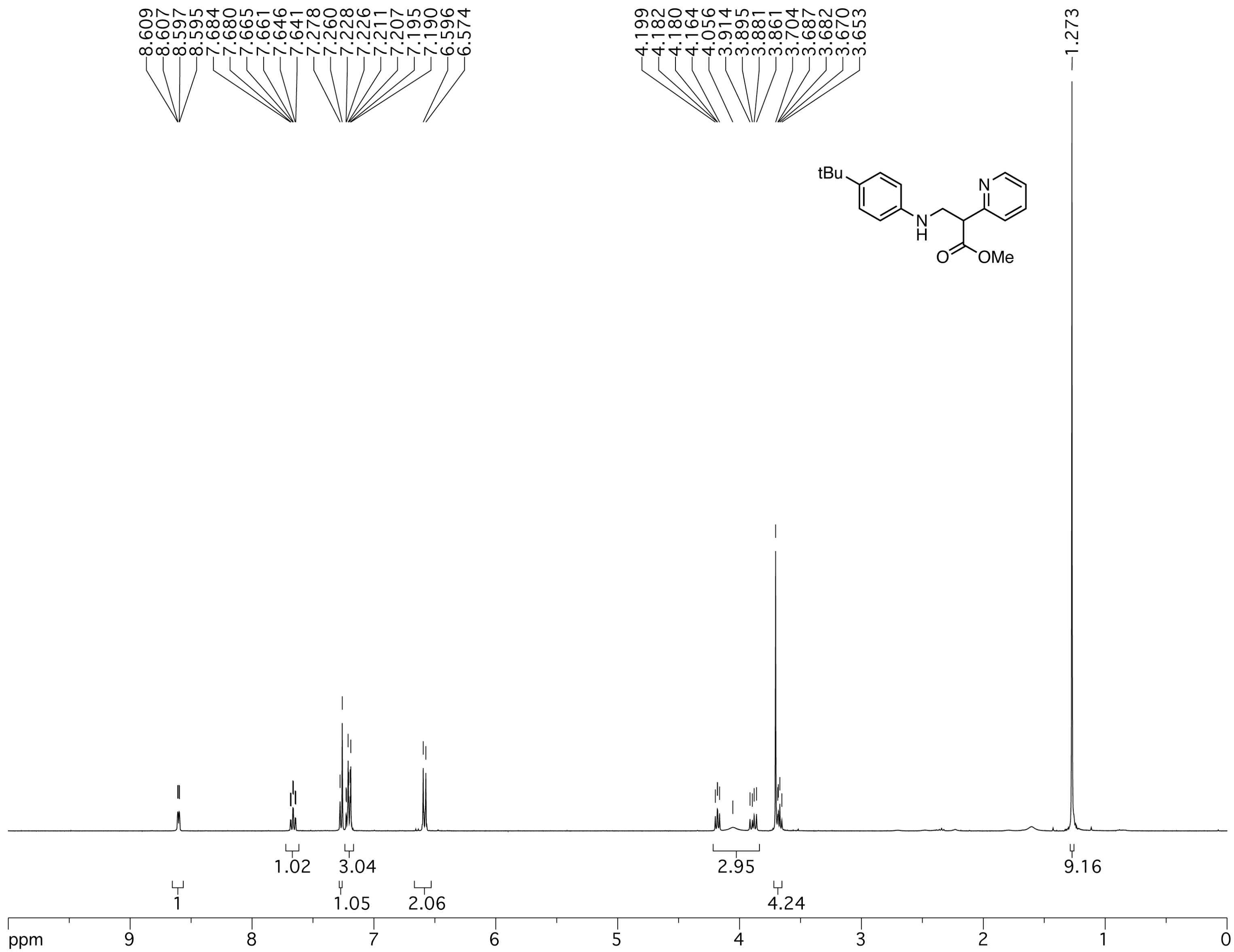

Figure S36. ^{1}H NMR spectrum of **3p** in $CDCl_3$ (400 MHz) measured at 23 °C.

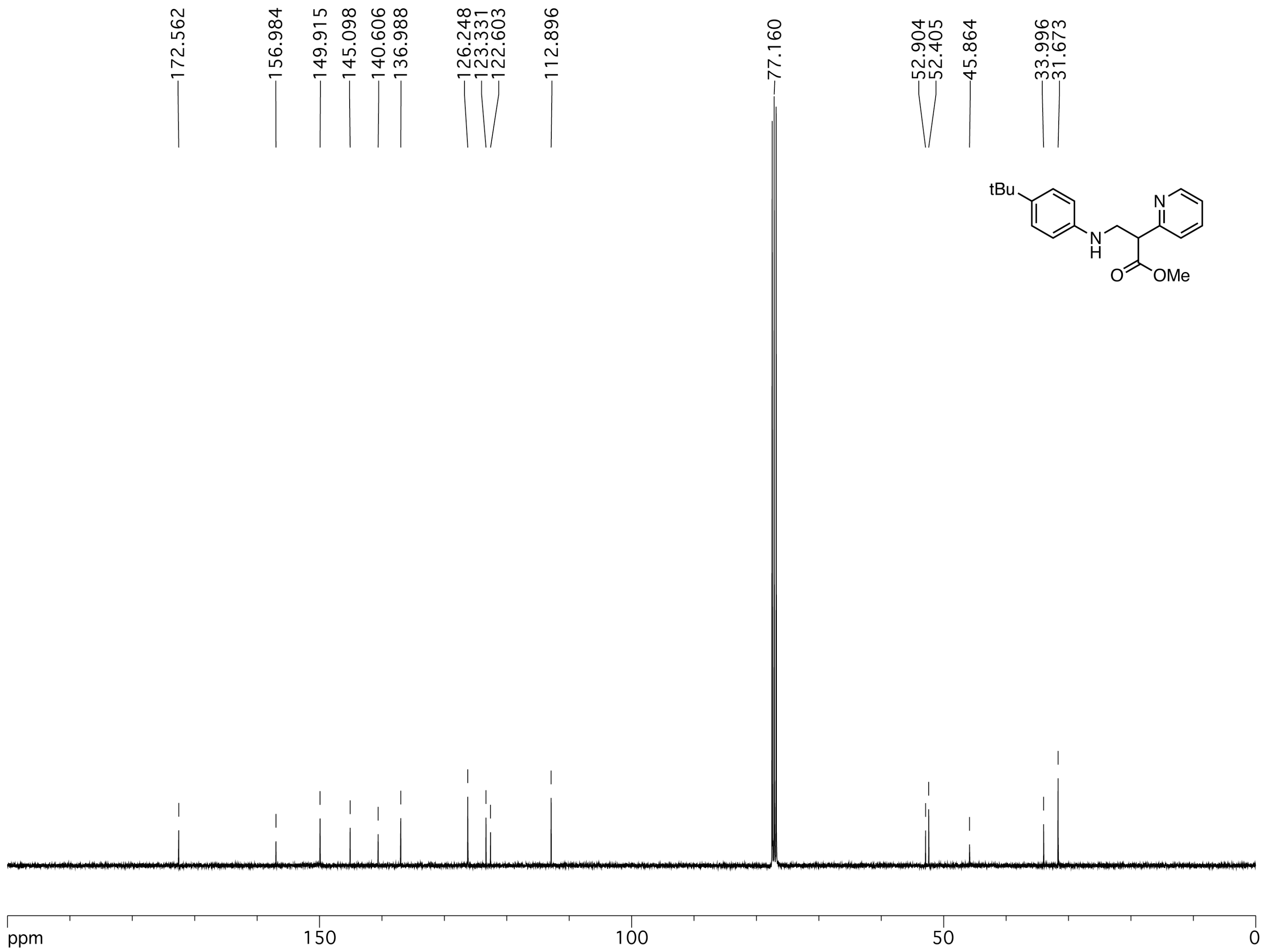

Figure S37. ^{13}C NMR spectrum of **3p** in $CDCl_3$ (100 MHz) measured at 23 °C.

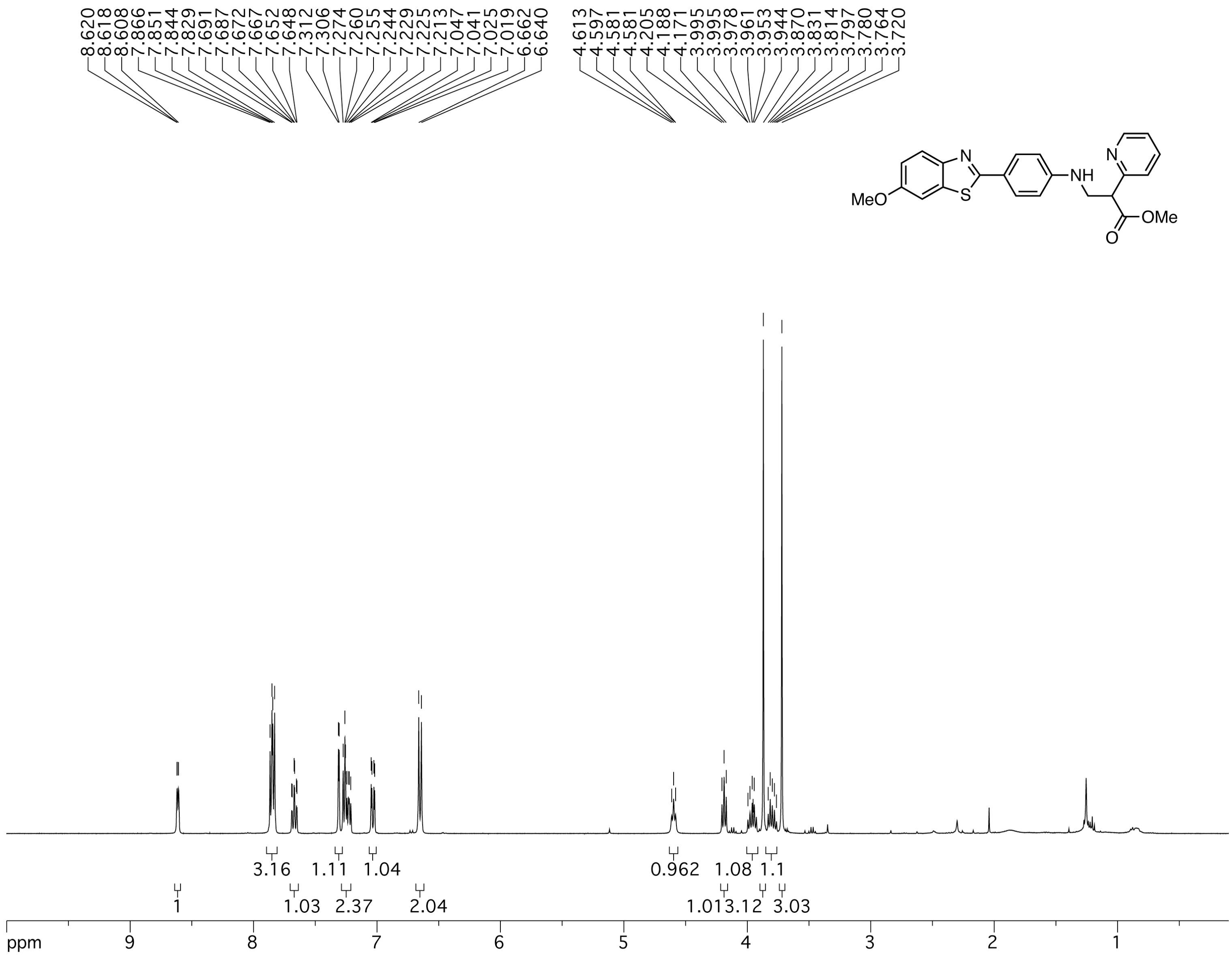

Figure S38. ^{1}H NMR spectrum of **3q** in $CDCl_3$ (400 MHz) measured at 23 °C.

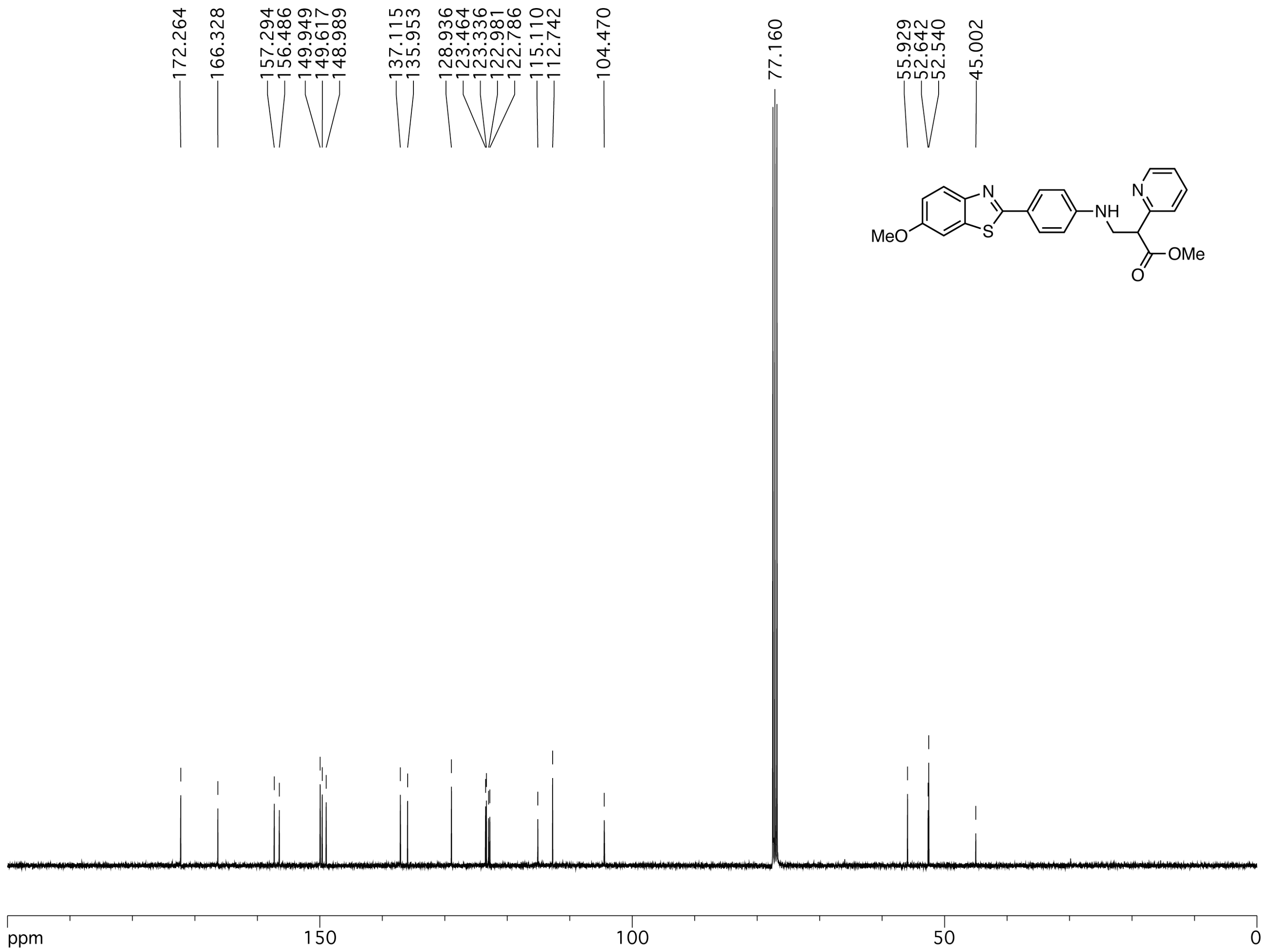

Figure S39. ^{13}C NMR spectrum of **3q** in $CDCl_3$ (100 MHz) measured at 23 °C.

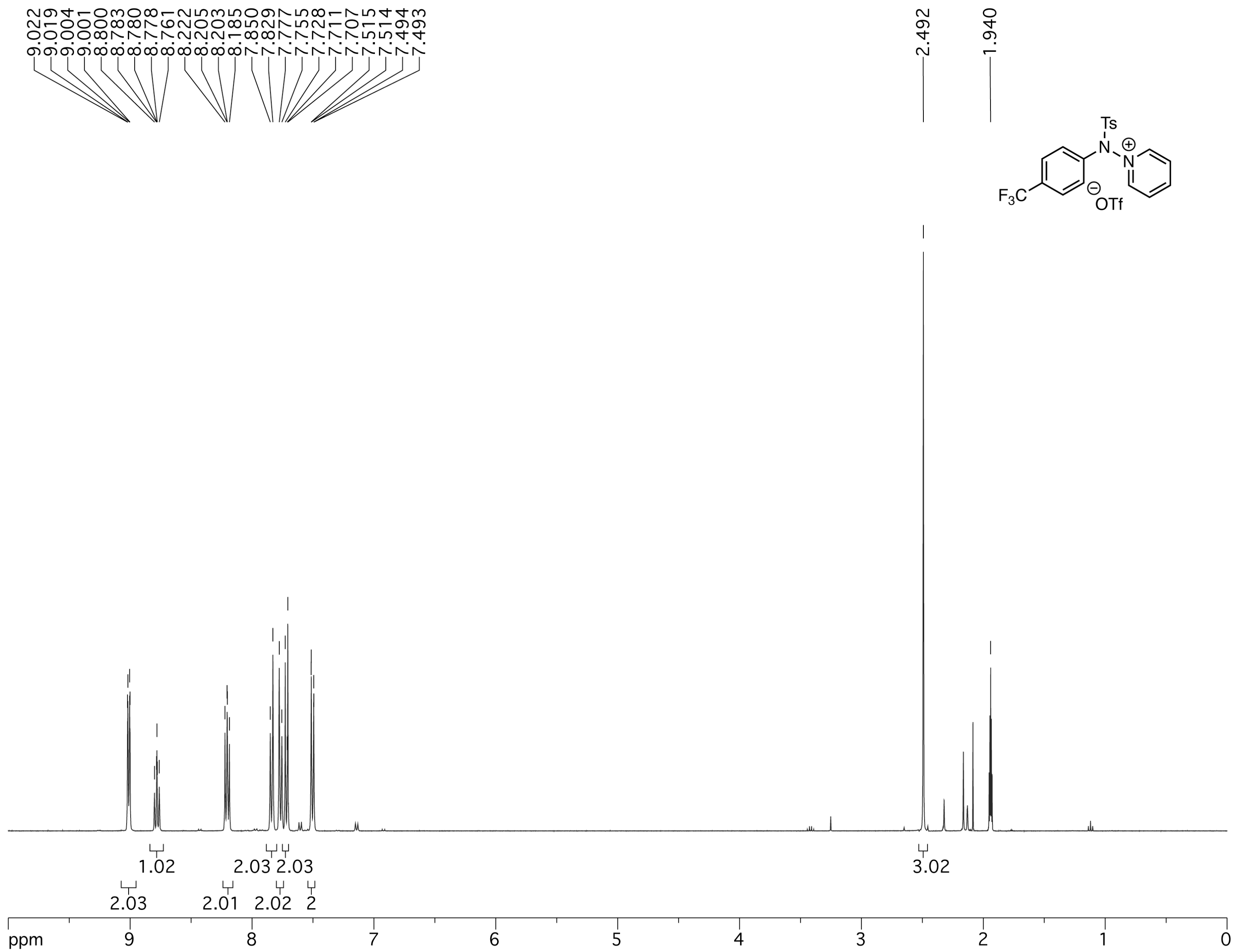

Figure S40. ^{1}H NMR spectrum of **4a** in CD_3CN (400 MHz) measured at 23 °C.

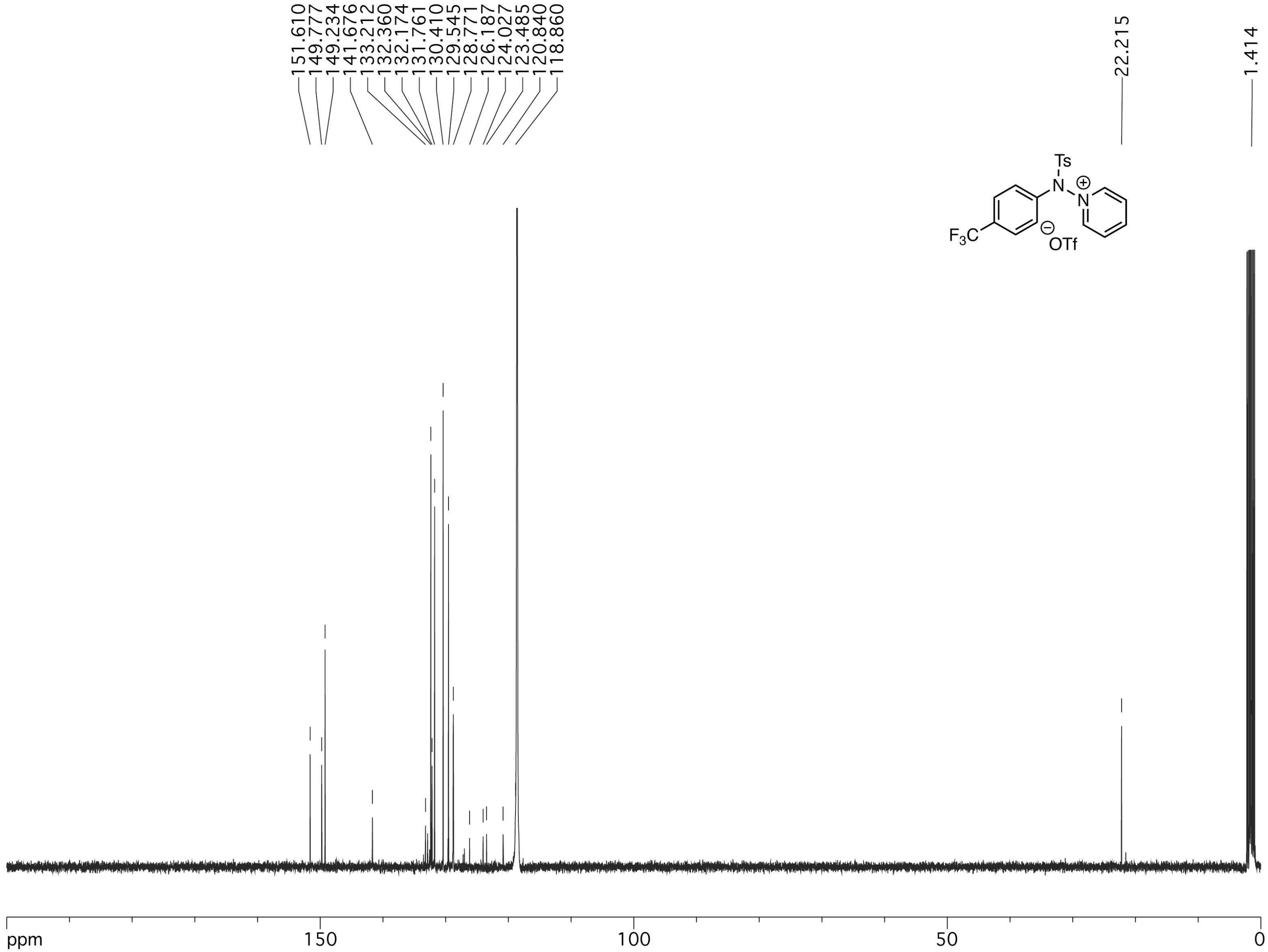

Figure S41. ^{13}C NMR spectrum of **4a** in CD_3CN (100 MHz) measured at 23 °C.

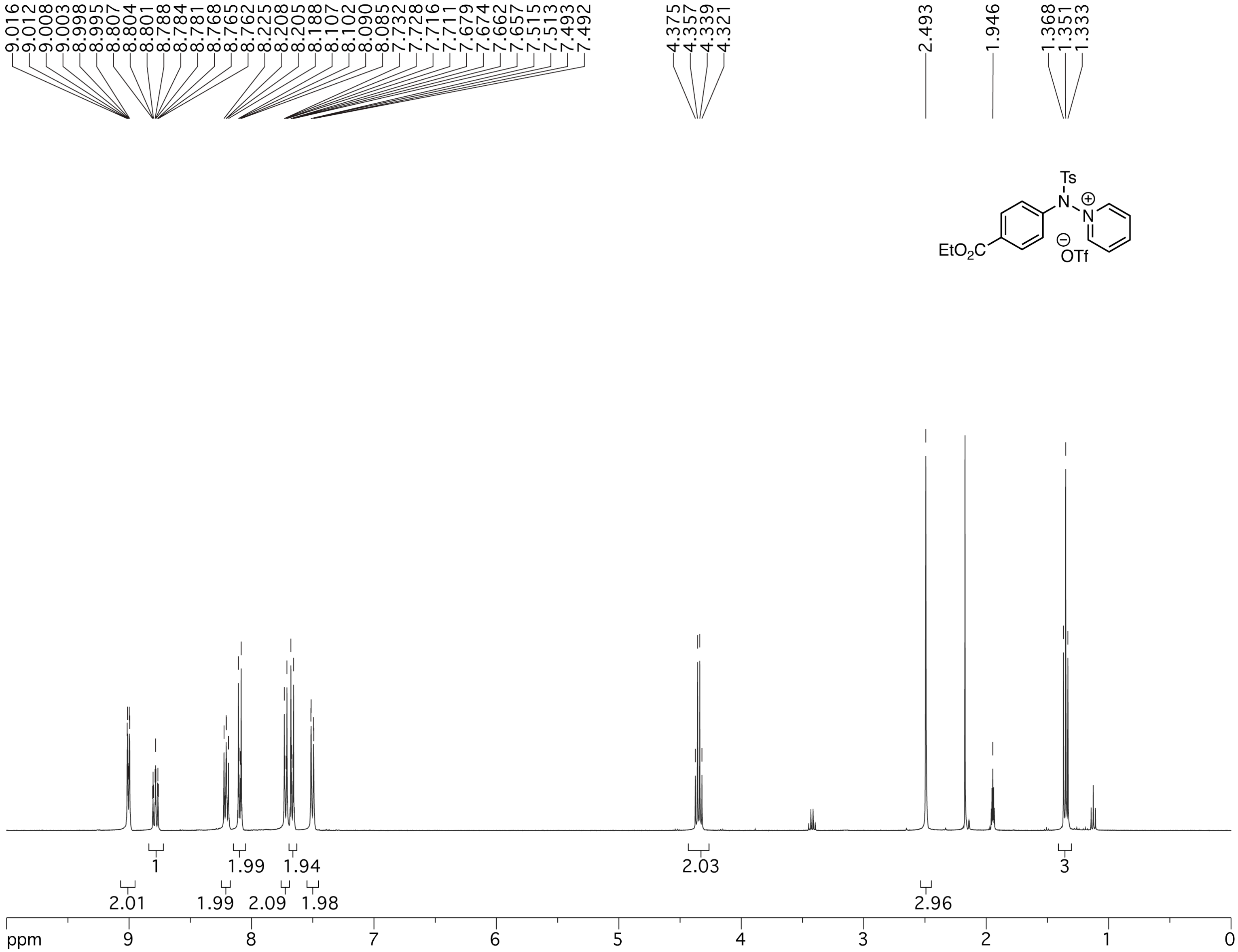

Figure S42. ^{1}H NMR spectrum of **4n** in CD_3CN (400 MHz) measured at 23 °C.

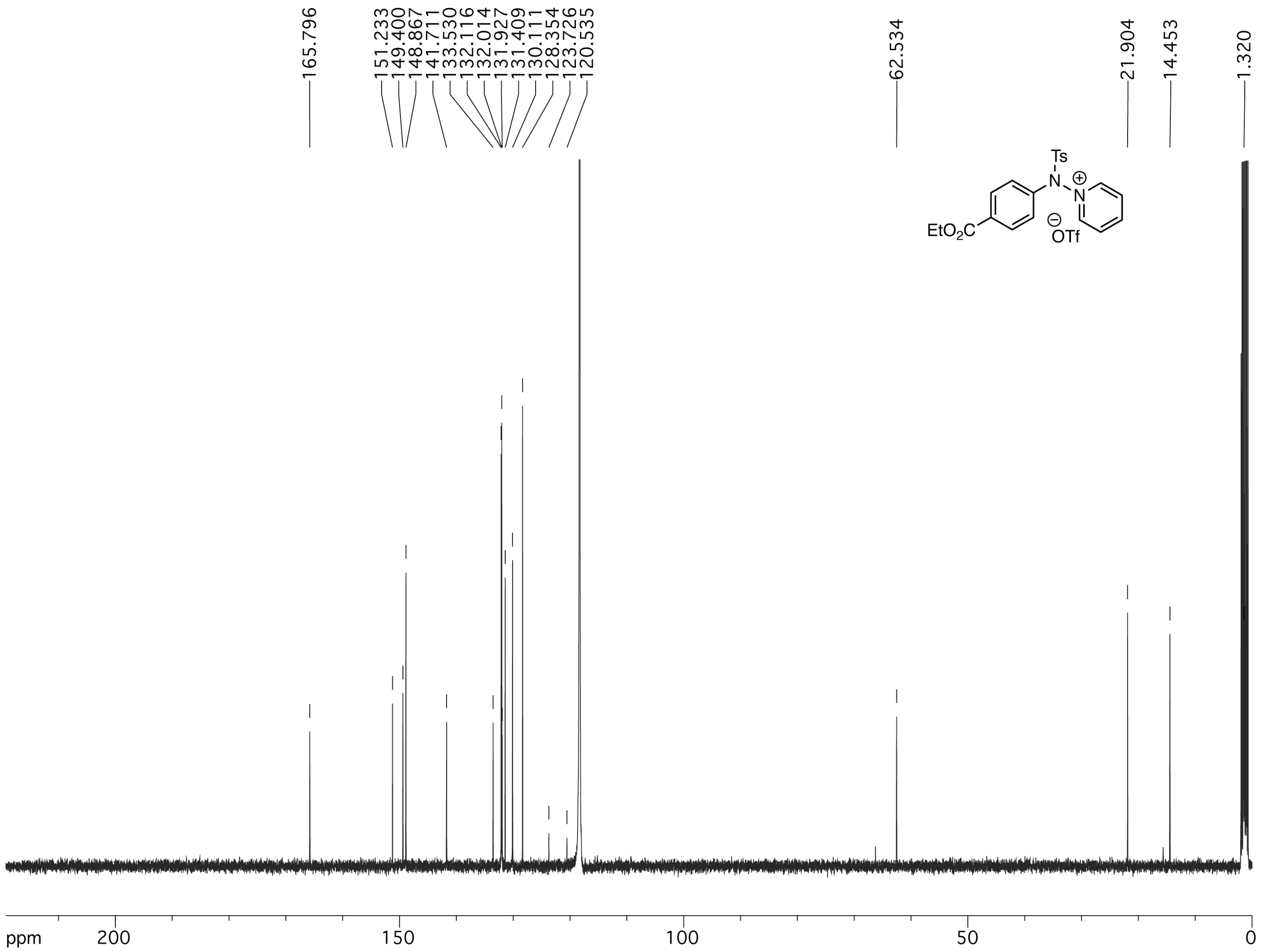

Figure S43. ^{13}C NMR spectrum of **4n** in CD_3CN (100 MHz) measured at 23 °C.

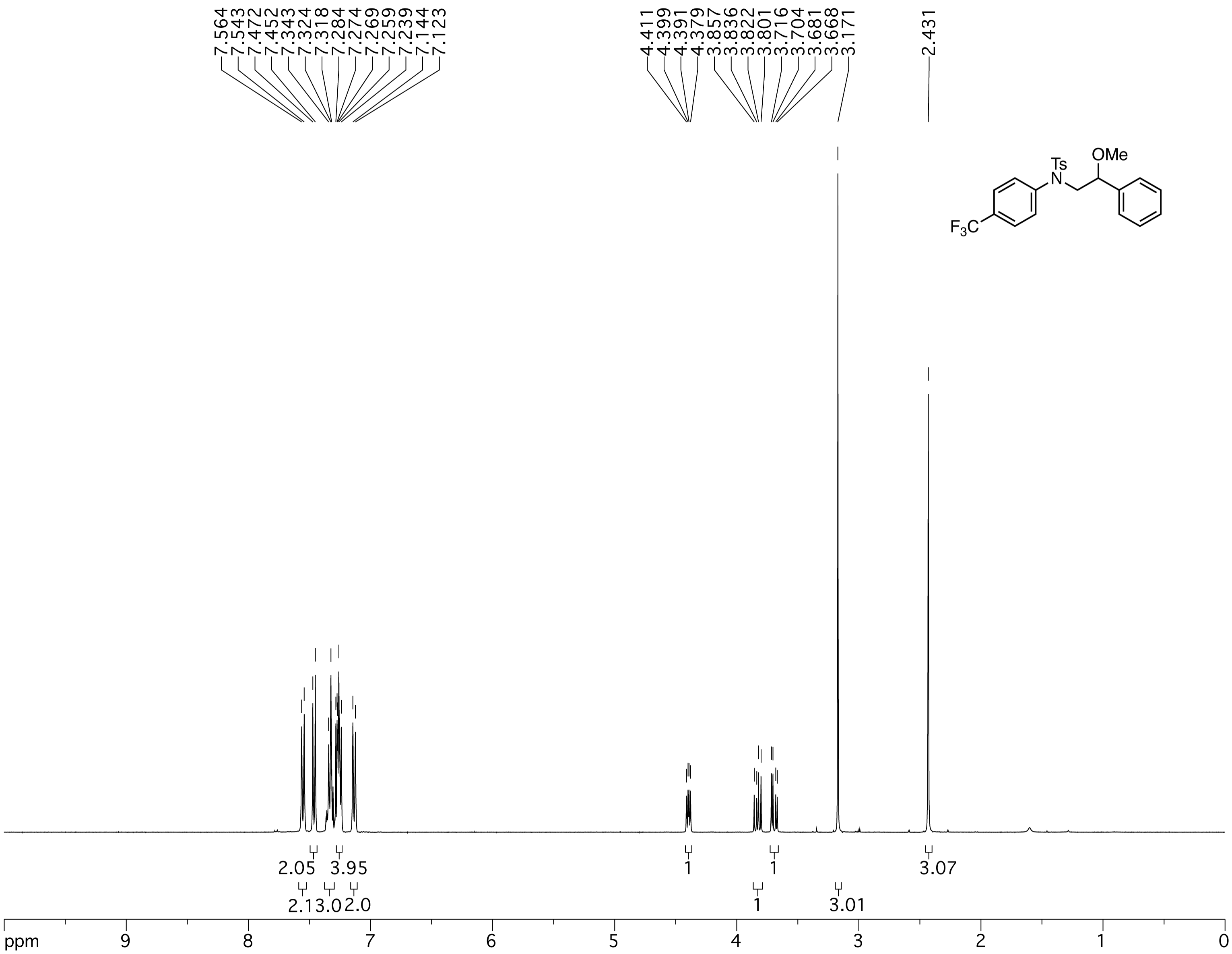

Figure S44. ^{1}H NMR spectrum of **6a** in $CDCl_3$ (400 MHz) measured at 23 °C.

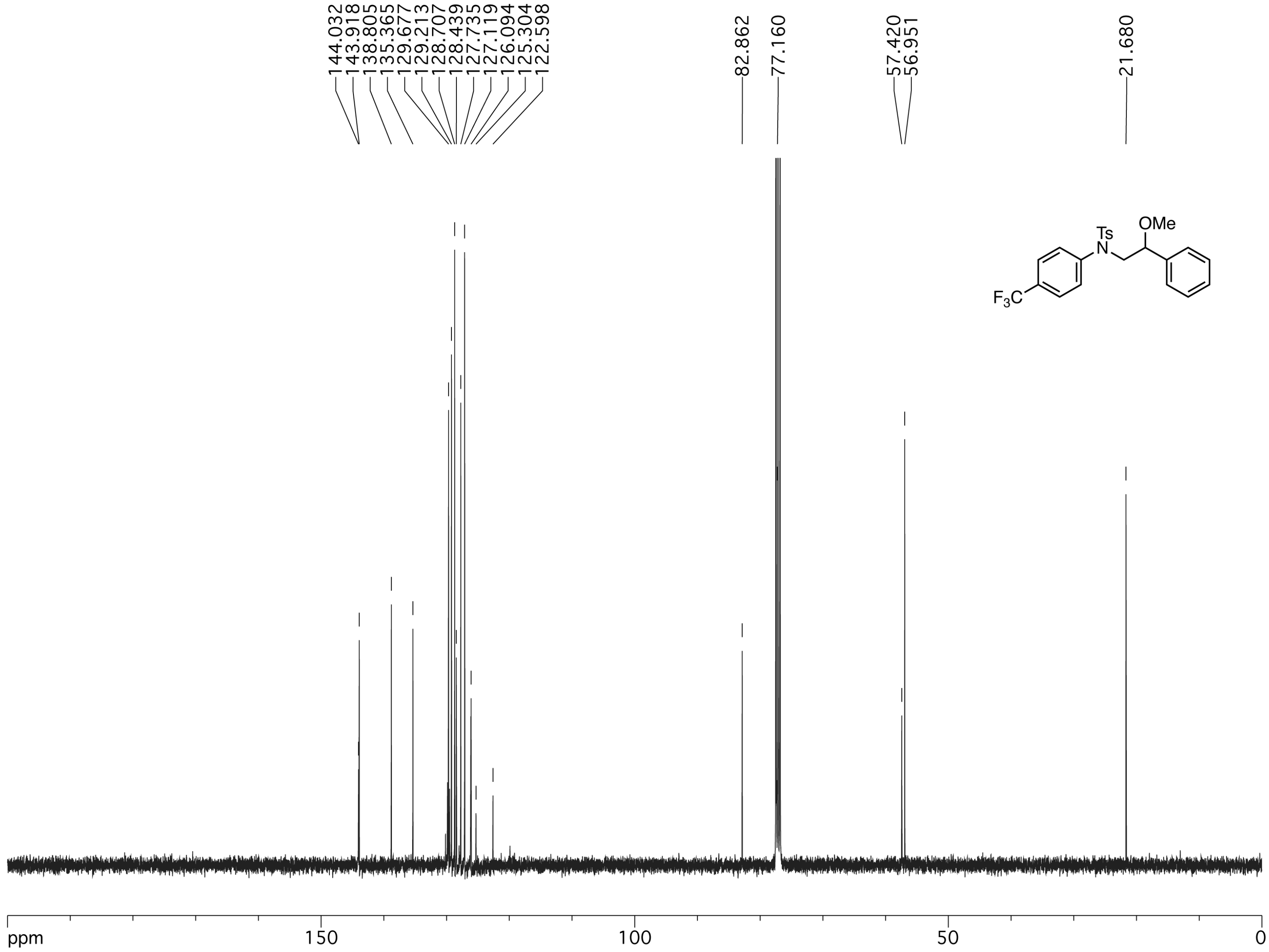

Figure S45. ^{13}C NMR spectrum of **6a** in $CDCl_3$ (100 MHz) measured at 23 °C.

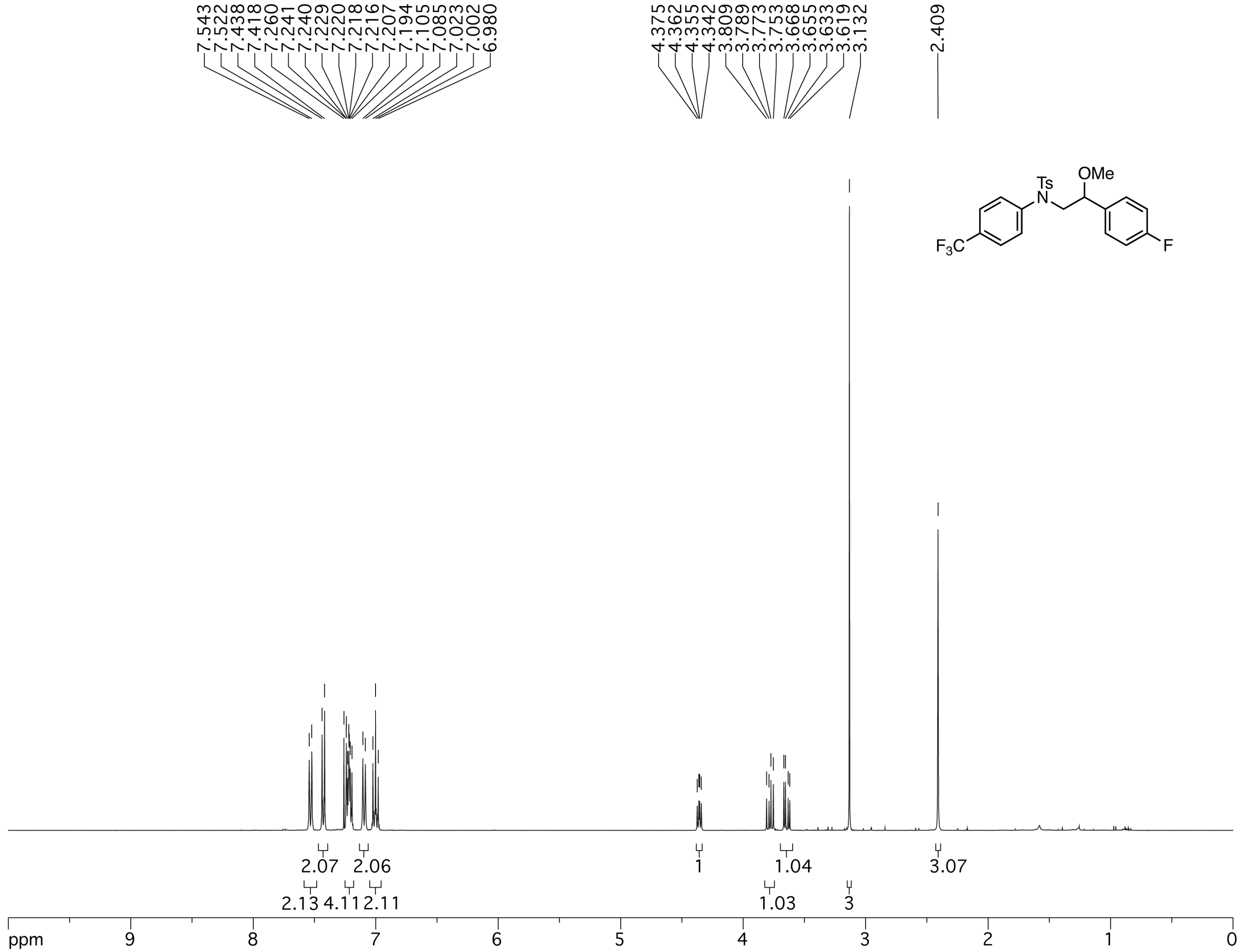

Figure S46. ^{1}H NMR spectrum of **6b** in $CDCl_3$ (400 MHz) measured at 23 °C.

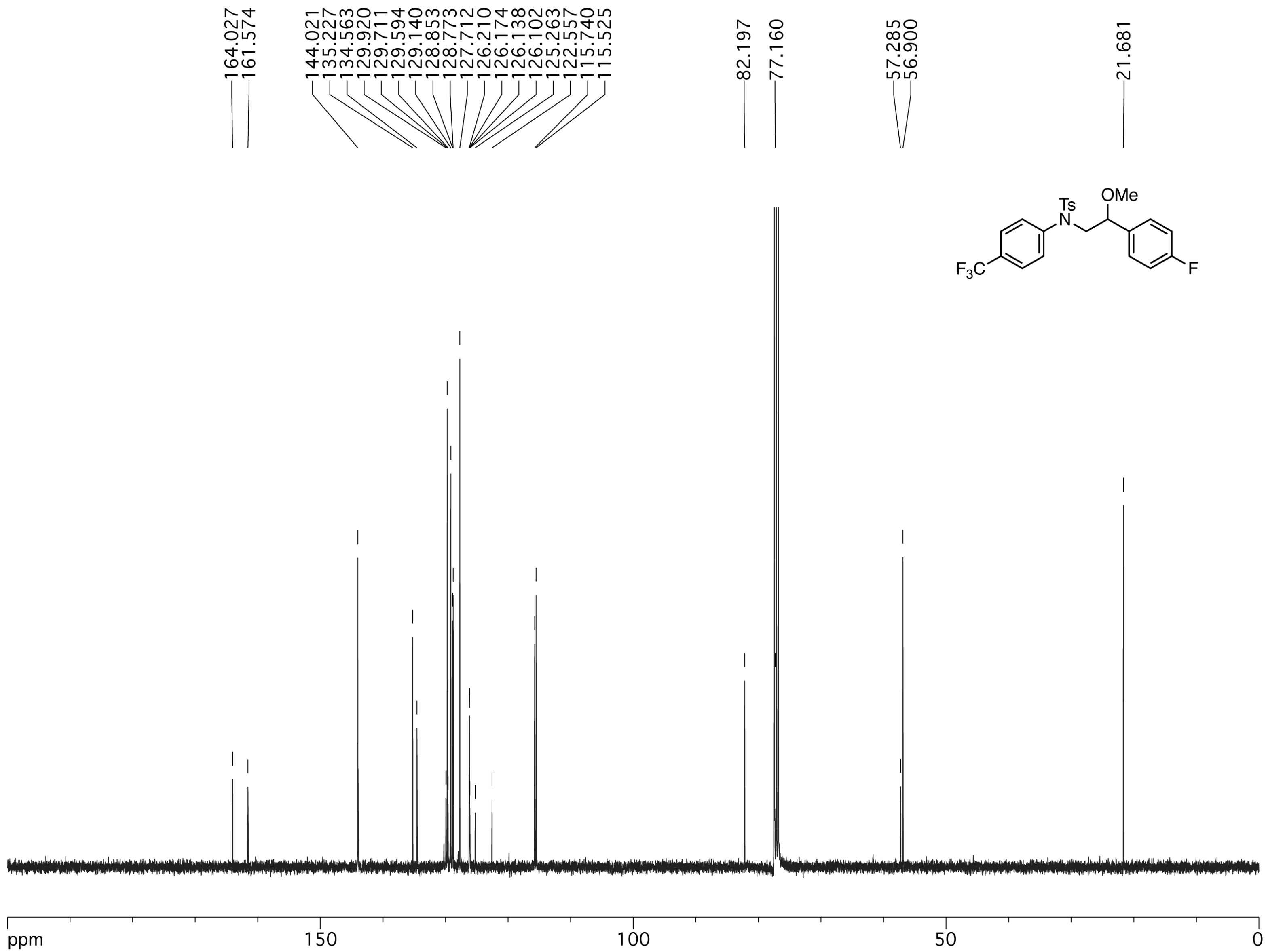

Figure S47. ^{13}C NMR spectrum of **6b** in $CDCl_3$ (100 MHz) measured at 23 °C.

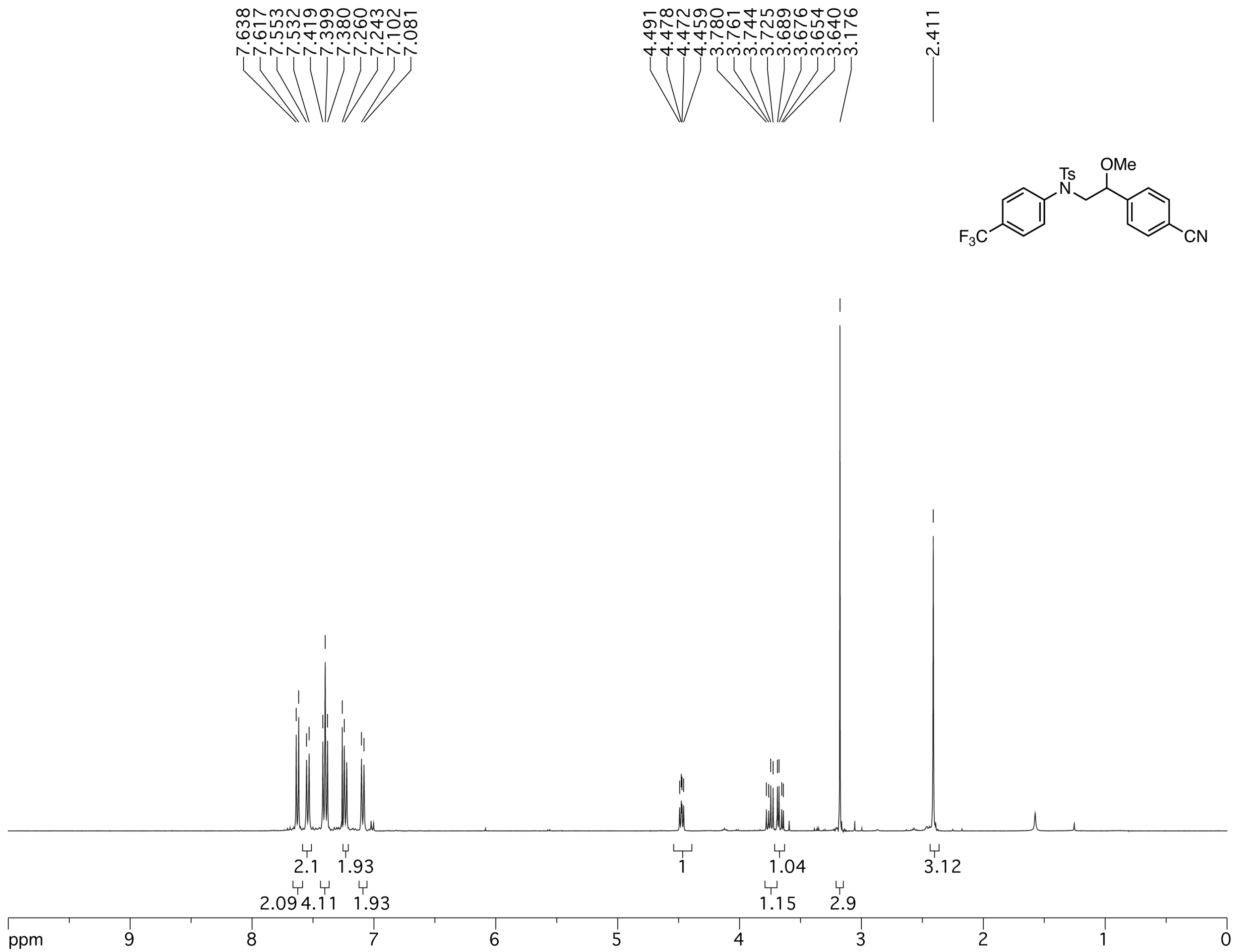

Figure S48. ^{1}H NMR spectrum of **6c** in $CDCl_3$ (400 MHz) measured at 23 °C.

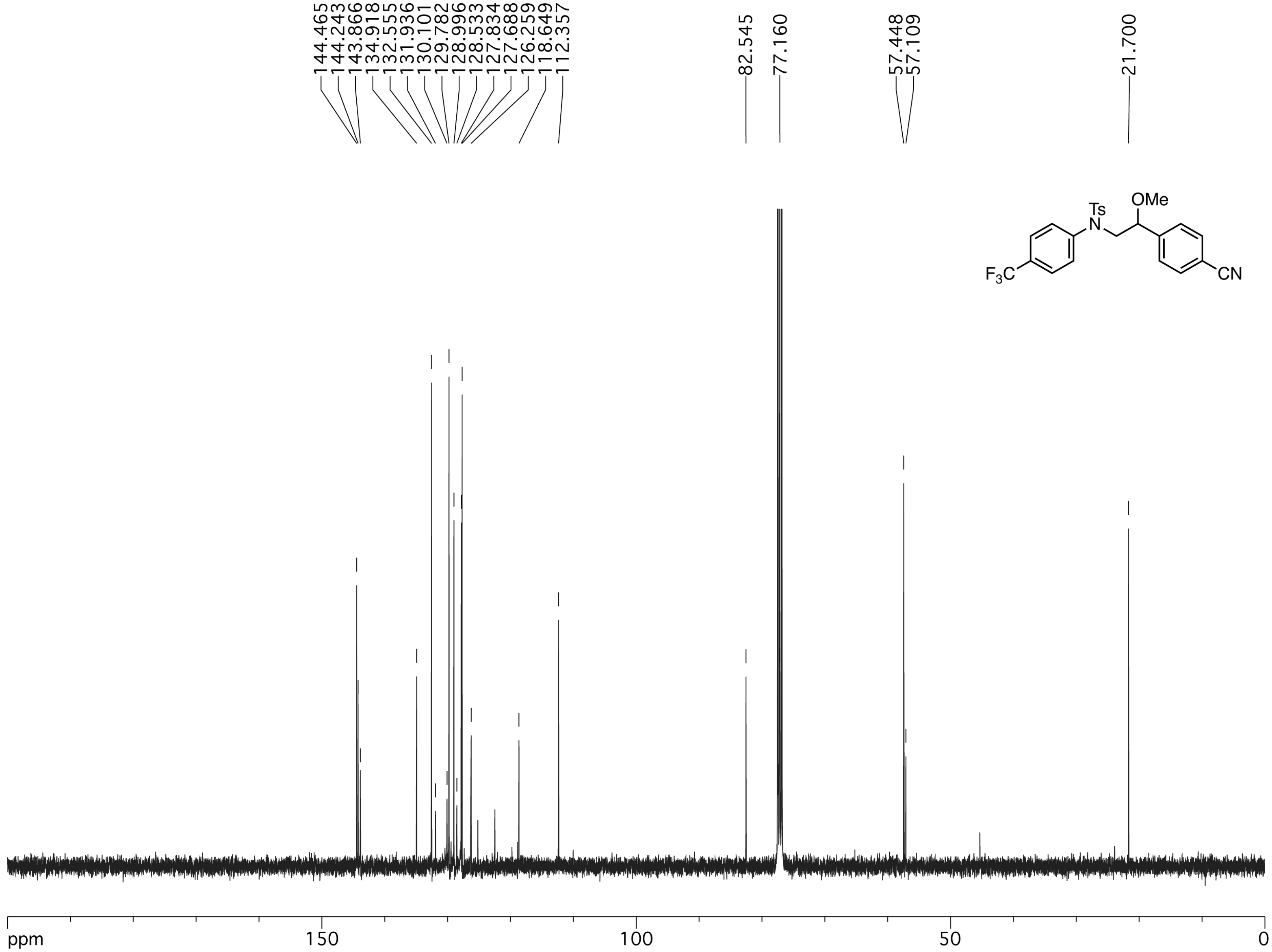

Figure S49. ^{13}C NMR spectrum of **6c** in $CDCl_3$ (100 MHz) measured at 23 °C.

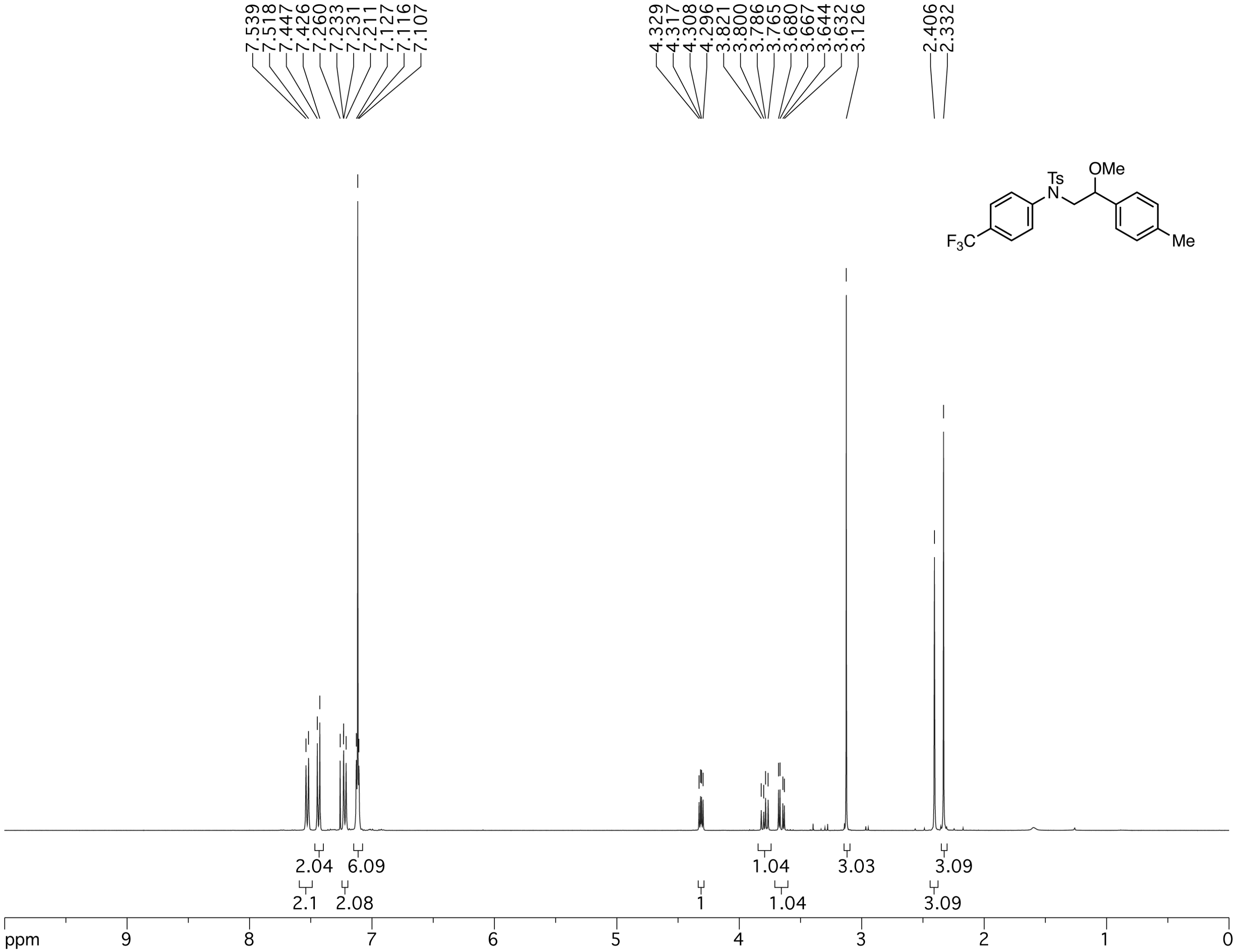

Figure S50. ^{1}H NMR spectrum of **6d** in $CDCl_3$ (400 MHz) measured at 23 °C.

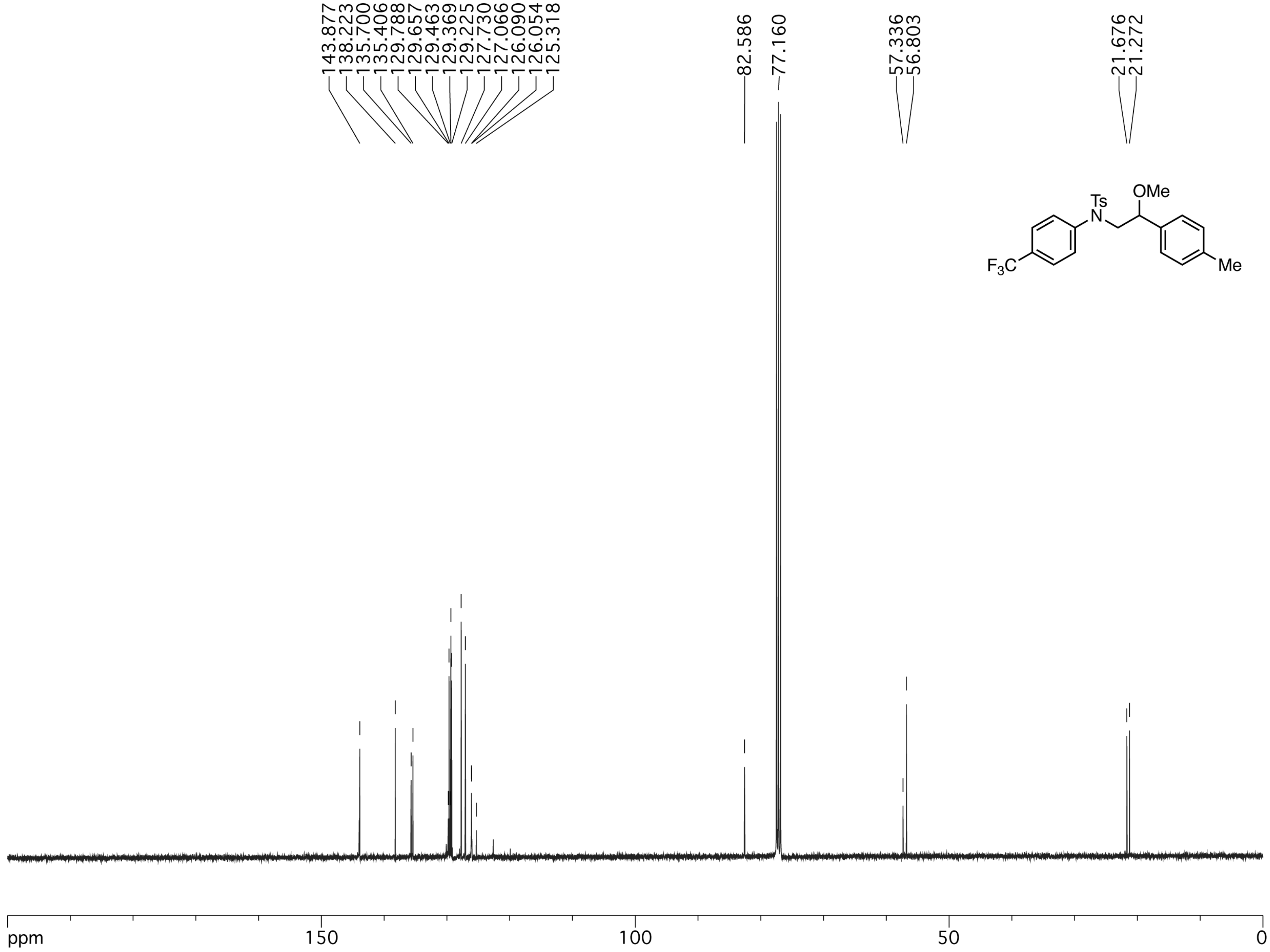

Figure S51. ^{13}C NMR spectrum of **6d** in $CDCl_3$ (100 MHz) measured at 23 °C.

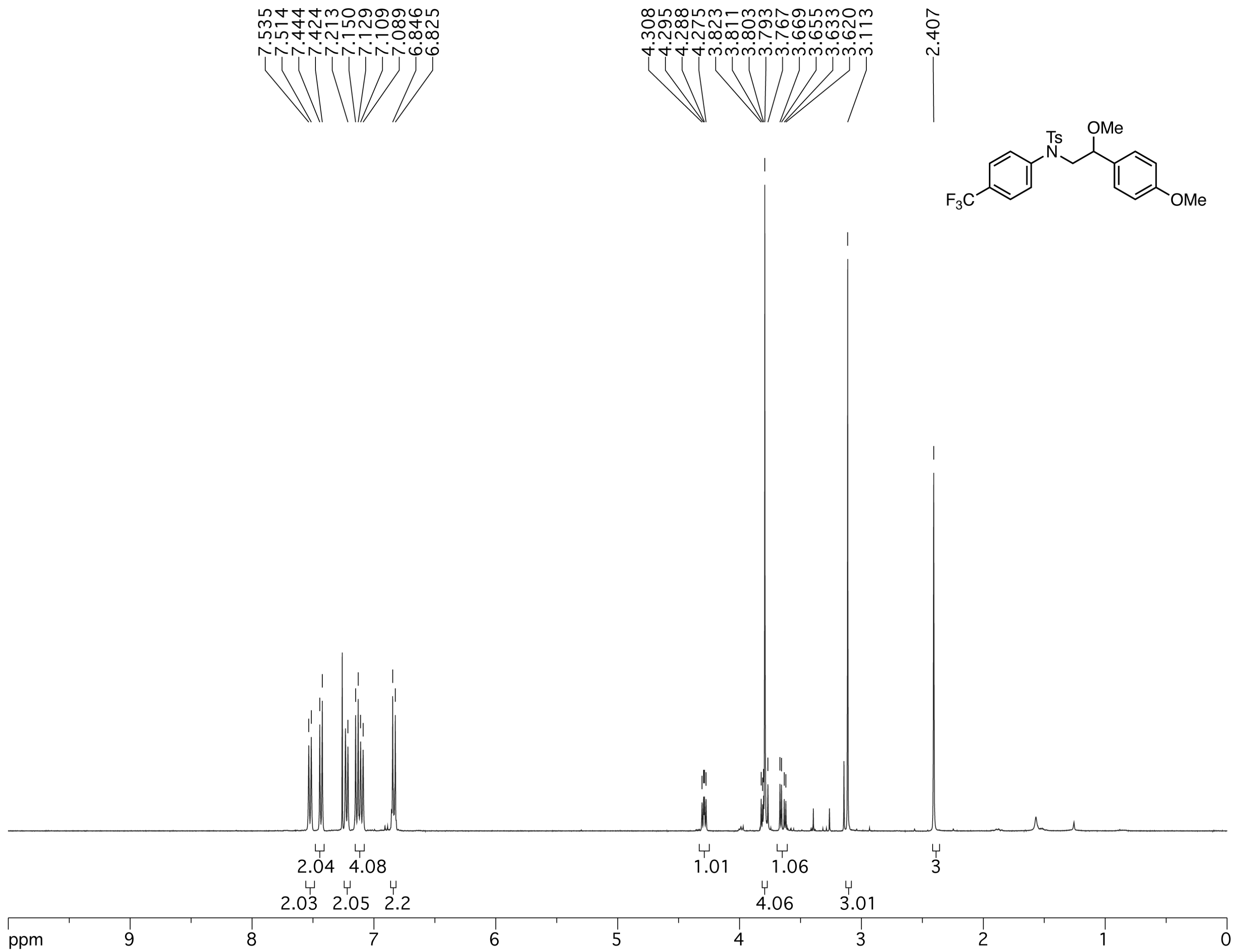

Figure S52. ^{1}H NMR spectrum of **6e** in $CDCl_3$ (400 MHz) measured at 23 °C.

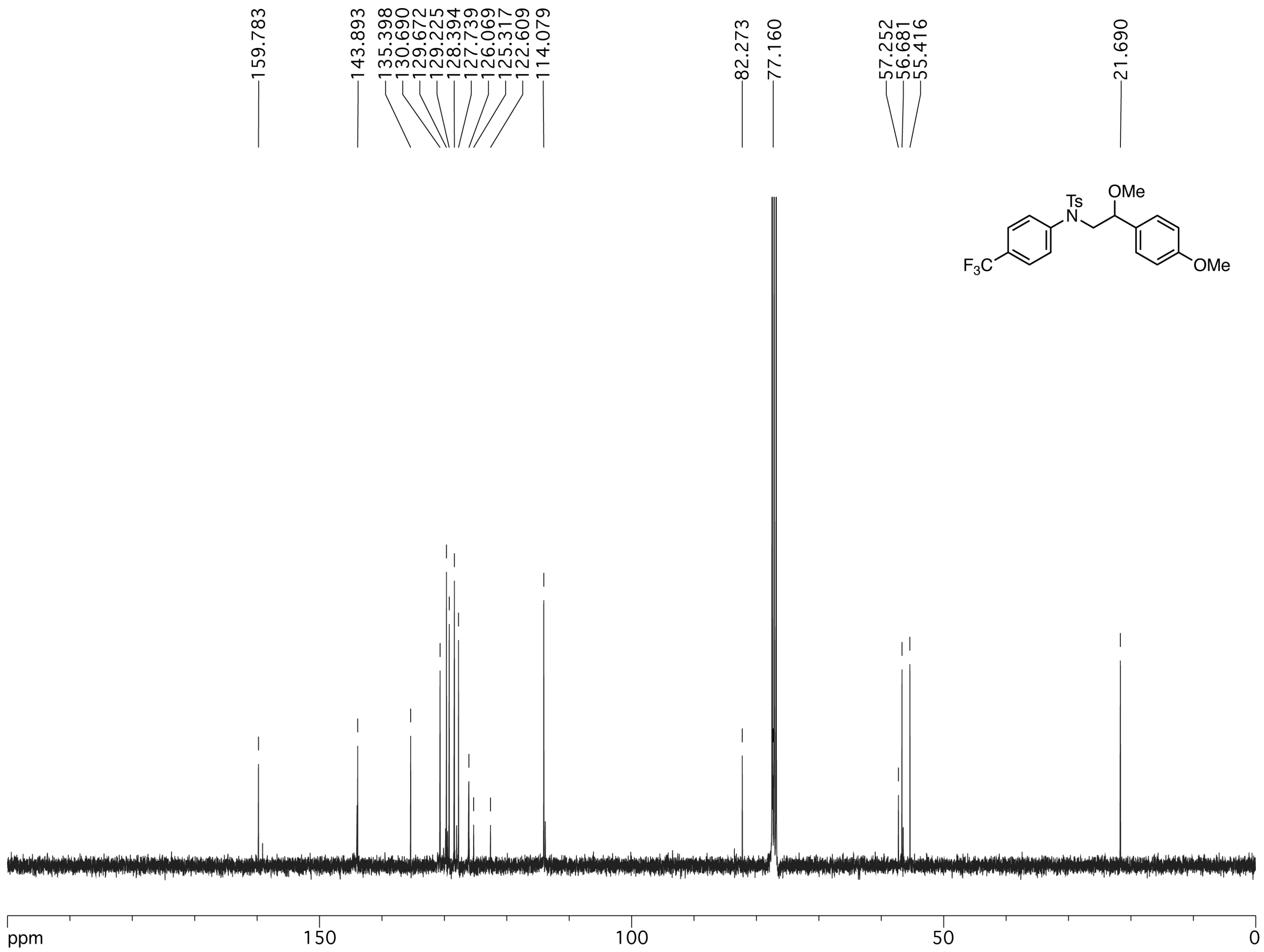

Figure S53. ^{13}C NMR spectrum of **6e** in $CDCl_3$ (100 MHz) measured at 23 °C.

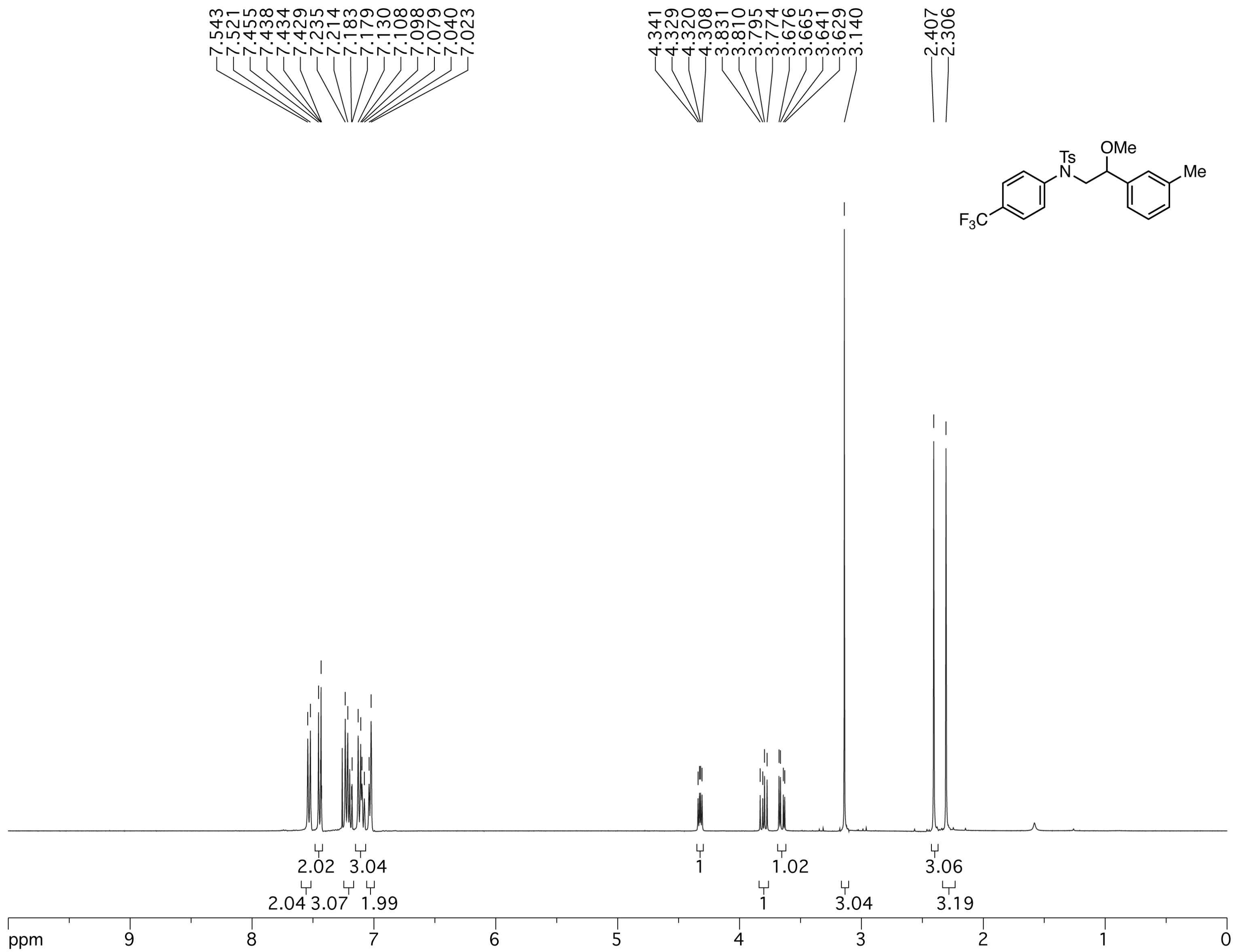

Figure S54. ^{1}H NMR spectrum of **6f** in $CDCl_3$ (400 MHz) measured at 23 °C.

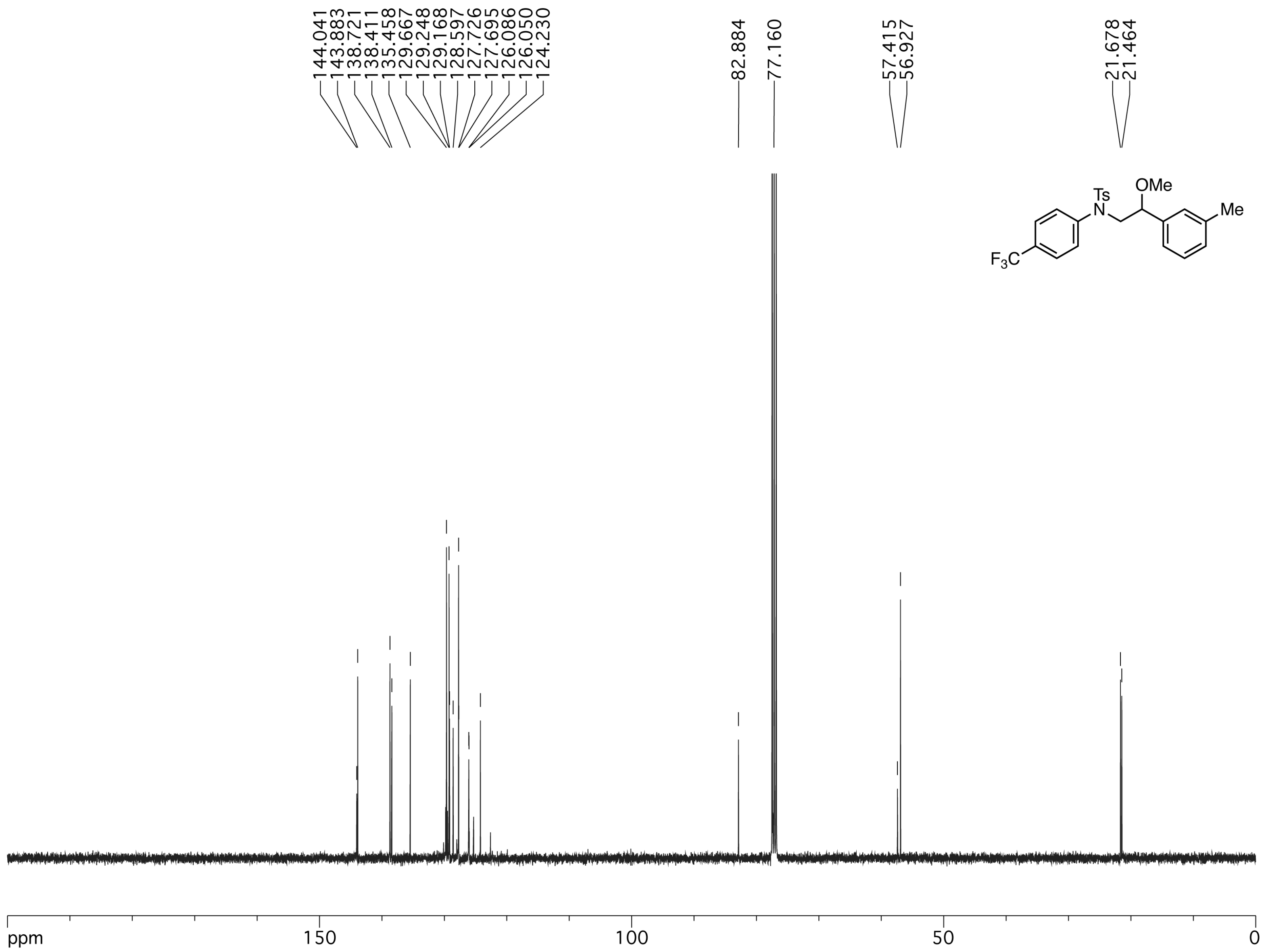

Figure S55. ^{13}C NMR spectrum of **6f** in $CDCl_3$ (100 MHz) measured at 23 °C.

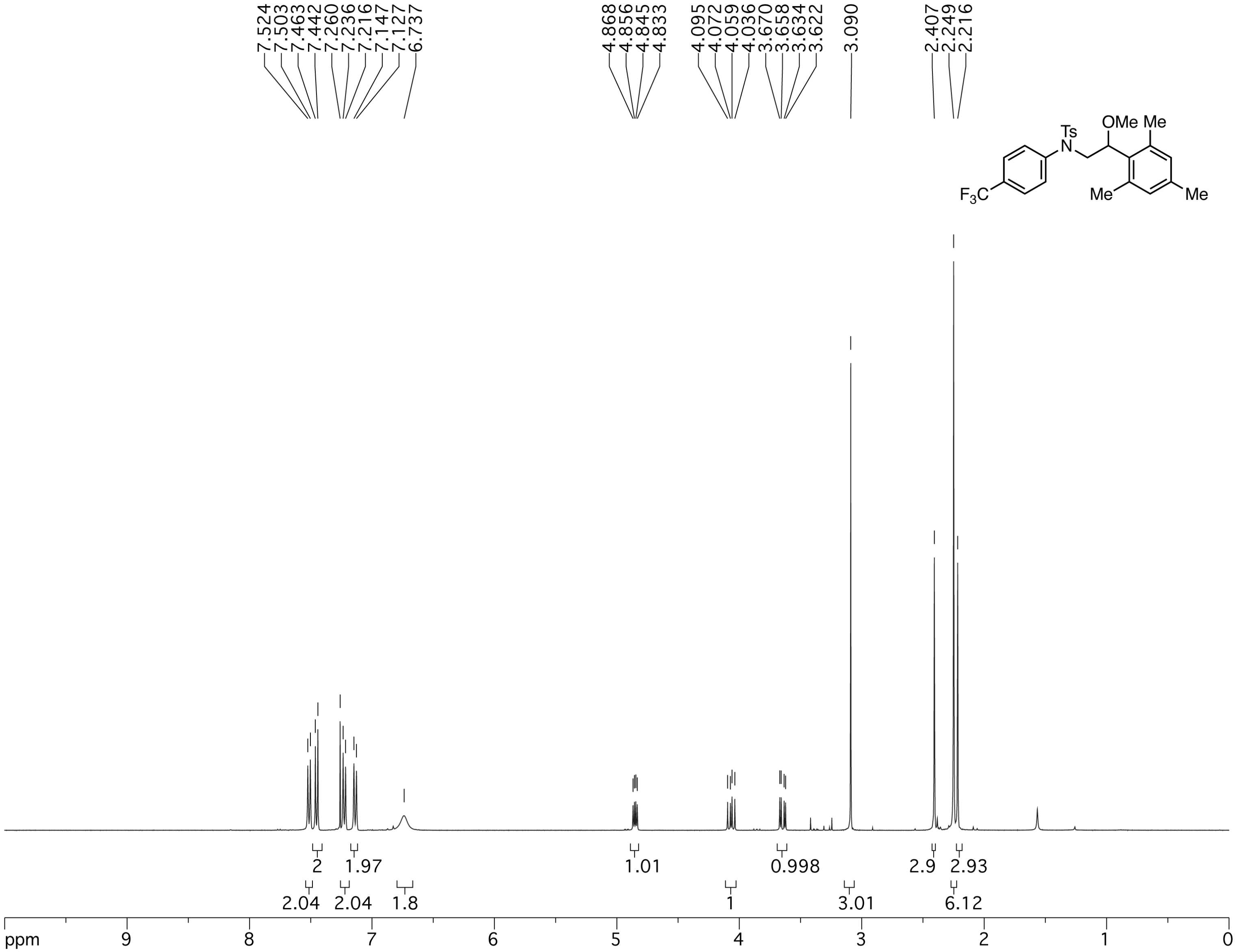

Figure S56. ^{1}H NMR spectrum of **6g** in $CDCl_3$ (400 MHz) measured at 23 °C.

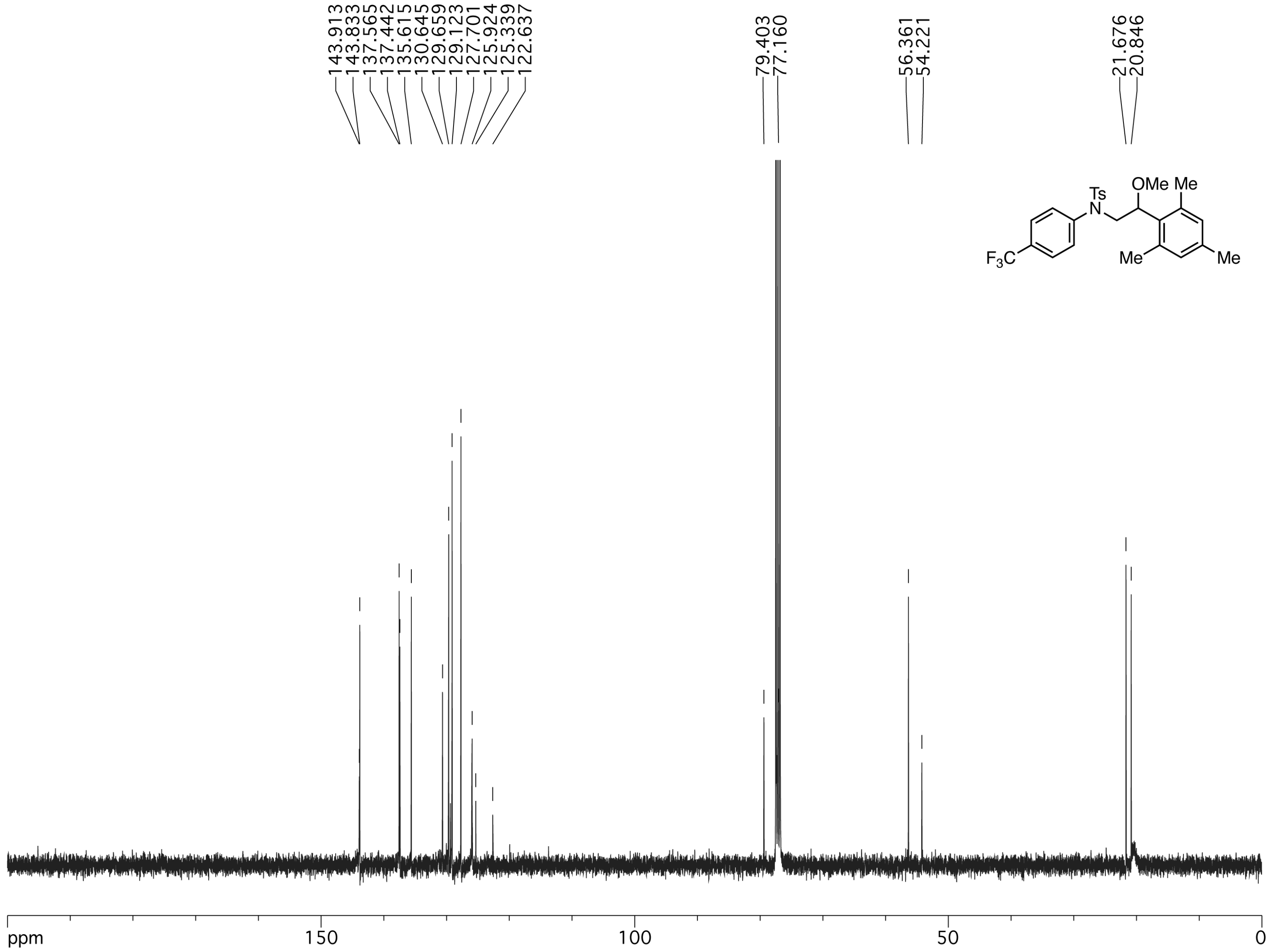

Figure S57. ^{13}C NMR spectrum of **6g** in $CDCl_3$ (100 MHz) measured at 23 °C.

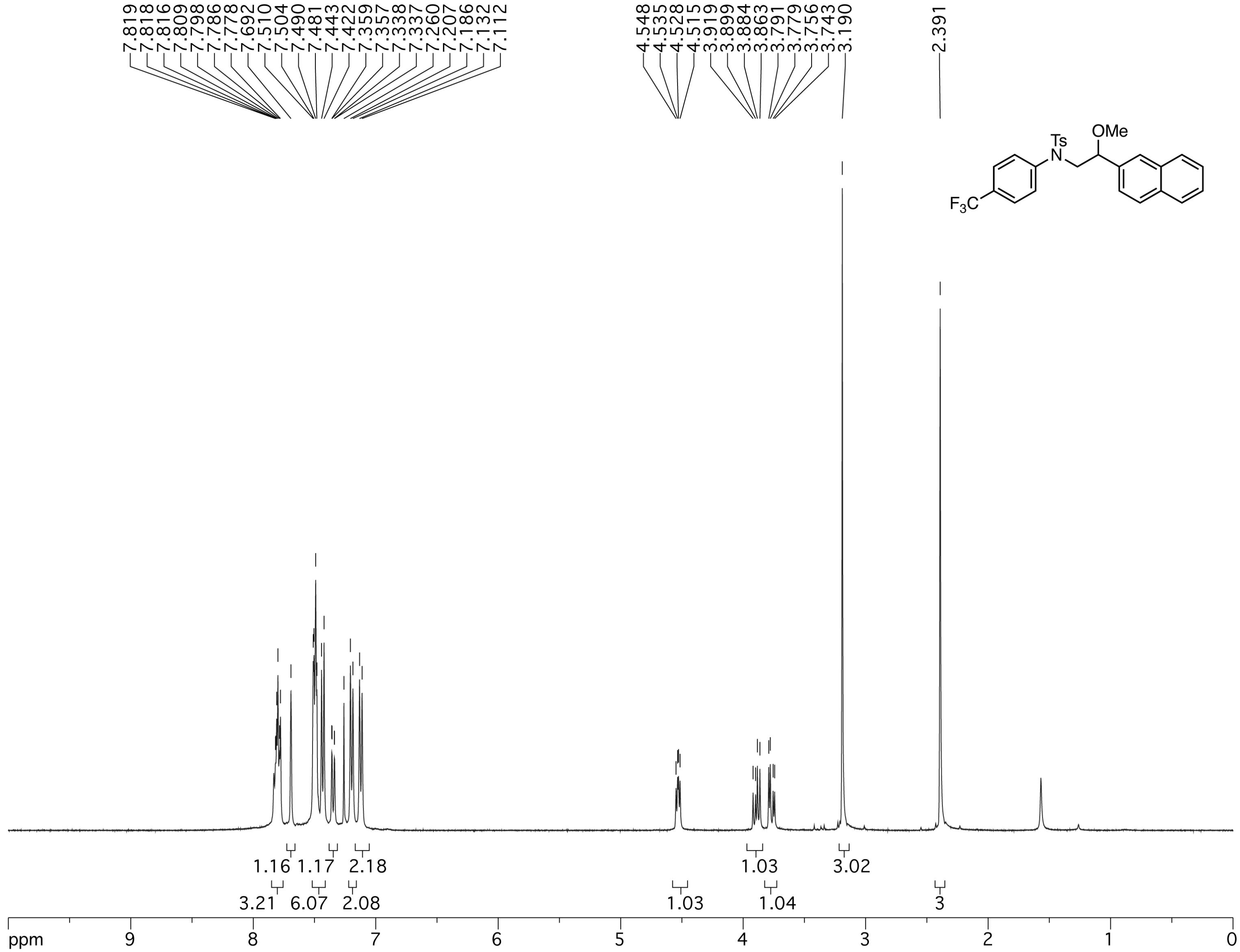

Figure S58. ^{1}H NMR spectrum of **6h** in $CDCl_3$ (400 MHz) measured at 23 °C.

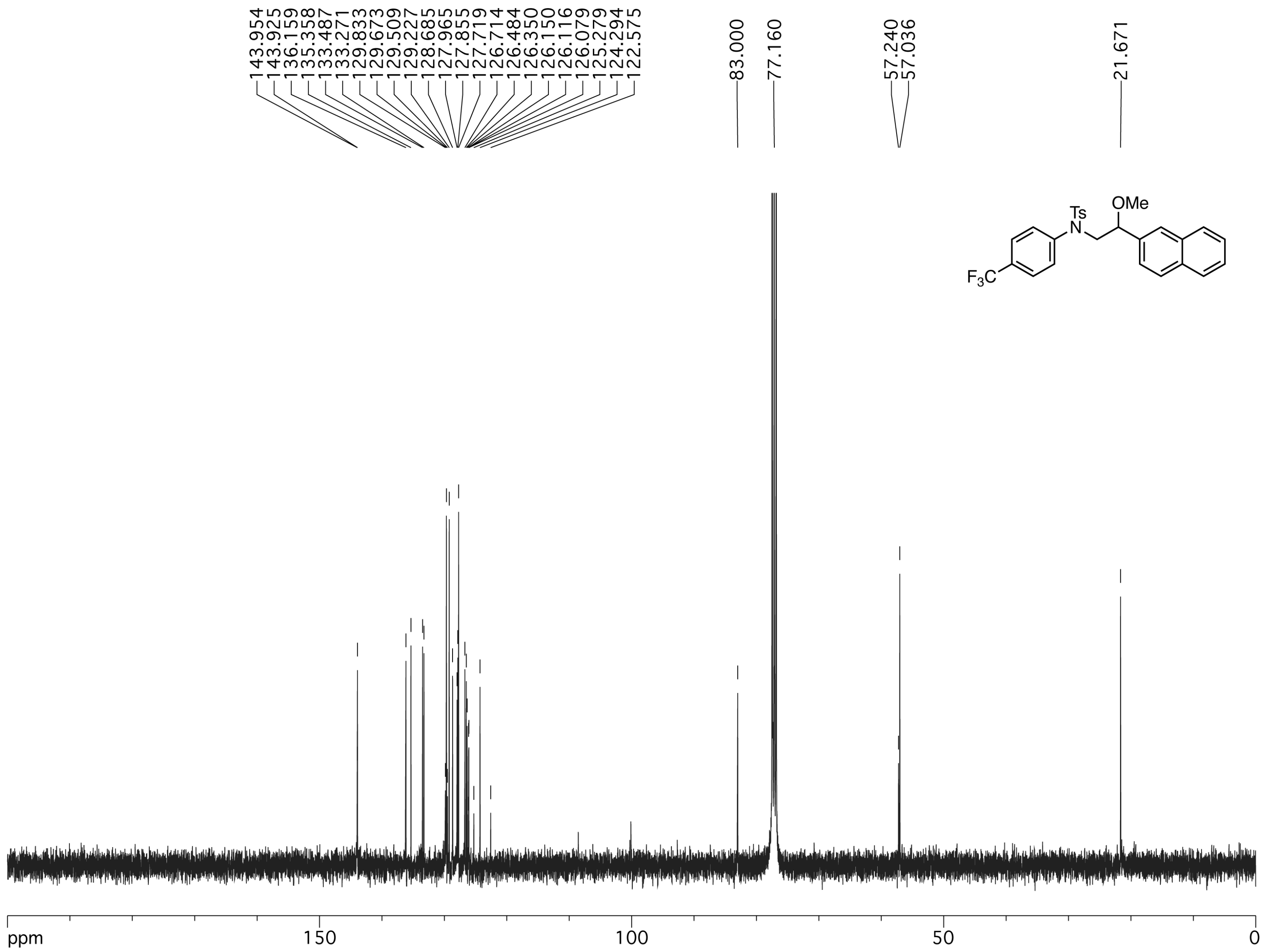

Figure S59. ^{13}C NMR spectrum of **6h** in $CDCl_3$ (100 MHz) measured at 23 °C.

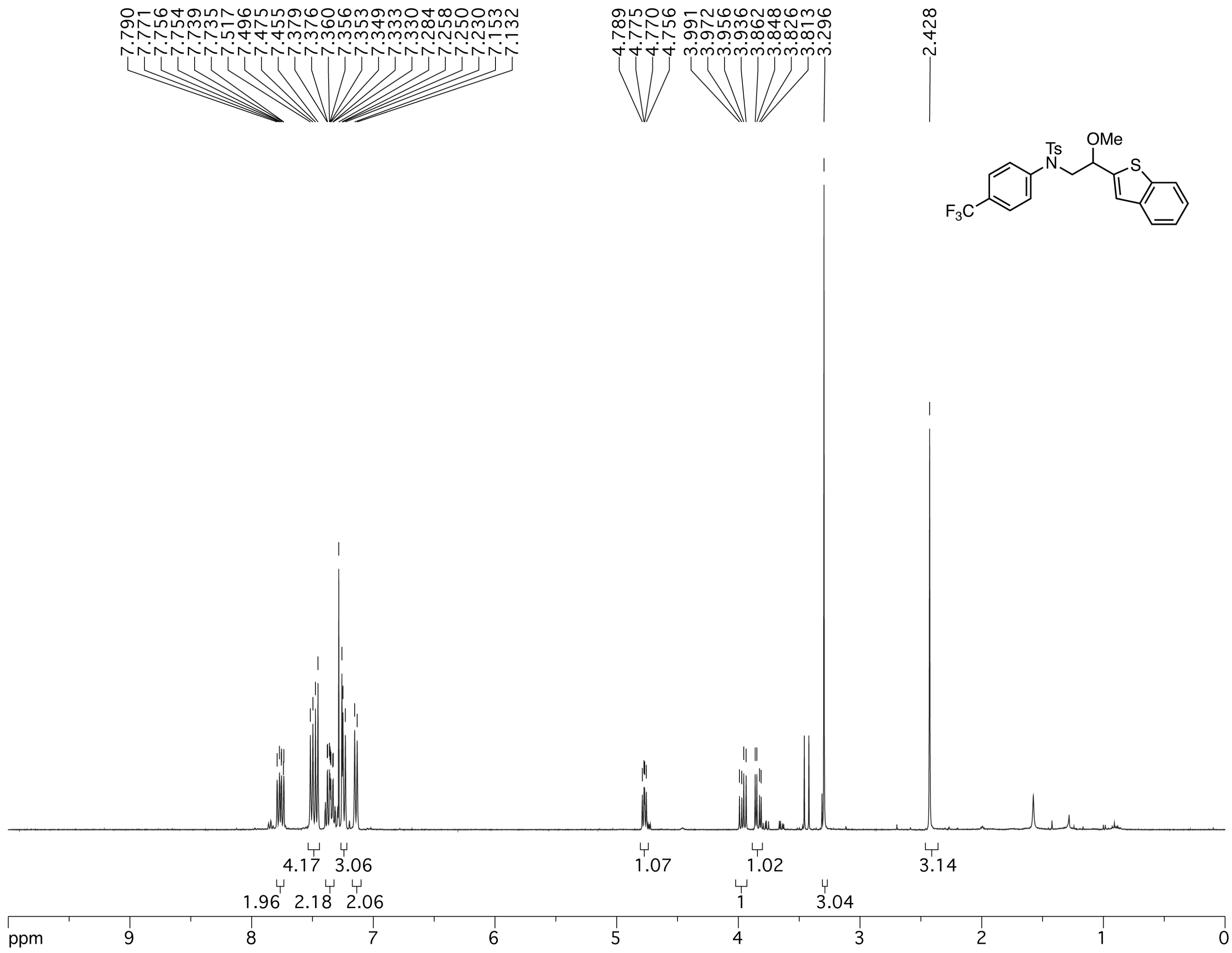

Figure S60. ^{1}H NMR spectrum of **6i** in $CDCl_3$ (400 MHz) measured at 23 °C.

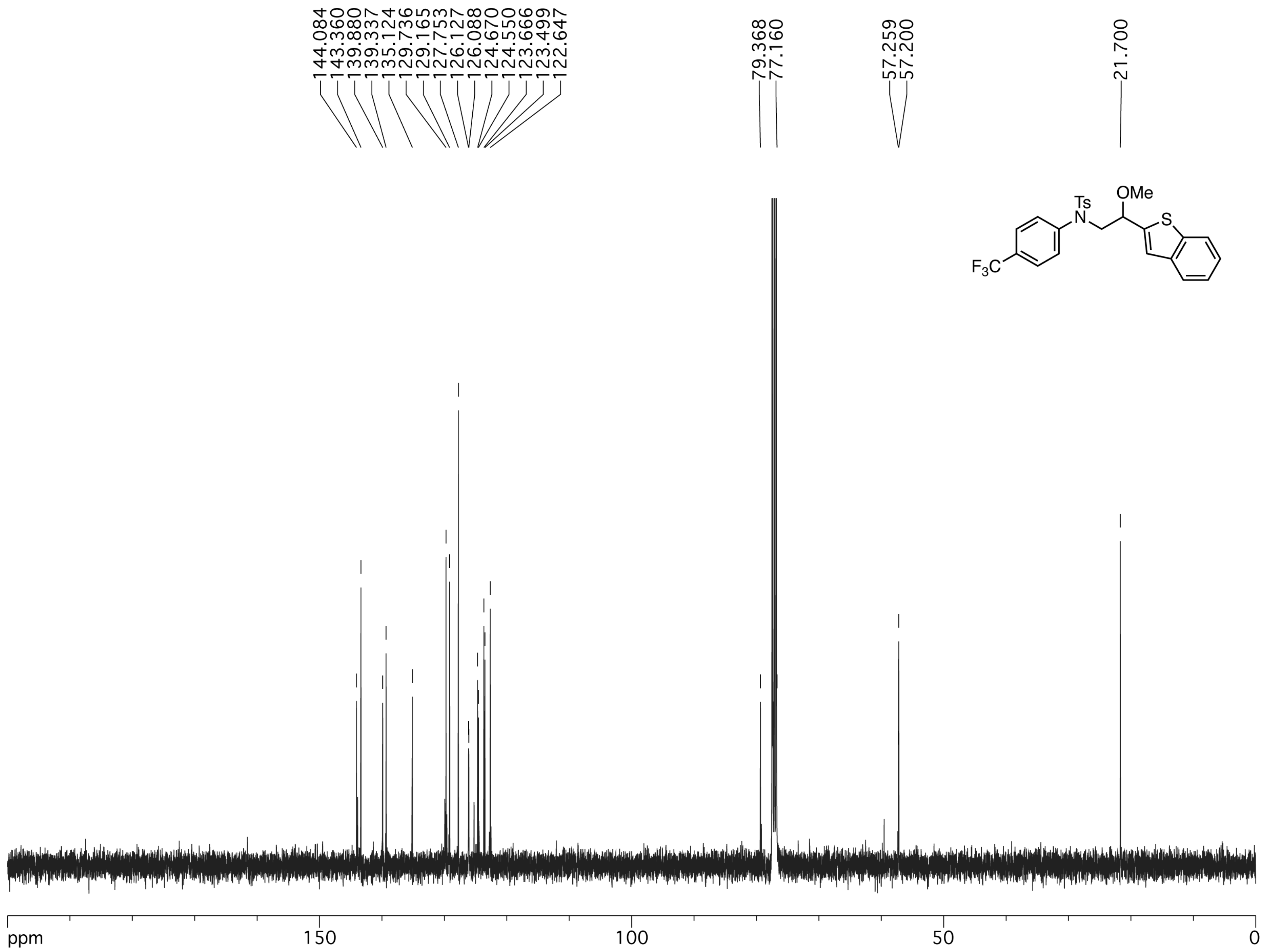

Figure S61. ^{13}C NMR spectrum of **6i** in $CDCl_3$ (100 MHz) measured at 23 °C.

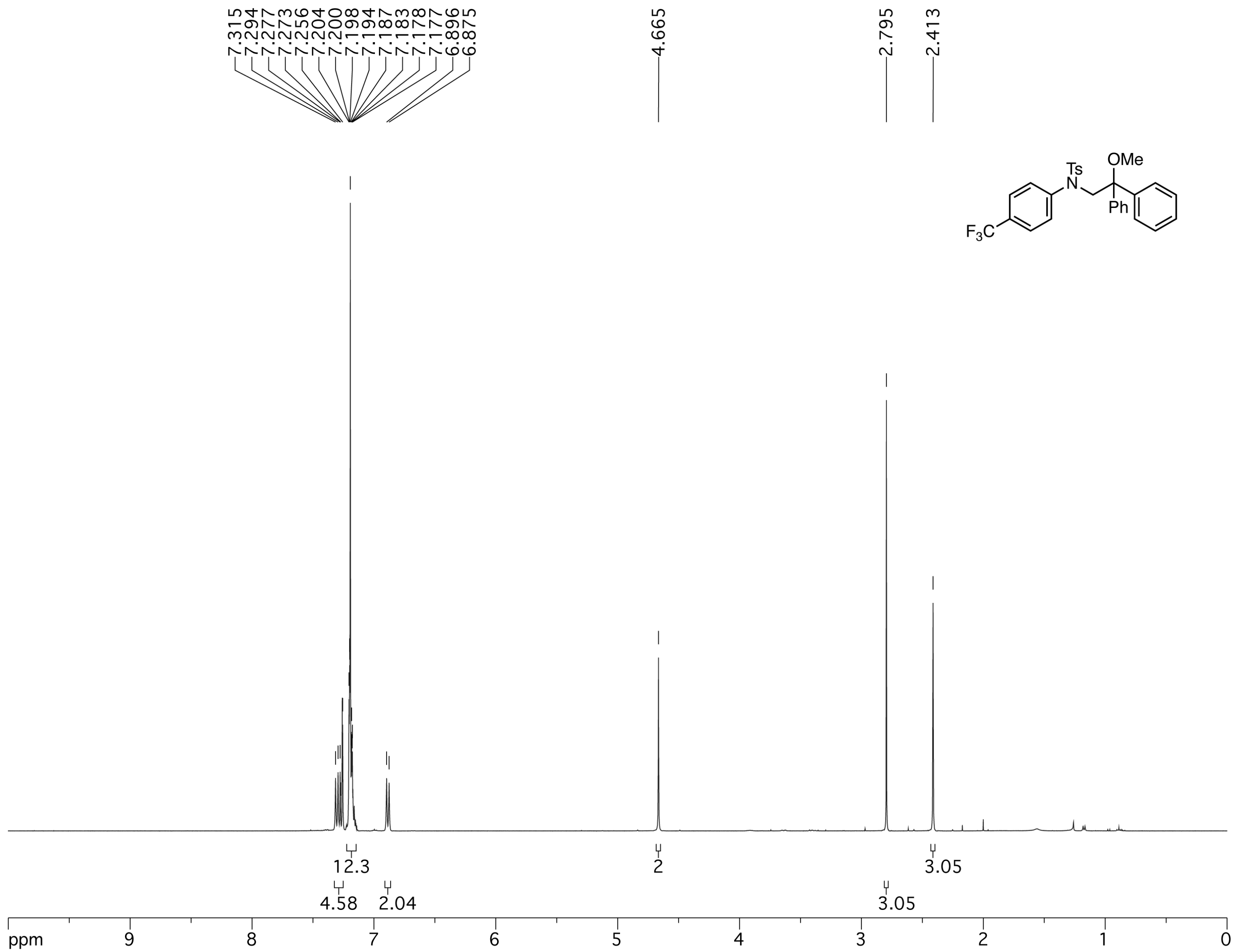

Figure S62. ¹H NMR spectrum of **6j** in $CDCl_3$ (400 MHz) measured at 23 °C.

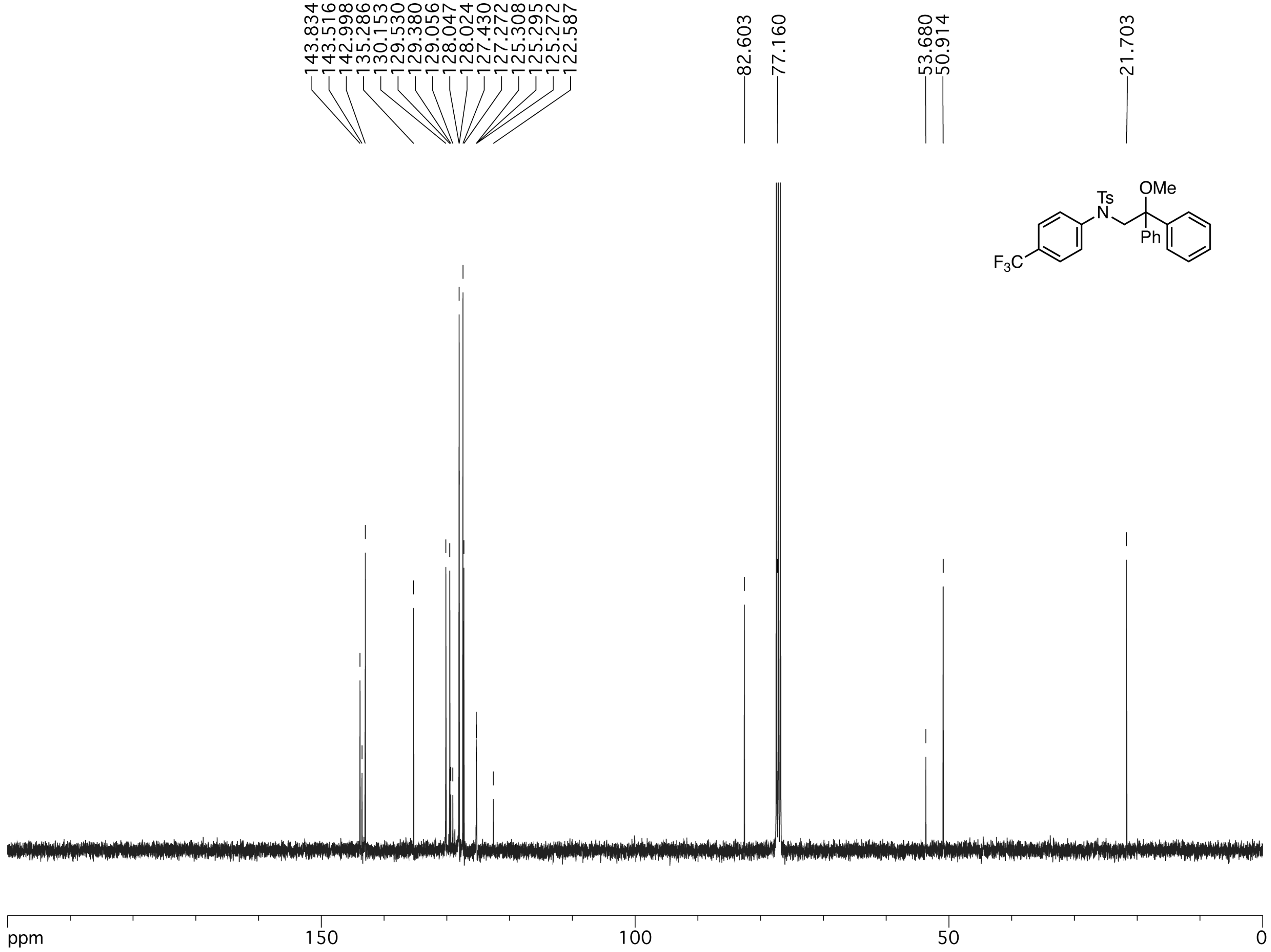

Figure S63. ^{13}C NMR spectrum of **6j** in $CDCl_3$ (100 MHz) measured at 23 °C.

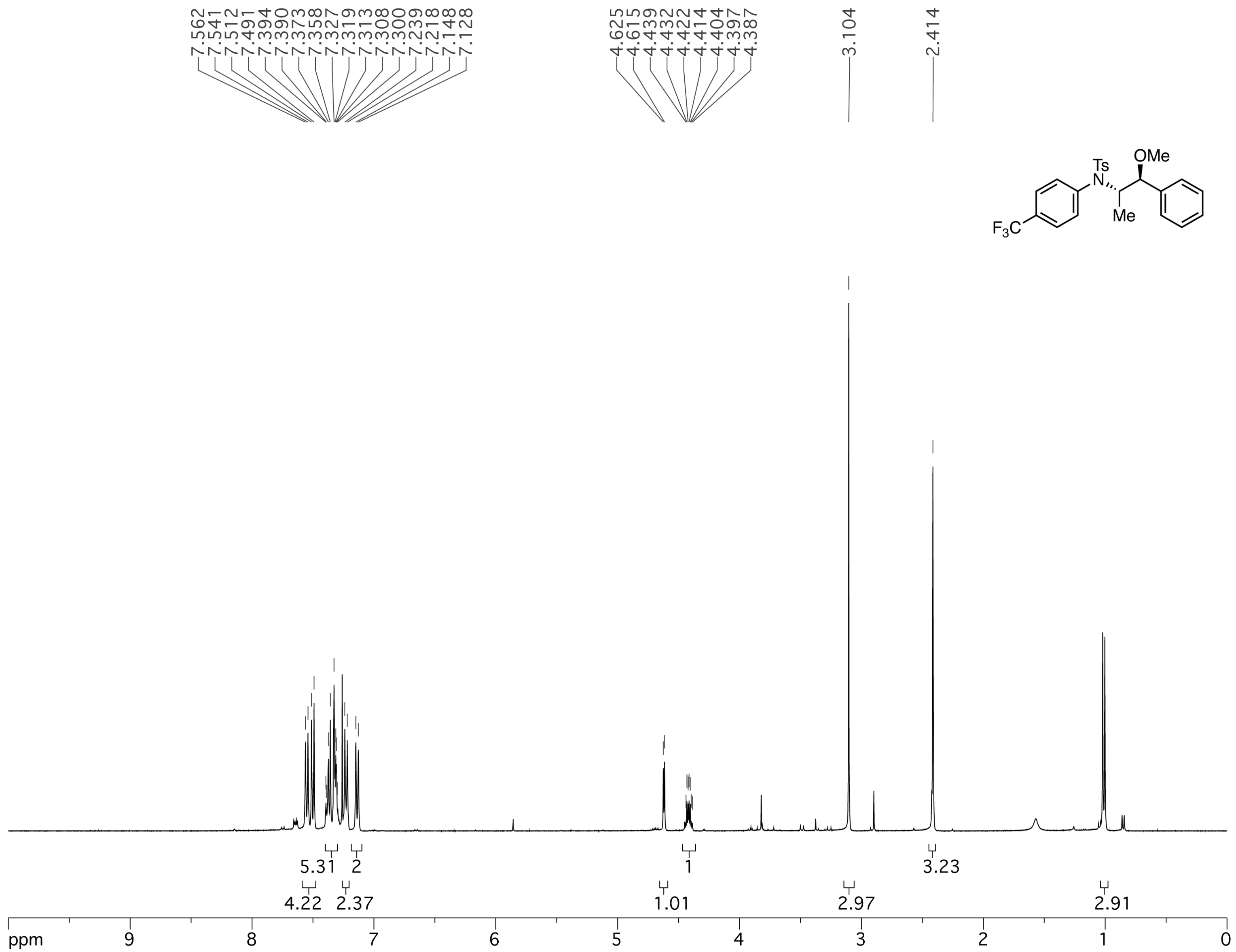

Figure S64. ^{1}H NMR spectrum of **6k** in $CDCl_3$ (400 MHz) measured at 23 °C.

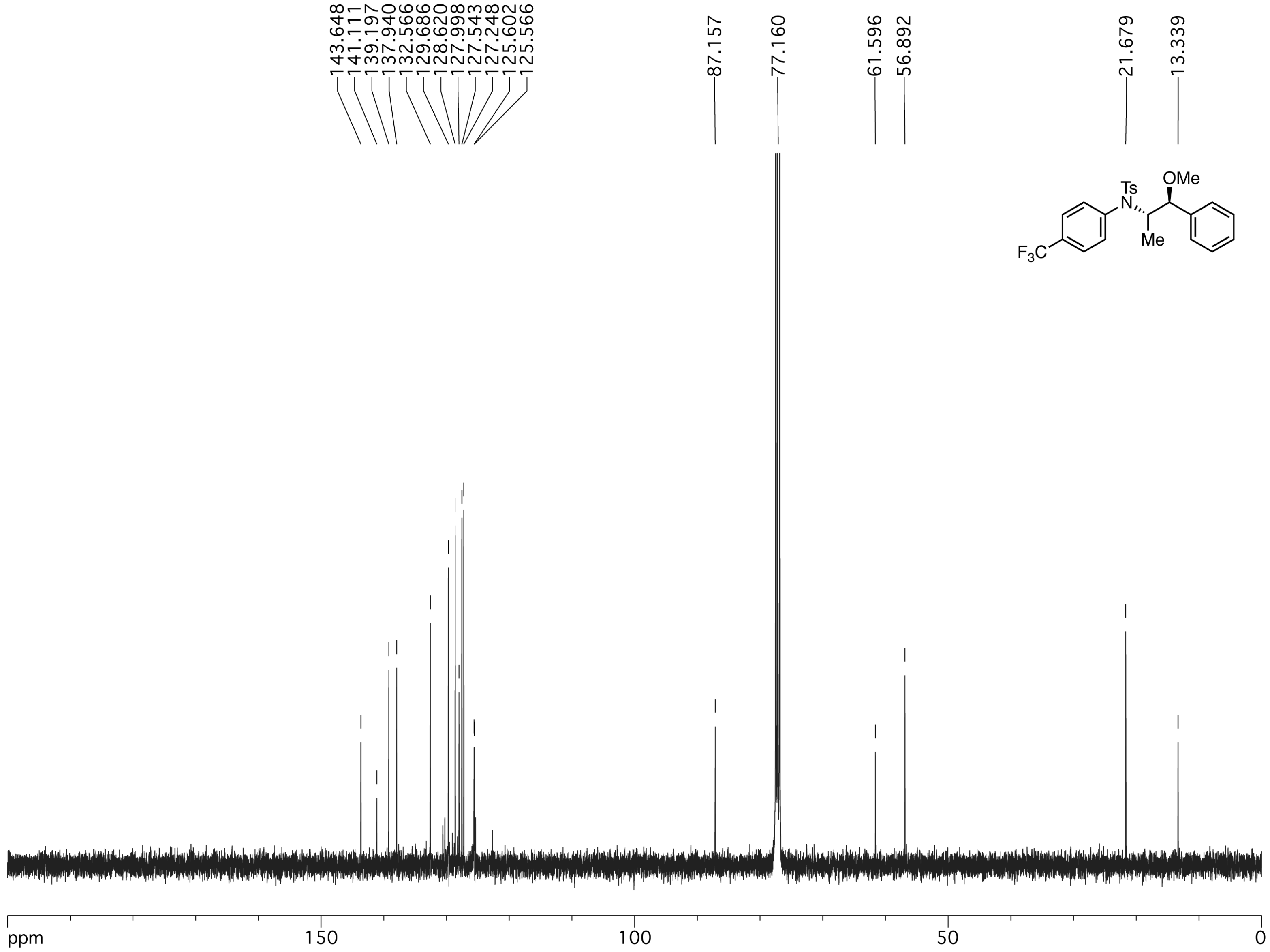

Figure S65. ^{13}C NMR spectrum of **6k** in $CDCl_3$ (100 MHz) measured at 23 °C.

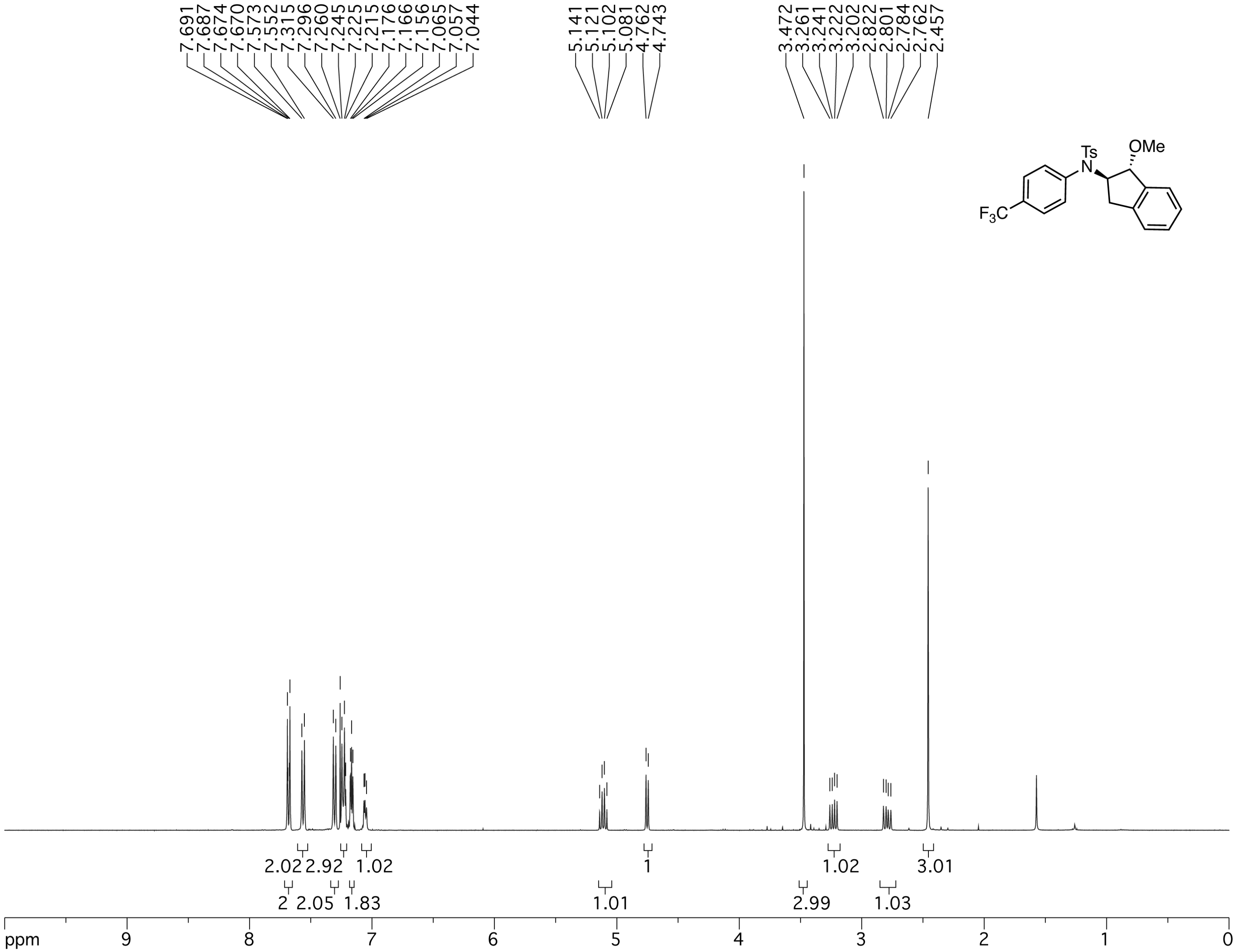

Figure S66. ^{1}H NMR spectrum of trans-**6l** (major diastereomer) in $CDCl_3$ (400 MHz) measured at 23 °C.

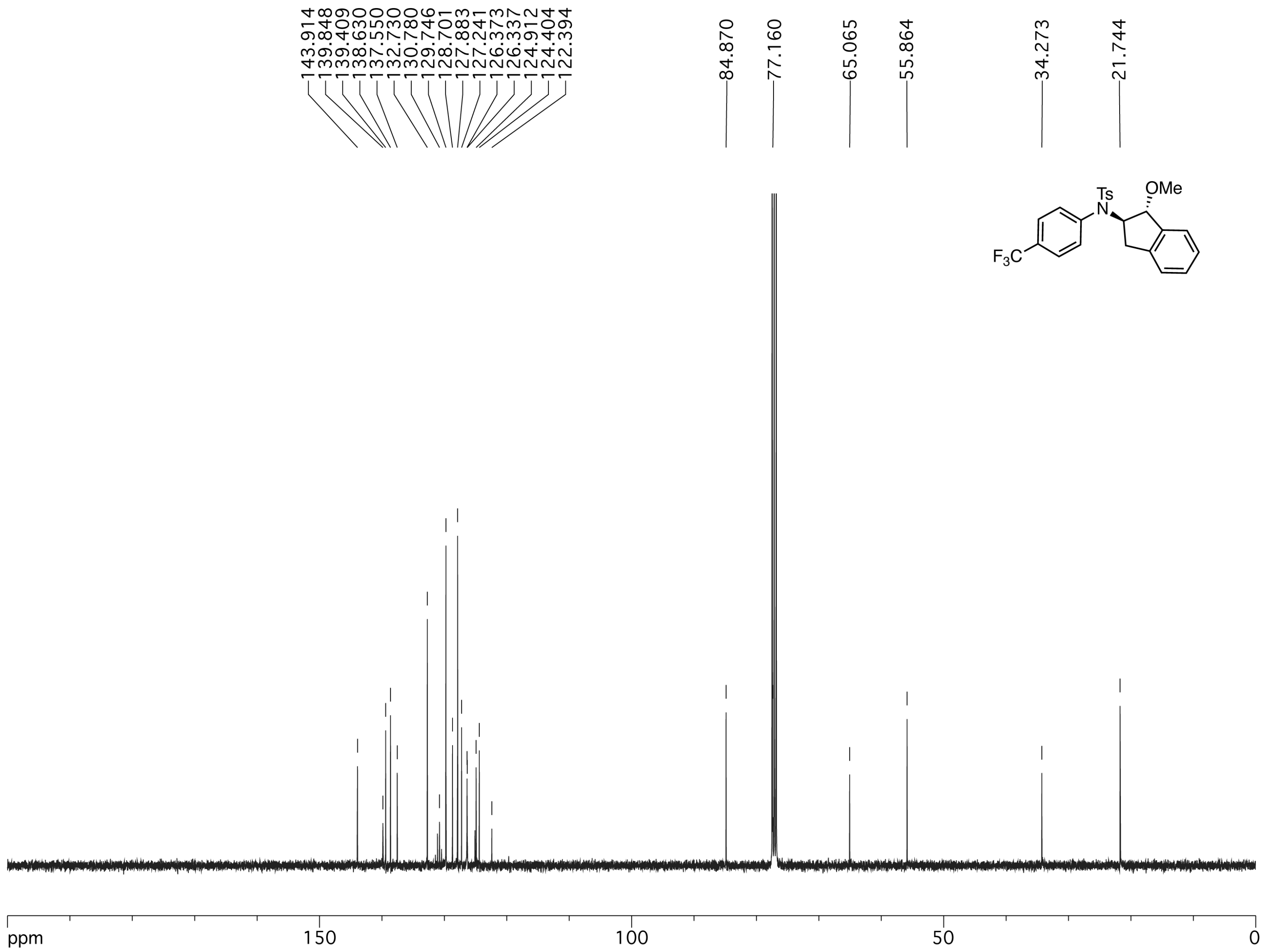

Figure S67. ^{13}C NMR spectrum of trans-**6l** (major diastereomer) in $CDCl_3$ (100 MHz) measured at 23 °C.

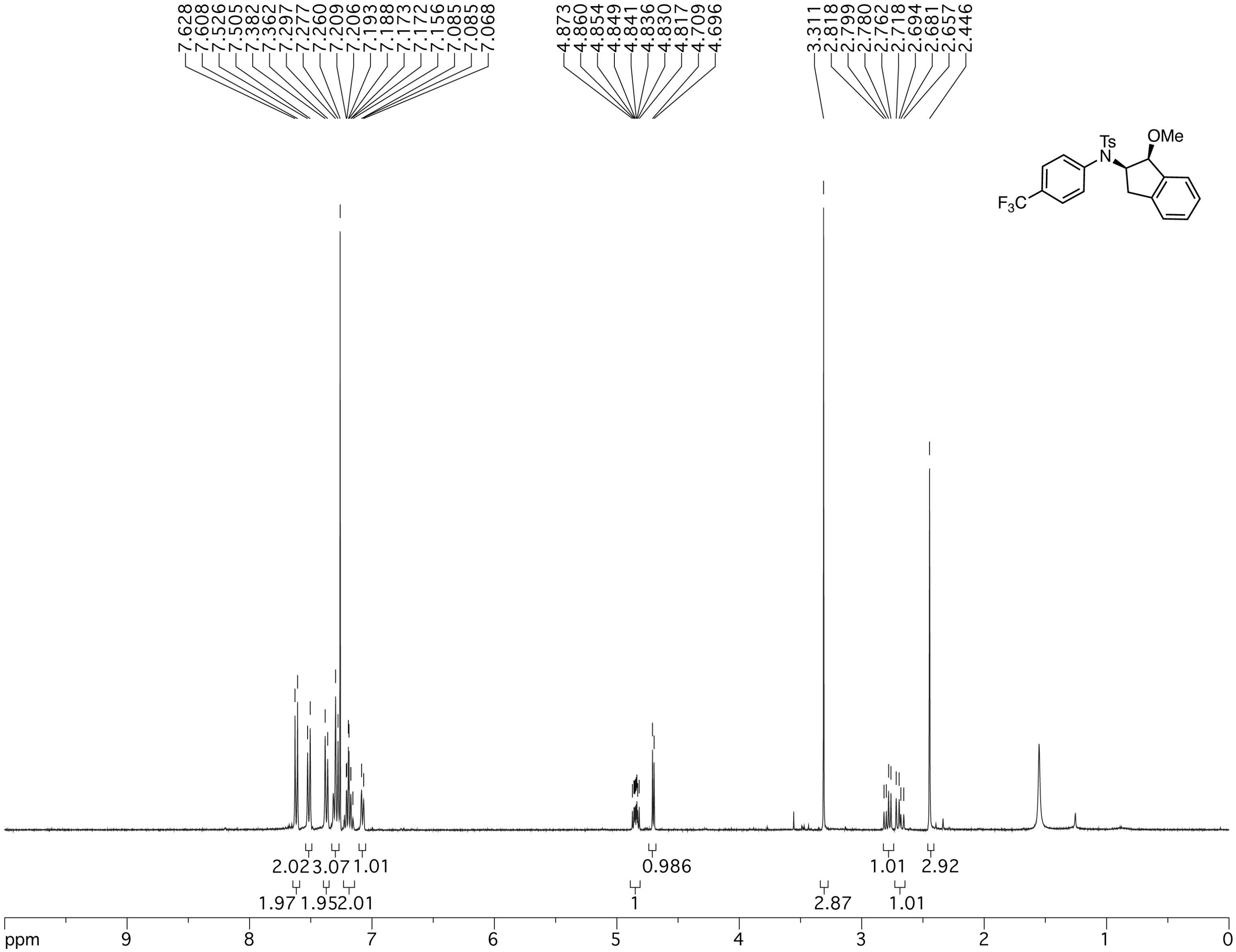

Figure S68. ^{1}H NMR spectrum of cis- **6l** (minor diastereomer) in $CDCl_3$ (400 MHz) measured at 23 °C.

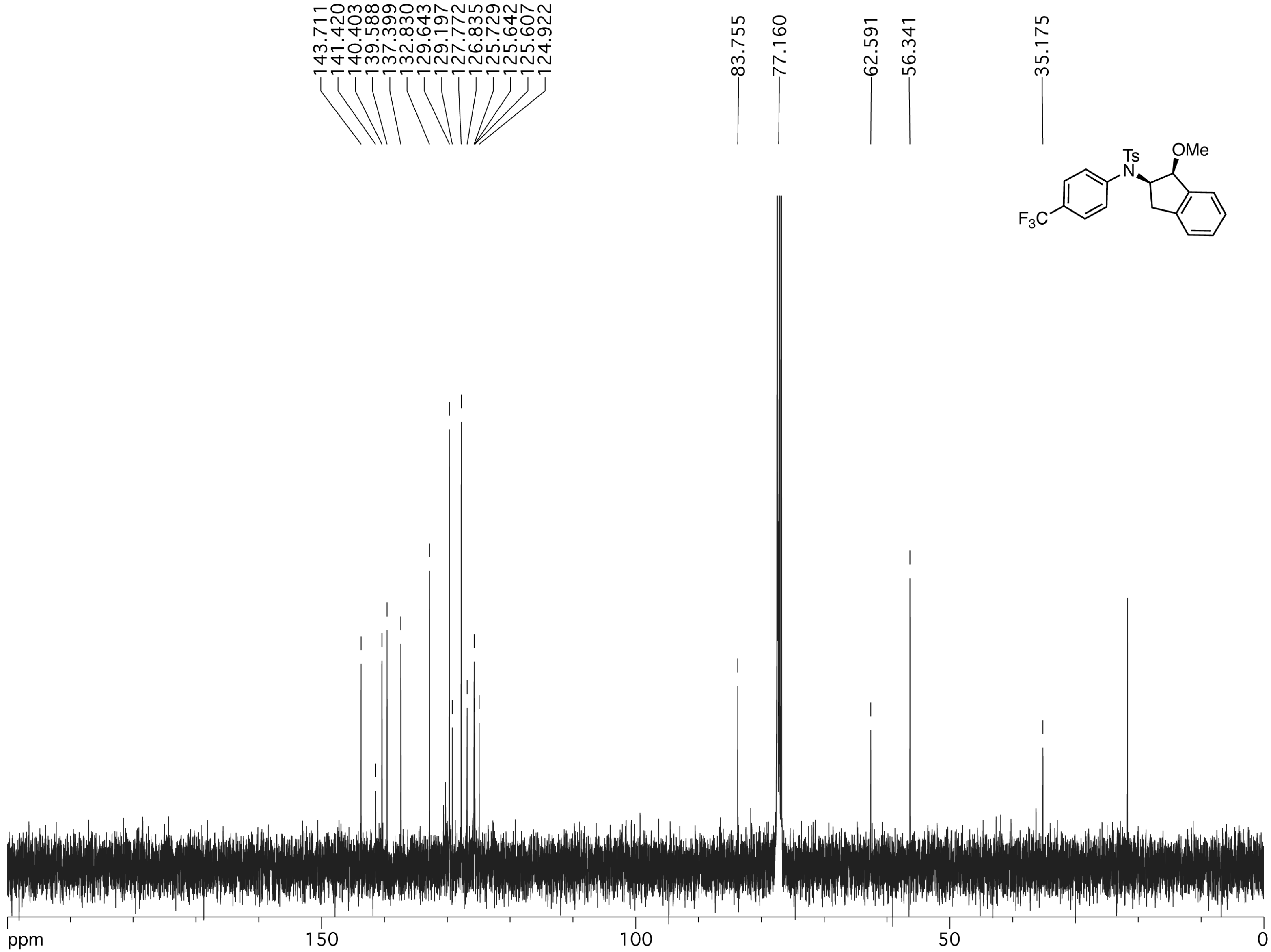

Figure S69. ^{13}C NMR spectrum of **6l** (minor diastereomer) in $CDCl_3$ (100 MHz) measured at 23 °C.

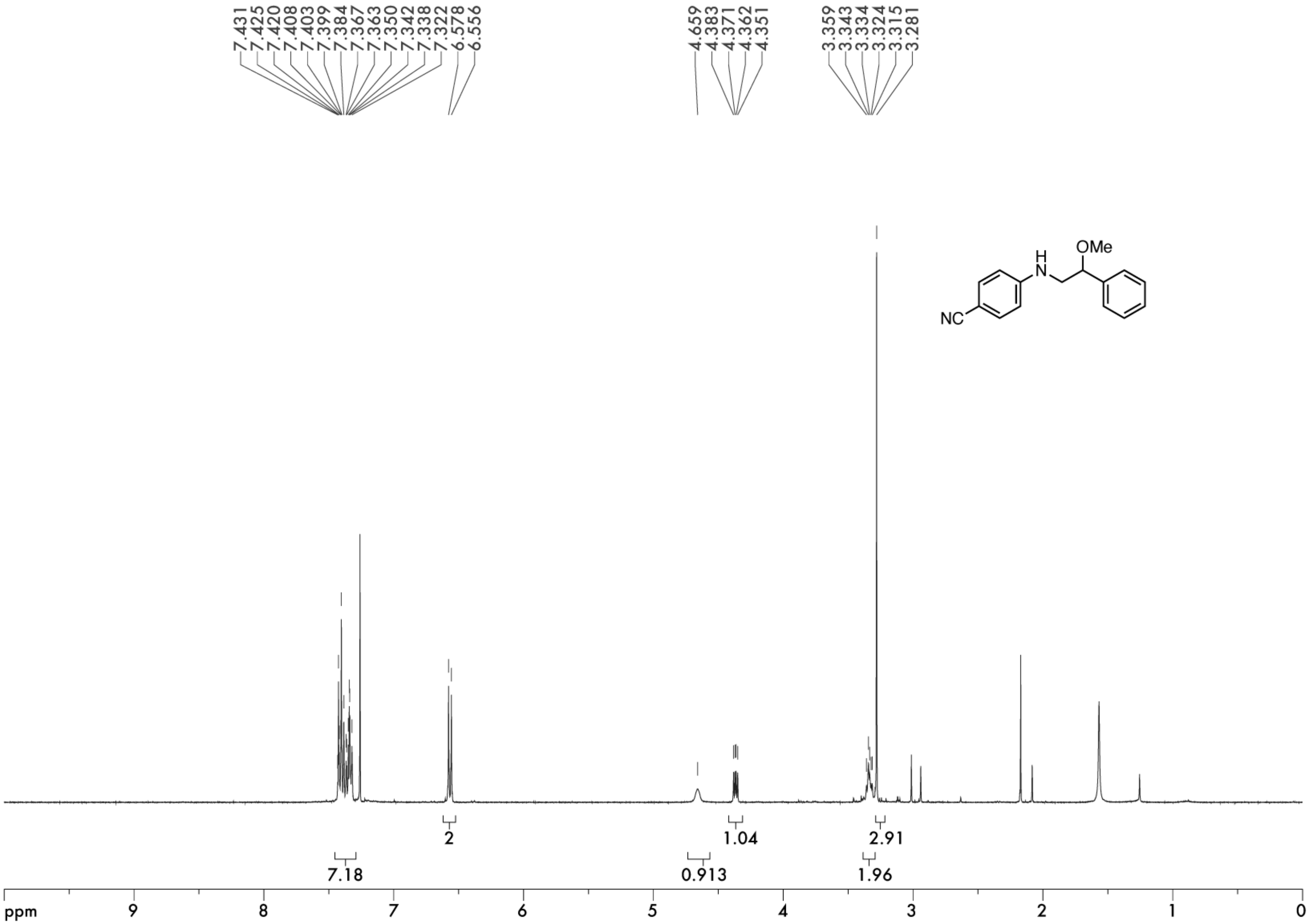

Figure S70. ^{1}H NMR spectrum of **6m** in $CDCl_3$ (400 MHz) measured at 23 °C.

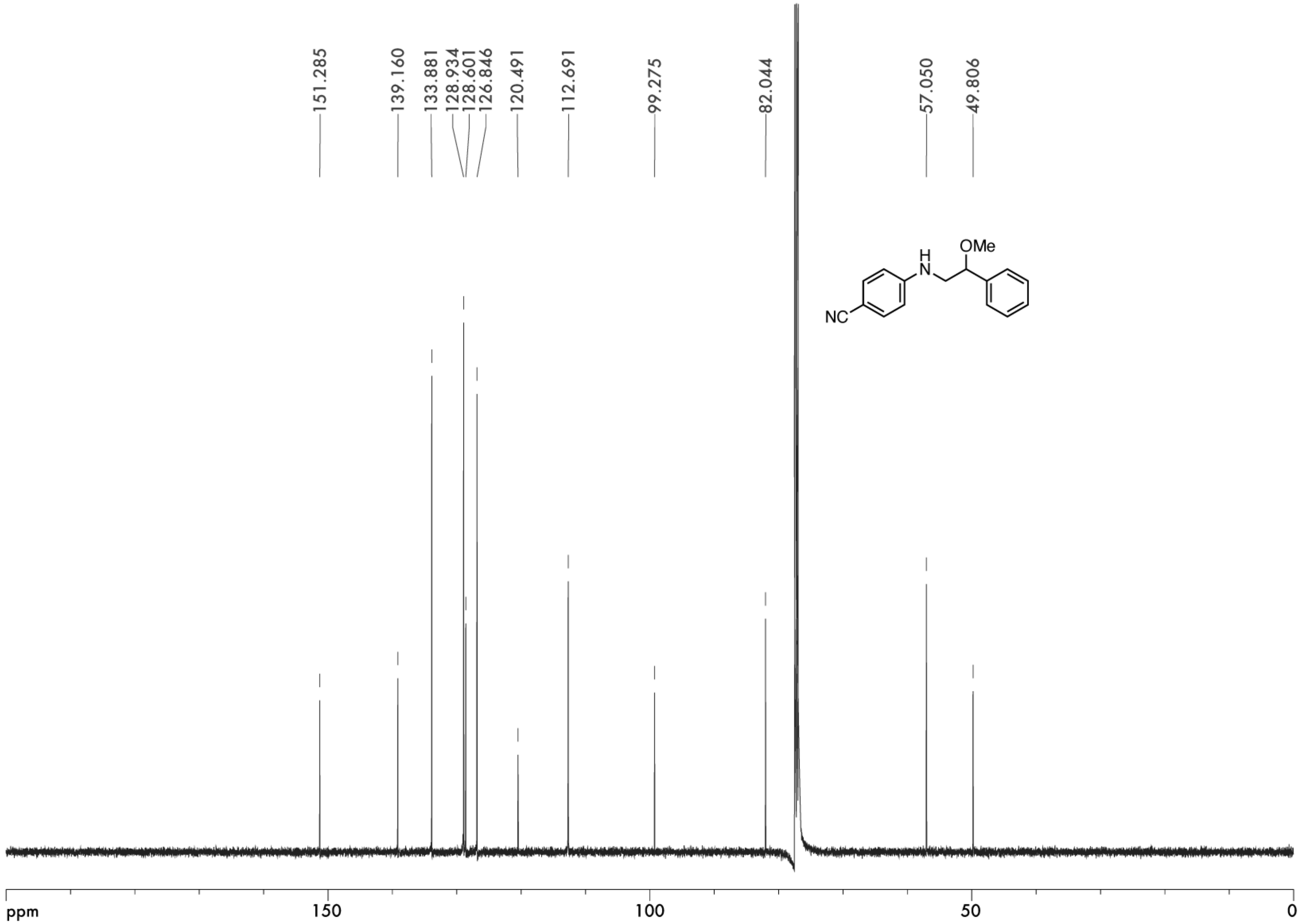

Figure S71. ^{13}C NMR spectrum of **6m** in $CDCl_3$ (100 MHz) measured at 23 °C.

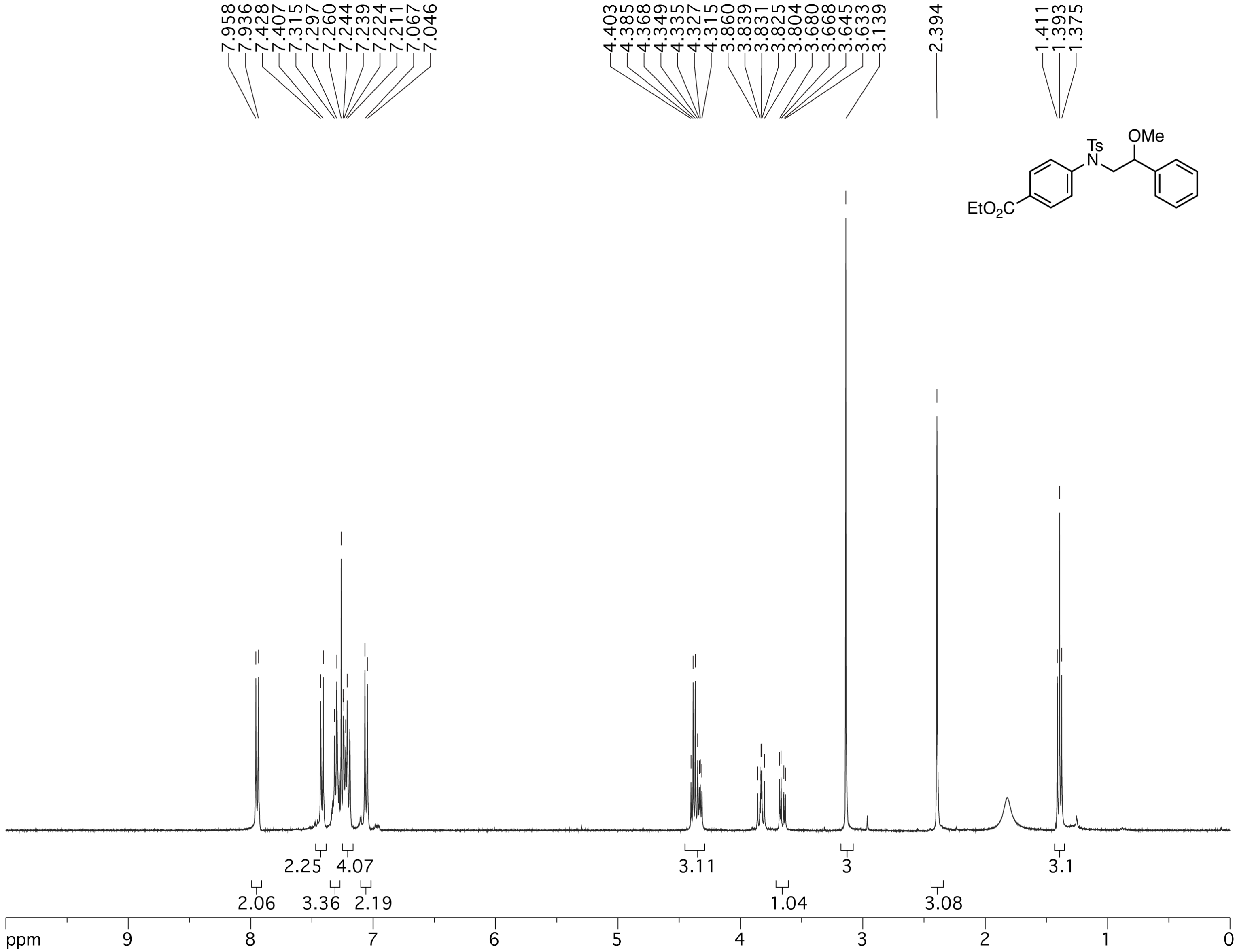

Figure S72. ^{1}H NMR spectrum of **6n** in $CDCl_3$ (400 MHz) measured at 23 °C.

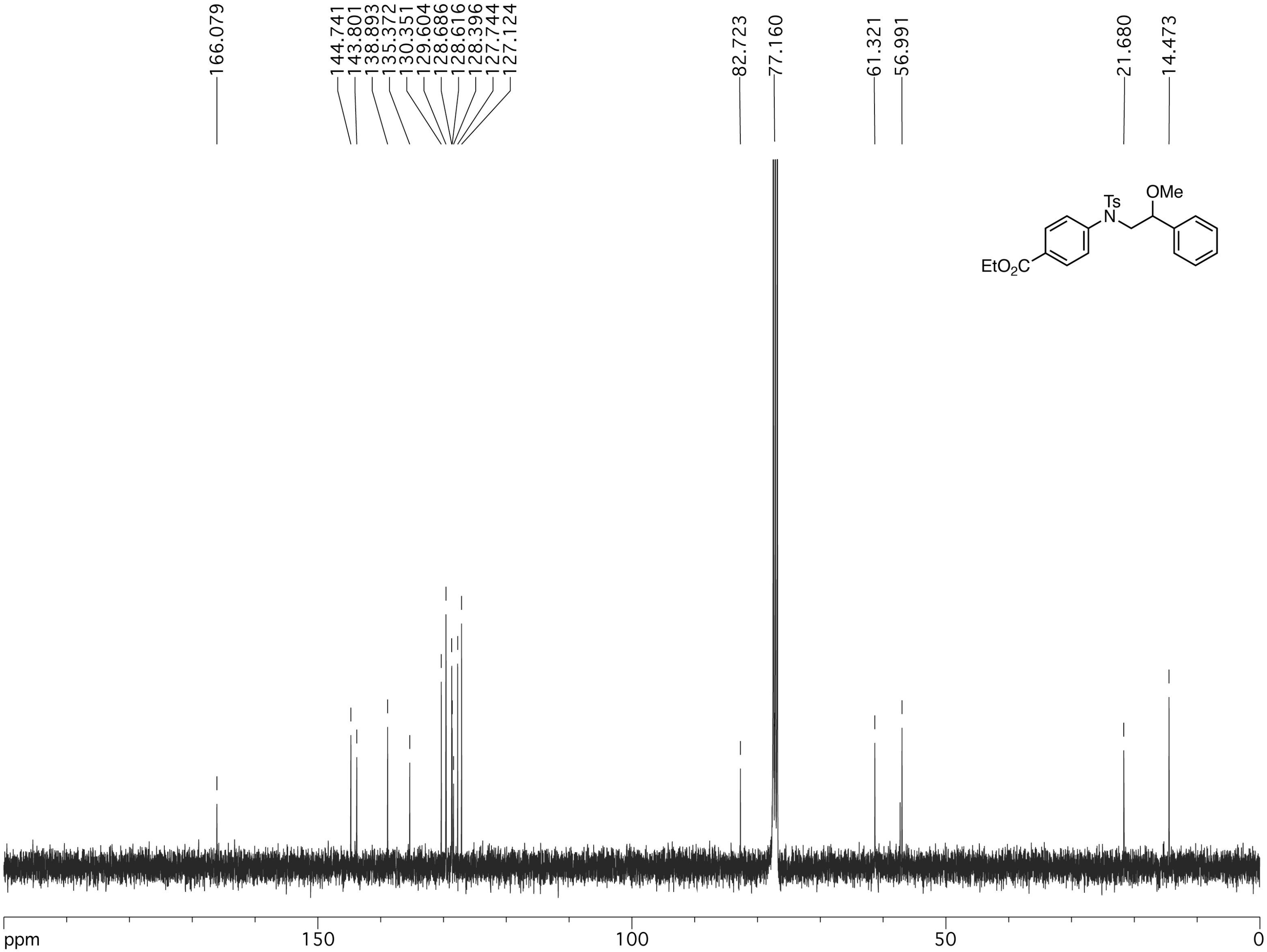

Figure S73. ^{13}C NMR spectrum of **6n** in $CDCl_3$ (100 MHz) measured at 23 °C.

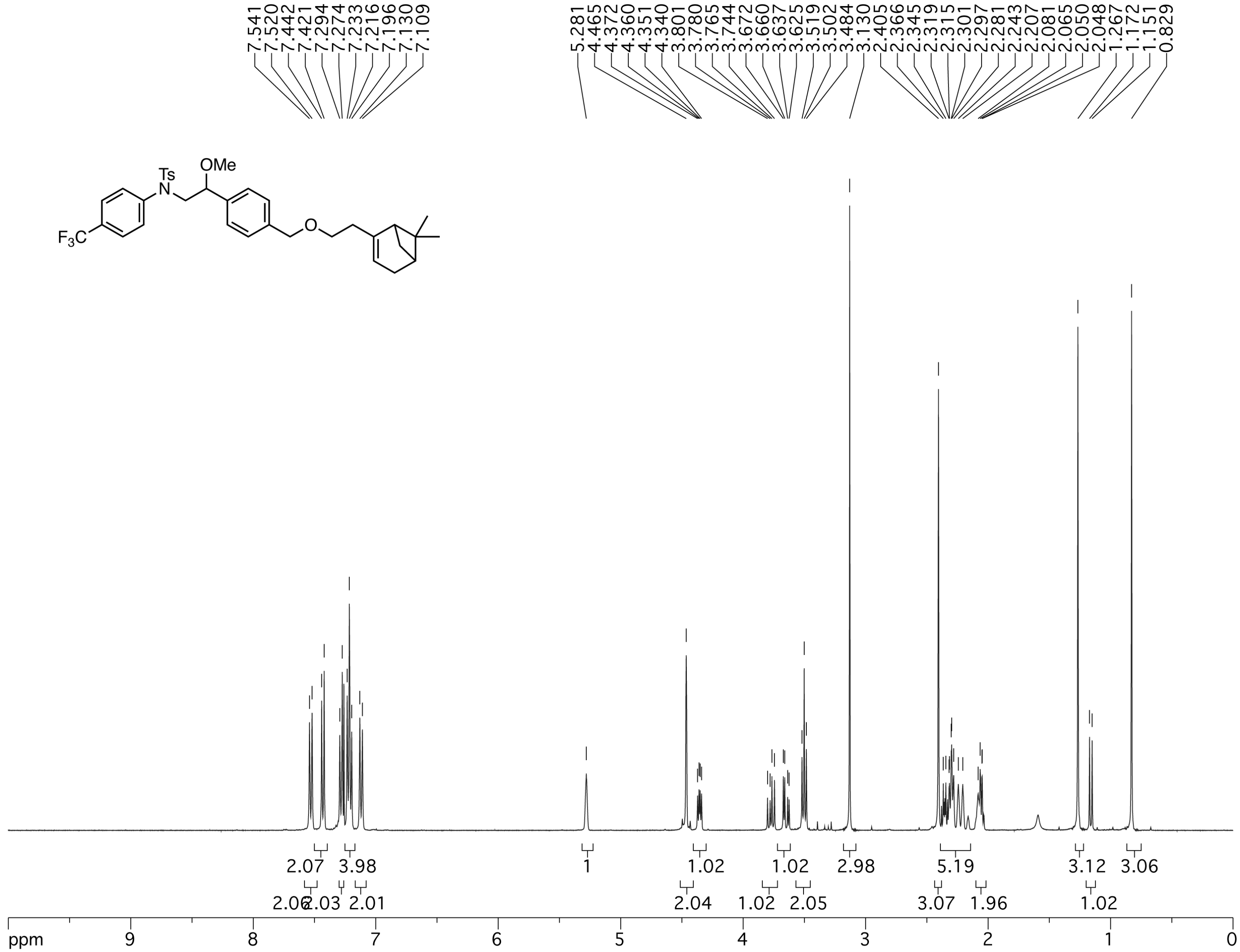

Figure S74. ^{1}H NMR spectrum of **6o** in $CDCl_3$ (400 MHz) measured at 23 °C.

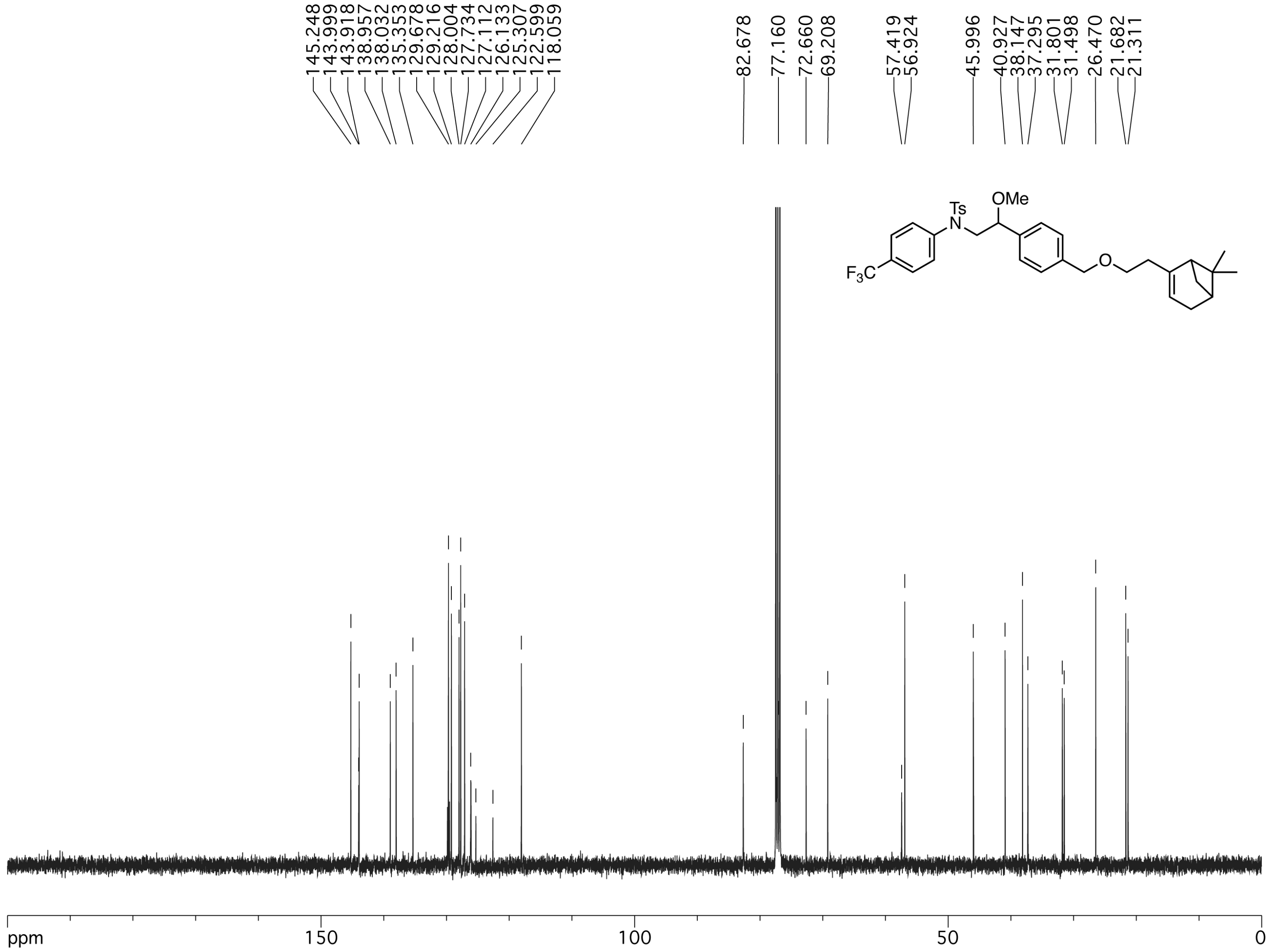

Figure S75. ^{13}C NMR spectrum of **6o** in $CDCl_3$ (100 MHz) measured at 23 °C.

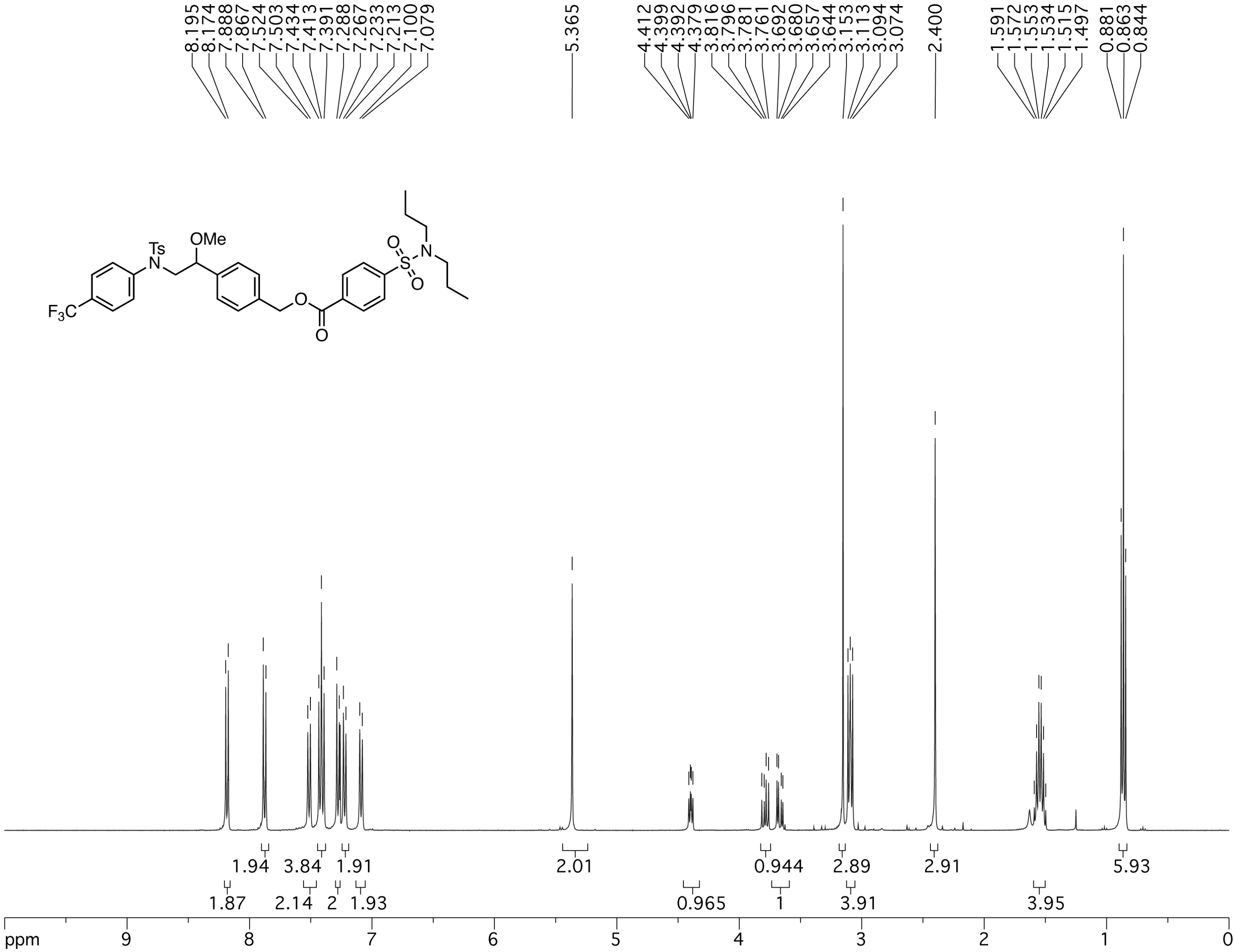

Figure S76. ^{1}H NMR spectrum of **6p** in $CDCl_3$ (400 MHz) measured at 23 °C.

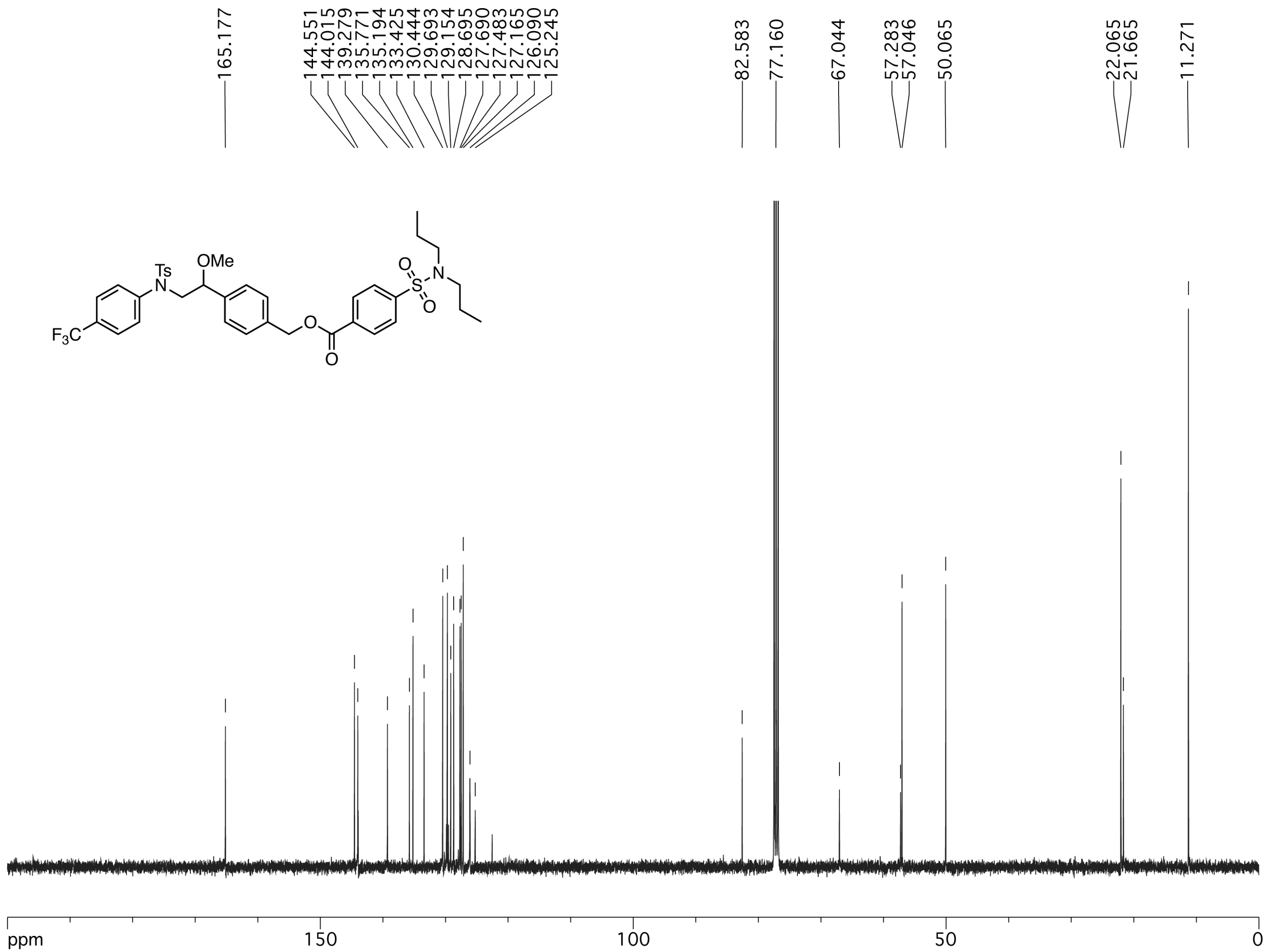

Figure S77. ^{13}C NMR spectrum of **6p** in $CDCl_3$ (100 MHz) measured at 23 °C.

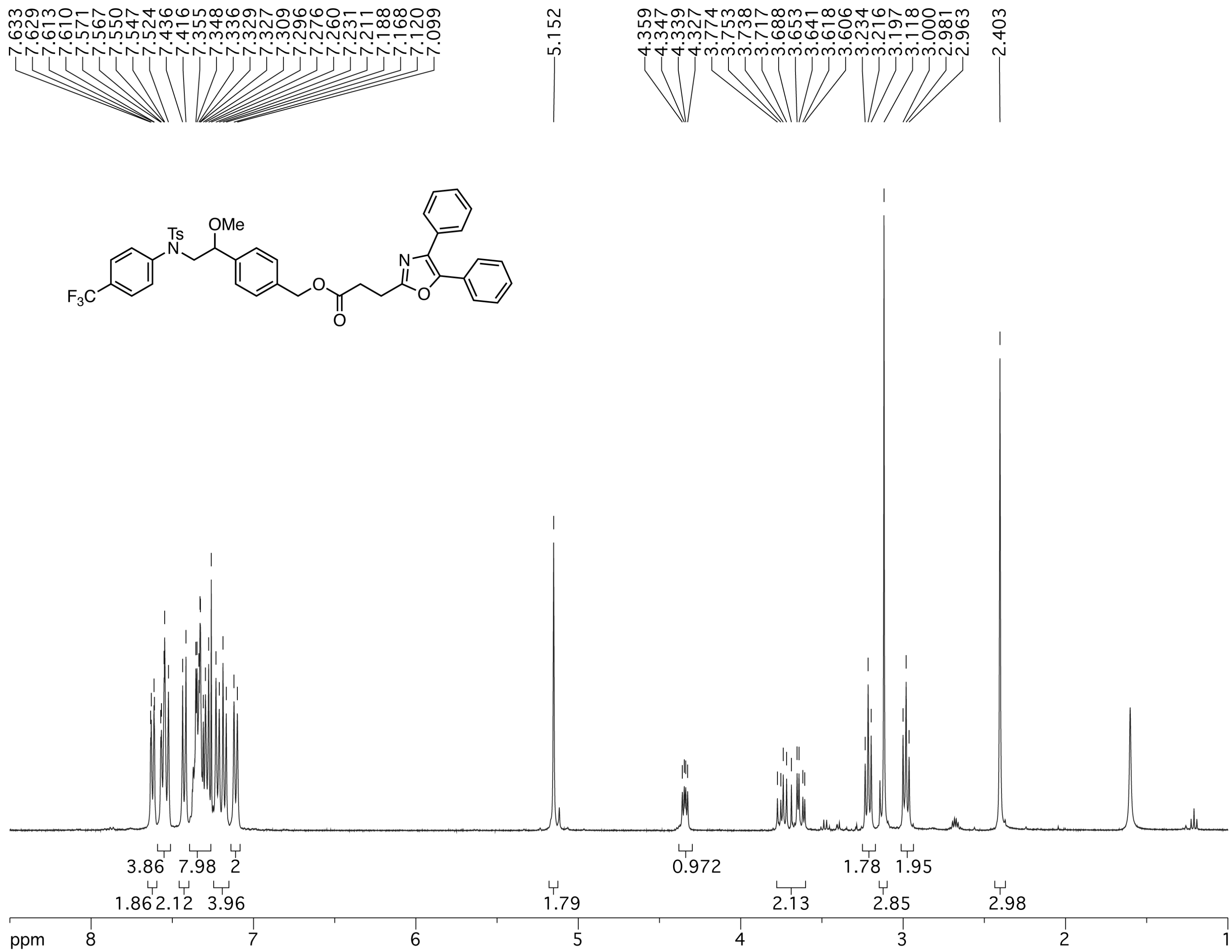

Figure S78. ^{1}H NMR spectrum of **6q** in $CDCl_3$ (400 MHz) measured at 23 °C.

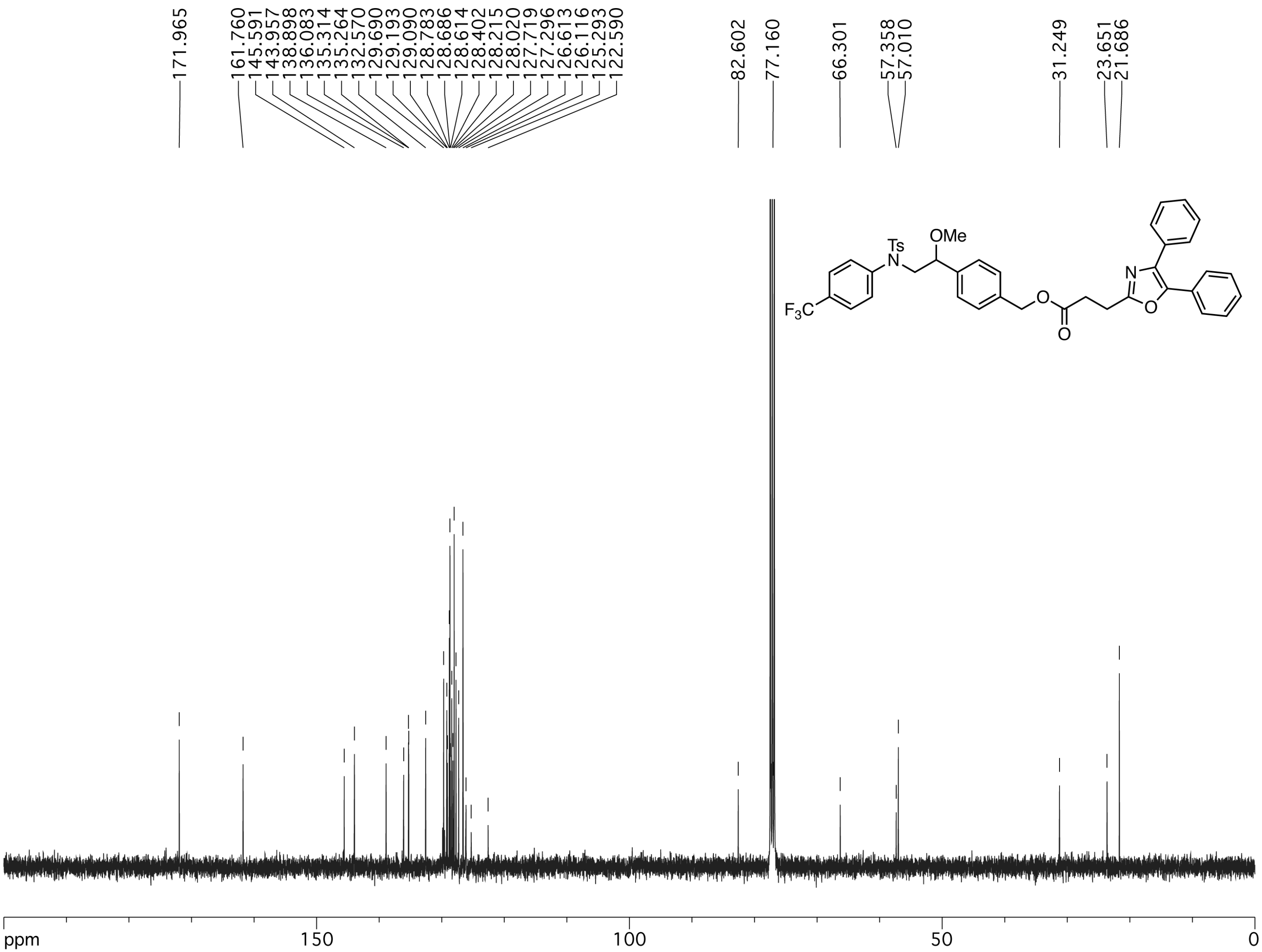

Figure S79. ^{13}C NMR spectrum of **6q** in $CDCl_3$ (100 MHz) measured at 23 °C.

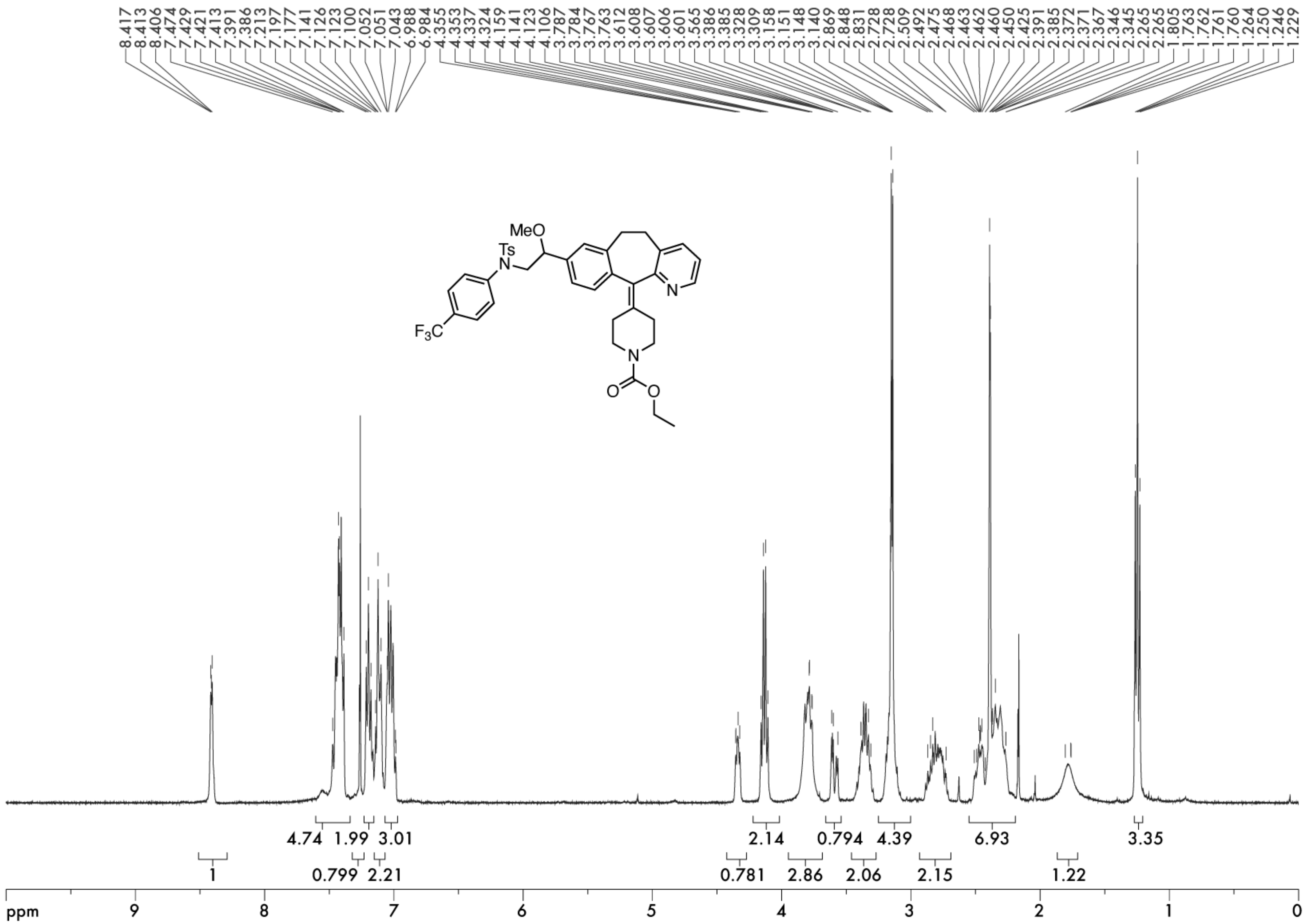

Figure S80. ^{1}H NMR spectrum of **6r** in $CDCl_3$ (400 MHz) measured at 23 °C.

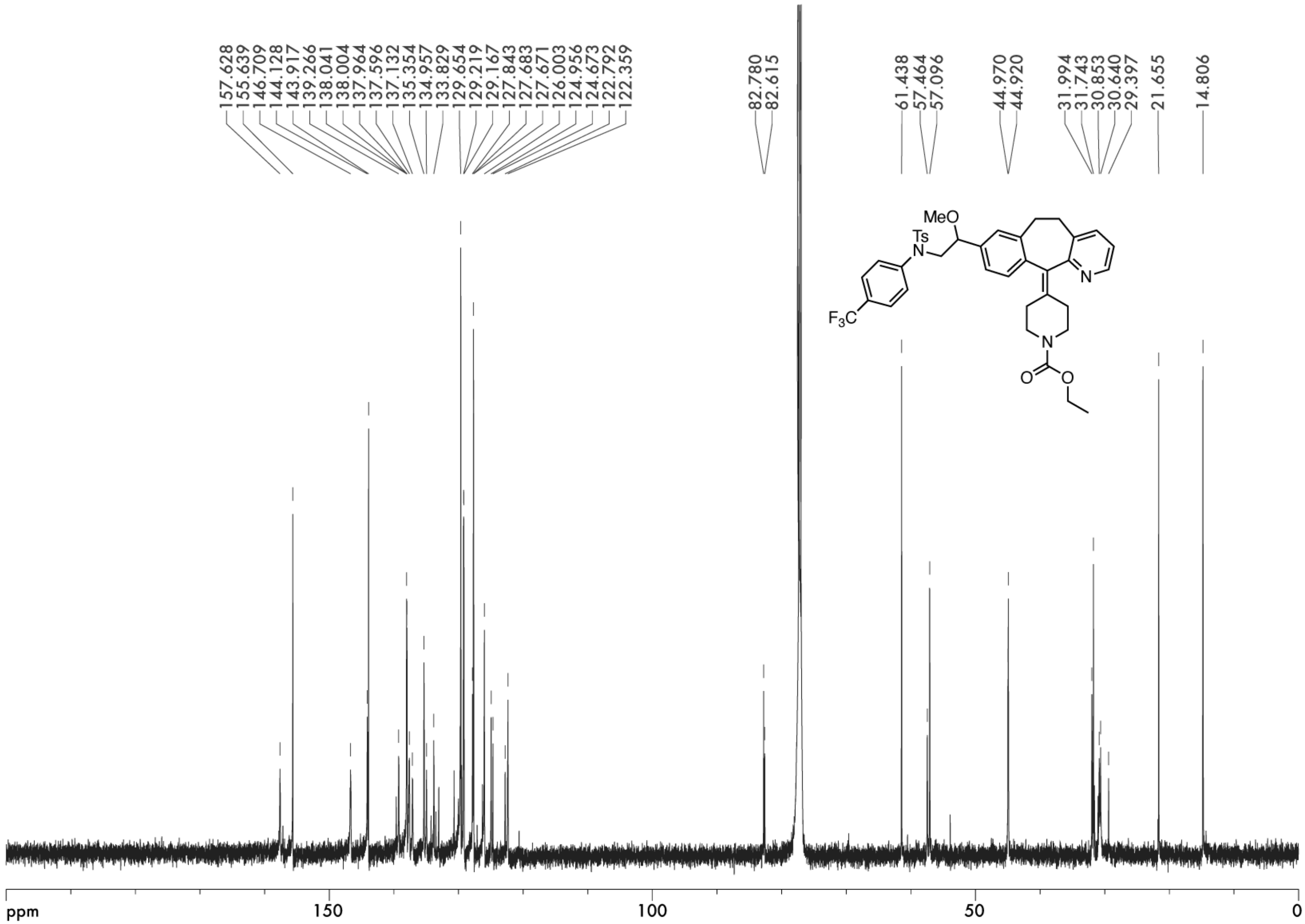

Figure S81. ^{13}C NMR spectrum of **6r** in $CDCl_3$ (100 MHz) measured at 23 °C.

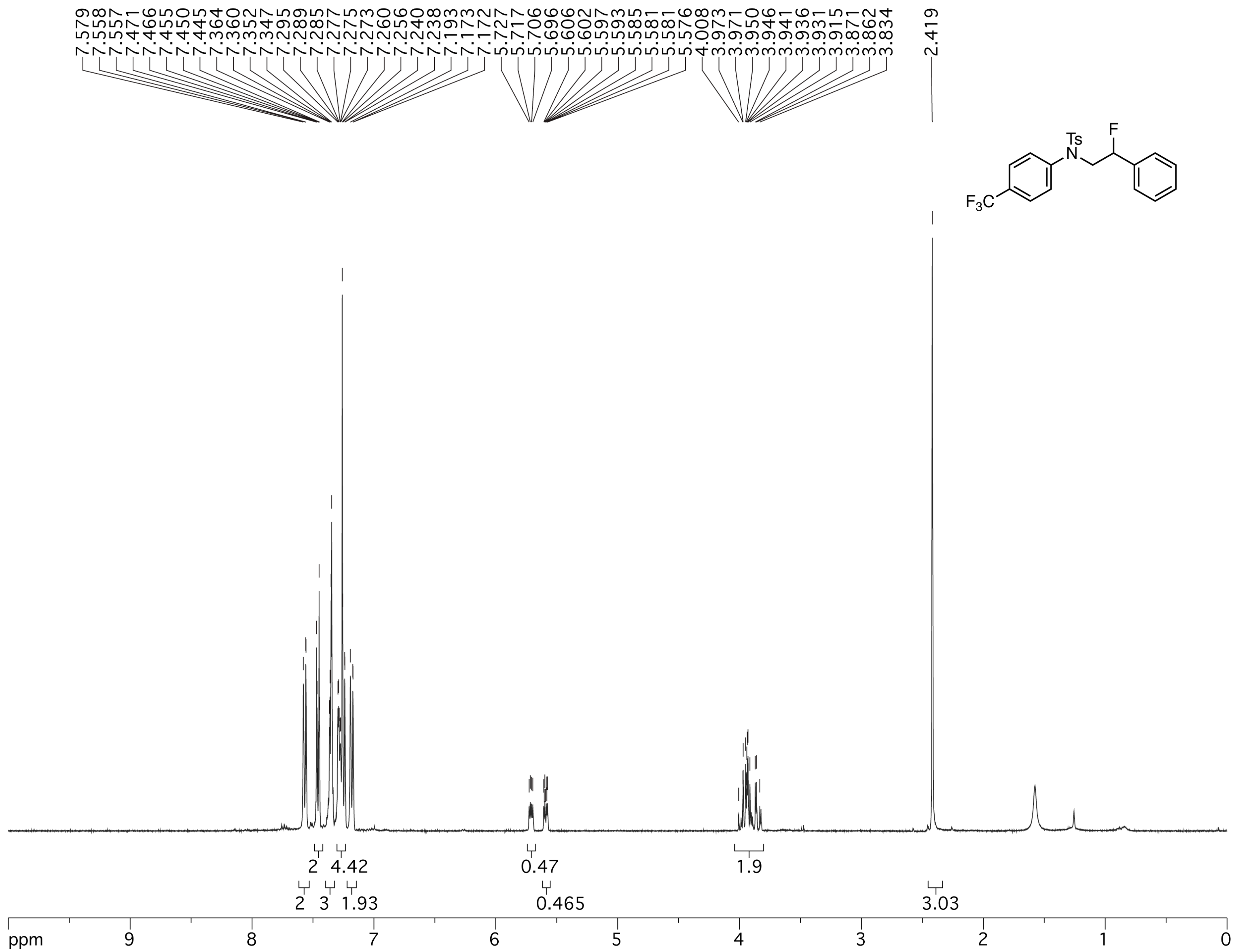

Figure S82. ^{1}H NMR spectrum of **6aa** in $CDCl_3$ (400 MHz) measured at 23 °C.

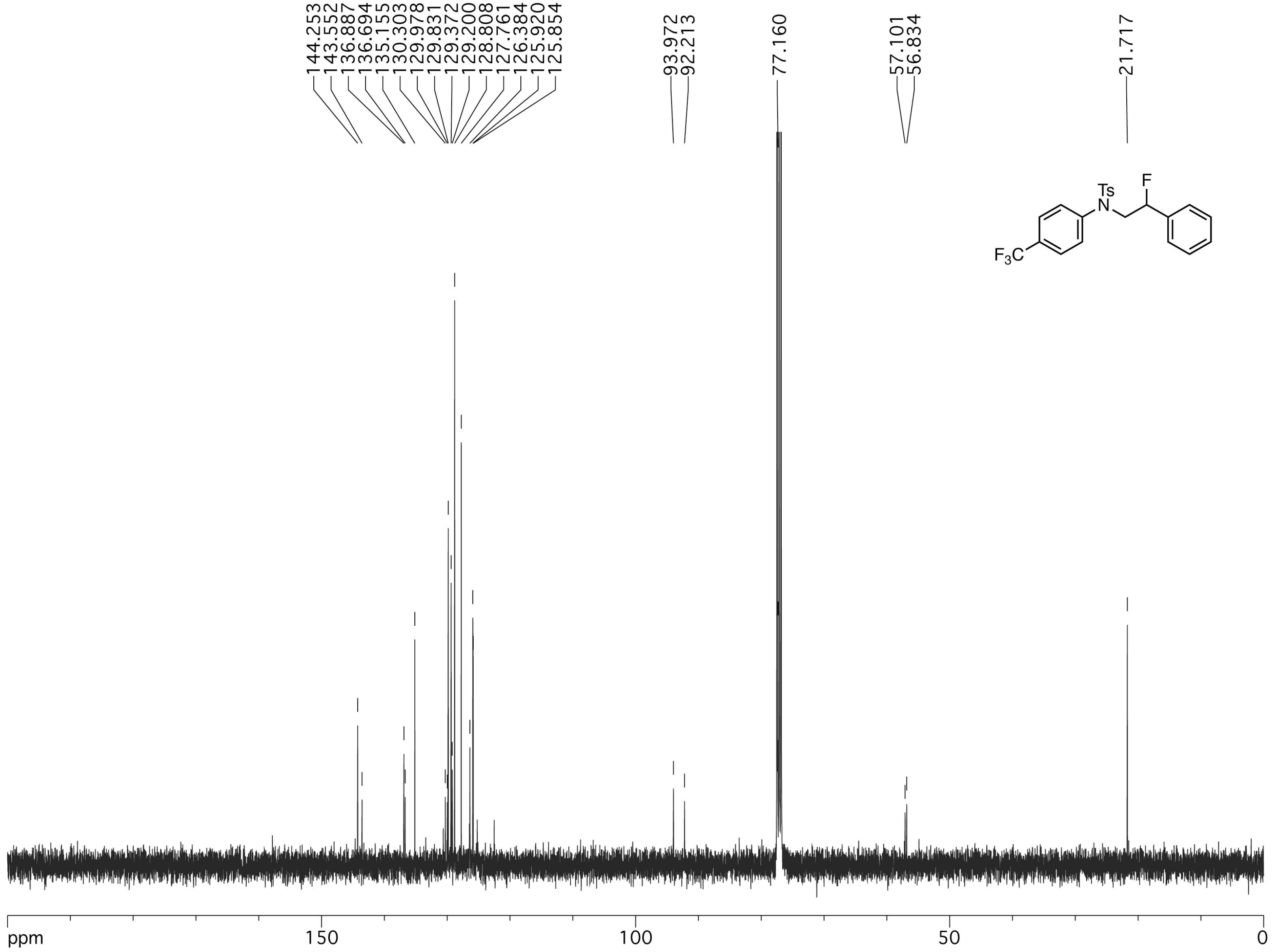

Figure S83. ^{13}C NMR spectrum of **6aa** in $CDCl_3$ (100 MHz) measured at 23 °C.

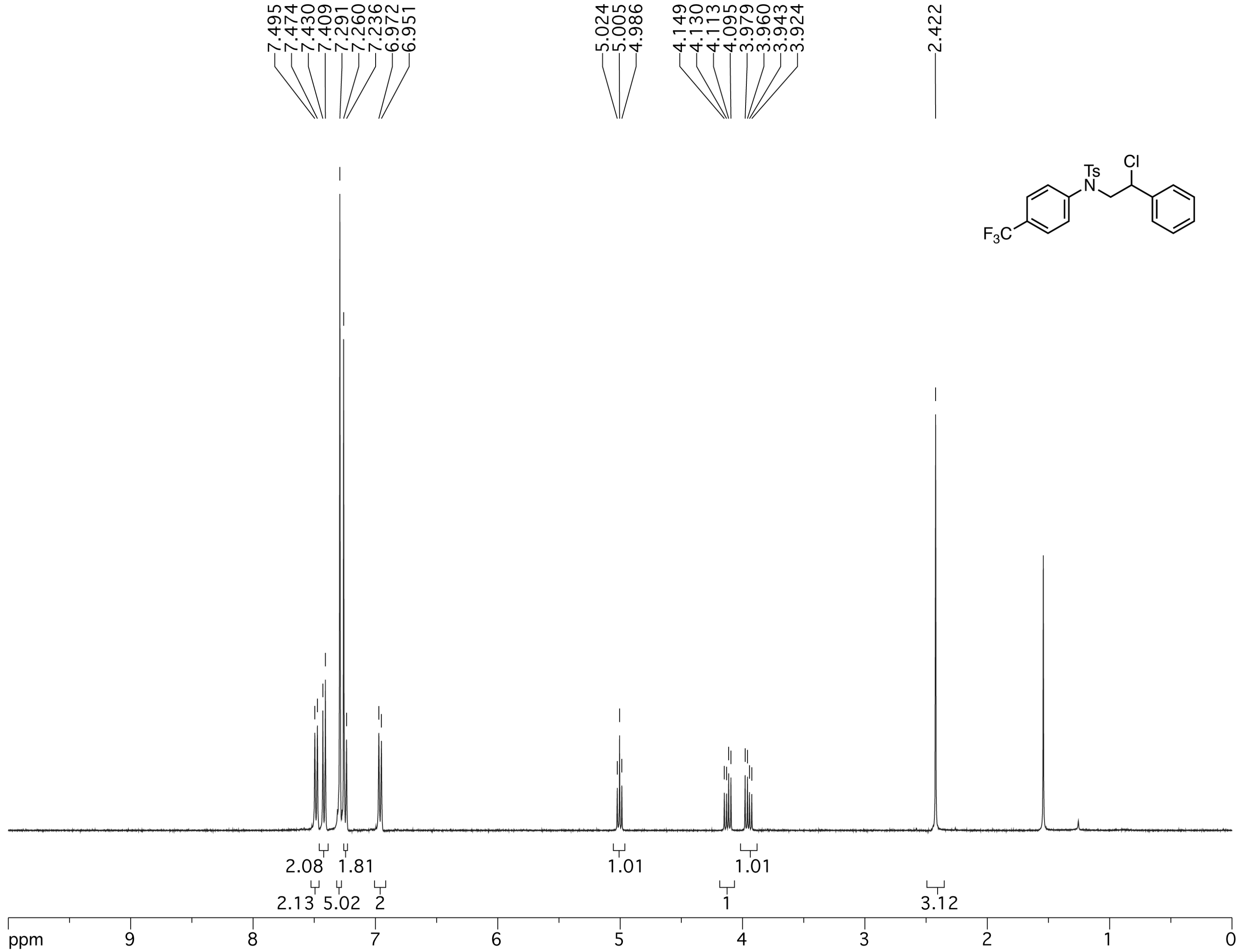

Figure S84. ^{1}H NMR spectrum of **6ab** in $CDCl_3$ (400 MHz) measured at 23 °C.

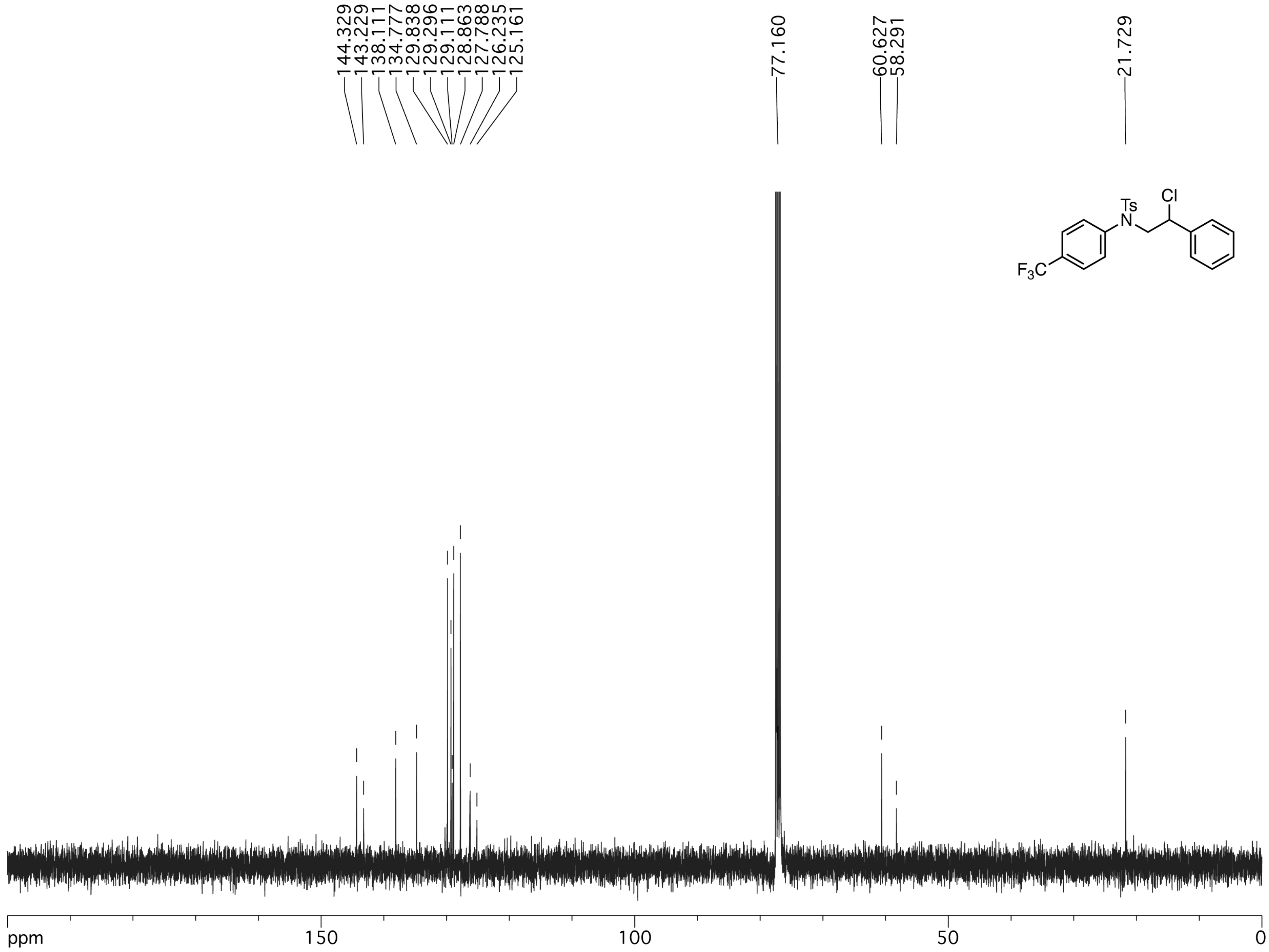

Figure S85. ^{13}C NMR spectrum of **6ab** in $CDCl_3$ (100 MHz) measured at 23 °C.

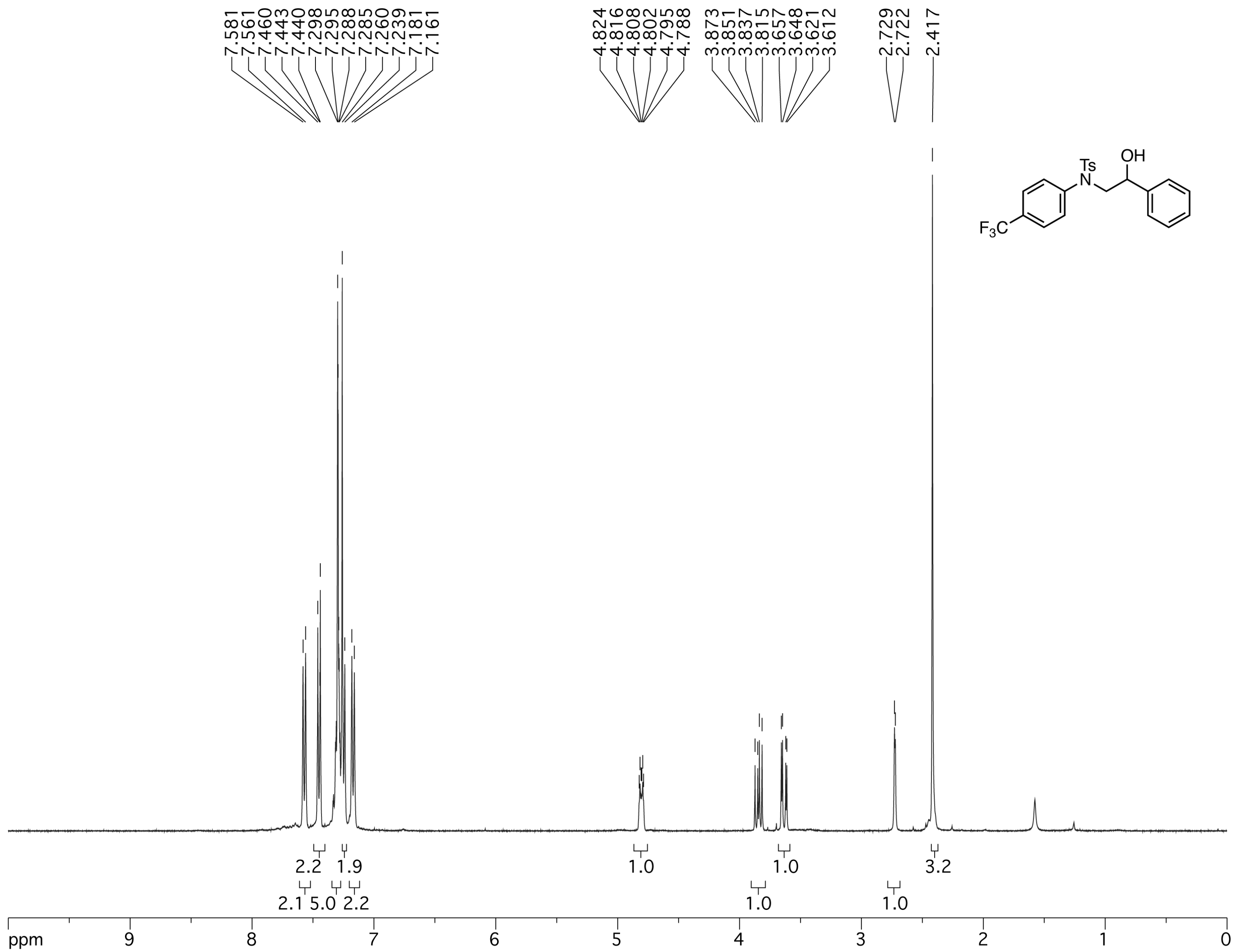

Figure S86. ^{1}H NMR spectrum of **6ac** in $CDCl_3$ (400 MHz) measured at 23 °C.

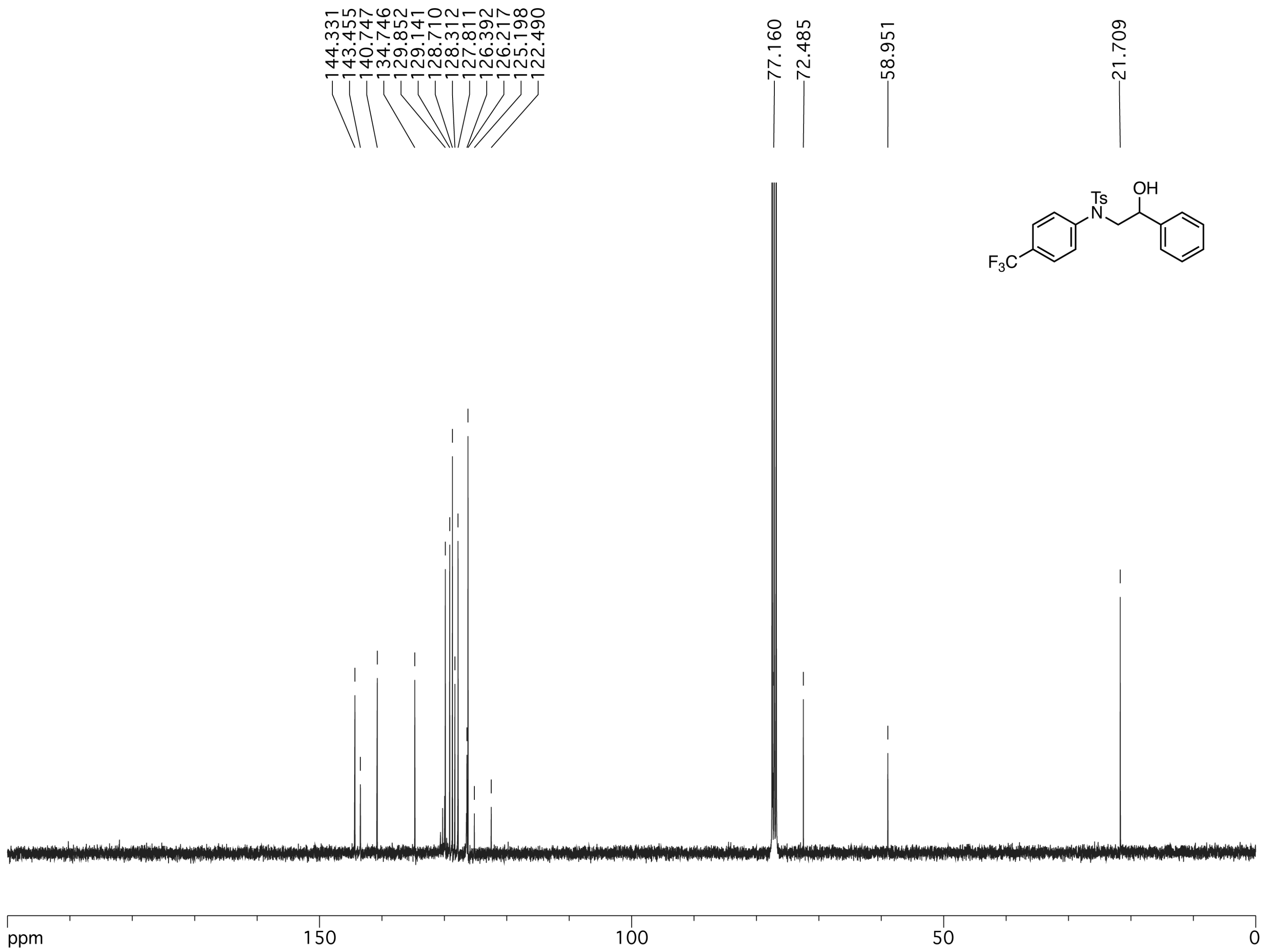

Figure S87. ^{13}C NMR spectrum of **6ac** in $CDCl_3$ (100 MHz) measured at 23 °C.

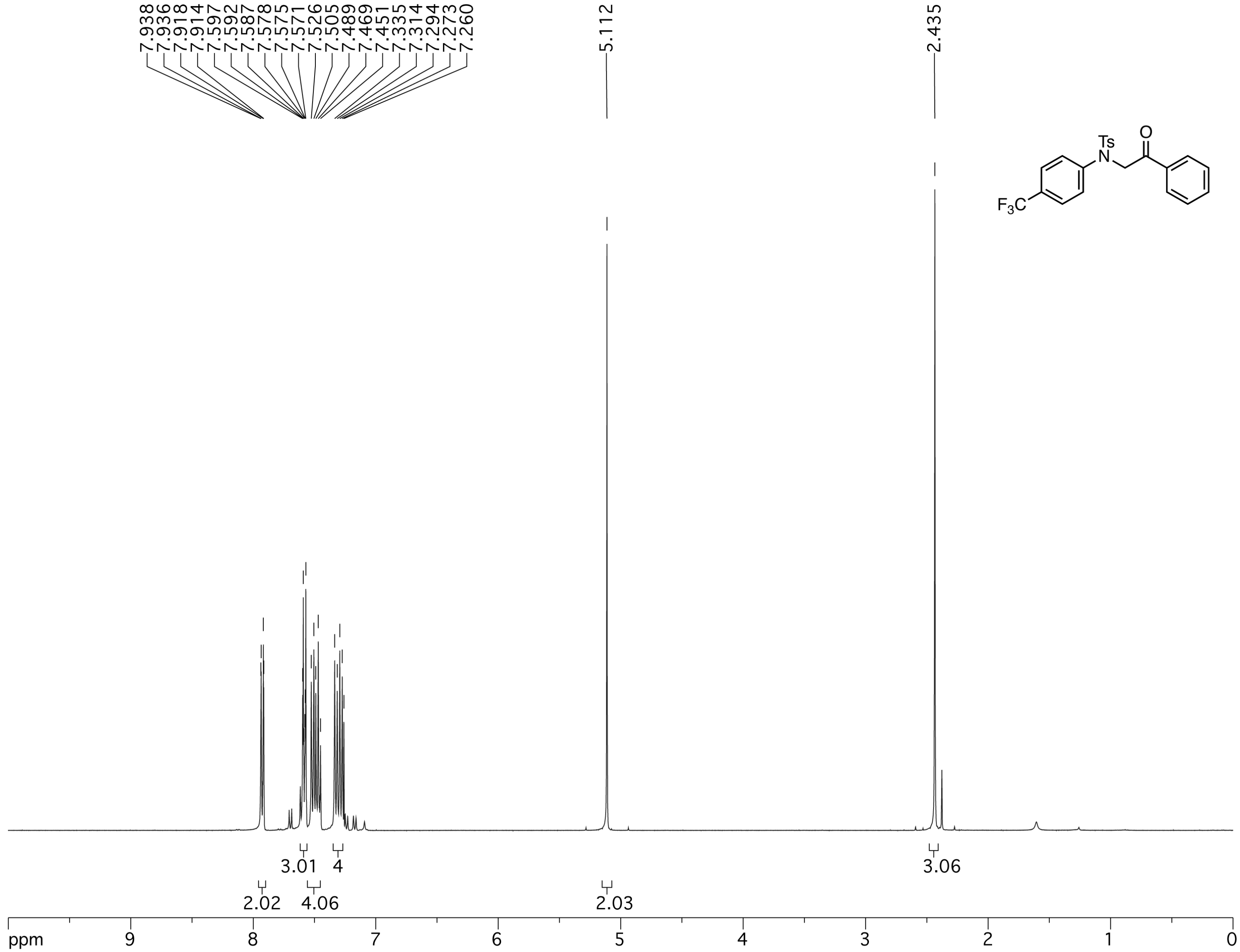

Figure S88. ^{1}H NMR spectrum of **6ad** in $CDCl_3$ (400 MHz) measured at 23 °C.

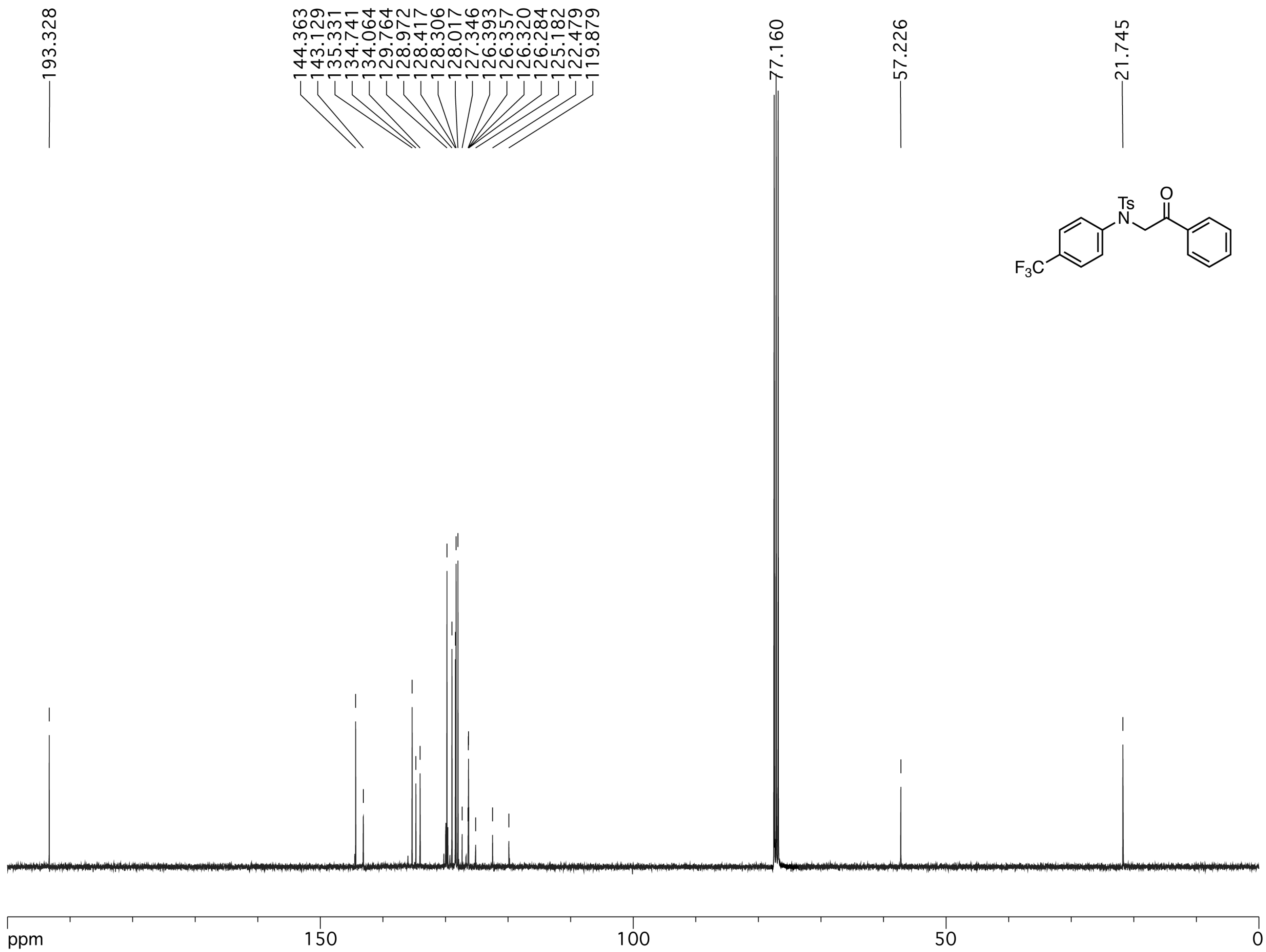

Figure S89. ^{13}C NMR spectrum of **6ad** in $CDCl_3$ (100 MHz) measured at 23 °C.

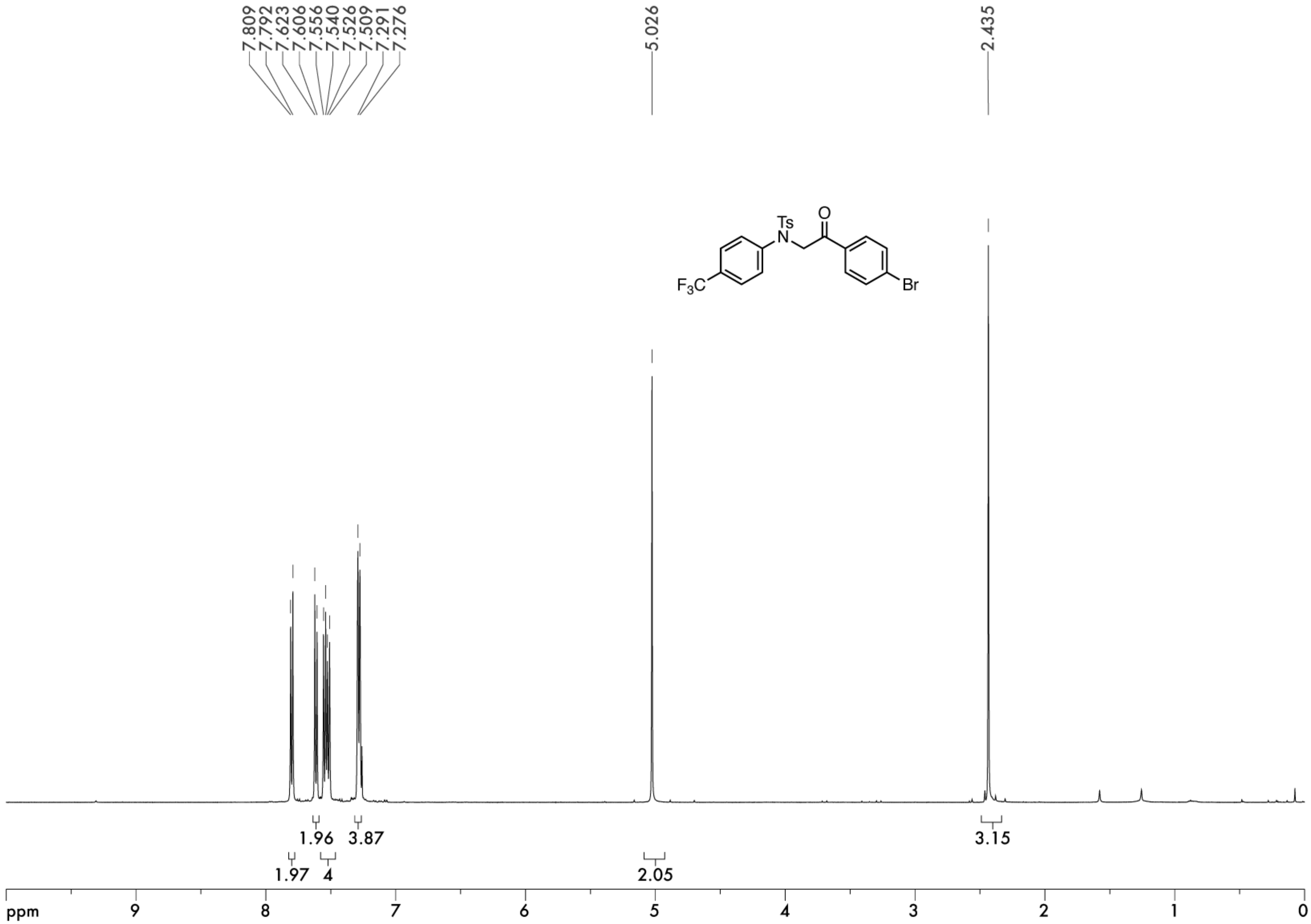

Figure S90. ¹H NMR spectrum of **6ae** in $CDCl_3$ (400 MHz) measured at 23 °C.

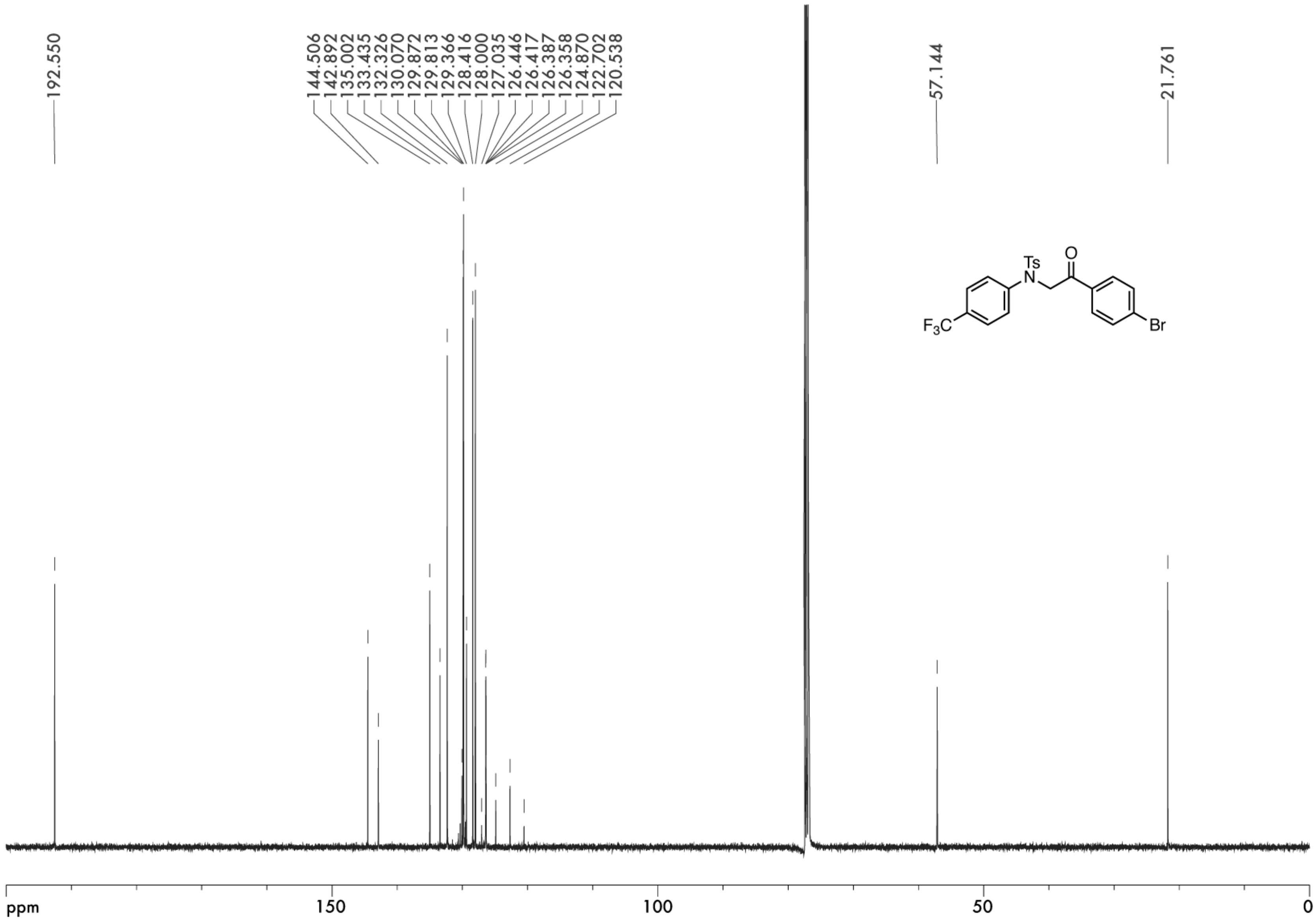

Figure S91. ^{13}C NMR spectrum of **6ae** in $CDCl_3$ (100 MHz) measured at 23 °C.

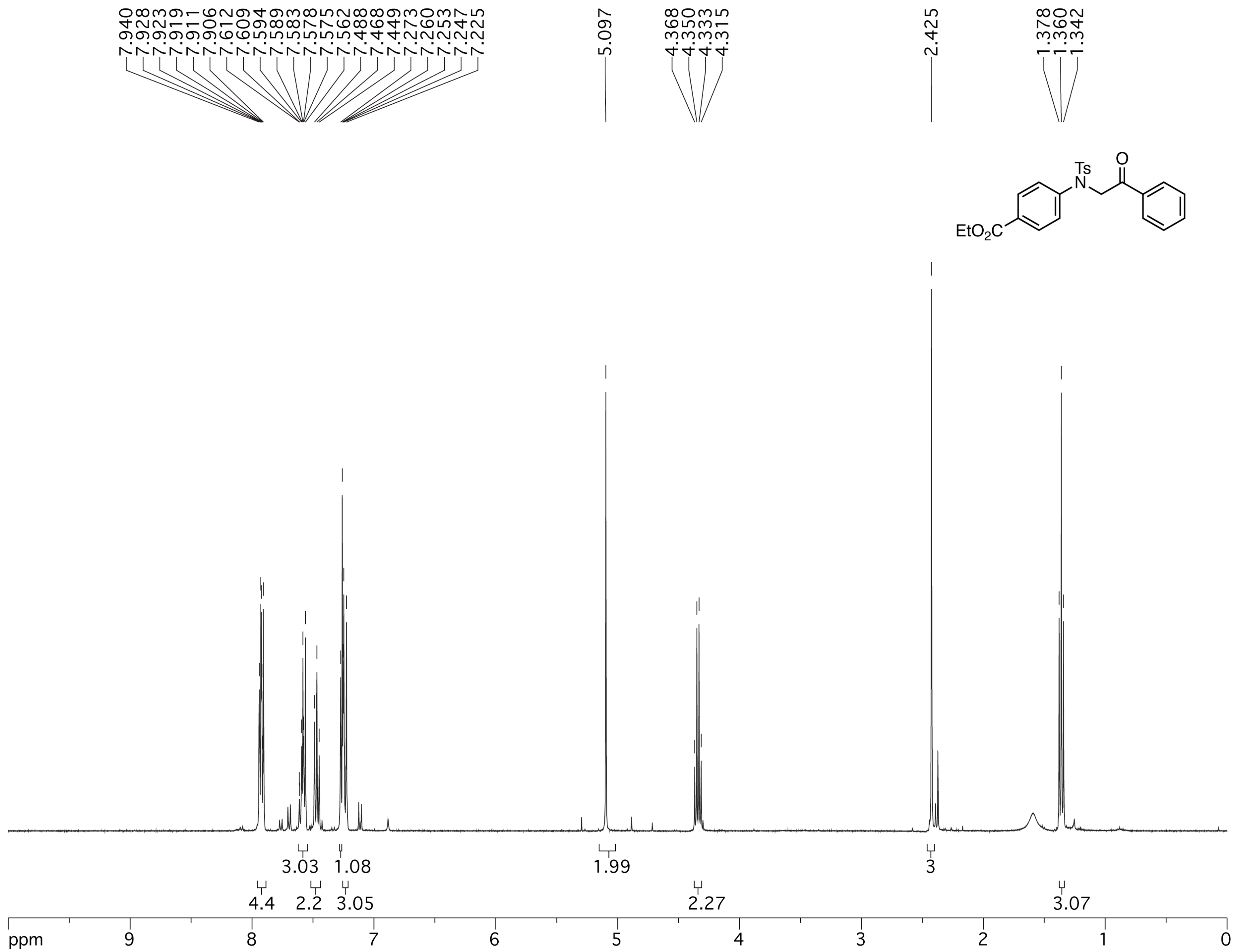

Figure S92. ^{1}H NMR spectrum of **6af** in $CDCl_3$ (400 MHz) measured at 23 °C.

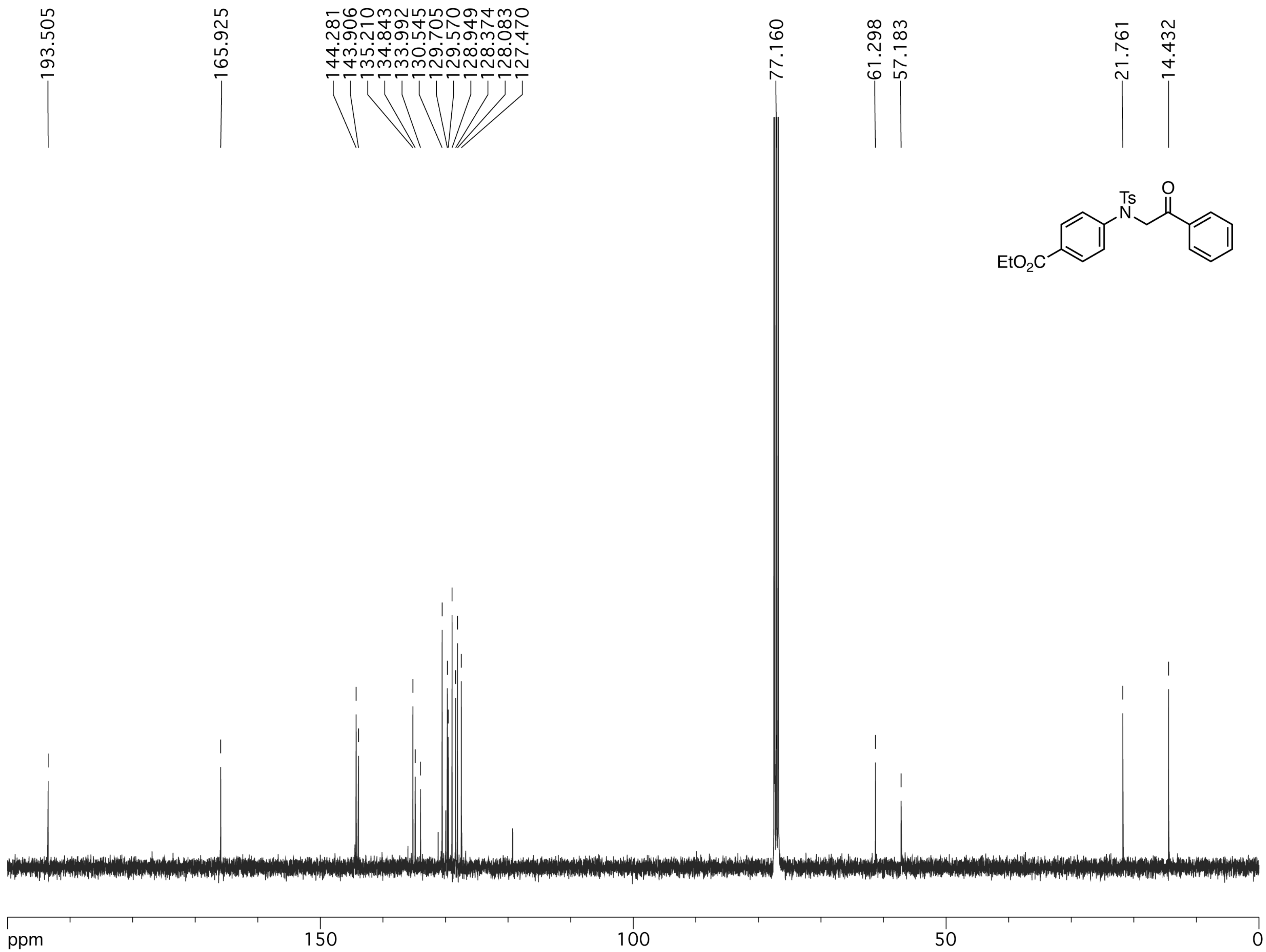

Figure S93. ^{13}C NMR spectrum of **6af** in $CDCl_3$ (100 MHz) measured at 23 °C.

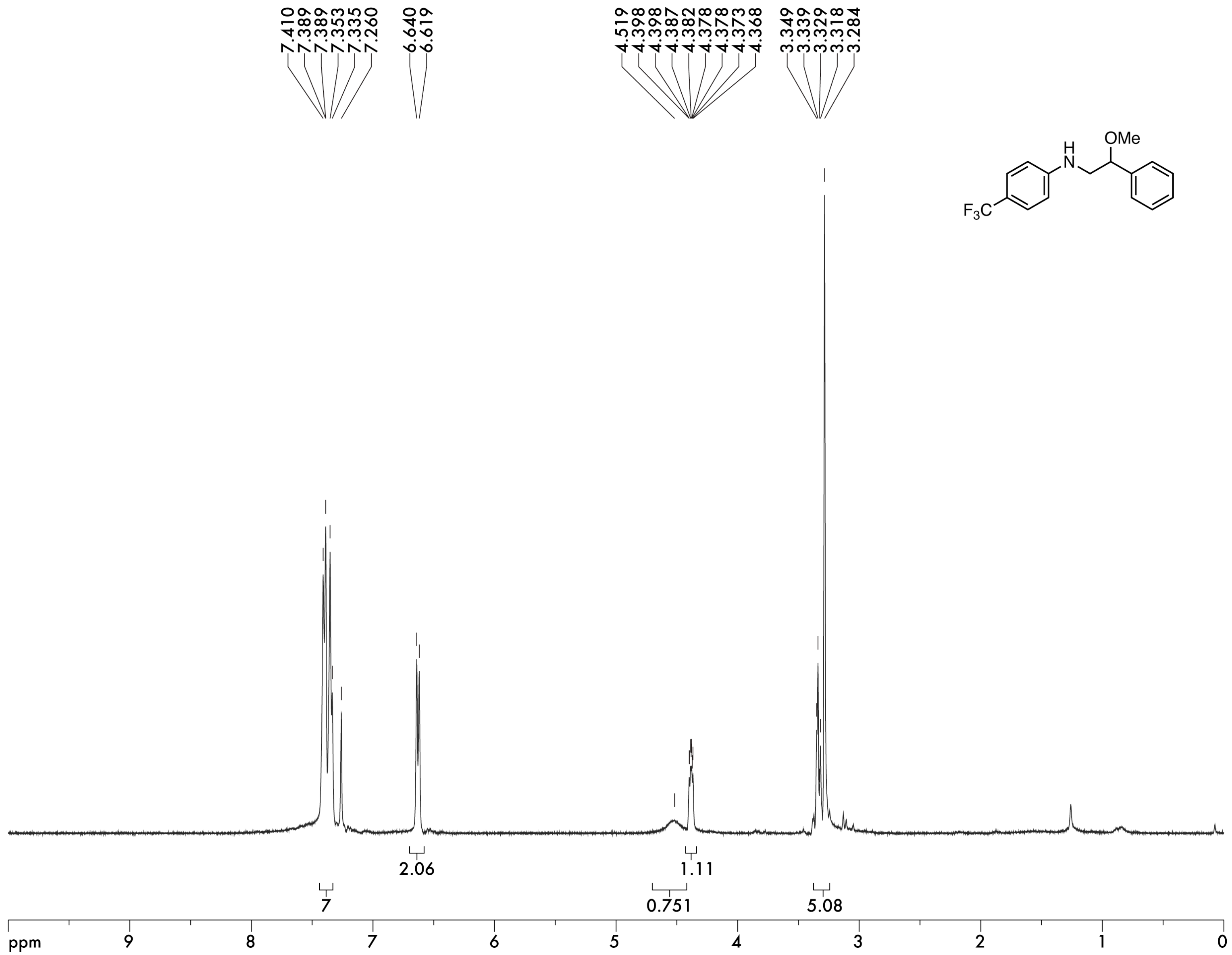

Figure S94. ^{1}H NMR spectrum of **6a'** in $CDCl_3$ (400 MHz) measured at 23 °C.

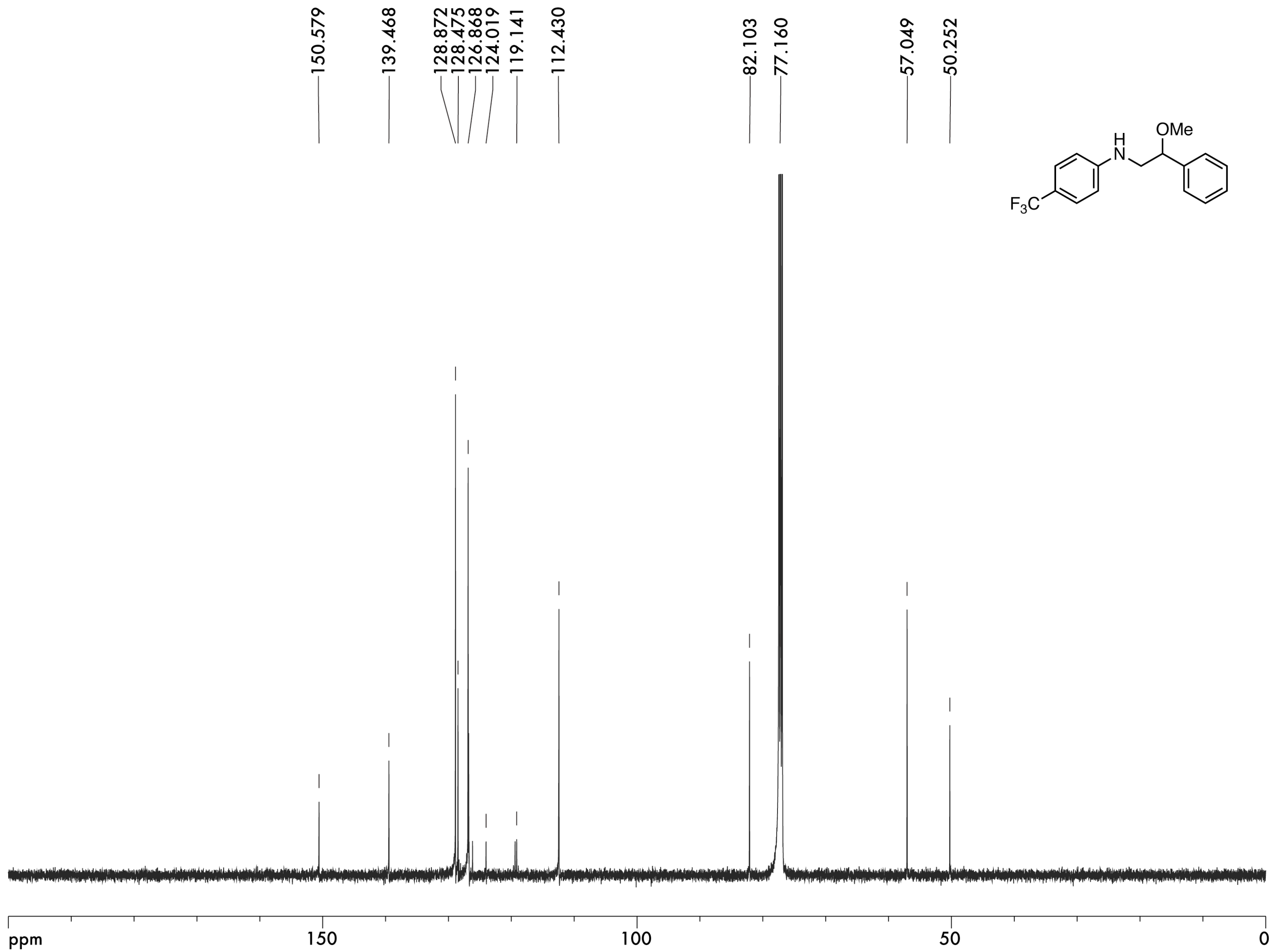

Figure S95. ^{13}C NMR spectrum of **6a’** in $CDCl_3$ (100 MHz) measured at 23 °C.

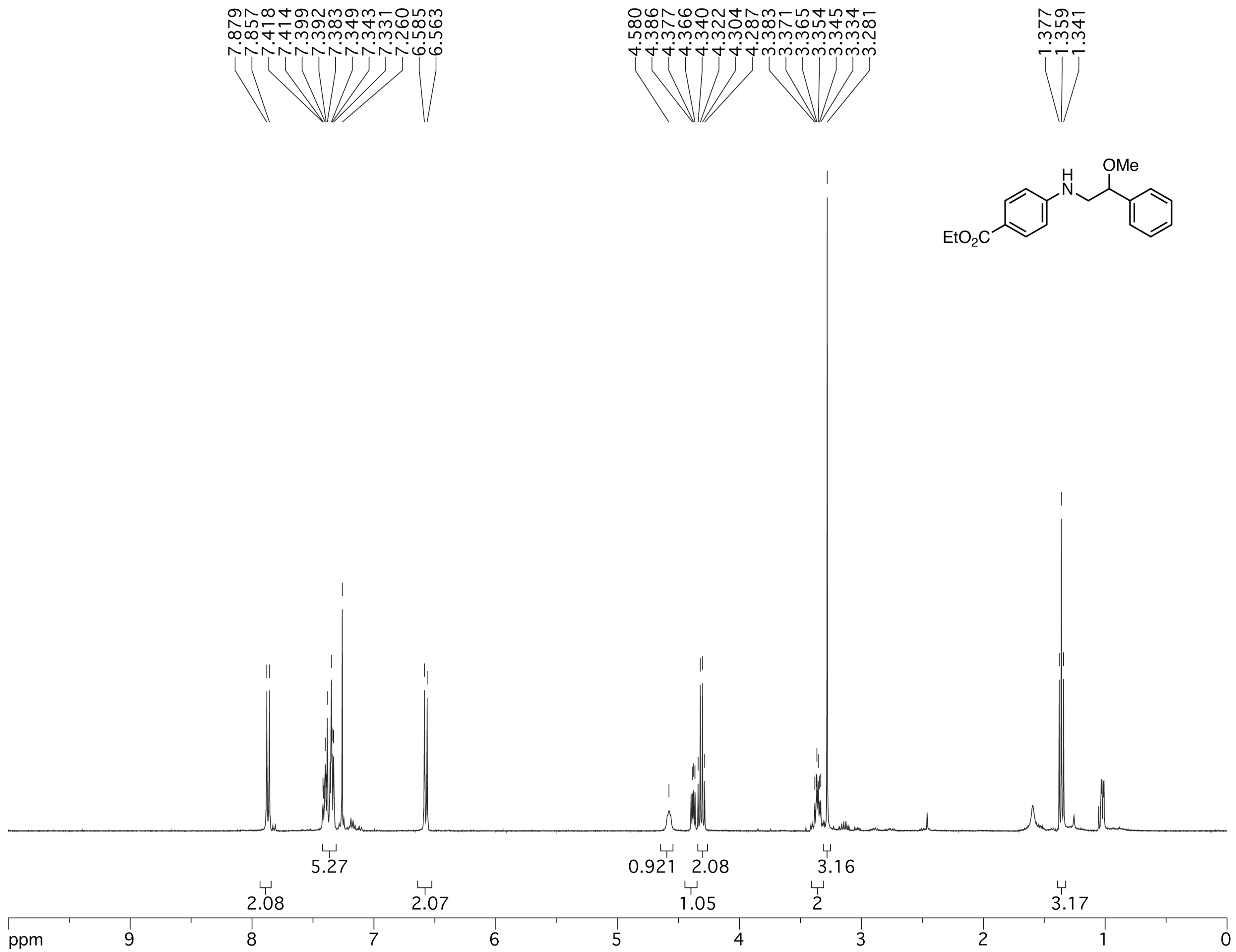

Figure S96. ^{1}H NMR spectrum of **6n'** in $CDCl_3$ (400 MHz) measured at 23 °C.

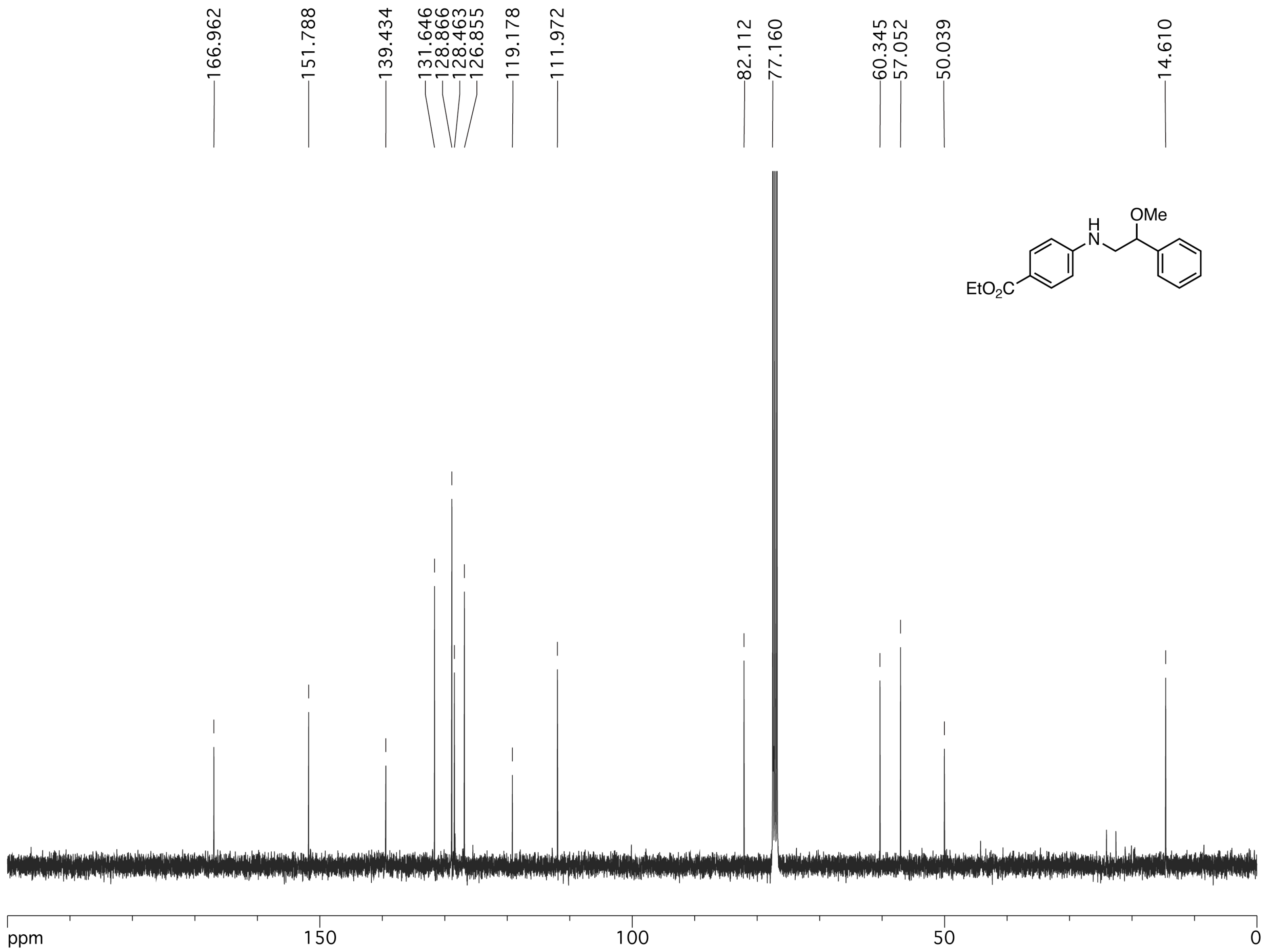

Figure S97. ^{13}C NMR spectrum of **6n'** in $CDCl_3$ (100 MHz) measured at 23 °C.

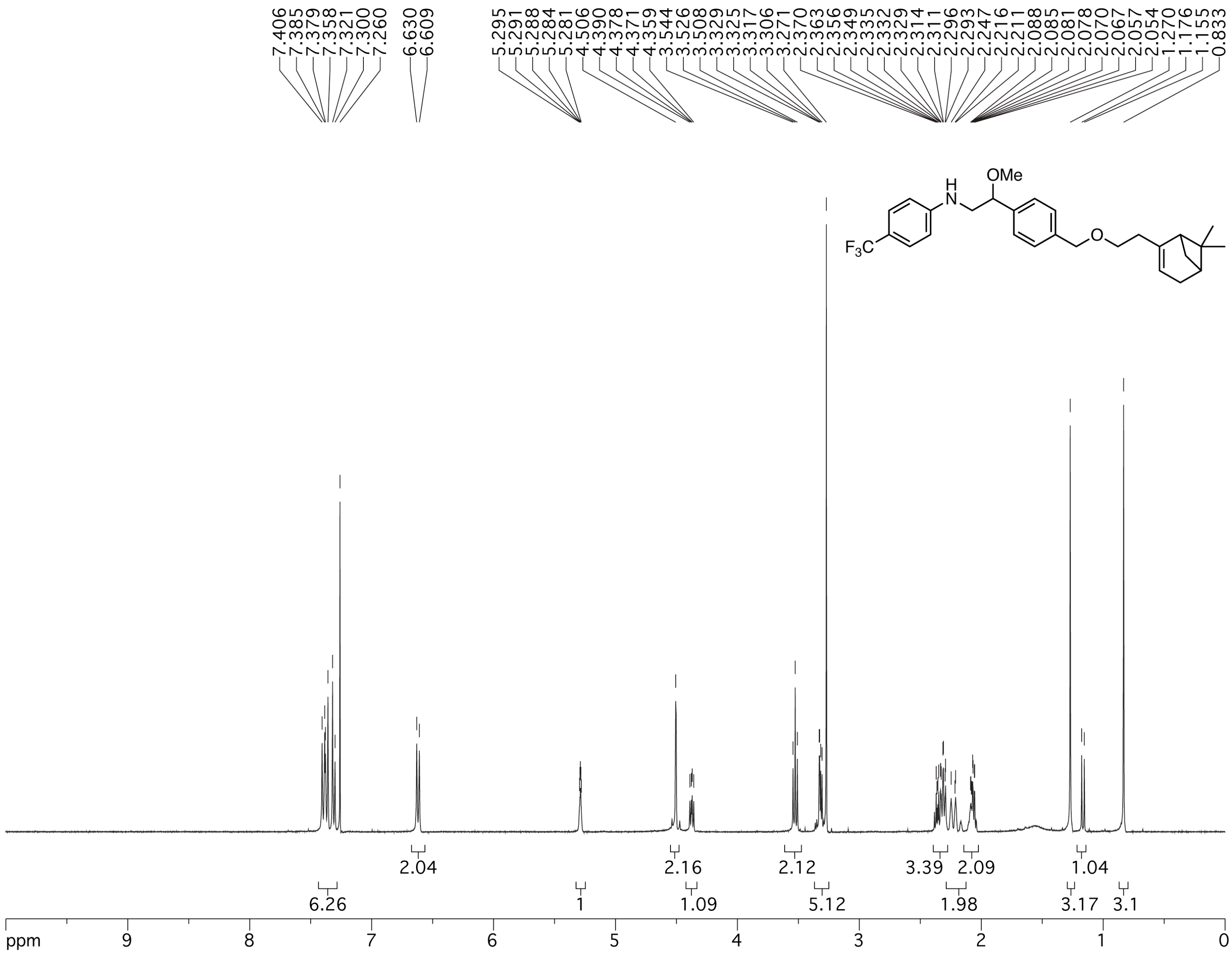

Figure S98. ^{1}H NMR spectrum of **6p'** in $CDCl_3$ (400 MHz) measured at 23 °C.

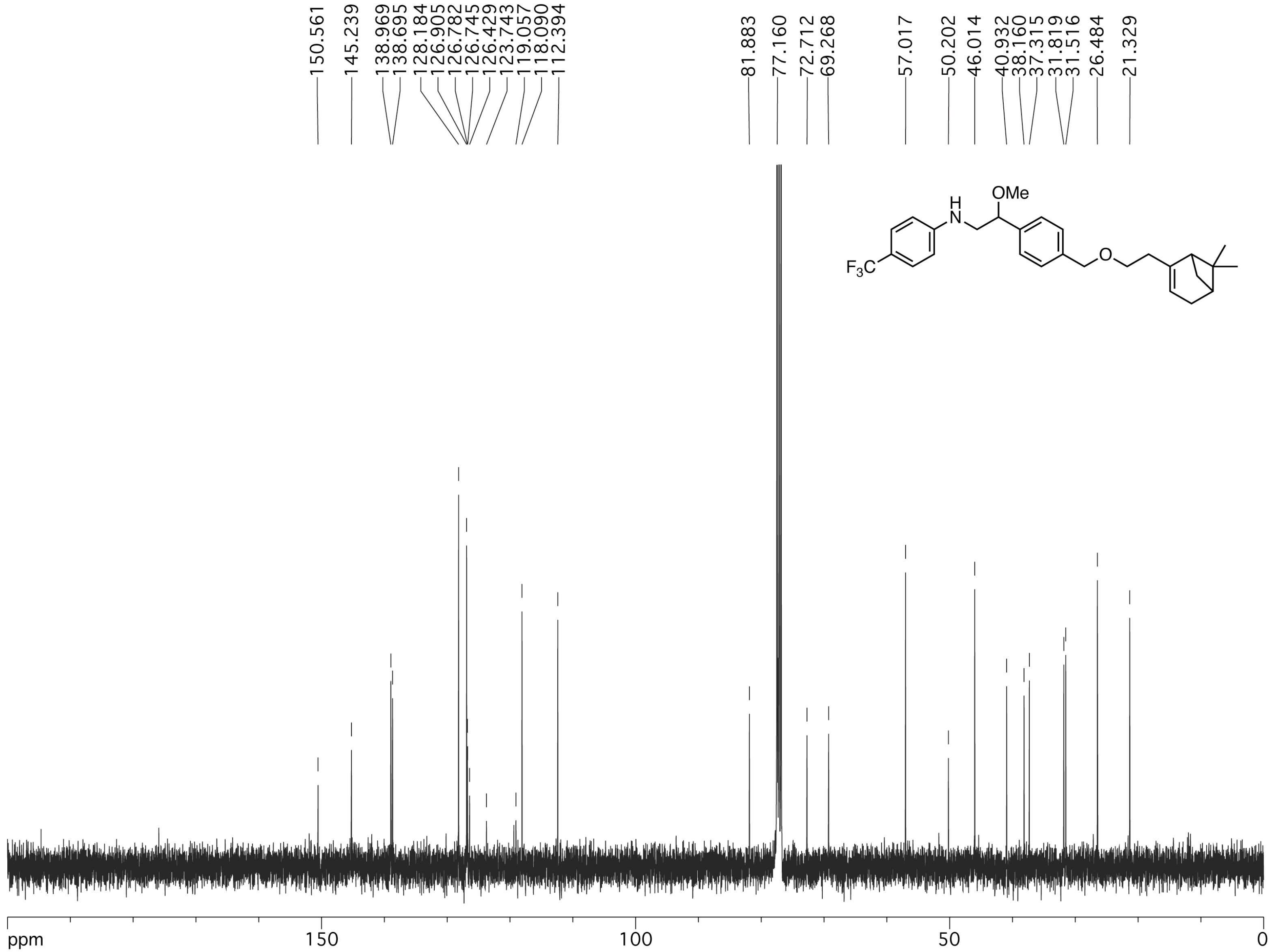

Figure S99. ^{13}C NMR spectrum of **6p'** in $CDCl_3$ (100 MHz) measured at 23 °C.

H. References

(1) Pangborn, A. B.; Giardello, M. A.; Grubbs, R. H.; Rosen, R. K.; Timmers, F. J. Safe and convenient procedure for solvent purification. *Organometallics* **1996**, *15*, 1518–1520.

(2) Roychowdhury, P.; Waheed, S.; Sengupta, U.; Herrera, R. G.; Powers, D. C. Synthesis of Secondary Amines via Self-Limiting Alkylation. *Org. Lett.* **2024**, *26*, 4926–4931.

(3) Liu, D.; Yang, K.; Fang, D.; Li, S.-J.; Lan, Y.; Chen, Y. Formyl Radical Generation from α-Chloro N-Methoxyphthalimides Enables Selective Aldehyde Synthesis. *Angew. Chem. Int. Ed.* **2023**, *62*, e202213686.

(4) Campbell, M. W.; Compton, J. S.; Kelly, C. B.; Molander, G. A. Three-Component Olefin Dicarbofunctionalization Enabled by Nickel/Photoredox Dual Catalysis. *J. Am. Chem. Soc.* **2019**, *141*, 20069–20078.

(5) Li, Z.; Yazaki, R.; Ohshima, T. Chemo- and Regioselective Direct Functional Group Installation through Catalytic Hydroxy Group Selective Conjugate Addition of Amino Alcohols to α,β-Unsaturated Sulfonyl Compounds. *Org. Lett.* **2016**, *18*, 3350–3353.

(6) Chen, Y.-X.; He, J.-T.; Wu, M.-C.; Liu, Z.-L.; Tang, K.; Xia, P.-J.; Chen, K.; Xiang, H.-Y.; Chen, X.-Q.; Yang, H. Photochemical Organocatalytic Aerobic Cleavage of C═C Bonds Enabled by Charge-Transfer Complex Formation. *Org. Lett.* **2022**, *24*, 3920–3925.

(7) Niu, D.; Buchwald, S. L. Design of Modified Amine Transfer Reagents Allows the Synthesis of α-Chiral Secondary Amines via CuH-Catalyzed Hydroamination. *J. Am. Chem. Soc.* **2015**, *137*, 9716–9721.

(8) Khan, I.; Reed-Berendt, B. G.; Melen, R. L.; Morrill, L. C. FLP-Catalyzed Transfer Hydrogenation of Silyl Enol Ethers. *Angew. Chem. Int. Ed.* **2018**, *57*, 12356–12359.

(9) Dolomanov, O. V.; Bourhis, L. J.; Gildea, R. J.; Howard, J. A.; Puschmann, H. OLEX2: a complete structure solution, refinement and analysis program. *J. Appl. Crystallogr.* **2009**, *42*, 339-341. Sheldrick, G. M. A short history of SHELX. *Acta Crystallogr. Sect. A* **2008**, *64*, 112-122.

(10) Sheldrick, G. M. Crystal structure refinement with SHELXL. *Acta Crystallogr. Sect. C* **2015**, *71*, 3–8.

(11) Mamillapalli, N. C.; Sekar, G. Metal free chemoselective reduction of α-keto amides using TBAF as catalyst. *RSC Adv.* **2014**, *4*, 61077–61085.

(12) Wu, Y.; Kim, D.; Teets, T. S. Photophysical Properties and Redox Potentials of Photosensitizers for Organic Photoredox Transformations. *Synlett* **2022**, *33*, 1154–1179.
